# Rh(iii)-catalyzed highly site- and regio-selective alkenyl C–H activation/annulation of 4-amino-2-quinolones with alkynes *via* reversible alkyne insertion†

**DOI:** 10.1039/d3sc03987k

**Published:** 2023-09-14

**Authors:** Naohiro Hirako, Takeshi Yasui, Yoshihiko Yamamoto

**Affiliations:** a Department of Basic Medicinal Sciences, Graduate School of Pharmaceutical Sciences, Nagoya University Furo-cho Chikusa Nagoya 464-8603 Japan t-yasui@ps.nagoya-u.ac.jp yamamoto-yoshi@ps.nagoya-u.ac.jp

## Abstract

3,4-Fused 2-quinolone frameworks are important structural motifs found in natural products and biologically active compounds. Intermolecular alkenyl C–H activation/annulation of 4-amino-2-quinolone substrates with alkynes is one of the most efficient methods for accessing such structural motifs. However, this is a formidable challenge because 4-amino-2-quinolones have two cleavable C–H bonds: an alkenyl C–H bond at the C3-position and an aromatic C–H bond at the C5-position. Herein, we report the Rh(iii)-catalyzed highly site-selective alkenyl C–H functionalization of 4-amino-2-quinolones to afford 3,4-fused 2-quinolones. This method has a wide substrate scope, including unsymmetrical internal alkynes, with complete regioselectivity. Several control experiments using an isolated key intermediate analog suggested that the annulation reaction proceeds *via* reversible alkyne insertion involving a binuclear Rh complex although alkyne insertion is generally recognized as an irreversible process due to the high activation barrier of the reverse process.

## Abstract 2

A practical approach for preparing 3,4-fused 2-quinolones has been disclosed. The Rh(iii)-catalyzed highly selective alkenyl C–H activation/annulation of 4-amino-2-quinolones was achieved *via* an unprecedented reversible alkyne insertion.

## Graphical abstract

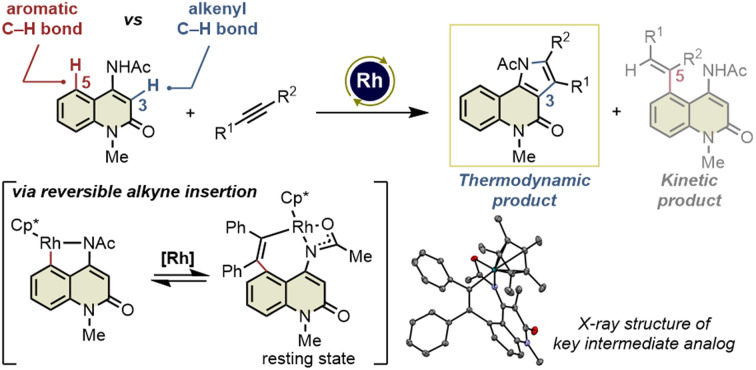

## Introduction

**Scheme 1:**
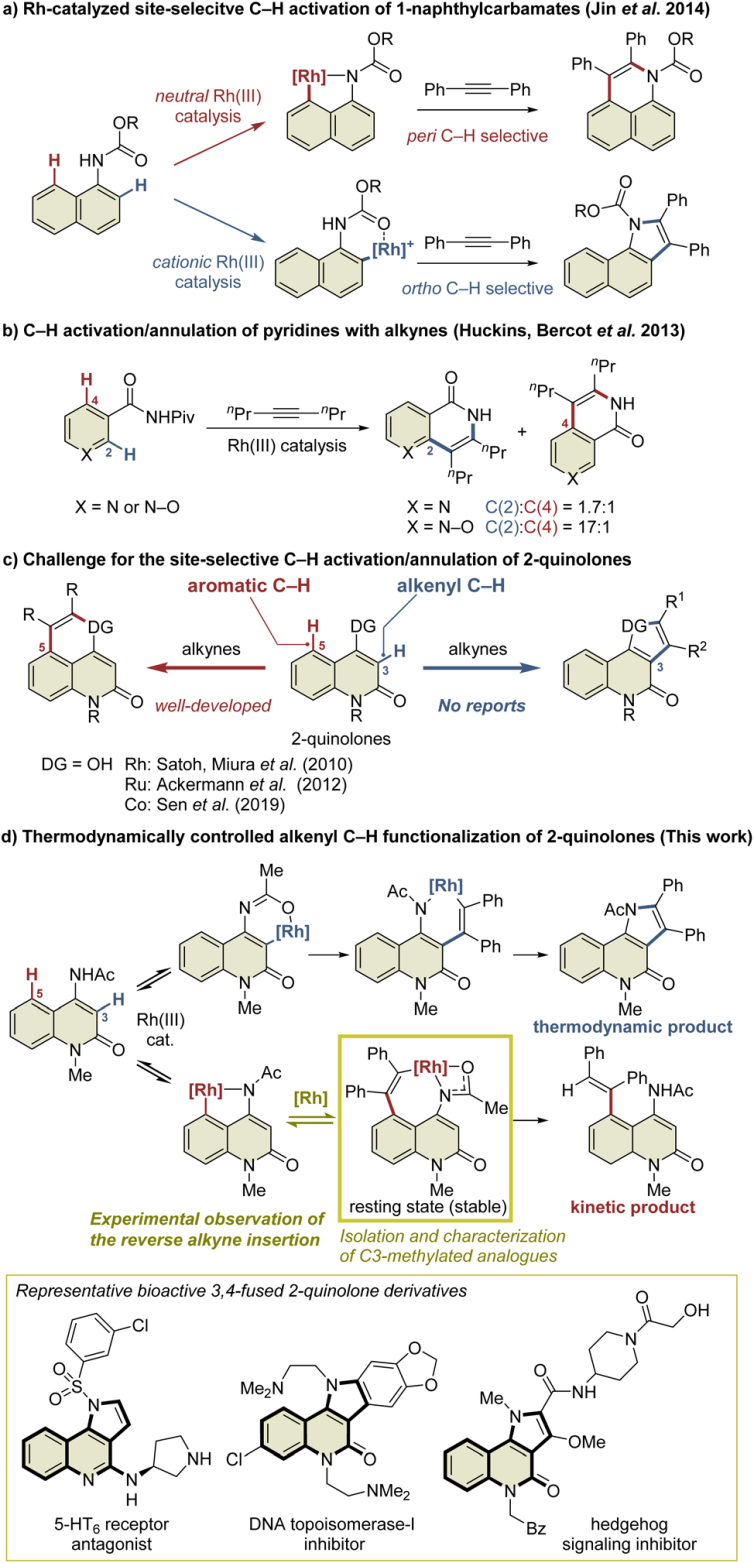
Site-selective C–H functionalization of aromatic compounds with two cleavable C–H bonds.

Transition-metal-catalyzed site-selective C–H functionalization with the aid of directing groups is one of the most powerful methods of accessing complex molecules from readily available starting materials with high efficiency.^1^ The Rh(iii)-catalyzed oxidative annulation of aromatic compounds with alkynes *via* C–H activation has been recognized as an efficient method of synthesizing fused carbo- and heterocycles such as naphthalenes, isoquinolones, isoquinolines, indoles, and isocoumarins.^2^ However, the site-selective C–H functionalization of aromatic compounds with multiple cleavable C–H bonds, such as the *ortho* and *peri* C–H bonds of the naphthalene ring, remains a challenge. Several research groups have addressed this issue by developing Rh(iii)-catalyzed site-selective functionalizations of such aromatic compounds.^3^ For example, Jin *et al.* reported the Rh(iii)-catalyzed site-selective C–H functionalization of 1-naphthylcarbamates (Scheme 1a).^4^ In this reaction, a neutral Rh(iii) catalyst enabled *peri* C–H activation *via* selective coordination to the carbamate nitrogen, while a cationic Rh(iii) catalyst selectively activated the *ortho* C–H bond *via* coordination to the carbamate oxygen in preference to the nitrogen due to its Lewis acidity. In contrast, the C–H functionalization of aromatic heterocycles is more complicated because these heterocycles usually have a greater electronic bias than aromatic carbocycles such as benzene and naphthalene.^5^ For example, Huckins, Bercot, *et al.* reported that the C–H functionalization of pyridine *N*-oxides proceeds selectively at the C2-position, whereas the reaction of the corresponding pyridine analog proceeds with poor site-selectivity (Scheme 1b).^6*a*^ The computational mechanistic study conducted by Huckins, Thiel, Houk, *et al.* elucidated that the electrostatic interaction between the substrates and alkynes is the pivotal factor affecting the selectivity, whereas the selectivity-determining step can be either C–H activation or alkyne insertion depending on alkynes used.^6*b*^ However, mechanistic studies to gain insight into the principles behind such selectivity in the C–H functionalization of aromatic heterocycles have rarely been conducted.

2-Quinolone derivatives fused at the C3- and C4-positions are important motifs found in natural products and biologically active substances.^7^ The annulation reaction of 2-quinolones involving C–H activation is one of the most efficient methods of preparing 3,4-fused 2-quinolones such as indoloquinolones.^8^ In particular, the intermolecular annulation of 2-quinolones is highly desirable, as substrates are readily available compared to those required for intramolecular reactions. However, 2-quinolones bearing a directing group at the C4-position possess two cleavable C–H bonds: an alkenyl C–H bond at the C3-position and an aromatic C–H bond at the C5-position (Scheme 1c). For selective access to 3,4-fused 2-quinolones, C3-selective C–H functionalization is required. As an effective method of managing this issue, Pd/norbornene-mediated C3-selective C–H functionalizations (the Catellani reaction) of 4-iodo-2-quinolones have recently been developed by the present authors and other research groups.^9,10^ However, the related annulation of 4-iodo-2-quinolones with activated alkynes, such as dimethyl acetylenedicarboxylate, can potentially afford both 3,4- and 4,5-fused 2-quinolone products.^11^ Thus, the C3-selective annulation of 2-quinolones with alkynes is a formidable challenge, whereas the C5-selective annulation has been well developed.^12^

Herein, we report the Rh(iii)-catalyzed highly selective alkenyl C–H functionalization of 4-amino-2-quinolones, providing access to 3,4-fused 2-quinolones (Scheme 1d). The mechanistic study suggested that the present C3-selective annulation reaction proceeds *via* an unprecedented reversible alkyne insertion. In general, the reverse process of alkyne insertion is recognized as an unfavorable process. In fact, only a few examples of β-carbon elimination of alkenylmetal species have been reported to date.^13^ Ishii *et al.* reported the β-carbon elimination of Ru or Rh complexes, and indicated that the β-carbon elimination was thermodynamically unfavorable.^13*b*,*c*^ On the other hand, in the previously reported Rh(iii)-catalyzed C–H activation/annulation reactions, the DFT calculations indicated that the β-carbon elimination of alkenylrhodium intermediates required relatively high activation energy, which is estimated to be more than +27 kcal mol^−1^ in most cases.^14^ Thus, subsequent processes such as reductive elimination are generally more facile to occur, and the β-carbon elimination of an alkenylrhodium complex is hard to observe, even if possible. Nevertheless, in this study, we have succeeded in the preparation of a key alkenylrhodium intermediate analog and the observation of the β-carbon elimination involving a binuclear Rh complex.

The resulting product has a pyrroloquinolone scaffold, which is found in a number of bioactive compounds such as hedgehog signaling inhibitors,^7*b*^ 5-HT_6_ receptor antagonists,^7*d*^ and topoisomerase-I inhibitors.^7*e*^ As an efficient method for the construction of this scaffold, Cai *et al.* have reported the Cu-catalyzed tandem [3 + 2] cycloaddition/C–C coupling of *N*-methyl-*N*-(2-iodophenyl) propiolamides with isocyanides,^15*a*^ although preparation methods of pyrroloquinolone derivatives are still scarce and are mostly based on linear synthesis.^15^ However, the preparation of isocyanides generally requires several steps, most of which still rely on the dehydration of formamides with highly electrophilic dehydration reagents such as POCl_3_ and *p*-TsCl.^16^ In addition, this method is unsuitable for the collective synthesis of diverse products because lengthy synthetic manipulations are required for the introduction of different substituents on the pyrrole ring. Compared with this method, in the present Rh-catalyzed annulation reaction, numerous alkynes can be used as stable and readily available reagents, allowing quick access to diverse pyrroloquinolone derivatives from 4-amino-2-quinolones.

## Results and discussion

### Reaction development

This study was commenced with the reaction of 4-amino-*N*-Boc-2-quinolone (1a) using a cationic Rh(iii) catalyst ([Cp*RhCl_2_]_2_/AgSbF_6_) to realize C3-selective annulation with diphenylacetylene, based on Jin's report.^4^ However, the undesired C5-annulated product 5aa was selectively obtained in 65% yield, along with several unidentified products (Table 1, entry 1). The use of the neutral Rh(iii) catalyst ([Cp*RhCl_2_]_2_) also resulted in the selective formation of 5aa (entry 2). These results indicate that the principle for the site-selective annulation of 1-naphthalenylcarbamates with alkynes is not applicable to the present reaction. Surprisingly, however, the reaction of 4-amino-*N*-acetyl-2-quinolone (1b) using the neutral Rh(iii) catalyst afforded the desired C3-annulated product 3ba in 63% yield without the formation of C5-functionalized products (entry 3). An optimization study revealed that the use of benzotrifluoride as the solvent gave the highest yield of 3ba (entry 4). Notably, when Cp*Rh(OAc)_2_·H_2_O was used as the catalyst, 3ba was obtained in 65% yield, along with C5-functionalized product 4ba in 5% yield (entry 5); the latter might be generated *via* protodemetalation associated with increasing the amount of acetate derived from the catalyst. However, the reaction did not proceed when one equivalent of acetic acid was added to promote the formation of 4ba (entry 6).

**Table 1:** Selected optimization study^a^

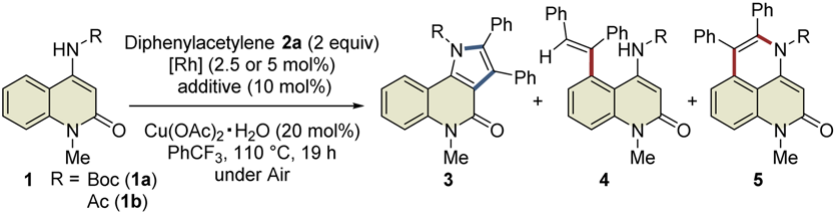						
Entry	1	Additive	3 (%)	4 (%)	5 (%)	RSM (%)
1^b^	1a	AgSbF_6_	<10	n.d.	65	0
2^c^	1a	—	n.d.	n.d.	78	0
3^c^^,^^d^	1b	—	63	n.d.	n.d.	0
4	1b	—	68	n.d.	n.d.	0
5^e^	1b	—	65	5	n.d.	0
6^f^	1b	—	n.d.	n.d.	n.d.	92

a Reaction conditions: 1 (0.2 mmol), 2a (0.4 mmol), [Cp*RhCl_2_]_2_ (2.5 mol%) or Cp*Rh(OAc)_2_·H_2_O (5 mol%), Cu(OAc)_2_·H_2_O (20 mol%), PhCF_3_ (2 mL). For details of the optimization study, see Tables S1 and S2. The isolated yields are reported. n.d. = not detected.

b Yields were determined by ^1^H NMR spectroscopy of the crude mixture.

c 1.2-Dichloroethane was used as the solvent instead of PhCF_3_.

d 2 equiv. of Cu(OAc)_2_·H_2_O was used under Ar atmosphere.

e Cp*Rh(OAc)_2_·H_2_O was used instead of [Cp*RhCl_2_]_2_.

f AcOH (1 equiv.) was added.

#### Substrate scope

**Scheme 2:**
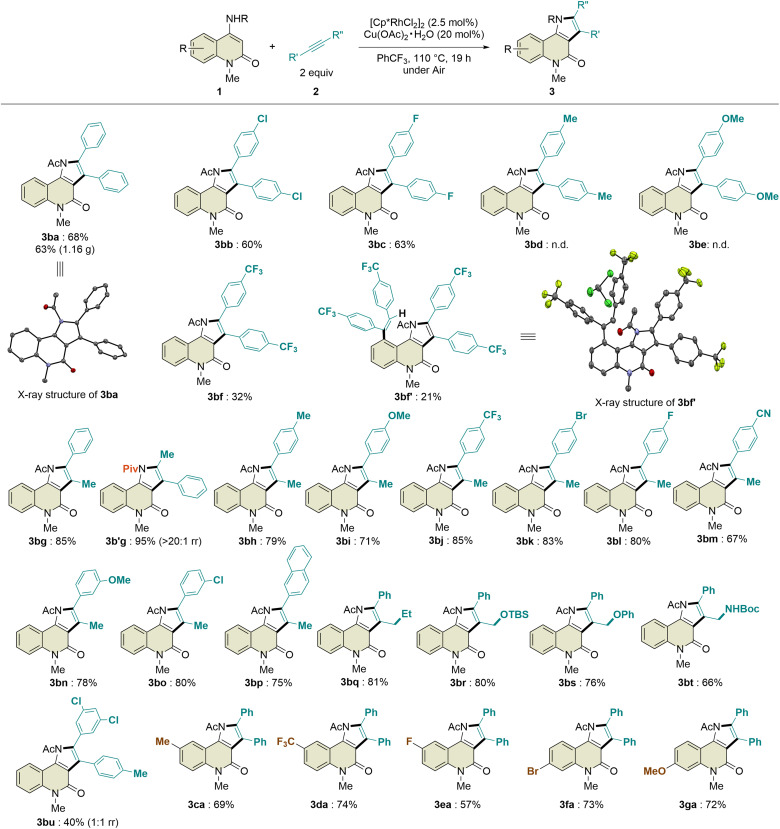
Substrate scope.

The scope of the C3-selective C–H bond activation/annulation reaction was explored under the optimal reaction conditions (Scheme 2). First, various symmetrical internal alkynes were investigated. Diarylacetylenes with halogen substituents, such as di(*p*-chlorophenyl)acetylene (2b) and di(*p*-fluorophenyl)acetylene (2c), readily reacted with 1b to afford the desired products 3bb and 3bc in 60% and 63% yields, respectively, whereas diarylacetylenes bearing electron-donating groups did not afford the corresponding products (3bd and 3be). Interestingly, the reaction of 1b with alkyne 2f, which has two trifluoromethyl groups, produced not only 3bf but also 3bf′. The structure of 3bf′ was unambiguously confirmed by X-ray crystallographic analysis. To our delight, the reaction using unsymmetrical internal alkynes 2g–t proceeded smoothly to afford the corresponding products 3bg–bt as single regioisomers in 66–85% yields. In contrast, the use of unsymmetrical internal alkyne 2u possessing 3,5-dichlorophenyl and 4-methylphenyl groups resulted in the yield of 3bu with a 1 : 1 regioisomeric ratio. In addition, 4-octyne and phenylacetylene were not tolerated in this transformation. Regarding the substituents on the benzene ring of the 2-quinolone scaffold, methyl, trifluoromethyl, fluoro, bromo, and methoxy groups at the C6- or C7-positions were compatible with this reaction, affording the desired products 3ca–ga in 57–74% yields with excellent site-selectivity. Remarkably, the introduction of a pivaloyl group instead of the acetyl group in 1b led to the formation of 3b′g in 95% yield with excellent regioselectivity (>20 : 1 rr), which is probably due to the steric hindrance between the *tert*-butyl and phenyl groups. To evaluate the potential utility of this reaction, we performed a scale-up experiment, which produced 1.16 g of 3ba without a significant decrease in the yield (63%).

#### Mechanistic study

**Scheme 3:**
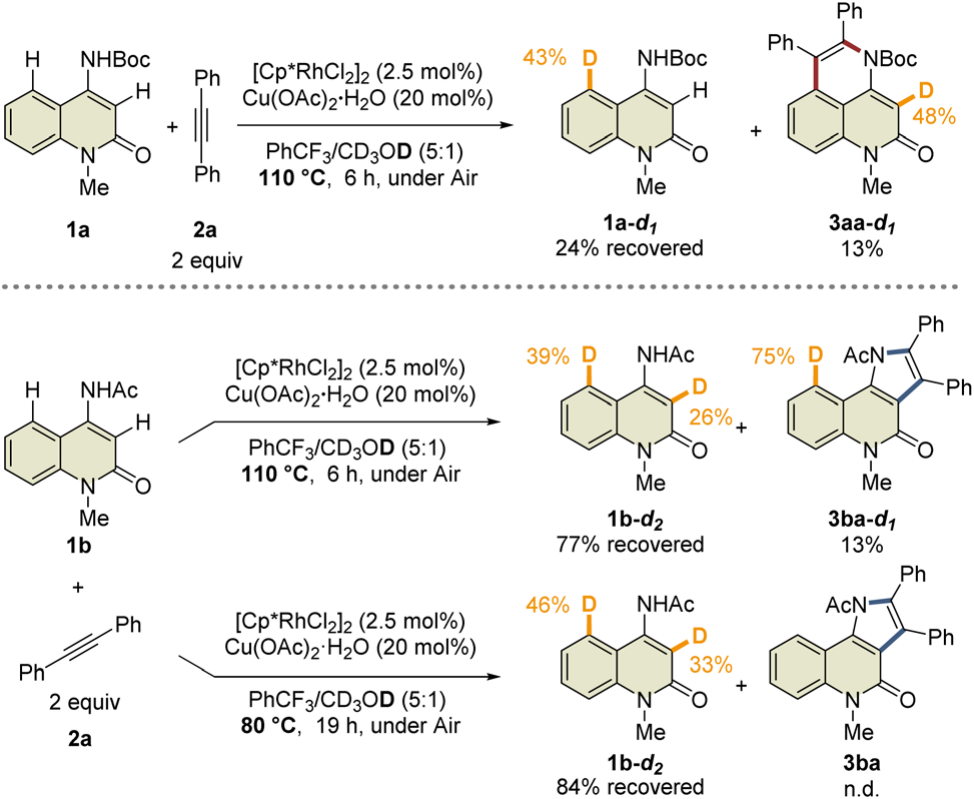
Analysis of hydrogen/deuterium exchange.

To gain insight into the reaction mechanism, several control experiments were performed. First, 1a was subjected to the standard reaction conditions in the presence of excess methanol-*d*_4_ (Scheme 3). Deuterium incorporation was observed only at the C5-position, indicating that the site-selectivity was determined during the C–H activation step. In sharp contrast, hydrogen/deuterium exchange with 1b led to deuterium incorporation at both the C3- and C5-positions, indicating that these C–H bonds could be reversibly cleaved and that C–H activation was not the selectivity-determining step. It was also determined that the C–H activation step was not turnover-limiting because deuterium incorporation was observed even at 80 °C, where the reaction did not proceed.

**Fig. 1:**
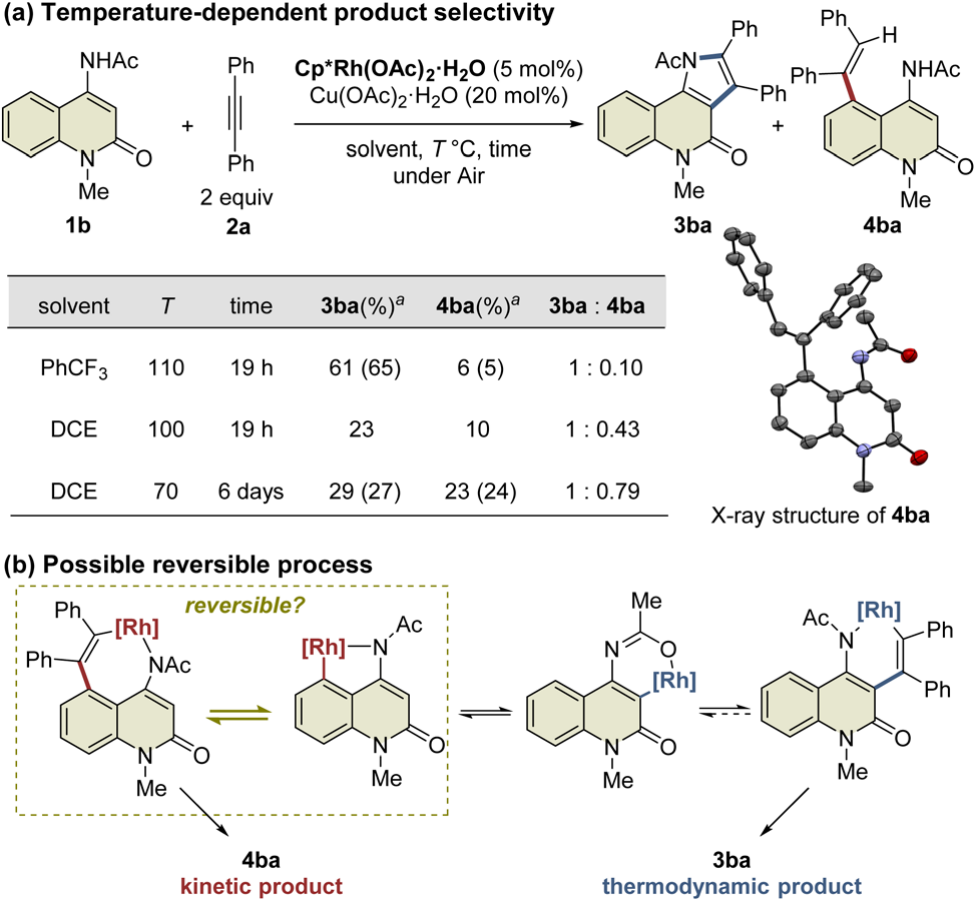
Temperature-dependence of the reaction. ^*a*^Yields are determined by ^1^H NMR. Isolated yields are shown in parentheses.

During the reaction optimization study, we found that not only C3-functionalized product 3ba but also C5-functionalized product 4ba were obtained when Cp*Rh(OAc)_2_·H_2_O was used as the catalyst (Table 1, entry 5), indicating that alkyne insertion can also occur at the C5-position. Interestingly, lowering the reaction temperature to 100 °C led to an increase in the ratio of 4ba to 3ba (Fig. 1a). Furthermore, the same reaction conducted at 70 °C for 6 d afforded 3ba and 4ba in 29% and 23% NMR yields, respectively. These results clearly show that 3ba was formed under thermodynamic control, whereas 4ba was formed under kinetic control. Based on these results, we assumed that the alkyne insertion into the five-membered rhodacycle intermediate generated by C5–H activation is a reversible process (Fig. 1b). Although the β-carbon elimination from an alkenylmetal complex is very rare, Ishii *et al.* recently reported that a related seven-membered rhodacycle undergoes an exchange reaction of the alkenyl unit through β-carbon elimination.^13*c*^ Similar alkyne deinsertion could also occur in the present reaction system.

**Fig. 2:**
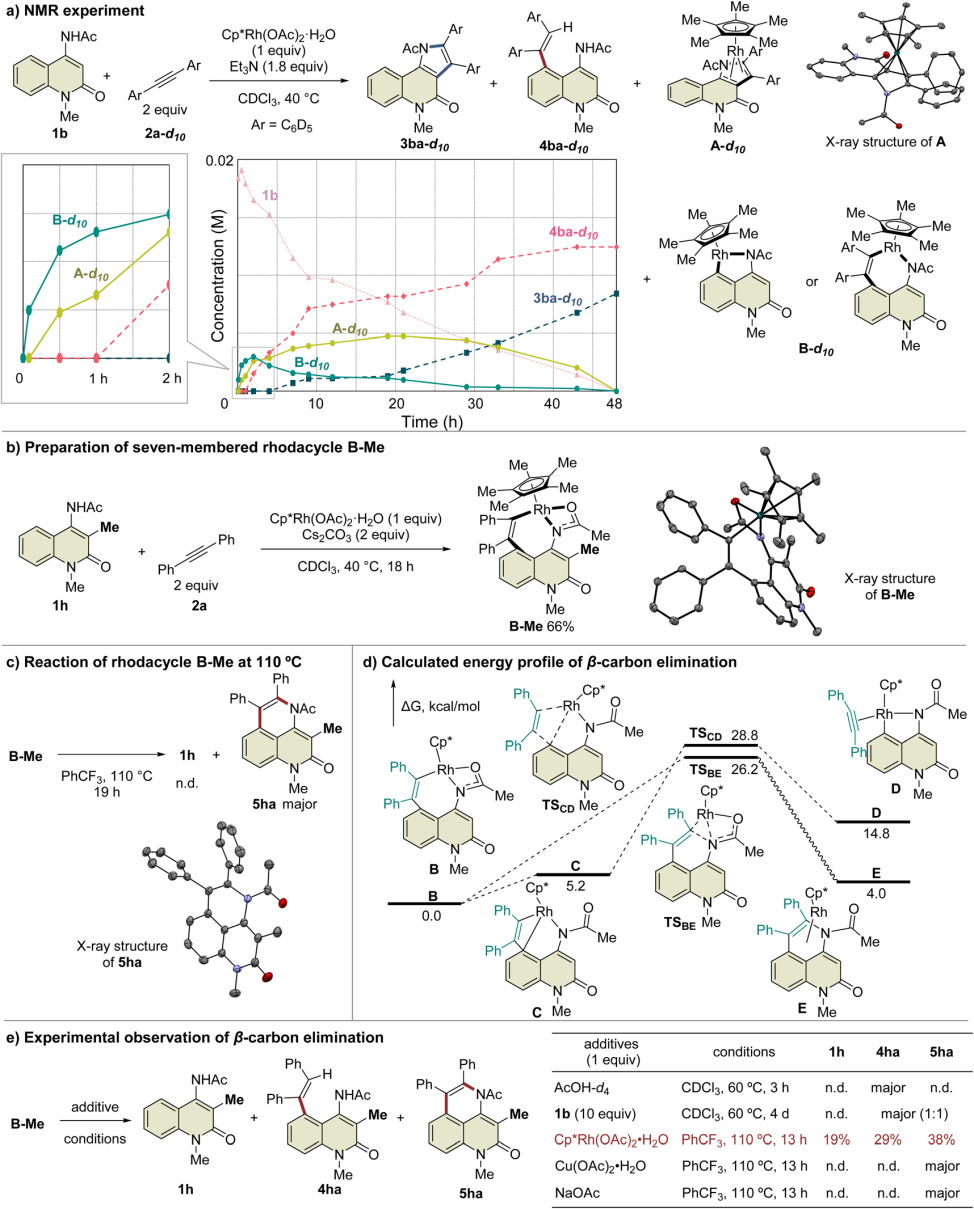
Mechanistic study involving rhodacycle intermediates.

To prove this hypothesis, we performed an NMR experiment using 1b and 2a-*d*_10_ in the presence of a stoichiometric amount of Cp*Rh(OAc)_2_·H_2_O as a preliminary investigation. Triethylamine was used as an additive to suppress the protodemetalation. When we monitored the progress of the reaction at 40 °C in CDCl_3_, two different species, which might be rhodacycle intermediates, other than 3ba-*d*_10_ and 4ba-*d*_10_, were observed immediately after initiation of the reaction (Fig. 2a). To our delight, one of these intermediates was isolated by silica gel column chromatography, and X-ray crystallographic analysis revealed the structure to be the rhodium sandwich complex A-*d*_10_ with an *η*^4^-pyrrole ligand. However, another intermediate B-*d*_10_ could not be isolated because of its instability. In the NMR experiment, a distinctive ^1^H NMR signal of the alkenyl proton derived from intermediate B-*d*_10_ was observed at *δ* 6.35 ppm as a singlet (Fig. S7†). In addition, the corresponding signal of the Cp* methyl groups was also observed at *δ* 1.32 ppm as a singlet. Based on these observations, we assumed that intermediate B-*d*_10_ might be a five-membered rhodacycle generated by C5–H activation or a seven-membered rhodacycle formed by subsequent alkyne insertion. Reaction monitoring by ^1^H NMR revealed the formation of a small amount of rhodacycle B-*d*_10_ over a long period of time, suggesting that this intermediate might be in a resting state and gradually evolved into 4ba-*d*_10_*via* protodemetalation or 3ba-*d*_10_ through the regeneration of 1b in equilibrium. To obtain further information regarding intermediate B, we designed a C3-methylated 2-quinolone 1h to prevent the C3-metalation, in order to selectively prepare the corresponding rhodacycle intermediate (Fig. 2b). The treatment of 1h with Cp*Rh(OAc)_2_·H_2_O (1 equiv.) and 2a (2 equiv.) at 40 °C in CDCl_3_ led to a rapid and quantitative formation of rhodacycle B-Me, for which the ^1^H NMR signals are similar to those of intermediate B observed in the NMR experiment (Fig. S7†). Complex B-Me was isolated in 66% yield and X-ray crystallographic analysis unambiguously revealed the structure to be a seven-membered rhodacycle. This rhodium complex adopted a three-legged piano stool geometry, in which the amidate moiety coordinates to the Rh center as a *κ*^2^-N,O bidentate ligand.

With the isolated key intermediate analog B-Me in hand, we sought to observe the β-carbon elimination experimentally. First, complex B-Me was heated to 110 °C in benzotrifluoride; however, 1h was not obtained, and reductive elimination proceeded to afford 5ha as the main product (Fig. 2c). To estimate the activation energy for the β-carbon elimination process, DFT calculations were performed at the SMD (DCE) B3LYP-D3(BJ)/6-311++G(d,p)-SDD//B3LYP-D3(BJ)/6-31G(d)-LanL2DZ level of theory. The calculated activation energy for the β-carbon elimination was 28.8 kcal mol^−1^, whereas that for reductive elimination was 26.2 kcal mol^−1^, indicating that it is difficult for β-carbon elimination to proceed before reductive elimination (Fig. 2d). Accordingly, we hypothesized that a mediator is required to promote the β-carbon elimination. Several additives utilized in the present catalytic reaction were examined (Fig. 2e and Table S3†). The addition of AcOH-*d*_4_ resulted in protodemetalation to afford only 4ha. When an excess amount of 1b was used, 1 : 1 mixture of 4ha and 5ha were observed while 1h was not detected. Finally, we found that the addition of Cp*Rh(OAc)_2_·H_2_O (1 equiv.) promoted β-carbon elimination from B-Me to afford 1h in 19% yield, along with the formation of 4ha and 5ha, although the addition of Cu(OAc)_2_ or NaOAc as an acetate source resulted in the formation of only 5ha. This suggests that β-carbon elimination from B-Me involves a binuclear (or polynuclear) Rh complex, although further investigations are required to elucidate the details of the mechanism of β-carbon elimination in the catalytic reaction process.

**Scheme 4:**
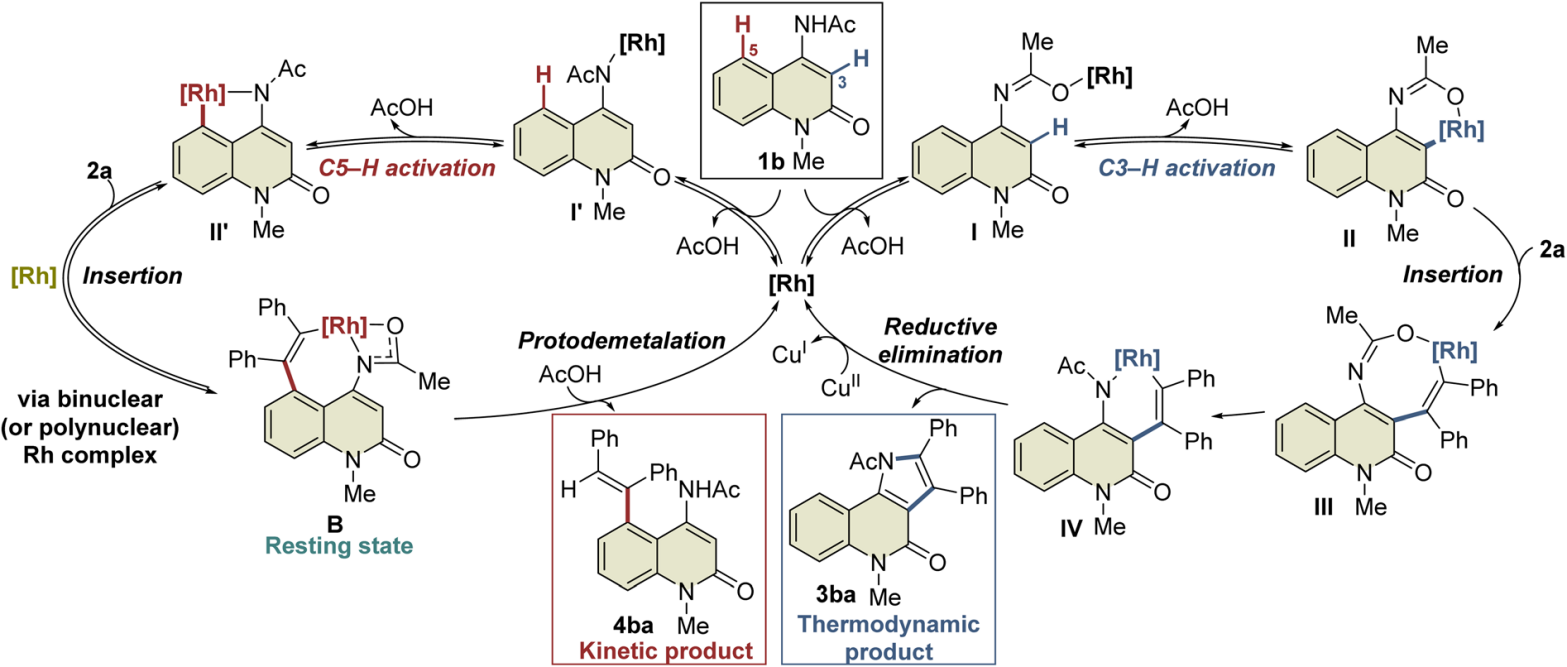
Proposed reaction mechanism.

A plausible mechanism based on these results is illustrated in Scheme 4. Initially, complex I′ is formed by the coordination of the Rh catalyst to the amide nitrogen of 1b, followed by the formation of five-membered rhodacycle II′*via* C5–H activation. Subsequent alkyne insertion provides seven-membered rhodacycle B, which is a reversible process that involves another Rh catalyst. On the other hand, C3–H activation proceeds *via* the coordination of the Rh catalyst to the amide oxygen to form the six-membered rhodacycle II. All these processes are reversible, and complex B is in a resting state. A catalytic amount of acetic acid generated *in situ* can protonate complex B (or the binuclear Rh complex) to afford 4ba under kinetic control, while alkyne insertion and reductive elimination from complex II proceed to afford the thermodynamic product 3ba. In the above-mentioned NMR experiment, complex B was formed much faster than the 3ba-Rh(i) complex (complex A) in the initial period of the reaction (Fig. 2a), and no NMR signals corresponding to intermediates III or IV were detected during the experiment. These results indicate that alkyne insertion from intermediate II may be a relatively slow process while reductive elimination from intermediate IV may occur more easily. Moreover, because of the low concentration of acetic acid in the catalytic process, protodemetalation from complex B is probably much slower than alkyne insertion and reductive elimination from intermediate II, allowing the smooth progress of C3-selective functionalization at the elevated temperature.

## Conclusions

In summary, we established an efficient method for preparing 3,4-fused 2-quinolones *via* the Rh(iii)-catalyzed selective alkenyl C–H functionalization of 4-amino-2-quinolones bearing an *N*-acetyl group as a directing group. This protocol has a wide substrate scope, including unsymmetrical internal alkynes, with complete regioselectivity. Several control experiments using an isolated key intermediate analog suggested that the reaction process involves an unprecedented reversible alkyne insertion process, leading to the simultaneous existence of key intermediates for both C3- and C5-functionalization in equilibrium. Alkyne insertion and reductive elimination from the C3-metalated intermediate may occur more readily than protodemetalation (or reductive elimination) from the seven-membered rhodacycle intermediate leading to C5-functionalization, allowing selective functionalization of the alkenyl C3–H bond over the aromatic C5–H bond. Further studies, including theoretical calculations, to elucidate the details of the reaction process are underway in our laboratory.

## Data availability

Data for the crystal structure reported in this paper have been deposited at the Cambridge Crystallographic Data Centre (CCDC) under the deposition number 2278040 (for 3ba), 2278041 (for 3bf′), 2278042 (for 4ba), 2278043 (for 5ha), 2278044 (for A), and 2278045 (for B-Me). All other data including experimental procedures, compound characterization, NMR spectra, theoretical calculations, and supporting figures and tables are recorded in the ESI.†

## Author contributions

N. H. performed experiments, computational studies, and X-ray crystal structure analysis. N. H. and T. Y. wrote the manuscript. T. Y. and Y. Y designed, advised, and directed the project. All authors edited the manuscript.

## Conflicts of interest

There are no conflicts to declare.

## Supplementary Material

SC-014-D3SC03987K-s001

SC-014-D3SC03987K-s002
