# Supplementary material for: Rh(iii)-catalyzed highly site- and regio-selective alkenyl C–H activation/annulation of 4-amino-2-quinolones with alkynes via reversible alkyne insertion
Source: Chem Sci. 2023 Sep 14;14(39):10971–8. doi: 10.1039/d3sc03987k (PMC10566469; doi:10.1039/d3sc03987k)

Electronic Supplementary Material (ESI) for Chemical Science.
This journal is © The Royal Society of Chemistry 2023

Supporting Information

Rh(III)-Catalyzed Vinylic C–H Activation/Annulation of 4-Amino-2-quinolones with Alkynes: Thermodynamically Controlled Site-Selectivity via Reversible Alkyne Insertion

Naohiro Hirako, Takeshi Yasui,* and Yoshihiko Yamamoto*

Department of Basic Medicinal Sciences, Graduate School of Pharmaceutical Sciences, Nagoya University, Furo-cho Chikusa, Nagoya 464-8603, Japan

t-yasui@ps.nagoya-u.ac.jp, yamamoto-yoshi@ps.nagoya-u.ac.jp

Table of Contents

1. Optimization of Reaction Conditions S2
2. General Information S4
3. Representative Procedure for the Rhodium-Catalyzed C–H Functionalization of **1** S4
4. Characterization of 3,4-Fused 2-Quinolone Derivatives **3** S4
5. Isolation and Characterization of C5-Functionalized 2-Quinolone Derivatives **4** and **5** S16
6. Synthesis and Characterization of 2-Quinolone substrates **1** S18
7. Mechanistic Studies S29
8. X-Ray Diffraction Analysis S42
9. DFT Calculations S48
10. Reference S59
11. NMR Spectra S62

1. Optimization of Reaction Conditions

Table S1. Optimization of reaction conditions[a]

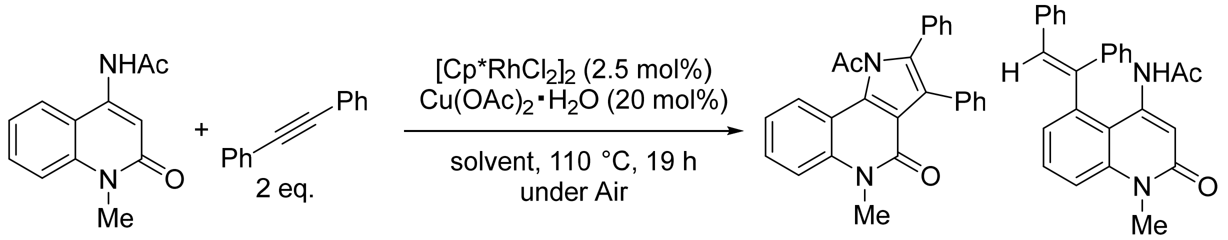

1b **2a** **3ba** **4ba**

entry	additive	solvent	**3ba** (%)	**4ba** (%)	**1b** (%)	memo
1[b]	$AgSbF_6$ (10 mol%)	DCE	43	n.d.	n.d.	**5ba** was obtained in 48% yield.
2[b]	-	DCE	63	n.d.	n.d.	
3	-	DCE	10	7	59	AgOAc(2 eq.) was used instead of $Cu(OAc)_2{\cdot}H_2O$
4	-	DCE	62	n.d.	n.d.	A trace amount of **5ba** was obtained.
5	-	DCE	n.d.	n.d.	91	Run at 100 ºC
6	-	*t*-AmOH	20	n.d.	67	
7	-	DMF	n.d.	n.d.	27	**3ba'** was obtained in 54% yield.
8	-	DMA	n.d.	n.d.	21	**3ba'** was obtained in 54% yield.
9	-	MeCN	n.d.	n.d.	-	complex mixture
10	-	toluene	n.d.	n.d.	87	
11	-	$PhCF_3$	68	n.d.	n.d.	
12	AgOAc (10 mol%)	$PhCF_3$	25	35	n.d.	
13	NaOAc (2 eq.)	$PhCF_3$	18	n.d.	40	

[a]Reaction conditions: **1b** (0.2 mmol), **2a** (0.4 mmol), $Cu(OAc)_2{\cdot}H_2O$ (20 mol%), $[Cp^*RhCl_2]_2$ (2.5 mol%), solvent (2 mL). Isolated yields are reported. [b]$Cu(OAc)_2{\cdot}H_2O$ (2 eq.) were used under an Ar atmosphere. n.d. = not detected.

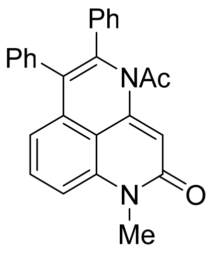

5ba

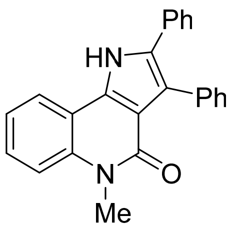

3ba'

Table S2. Effect of rhodium catalysts and temperature[a]

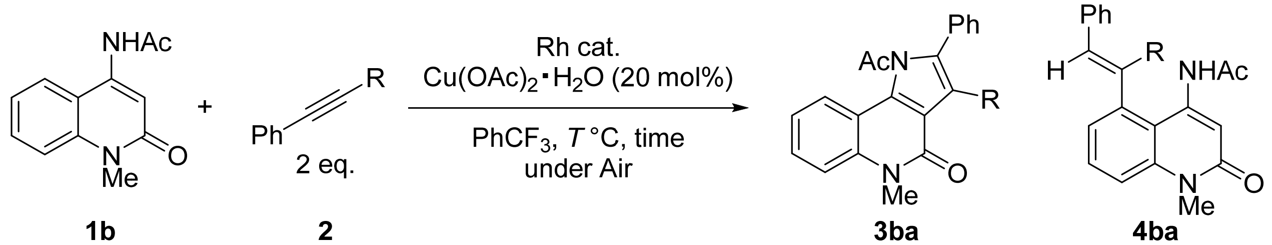

entry	R	Rh cat. (mol%)	additive (eq.)	*T*	time	**3ba** (%)[b]	**4ba** (%)[b]
1	Ph	$[Cp^*RhCl_2]_2$ (2.5)	-	110	19 h	(68)	n.d.
2	Me	$[Cp^*RhCl_2]_2$ (2.5)	-	110	12 h	87(85)	n.d.
3	Ph	$Cp^*Rh(OAc)_2 \cdot H_2O$ (5)	-	110	19 h	61(65)	6(5)
4	Me	$Cp^*Rh(OAc)_2 \cdot H_2O$ (5)	-	110	12 h	75(75)	8(8)
5[c]	Ph	$[Cp^*RhCl_2]_2$ (2.5)	-	100	19 h	n.d.	n.d.
6[c]	Ph	$Cp^*Rh(OAc)_2 \cdot H_2O$ (5)	-	100	19 h	23	10
7[c]	Ph	$Cp^*Rh(OAc)_2 \cdot H_2O$ (5)	-	70	6 days	29(27)	23(24)
8[c]	Me	$Cp^*Rh(OAc)_2 \cdot H_2O$ (5)	-	40	7 days	13	8
9	Me	$[Cp^*RhCl_2]_2$ (2.5)	AcOH (1)	110	19 h	n.d.	n.d.
10	Me	$[Cp^*RhCl_2]_2$ (2.5)	HFIP (1)	110	19 h	24	n.d.
11	Me	$Cp^*Rh(OAc)_2 \cdot H_2O$ (5)	TBACl (0.2)	70	24 h	n.d.	n.d.
12	Ph	$[CpRhI_2]_2$ (5)	-	110	19 h	n.d.	n.d.
13	Ph	$[Cp^ERhCl_2]_2$ (5)	-	110	19 h	14	n.d.

[a]Reaction conditions: **1b** (0.2 mmol), **2** (0.4 mmol), $Cu(OAc)_2 \cdot H_2O$ (20 mol%), Rh cat. (2.5 or 5 mol%), $PhCF_3$ (2 mL). [b]Yields were determined by ^1H NMR analysis. Isolated yields are shown in parentheses. [c]DCE was used as a solvent. n.d. = not detected.

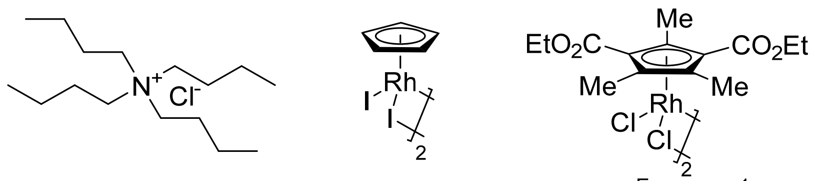

TBACl $[CpRhI_2]_2$ $[Cp^ERhCl_2]_2$[1]

2. General Information

General Considerations: All air- and moisture-sensitive reactions were performed under an argon (Ar) atmosphere. Analytical thin layer chromatography was performed using 0.25 mm silica gel plate (Merck TLC Silica gel 60 F_{254}). Column chromatography was performed on silica gel (Cica silica gel 60N) with solvents specified below. Melting points were recorded on SRS OptiMelt MPA100. NMR spectra were recorded on JEOL ESC-400 spectrometer (^{1}H/400 MHz,^{13}C/100 MHz and ^{19}F/376 MHz,) for samples in $CDCl_3$ and DMSO-d_6, CD_3OD solutions at 25 ˚C. ^{1}H NMR chemical shifts are reported in terms of chemical shift (δ, ppm) relative to the singlet at ($CDCl_3$ and CD_3OD δ 0.00 ppm for tetramethylsilane, DMSO-d_6 δ 2.49 ppm for the DMSO). ^{13}C NMR spectra were fully decoupled and are reported in terms of chemical shift (δ, ppm) relative to the solvent peak ($CDCl_3$, δ 77.00 ppm, DMSO, δ 39.52 ppm). ^{19}F NMR spectra are reported in terms of chemical shift (δ, ppm) relative to the singlet at δ −63.7 ppm for *α,α,α*-trifluorotoluene as an external standard. Splitting patterns are designated as follows: s, singlet; d, doublet; t, triplet; q, quartet; quint, quintet; sext, sextet; sept, septet; m, multiplet. Coupling constants are reported in Hz. Infrared spectra were recorded on JASCO FT/IR-230 spectrometer. High-resolution mass spectra were recorded on JEOL JMS-T100LP mass spectrometer.

Reagents and Solvents: Alkynes **2b-f**[2], **2h-j**[3], **2k**[4], **2l**[4], **2m**[5], **2n**[4], **2o**[3], **2p**[4], **2r**[6], **2s**[7], **2t**[8], $Cp^*Rh(OAc)_2·H_2O$[9] and $[Cp^*RhCl_2]_2$[10] were prepared according to the literature. Unless otherwise noted below, commercial reagents and solvents were purchased from Aldrich, Kanto Chemical, TCI, and Wako and used as received.

3. Representative Procedure for the Rhodium-Catalyzed C–H Functionalization of 1

A Schlenk tube was charged with $[Cp^*RhCl_2]_2$ (3.1 mg, 0.0050 mmol, 2.5 mol%), $Cu(OAc)_2·H_2O$ (7.9 mg, 0.040 mmol, 20 mol%), **1b** (43.2 mg, 0.20 mmol), **2a** (71.4 mg, 0.40 mmol) and $PhCF_3$ (2.0 mL). The reaction mixture was stirred at 110 °C for 19 h under air atmosphere using an oil bath. Then, the mixture was cooled to room temperature and filtered through a pad of Celite® with $CHCl_3$ to remove the metal salts. After concentration in vacuo, the residue was purified by silica gel column chromatography (Hexane/EtOAc = 3:1) to give **3ba**.

4. Characterization of 3,4-Fused 2-Quinolone Derivatives 3

1-Acetyl-5-methyl-2,3-diphenyl-1,5-dihydro-4*H*-pyrrolo[3,2-*c*]quinolin-4-one (3ba)

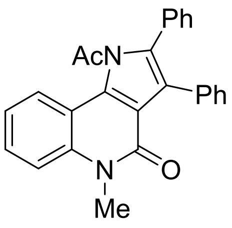

3ba

Analytical data for 3ba: 68% yield; white solid. Colorless crystals (mp 230.5–230.9 °C) for X-ray crystallographic analysis were obtained by recrystallization from DCM and hexane; ^{1}H NMR (400 MHz, $CDCl_3$) δ 7.81 (dd, J = 8.5, 1.4 Hz, 1H), 7.55-7.43 (m, 2H), 7.36-7.20 (m, 11H), 3.76 (s, 3H), 2.29 (s, 3H); ^{13}C NMR (100 MHz, $CDCl_3$) δ 176.4, 159.2, 138.3, 134.2, 132.8, 132.7, 131.0 (2C), 130.7 (2C), 128.59, 128.55 (2C), 128.3, 127.3 (2C), 126.8, 124.0, 122.3, 121.7, 115.5, 115.3, 113.5, 29.6, 29.3, One Csp^2 signal is missing due to overlapping; IR (neat) 3002, 1749, 1654 cm^{-1}; HRMS (DART) m/z: $[M+H]^+$ calcd for $C_{26}H_{21}N_2O_2$ 393.1603, found 393.1624.

1-Acetyl-2,3-bis(4-chlorophenyl)-5-methyl-1,5-dihydro-4*H*-pyrrolo[3,2-*c*]quinolin-4-one (3bb)

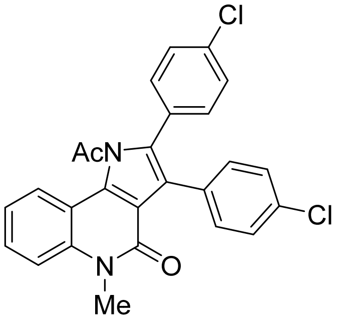

3bb

3bb was prepared following the representative procedure and purified by flash column chromatography on silica gel (Hexane/EtOAc = 4:1 to 3:1).

Analytical data for 3bb: 60% yield; yellow amorphous solid; ^{1}H NMR (400 MHz, $CDCl_3$) δ 7.76 (dd, J = 8.2, 1.4 Hz, 1H), 7.56-7.43 (m, 2H), 7.34-7.30 (m, 2H), 7.25-7.16 (m, 7H), 3.76 (s, 3H), 2.34 (s, 3H); ^{13}C NMR (100 MHz, $CDCl_3$) δ 175.9, 159.1, 138.4, 135.1, 133.1, 133.04, 133.00, 132.8, 132.3 (2C), 132.0 (2C), 131.0, 129.0 (2C), 128.6, 127.8 (2C), 123.1, 122.3, 121.9, 115.6, 115.0, 113.3, 29.7, 29.4; IR (KBr) 3061, 1751, 1655 cm^{-1}; HRMS (ESI) *m/z*: [M+Na]$^{+}$ calcd for $C_{26}H_{18}Cl_2N_2NaO_2$ 483.0643, found 483.0657.

1-Acetyl-2,3-bis(4-fluorophenyl)-5-methyl-1,5-dihydro-4*H*-pyrrolo[3,2-*c*]quinolin-4-one (3bc)

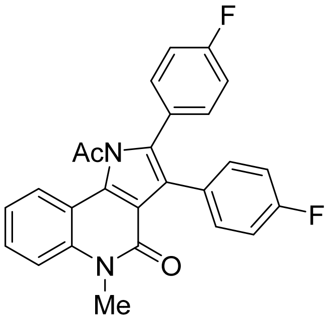

3bc

3bc was prepared following the representative procedure and purified by flash column chromatography on silica gel (Hexane/EtOAc = 4:1).

Analytical data for 3bc: 63% yield; yellow amorphous solid; ^{1}H NMR (400 MHz, $CDCl_3$) δ 7.76 (dd, J = 8.0, 1.1 Hz, 1H), 7.55-7.47 (m, 2H), 7.26-7.21 (m, 5H), 7.08-7.00 (m, 2H), 7.00-6.93 (m, 2H), 3.76 (s, 3H), 2.33 (s, 3H); ^{13}C NMR (100 MHz, $CDCl_3$) δ 176.0, 162.8 (d, $^{1}J_{CF}$ = 250.2 Hz), 162.0 (d, $^{1}J_{CF}$ = 246.3 Hz), 159.2, 138.3, 133.2, 132.8, 132.7 (d, $^{3}J_{CF}$ = 8.6 Hz, 2C), 132.6 (d, $^{3}J_{CF}$ = 8.6 Hz, 2C), 128.5 (d, $^{4}J_{CF}$ = 3.8 Hz), 126.4 (d, $^{4}J_{CF}$ = 3.8 Hz), 123.2, 122.3, 121.9, 115.9 (d, $^{2}J_{CF}$ = 22.0 Hz, 2C), 115.6, 115.1, 114.5 (d, $^{2}J_{CF}$ = 21.1 Hz, 2C), 113.4, 29.6, 29.4, One C*sp*^{2} signal is missing due to overlapping; ^{19}F NMR (376 MHz, $CDCl_3$): δ –112.4, –116.5; IR (KBr) 3005, 1760, 1656 cm^{-1}; HRMS (ESI) *m/z*: [M+Na]$^{+}$ calcd for $C_{26}H_{18}F_2N_2NaO_2$ 451.1234, found 451.1216.

1-Acetyl-5-methyl-2,3-bis(4-(trifluoromethyl)phenyl)-1,5-dihydro-4*H*-pyrrolo[3,2-*c*]quinolin-4-one (3bf)

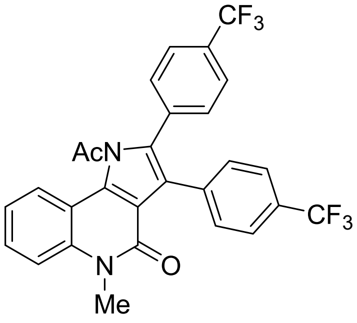

3bf

3bf was prepared following the representative procedure and purified by flash column chromatography on silica gel (Hexane/EtOAc = 4:1).

Analytical data for 3bf: 32% yield; yellow amorphous solid; ^{1}H NMR (400 MHz, $CDCl_3$) δ 7.77 (dd, J = 8.0, 1.1 Hz, 1H), 7.61 (d, J = 8.2 Hz, 2H), 7.57-7.49 (m, 4H), 7.42-7.36 (m, 4H), 7.31-7.27 (m, 1H), 3.77 (s, 3H), 2.35 (s, 3H); ^{13}C NMR (100 MHz, $CDCl_3$) δ 175.6, 159.0, 138.5, 136.2, 133.7, 133.5, 132.8, 131.3, 131.1, 130.9 (q, $^{2}J_{CF}$ = 32.6 Hz, 2C), 129.2 (q, $^{2}J_{CF}$ = 32.6 Hz, 2C), 129.0, 125.7 (q, $^{3}J_{CF}$ = 3.8 Hz, 2C), 124.5 (q, $^{3}J_{CF}$ = 3.8 Hz, 2C), 124.2 (q, $^{1}J_{CF}$ = 272.2 Hz), 123.7 (q, $^{1}J_{CF}$ = 271.3 Hz), 123.5, 122.3, 122.1, 115.7, 114.9, 113.1, 29.7, 29.4; ^{19}F NMR (376 MHz, $CDCl_3$): δ –63.4, –63.7; IR (neat) 1654, 1612 cm^{-1}; HRMS (DART) *m/z*: [M+H]$^{+}$ calcd for $C_{28}H_{19}F_6N_2O_2$ 529.1351, found 529.1368.

(*E*)-1-Acetyl-9-(1,2-bis(4-(trifluoromethyl)phenyl)vinyl)-5-methyl-2,3-bis(4-(trifluoromethyl)phenyl)-1,5-dihydro-4*H*-pyrrolo[3,2-*c*]quinolin-4-one (3bf')

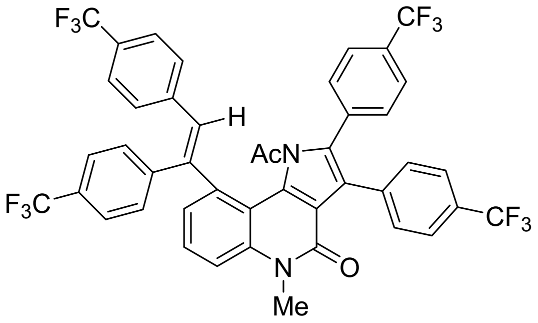

3bf'

3bf' was prepared following the representative procedure and purified by flash column chromatography on silica gel (Hexane/EtOAc = 1:1).

Analytical data for 3bf': 21% yield; white solid. Colorless crystals (mp 171.2–172.0 °C) for X-ray crystallographic analysis were obtained by recrystallization from $CHCl_3$ and hexane; ^{1}H NMR (400 MHz, $CDCl_3$) δ 7.63-7.48 (m, 5H), 7.48-7.39 (m, 6H), 7.25-7.15 (m, 6H), 7.11 (s, 1H), 6.99 (d, J = 7.8 Hz, 2H), 3.79 (s, 3H), 2.14 (s, 3H); ^{13}C NMR (100 MHz, $CDCl_3$) δ 170.4, 158.6, 142.1, 141.4, 140.1, 140.0, 139.6, 136.2, 135.6, 135.5, 133.9, 132.9, 131.6, 130.9, 130.8 (q, $^2J_{CF}$ = 32.6 Hz, 2C), 130.6, 130.3 (q, $^2J_{CF}$ = 32.6 Hz, 2C), 129.8 (q, $^2J_{CF}$ = 32.6 Hz, 2C), 129.4, 129.2 (q, $^2J_{CF}$ = 32.6 Hz, 2C), 128.7, 126.4, 125.6 (q, $^3J_{CF}$ = 3.8 Hz, 2C), 125.0, 124.9 (q, $^3J_{CF}$ = 3.8 Hz, 2C), 124.4 (q, $^3J_{CF}$ = 3.8 Hz, 2C), 124.3, 124.1 (q, $^1J_{CF}$ = 272.2 Hz), 123.9 (q, $^1J_{CF}$ = 272.2 Hz), 123.8 (q, $^1J_{CF}$ = 272.2 Hz), 123.6 (q, $^1J_{CF}$ = 277.0 Hz), 118.8, 115.6, 112.8, 29.9, 28.4, One C*sp*^{2} signal is missing due to overlapping; ^{19}F NMR (376 MHz, $CDCl_3$): δ –63.5 (3F), –63.6 (6F), –63.8 (3F); IR (neat) 1654 cm^{-1}; HRMS (ESI) *m/z*: [M+Na]$^+$ calcd for $C_{44}H_{26}F_{12}N_2NaO_2$ 865.1700, found 865.1687.

1-Acetyl-3,5-dimethyl-2-phenyl-1,5-dihydro-4*H*-pyrrolo[3,2-*c*]quinolin-4-one (3bg)

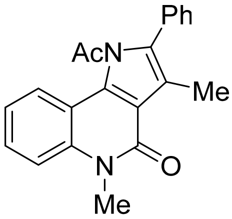

3bg

3bg was prepared following the representative procedure and purified by flash column chromatography on silica gel (Hexane/EtOAc = 4:1).

Analytical data for 3bg: 85% yield; yellow amorphous solid; ^{1}H NMR (400 MHz, $CDCl_3$) δ 7.83 (dd, J = 8.2, 0.9 Hz, 1H), 7.53-7.36 (m, 7H), 7.20 (ddd, J = 8.7, 5.3, 2.5 Hz, 1H), 3.80 (s, 3H), 2.45 (s, 3H), 2.20 (s, 3H); ^{13}C NMR (100 MHz, $CDCl_3$) δ 176.1, 160.4, 138.3, 133.7, 132.9, 131.3, 130.3 (2C), 128.8 (2C), 128.6, 128.1, 122.6, 121.6, 119.5, 116.6, 115.4, 114.0, 29.3, 29.2, 10.7; IR (neat) 1734, 1648 cm^{-1}; HRMS (DART) *m/z*: [M+H]$^+$ calcd for $C_{21}H_{19}N_2O_2$ 331.1447, found 331.1429.

The stereochemistry of **3bg** was assigned by NOESY experiments. The arrows shown below indicate the observed cross peaks.

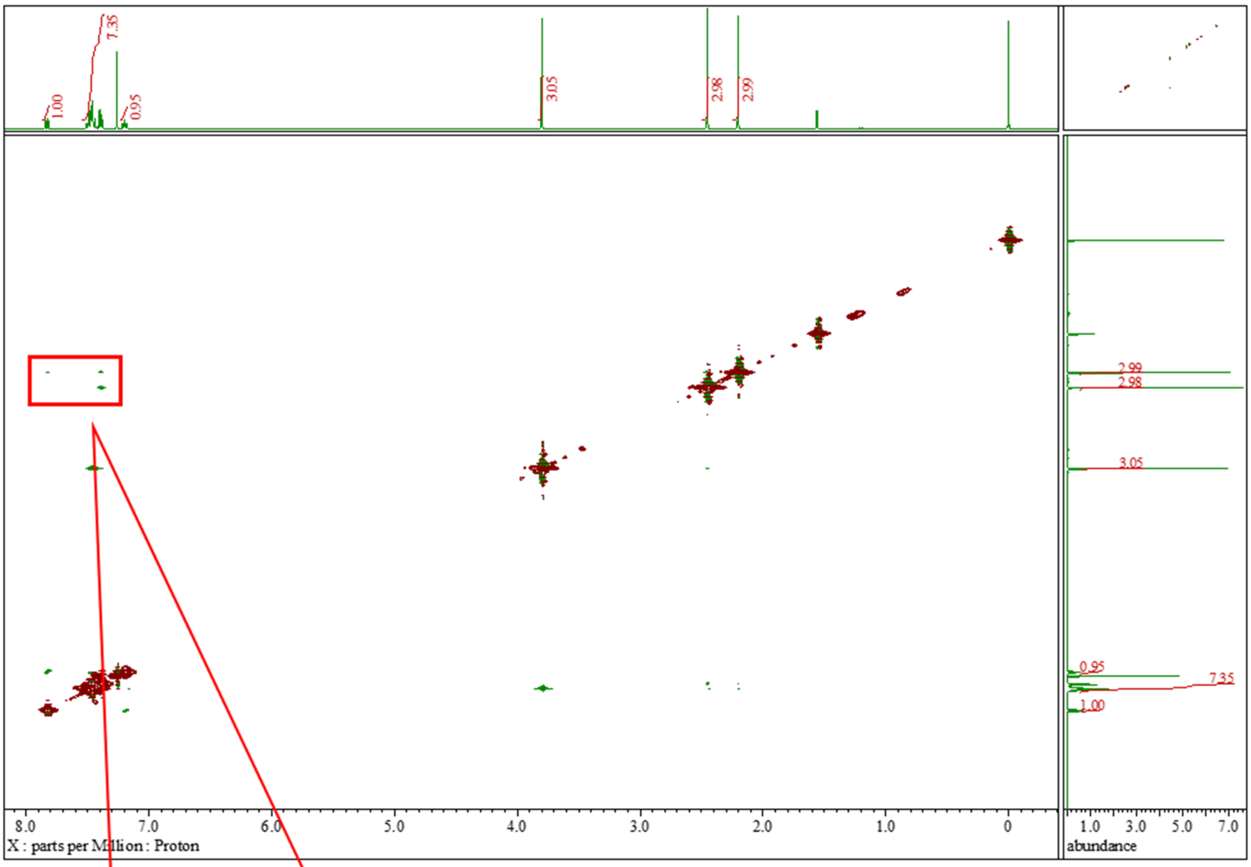

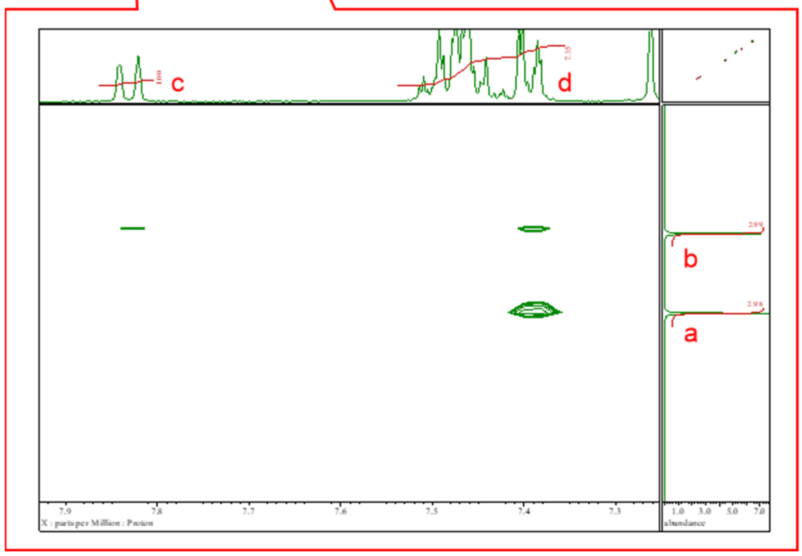

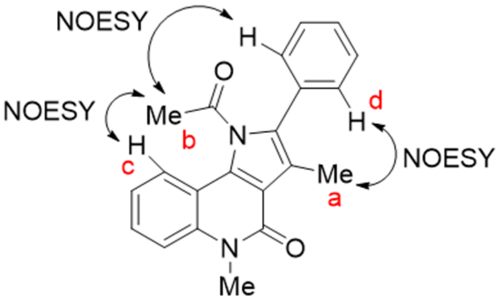

2,5-Dimethyl-3-phenyl-1-pivaloyl-1,5-dihydro-4*H*-pyrrolo[3,2-*c*]quinolin-4-one (3b'g)

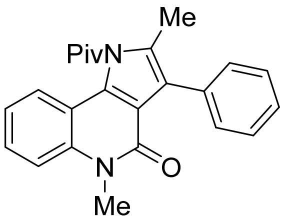

3b'g

3b'g was prepared following the representative procedure and purified by flash column chromatography on silica gel (Hexane/EtOAc = 4:1).

Analytical data for 3b'g: 95% yield; white amorphous solid; ^{1}H NMR (400 MHz, $CDCl_3$) δ 7.71 (d, *J* = 8.2 Hz, 1H), 7.51-7.43 (m, 6H), 7.41-7.35 (m, 1H), 7.18 (ddd, *J* = 8.0, 5.0, 3.0 Hz, 1H), 3.80 (s, 3H), 2.58 (s, 3H), 0.82 (s, 9H); ^{13}C NMR (100 MHz, $CDCl_3$) δ 189.4, 160.6, 138.0, 133.5, 132.0, 131.0, 130.7, 128.6, 128.2, 127.9, 121.6, 121.3, 118.0, 115.4, 115.0, 114.2, 45.9, 29.0, 27.4, 10.9; IR (KBr) 1736, 1649 cm^{-1}; HRMS (ESI) *m/z*: $[M+Na]^+$ calcd for $C_{24}H_{24}N_2NaO_2$ 395.1736, found 395.1739. The stereochemistry of **3b'g** was assigned by NOE experiments. The arrows shown below indicate the observed cross peaks.

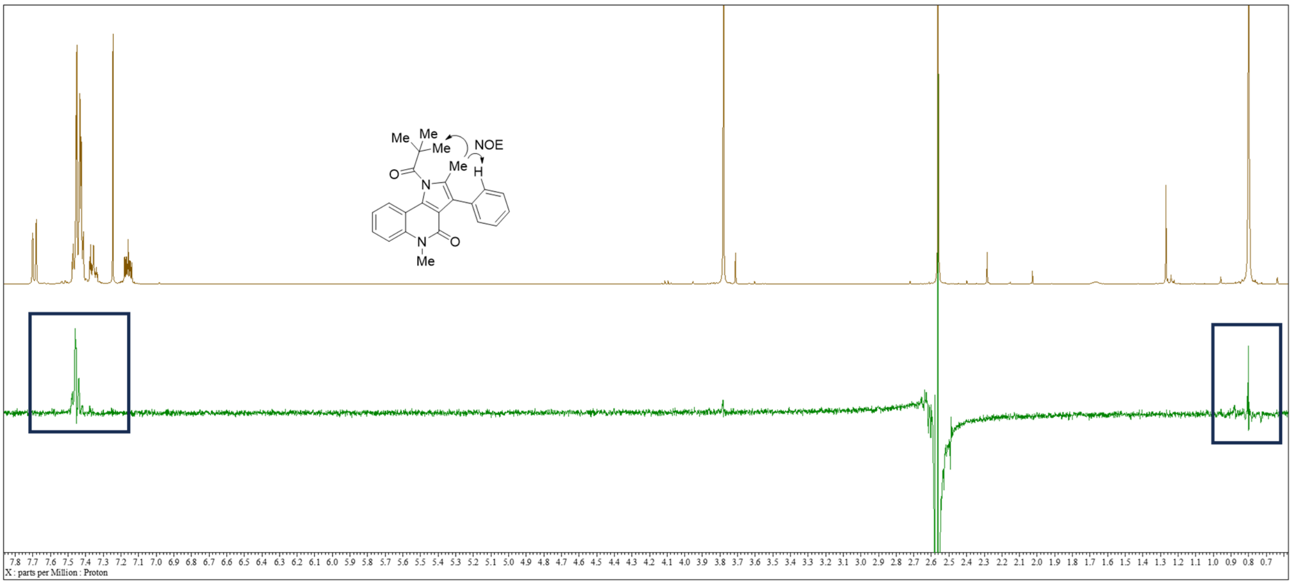

1-Acetyl-3,5-dimethyl-2-(*p*-tolyl)-1,5-dihydro-4*H*-pyrrolo[3,2-*c*]quinolin-4-one (3bh)

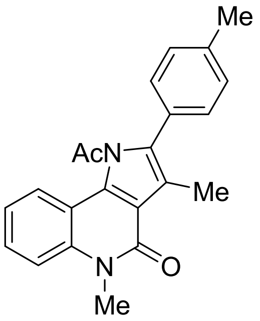

3bh

3bh was prepared following the representative procedure and purified by flash column chromatography on silica gel (Hexane/EtOAc = 4:1).

Analytical data for 3bh: 79% yield; white solid (mp 206.7–208.8 °C); ^{1}H NMR (400 MHz, $CDCl_3$) δ 7.82 (d, *J* = 7.8 Hz, 1H), 7.50-7.42 (m, 2H), 7.32-7.27 (m, 4H), 7.19 (ddd, *J* = 8.1, 5.6, 2.8 Hz, 1H), 3.80 (s, 3H), 2.44 (s, 3H), 2.43 (s, 3H), 2.21 (s, 3H); ^{13}C NMR (100 MHz, $CDCl_3$) δ 176.2, 160.4, 138.6, 138.3, 133.8, 132.8, 130.2 (2C), 129.5 (2C), 128.3, 128.0, 122.6, 121.6, 119.2, 116.7, 115.3, 114.0, 29.4, 29.2, 21.4, 10.7; IR (KBr) 1743, 1647 cm^{-1}; HRMS (ESI) *m/z*: $[M+Na]^+$ calcd for $C_{22}H_{20}N_2NaO_2$ 367.1422, found 367.1411.

1-Acetyl-2-(4-methoxyphenyl)-3,5-dimethyl-1,5-dihydro-4*H*-pyrrolo[3,2-*c*]quinolin-4-one (3bi)

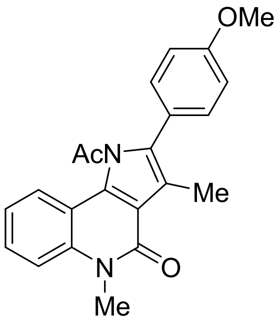
3bi

3bi was prepared following the representative procedure and purified by flash column chromatography on silica gel (Hexane/EtOAc = 2:1).

Analytical data for 3bi: 71% yield; white solid (mp 170.3–173.2 °C); ^{1}H NMR (400 MHz, $CDCl_3$) δ 7.81 (d, *J* = 8.2 Hz, 1H), 7.48-7.44 (m, 2H), 7.34-7.29 (m, 2H), 7.19 (ddd, *J* = 8.4, 5.4, 2.8 Hz, 1H), 7.04-6.99 (m, 2H), 3.88 (s, 3H), 3.80 (s, 3H), 2.43 (s, 3H), 2.22 (s, 3H); ^{13}C NMR (100 MHz, $CDCl_3$) δ 176.2, 160.4, 159.8, 138.2, 133.6, 132.6, 131.6 (2C), 127.9, 123.3, 122.5, 121.6, 119.0, 116.6, 115.3, 114.2 (2C), 114.0, 55.3, 29.3, 29.2, 10.7; IR (neat) 3003, 1739, 1648, 1250 cm^{-1}; HRMS (DART) *m/z*: $[M+H]^+$ calcd for $C_{22}H_{21}N_2O_3$ 361.1552, found 361.1543.

1-Acetyl-3,5-dimethyl-2-(4-(trifluoromethyl)phenyl)-1,5-dihydro-4*H*-pyrrolo[3,2-*c*]quinolin-4-one (3bj)

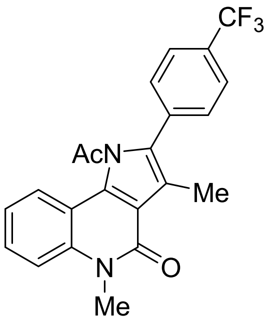
3bj

3bj was prepared following the representative procedure and purified by flash column chromatography on silica gel (Hexane/EtOAc = 4:1). The obtained solid was reprecipitated from $CHCl_3$ and Et_2O for melting point determination.

Analytical data for 3bj: 85% yield; white solid (mp 190.5–191.0 °C); ^{1}H NMR (400 MHz, $CDCl_3$) δ 7.80 (d, *J* = 7.8 Hz, 1H), 7.75 (d, *J* = 8.2 Hz, 2H), 7.58-7.44 (m, 4H), 7.22 (ddd, *J* = 8.2, 6.6, 1.6 Hz, 1H), 3.80 (s, 3H), 2.47 (s, 3H), 2.26 (s, 3H); ^{13}C NMR (100 MHz, $CDCl_3$) δ 175.6, 160.2, 138.5, 134.9, 133.5, 132.1, 130.7, 130.5 (q, $^{2}J_{CF}$ = 32.6 Hz, 2C), 128.5, 125.7 (q, $^{3}J_{CF}$ = 2.9 Hz, 2C), 123.9 (q, $^{1}J_{CF}$ = 272.2 Hz), 122.6, 121.8, 120.6, 116.6, 115.5, 113.7, 29.5, 29.2, 10.7; ^{19}F NMR (376 MHz, $CDCl_3$) δ –63.6; IR (neat) 1739, 1653, 1325 cm^{-1}; HRMS (DART) *m/z*: $[M+H]^+$ calcd for $C_{22}H_{18}F_3N_2O_2$ 399.1320, found 399.1309.

1-Acetyl-2-(4-bromophenyl)-3,5-dimethyl-1,5-dihydro-4*H*-pyrrolo[3,2-*c*]quinolin-4-one (3bk)

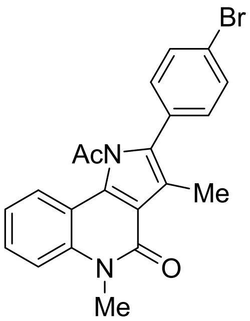
3bk

3bk was prepared following the representative procedure and purified by flash column chromatography on silica gel (Hexane/EtOAc = 3:1).

Analytical data for 3bk: 83% yield; yellow amorphous solid; ^{1}H NMR (400 MHz, $CDCl_3$) δ 7.79 (dd, *J* = 8.2, 0.9 Hz, 1H), 7.65-7.60 (m, 2H), 7.51-7.43 (m, 2H), 7.28-7.25 (m, 2H), 7.21 (ddd, *J* = 8.2, 6.4, 1.8 Hz, 1H), 3.80 (s, 3H), 2.44 (s, 3H), 2.25 (s, 3H); ^{13}C NMR (100 MHz, $CDCl_3$) δ 175.7, 160.3, 138.4, 133.2, 132.4, 132.0 (2C), 131.9 (2C), 130.1, 128.3, 123.0, 122.6, 121.7, 119.9, 116.6, 115.4, 113.8, 29.4, 29.2, 10.7; IR (KBr) 1744, 1646 cm^{-1}; HRMS (ESI) *m/z*: $[M+Na]^+$ calcd for $C_{21}H_{17}BrN_2NaO_2$ 431.0371, found 431.0398.

1-Acetyl-2-(4-fluorophenyl)-3,5-dimethyl-1,5-dihydro-4*H*-pyrrolo[3,2-*c*]quinolin-4-one (3bl)

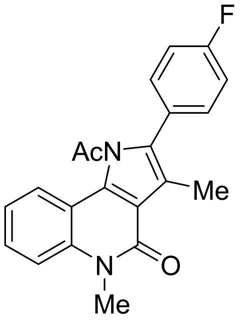

3bl

3bl was prepared following the representative procedure and purified by flash column chromatography on silica gel (Hexane/EtOAc = 3:1).

Analytical data for 3bl: 80% yield; yellow amorphous solid; ^{1}H NMR (400 MHz, $CDCl_3$) δ 7.78 (dd, *J* = 8.4, 1.4 Hz, 1H), 7.50-7.45 (m, 2H), 7.41-7.35 (m, 2H), 7.24-7.16 (m, 3H), 3.80 (s, 3H), 2.43 (s, 3H), 2.24 (s, 3H); ^{13}C NMR (100 MHz, $CDCl_3$) δ 175.8, 162.8 (d, $^1J_{CF}$ = 250.2 Hz), 160.3, 138.3, 133.0, 132.6, 132.3 (d, $^3J_{CF}$ = 8.6 Hz, 2C), 128.2, 127.2 (d, $^4J_{CF}$ = 2.9 Hz), 122.6, 121.7, 119.7, 116.6, 115.9 (d, $^2J_{CF}$ = 21.1 Hz, 2C), 115.4, 113.9, 29.3, 29.2, 10.7; ^{19}F NMR (376 MHz, $CDCl_3$) δ –112.9; IR (KBr) 2928, 1748, 1647 cm^{-1}; HRMS (ESI) *m/z*: [M+Na]$^+$ calcd for $C_{21}H_{17}FN_2NaO_2$ 371.1172, found 371.1142.

4-(1-Acetyl-3,5-dimethyl-4-oxo-4,5-dihydro-1*H*-pyrrolo[3,2-*c*]quinolin-2-yl)benzonitrile (3bm)

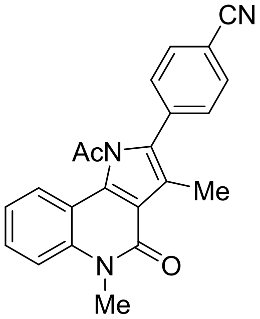

3bm

3bm was prepared following the representative procedure and purified by flash column chromatography on silica gel (Hexane/EtOAc = 2:1).

Analytical data for 3bm: 67% yield; white amorphous solid; ^{1}H NMR (400 MHz, $CDCl_3$) δ 7.79 (d, *J* = 8.2 Hz, 3H), 7.58-7.46 (m, 4H), 7.26-7.21 (m, 1H), 3.80 (s, 3H), 2.48 (s, 3H), 2.28 (s, 3H); ^{13}C NMR (100 MHz, $CDCl_3$) δ 175.4, 160.2, 138.6, 135.8, 133.9, 132.4 (2C), 131.7, 130.9 (2C), 128.7, 122.7, 121.9, 121.2, 118.4, 116.7, 115.6, 113.6, 112.2, 29.5, 29.3, 10.8; IR (KBr) 3066, 2226, 1739, 1651 cm^{-1}; HRMS (ESI) *m/z*: [M+Na]$^+$ calcd for $C_{22}H_{17}N_3NaO_2$ 378.1219, found 378.1228.

1-Acetyl-2-(3-methoxyphenyl)-3,5-dimethyl-1,5-dihydro-4*H*-pyrrolo[3,2-*c*]quinolin-4-one (3bn)

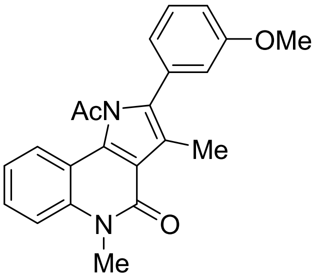

3bn

3bn was prepared following the representative procedure and purified by flash column chromatography on silica gel (Hexane/EtOAc = 4:1 to 3:1).

Analytical data for 3bn: 78% yield; yellow solid (mp 139.7–142.1 °C); ^{1}H NMR (400 MHz, $CDCl_3$) δ 7.84 (dd, *J* = 8.5, 1.4 Hz, 1H), 7.49-7.44 (m, 2H), 7.40 (m, 1H), 7.19 (ddd, *J* = 8.2, 6.0, 2.3 Hz, 1H), 7.00-6.92 (m, 3H), 3.86 (s, 3H), 3.80 (s, 3H), 2.47 (s, 3H), 2.23 (s, 3H); ^{13}C NMR (100 MHz, $CDCl_3$) δ 176.1, 160.3, 159.7, 138.3, 133.4, 132.8, 132.6, 129.9, 128.0, 122.6, 122.5, 121.6, 119.4, 116.5, 115.8, 115.3, 114.1, 113.9, 55.3, 29.3, 29.2, 10.7; IR (KBr) 3058, 1746, 1640 cm^{-1}; HRMS (ESI) *m/z*: [M+Na]$^+$ calcd for $C_{22}H_{20}N_2NaO_3$ 383.1372, found 383.1348.

1-Acetyl-2-(3-chlorophenyl)-3,5-dimethyl-1,5-dihydro-4*H*-pyrrolo[3,2-*c*]quinolin-4-one (3bo)

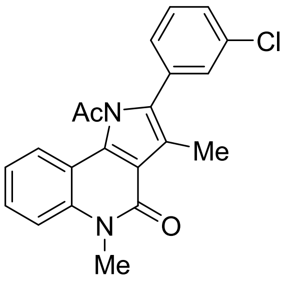

3bo

3bo was prepared following the representative procedure and purified by flash column chromatography on silica gel (Hexane/EtOAc = 4:1).

Analytical data for 3bo: 80% yield; yellow solid (mp 191.5–192.8 °C); ^{1}H NMR (400 MHz, $CDCl_3$) δ 7.80 (dd, *J* = 8.7, 0.9 Hz, 1H), 7.52-7.44 (m, 3H), 7.44-7.39 (m, 4H), 7.23-7.18 (m, 1H), 3.80 (s, 3H), 2.45 (s, 3H), 2.26 (s, 3H); ^{13}C NMR (100 MHz, $CDCl_3$) δ 175.5, 160.3, 138.4, 134.7, 133.3, 133.0, 132.1, 130.3, 130.0, 128.8, 128.6, 128.3, 122.7, 121.7, 120.2, 116.6, 115.4, 113.8, 29.4, 29.2, 10.7; IR (KBr) 3049, 1749, 1642 cm^{-1}; HRMS (ESI) *m/z*: $[M+Na]^+$ calcd for $C_{21}H_{17}ClN_2NaO_2$ 387.0876, found 387.0857.

1-Acetyl-3,5-dimethyl-2-(naphthalen-2-yl)-1,5-dihydro-4*H*-pyrrolo[3,2-*c*]quinolin-4-one (3bp)

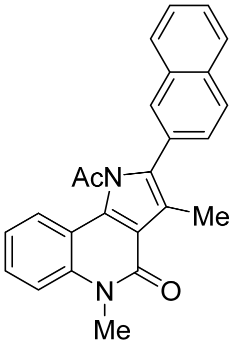

3bp

3bp was prepared following the representative procedure and purified by flash column chromatography on silica gel (Hexane/EtOAc = 3:1).

Analytical data for 3bp: 75% yield; white solid (mp 213.0–213.9 °C); ^{1}H NMR (400 MHz, $CDCl_3$) δ 7.96 (d, *J* = 8.7 Hz, 1H), 7.94-7.89 (m, 2H), 7.87 (d, *J* = 8.2 Hz, 2H), 7.61-7.54 (m, 2H), 7.53-7.45 (m, 3H), 7.21 (ddd, *J* = 8.8, 4.9, 2.8 Hz, 1H), 3.82 (s, 3H), 2.50 (s, 3H), 2.20 (s, 3H); ^{13}C NMR (100 MHz, $CDCl_3$) δ 176.1, 160.4, 138.4, 133.7, 133.12, 133.10, 132.9, 129.7, 128.7, 128.6, 128.2, 128.1, 127.8, 127.5, 126.9, 126.8, 122.7, 121.7, 120.0, 116.7, 115.4, 114.0, 29.4, 29.2, 10.8; IR (KBr) 2928, 1743, 1645cm^{-1}; HRMS (ESI) *m/z*: $[M+Na]^+$ calcd for $C_{25}H_{20}N_2NaO_2$ 403.1423, found 403.1397.

1-Acetyl-5-methyl-2-phenyl-3-propyl-1,5-dihydro-4*H*-pyrrolo[3,2-*c*]quinolin-4-one (3bq)

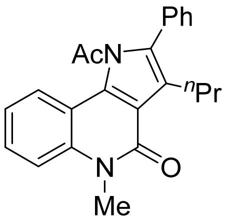

3bq

3bq was prepared following the representative procedure and purified by flash column chromatography on silica gel (Hexane/EtOAc = 3:1).

Analytical data for 3bq: 81% yield; yellow amorphous solid; ^{1}H NMR (400 MHz, $CDCl_3$) δ 7.78 (d, *J* = 7.8 Hz, 1H), 7.50-7.44 (m, 5H), 7.40-7.36 (m, 2H), 7.19 (ddd, *J* = 8.1, 5.6, 2.8 Hz, 1H), 3.80 (s, 3H), 2.78-2.74 (m, 2H), 2.20 (s, 3H), 1.69-1.64 (m, 2H), 0.89 (t, *J* = 7.3 Hz, 3H); ^{13}C NMR (100 MHz, $CDCl_3$) δ 176.0, 159.9, 138.3, 133.8, 133.2, 131.5, 130.5 (2C), 128.7 (2C), 128.4, 128.0, 124.4, 122.6, 121.5, 116.2, 115.3, 113.8, 29.30, 29.26, 26.8, 24.7, 14.2; IR (neat) 2959, 1741, 1649, 1251 cm^{-1}; HRMS (DART) *m/z*: $[M+H]^+$ calcd for $C_{23}H_{23}N_2O_2$ 359.1760, found 359.1730.

1-Acetyl-3-(((*tert*-butyldimethylsilyl)oxy)methyl)-5-methyl-2-phenyl-1,5-dihydro-4*H*-pyrrolo[3,2-*c*]quinolin-4-one (3br)

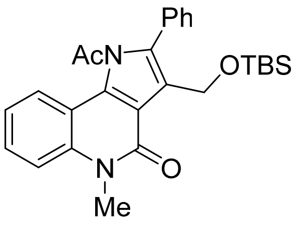

3br

3br was prepared following the representative procedure and purified by flash column chromatography on silica gel (Hexane/EtOAc = 10:1).

Analytical data for 3br: 80% yield; colorless gum; ^{1}H NMR (400 MHz, $CDCl_3$) δ 7.77 (dd, *J* = 8.2, 0.9 Hz, 1H), 7.62-7.53 (m, 2H), 7.51-7.41 (m, 5H), 7.18 (ddd, *J* = 8.2, 6.2, 2.1 Hz, 1H), 4.96 (s, 2H), 3.79 (s, 3H), 2.24 (s, 3H), 0.90 (s, 9H), 0.10 (s, 6H); ^{13}C NMR (100 MHz, $CDCl_3$) δ 176.4, 159.5, 138.3, 136.4, 132.7, 130.7, 130.4 (2C), 128.9, 128.5 (2C), 128.1, 122.2, 122.0, 121.6, 115.4, 115.3, 113.7, 55.5, 29.5, 29.4, 26.1 (3C), 18.6 , –5.2 (2C); IR (neat) 2928, 1748, 1652, 1254 cm^{-1}; HRMS (DART) *m/z*: [M+H]$^+$ calcd for $C_{27}H_{33}N_2O_3Si$ 461.2260, found 461.2283.

1-Acetyl-5-methyl-3-(phenoxymethyl)-2-phenyl-1,5-dihydro-4*H*-pyrrolo[3,2-*c*]quinolin-4-one (3bs)

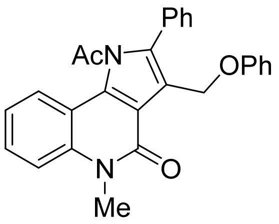

3bs

3bs was prepared following the representative procedure and purified by flash column chromatography on silica gel (Hexane/EtOAc = 4:1).

Analytical data for 3bs: 76% yield; yellow solid (mp 209.5–211.3 °C); ^{1}H NMR (400 MHz, $CDCl_3$) δ 7.81 (dd, *J* = 8.7, 0.9 Hz, 1H), 7.54-7.48 (m, 4H), 7.47-7.42 (m, 3H), 7.29-7.18 (m, 3H), 7.03-6.98 (m, 2H), 6.93 (ddd, *J* = 7.1, 7.1, 0.9 Hz, 1H), 5.31 (s, 2H), 3.80 (s, 3H), 2.27 (s, 3H); ^{13}C NMR (100 MHz, $CDCl_3$) δ 176.2, 159.6, 158.8, 138.4, 137.3, 133.0, 130.2 (2C), 130.0, 129.3 (2C), 129.2, 128.9 (2C), 128.4, 122.3, 121.8, 120.7, 117.8, 115.6, 115.5, 115.1 (2C), 113.6, 60.7, 29.5, 29.3; IR (neat) 1749, 1653 cm^{-1}; HRMS (DART) *m/z*: [M+H]$^+$ calcd for $C_{27}H_{23}N_2O_3$ 423.1709, found 423.1712.

***tert*-Butyl((1-acetyl-5-methyl-4-oxo-2-phenyl-4,5-dihydro-1*H*-pyrrolo[3,2-*c*]quinolin-3-yl)methyl)carbamate (3bt)**

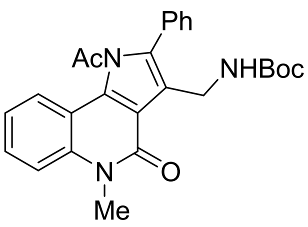

3bt

3bt was prepared following the representative procedure and purified by flash column chromatography on silica gel (Hexane/EtOAc = 4:1).

Analytical data for 3bt: 66% yield; white solid (mp 178.5–180.5 °C); ^{1}H NMR (400 MHz, $CDCl_3$) δ 7.82 (d, *J* = 7.8 Hz, 1H), 7.60-7.41 (m, 7H), 7.23 (ddd, *J* = 8.4, 5.8, 2.8 Hz, 1H), 6.60 (br s, 1H), 4.39 (d, *J* = 6.0 Hz, 2H), 3.84 (s, 3H), 2.20 (s, 3H), 1.43 (s, 9H); ^{13}C NMR (100 MHz, $CDCl_3$) δ 176.2, 160.2, 155.7, 138.2, 134.2, 133.4, 130.5 (2C), 130.0, 129.2, 129.0 (2C), 128.5, 122.5, 122.0, 120.1, 116.1, 115.6, 113.7, 78.6, 35.3, 29.6, 29.4, 28.5 (3C); IR (neat) 3392, 2978, 1748, 1707, 1636 cm^{-1}; HRMS (DART) *m/z*: [M+H]$^+$ calcd for $C_{26}H_{28}N_3O_4$ 446.2080, found 446.2108.

1-Acetyl-2-(3,5-dichlorophenyl)-5-methyl-3-(*p*-tolyl)-1,5-dihydro-4*H*-pyrrolo[3,2-*c*]quinolin-4-one with 1-acetyl-3-(3,5-dichlorophenyl)-5-methyl-2-(*p*-tolyl)-1,5-dihydro-4*H*-pyrrolo[3,2-*c*]quinolin-4-one (3bu)

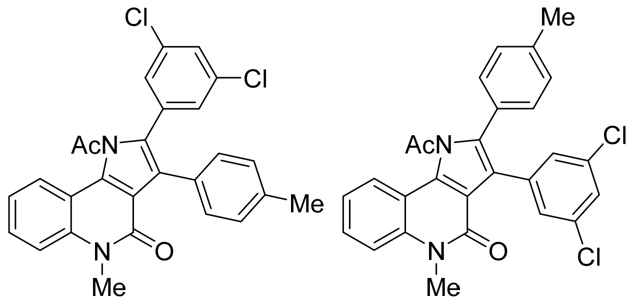

3bu

3bu was prepared following the representative procedure and purified by flash column chromatography on silica gel (Hexane/EtOAc = 3:1). Regioisomers (1:1) could be separated by column chromatography.

Analytical data for 3bu: regioisomer-1 20 % yield; yellow amorphous solid; ^{1}H NMR (400 MHz, $CDCl_3$) δ 7.77 (d, *J* = 8.2 Hz, 1H), 7.58-7.42 (m, 2H), 7.25-7.20 (m, 2H), 7.20-7.10 (m, 6H), 3.76 (s, 3H),2.38 (s, 3H), 2.29 (s, 3H); ^{13}C NMR (100 MHz, $CDCl_3$) δ 176.2, 159.0, 139.3, 138.3, 136.2, 134.9, 133.6, 132.6, 130.5 (C2), 129.6 (C4), 128.5, 127.0, 126.7, 122.3, 121.8, 120.9, 115.6, 114.9, 113.3, 29.6, 29.5, 21.4 One C*sp*^{2} signals are missing due to overlapping; IR (KBr) 1408, 1360 cm^{-1}; HRMS (ESI) *m/z*: [M+Na]$^+$ calcd for $C_{27}H_{20}Cl_2N_2NaO_2$ 497.0800, found 497.0812. **regioisomer-2** 20% yield; yellow amorphous solid; ^{1}H NMR (400 MHz, $CDCl_3$) δ 7.74 (d, *J* = 8.2 Hz, 1H), 7.57-7.45 (m, 2H), 7.33-7.25 (m, 2H), 7.20-7.05 (m, 6H), 3.74 (s, 3H),2.39 (s, 3H), 2.31 (s, 3H); ^{13}C NMR (100 MHz, $CDCl_3$) δ 175.5, 159.1, 138.6, 137.0, 135.1, 133.7, 133.5, 131.2, 130.6 (2C), 129.3 (2C), 128.9 (2C), 128.8, 128.5 (2C), 126.2, 125.6, 122.6, 122.0, 115.7, 115.5, 113.4, 29.8, 29.5, 21.4; IR (KBr) 1645, 1402, 1360; HRMS (ESI) *m/z*: [M+Na]$^+$ calcd for $C_{27}H_{20}Cl_2N_2NaO_2$ 497.0800, found 497.0783.

1-Acetyl-5,8-dimethyl-2,3-diphenyl-1,5-dihydro-4*H*-pyrrolo[3,2-*c*]quinolin-4-one (3ca)

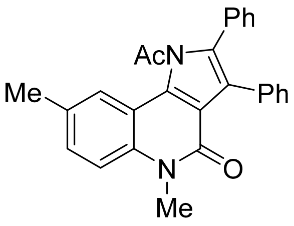

3ca

3ca was prepared following the representative procedure and purified by flash column chromatography on silica gel (Hexane/EtOAc = 3:1).

Analytical data for 3ca: 69% yield; yellow solid (mp 177.5–179.4 °C); ^{1}H NMR (400 MHz, $CDCl_3$) δ 7.57 (s, 1H), 7.40-7.17 (m, 12H), 3.73 (s, 3H), 2.44 (s, 3H), 2.29 (s, 3H); ^{13}C NMR (100 MHz, $CDCl_3$) δ 176.4, 159.1, 136.4, 134.1, 132.9, 132.6, 131.1, 131.0 (2C), 130.8 (2C), 129.5, 128.54, 128.52 (2C), 127.3 (2C), 126.8, 124.1, 122.3, 115.4, 113.5, 29.6, 29.3, 21.1, Two C*sp*^{2} signals are missing due to overlapping; IR (KBr) 2919, 1753, 1646 cm^{-1}; HRMS (ESI) *m/z*: [M+Na]$^+$ calcd for $C_{27}H_{22}N_2NaO_2$ 429.1579, found 429.1573.

1-Acetyl-5-methyl-2,3-diphenyl-8-(trifluoromethyl)-1,5-dihydro-4*H*-pyrrolo[3,2-*c*]quinolin-4-one (3da)

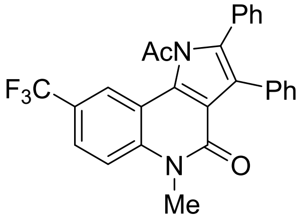

3da

3da was prepared following the representative procedure and purified by flash column chromatography on silica gel (Hexane/EtOAc = 4:1).

Analytical data for 3da: 74% yield; yellow solid (mp 210.3–211.4 °C); ^{1}H NMR (400 MHz, $CDCl_3$) δ 8.16 (d, *J* = 0.9 Hz, 1H), 7.72 (dd, *J* = 8.9, 1.6 Hz, 1H), 7.55 (d, *J* = 9.2 Hz, 1H), 7.37-7.30 (m, 3H), 7.28-7.23 (m, 7H), 3.77 (s, 3H), 2.27 (s, 3H); ^{13}C NMR (100 MHz, $CDCl_3$) δ 175.9, 159.1, 140.2, 135.1, 132.4, 131.9, 130.9 (2C), 130.6 (2C), 128.9, 128.7 (2C), 127.4 (2C), 127.0, 124.6, 124.4, 124.1 (q, $^{1}J_{CF}$ = 272.2 Hz), 123.7 (q, $^{2}J_{CF}$ = 32.6 Hz), 120.0, 119.9 (q, $^{3}J_{CF}$ = 3.8 Hz), 116.1, 115.7, 113.5, 29.6, 29.5; ^{19}F NMR (376 MHz, $CDCl_3$) δ –62.7; IR (KBr) 3059, 1750, 1659 cm^{-1}; HRMS (ESI) *m/z*: $[M+Na]^+$ calcd for $C_{27}H_{19}F_3N_2NaO_2$ 483.1296, found 483.1289.

1-Acetyl-8-fluoro-5-methyl-2,3-diphenyl-1,5-dihydro-4*H*-pyrrolo[3,2-*c*]quinolin-4-one (3ea)

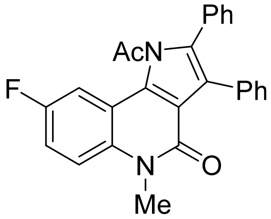

3ea

3ea was prepared following the representative procedure and purified by flash column chromatography on silica gel (Hexane/EtOAc = 3:1).

Analytical data for 3ea: 57% yield; white amorphous solid; ^{1}H NMR (400 MHz, $CDCl_3$) δ 7.56 (dd, *J* = 10.3, 6.0 Hz, 1H), 7.41 (dd, *J* = 9.2, 5.0 Hz, 1H), 7.36-7.30 (m, 3H), 7.25-7.19 (m, 8H), 3.74 (s, 3H), 2.25 (s, 3H); ^{13}C NMR (100 MHz, $CDCl_3$) δ 176.1, 158.8, 157.4 (d, $^{1}J_{CF}$ = 240.6 Hz), 134.9, 134.7, 132.6, 131.8, 130.9 (2C), 130.6, 130.5 (2C), 128.8, 128.7 (2C), 127.4 (2C), 126.9, 124.3, 116.8 (d, $^{3}J_{CF}$ = 8.6 Hz), 116.1, 115.7 (d, $^{2}J_{CF}$ = 23.0 Hz), 114.4 (d, $^{3}J_{CF}$ = 8.6 Hz), 108.4 (d, $^{2}J_{CF}$ = 25.9 Hz), 29.63, 29.56; ^{19}F NMR (376 MHz, $CDCl_3$) δ –121.1; IR (KBr) 3060, 1734, 1647 cm^{-1}; HRMS (ESI) *m/z*: $[M+Na]^+$ calcd for $C_{26}H_{19}FN_2NaO_2$ 433.1328, found 433.1312.

1-Acetyl-7-bromo-5-methyl-2,3-diphenyl-1,5-dihydro-4*H*-pyrrolo[3,2-*c*]quinolin-4-one (3fa)

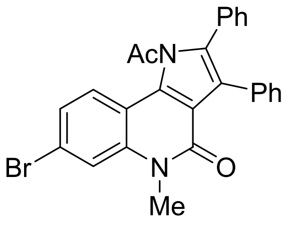

3fa

3fa was prepared following the representative procedure and purified by flash column chromatography on silica gel (Hexane/EtOAc = 3:1).

Analytical data for 3fa: 73% yield; yellow solid (mp 191.3–192.5 °C); ^{1}H NMR (400 MHz, $CDCl_3$) δ 7.95 (d, *J* = 2.3 Hz, 1H), 7.57 (dd, *J* = 9.2, 2.3 Hz, 1H), 7.37-7.31 (m, 3H), 7.28-7.23 (m, 8H), 3.82 (s, 3H), 2.26 (s, 3H); ^{13}C NMR (100 MHz, $CDCl_3$) δ 175.9, 158.8, 137.1, 134.9, 132.5, 131.3, 130.9 (2C), 130.7, 130.6 (2C), 128.8, 128.7, 128.6 (2C), 127.4 (2C), 126.9, 124.9, 124.2, 117.0, 116.0, 115.2, 114.7, 29.50, 29.46; IR (KBr) 3056, 1751, 1653 cm^{-1}; HRMS (ESI) *m/z*: $[M+Na]^+$ calcd for $C_{26}H_{19}BrN_2NaO_2$ 493.0528, found 493.0540.

1-Acetyl-7-methoxy-5-methyl-2,3-diphenyl-1,5-dihydro-4*H*-pyrrolo[3,2-*c*]quinolin-4-one (3ga)

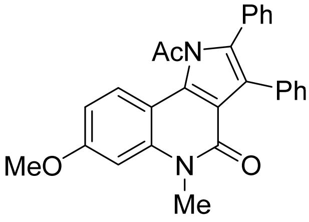

3ga

3ga was prepared following the representative procedure and purified by flash column chromatography on silica gel (Hexane/EtOAc = 3:1).

Analytical data for 3ga: 72% yield; white solid (mp 183.8–184.9 °C); ^{1}H NMR (400 MHz, $CDCl_3$) δ 7.78 (d, *J* = 8.7 Hz, 1H), 7.36-7.19 (m, 10H), 6.92 (d, *J* = 2.3 Hz, 1H), 6.83 (dd, *J* = 8.7, 2.3 Hz, 1H), 3.93 (s, 3H), 3.71 (s, 3H), 2.25 (s, 3H); ^{13}C NMR (100 MHz, $CDCl_3$) δ 176.5, 159.7, 159.4, 140.1, 133.33, 133.28, 133.0, 131.0 (2C), 130.6 (2C), 128.5 (2C), 128.4, 127.3 (2C), 126.7, 124.0, 123.8, 113.6, 108.4, 107.5, 100.4, 55.5, 29.5, 29.4, One C*sp*^{2} signal is missing due to overlapping; IR (KBr) 3006, 1735, 1641 cm^{-1}; HRMS (ESI) *m/z*: $[M+Na]^+$ calcd for $C_{27}H_{22}N_2NaO_3$ 445.1528, found 445.1509.

5-Methyl-2,3-diphenyl-1,5-dihydro-4*H*-pyrrolo[3,2-*c*]quinolin-4-one (3ba')

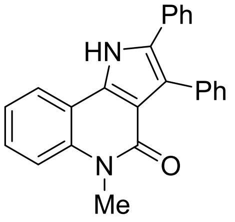

3ba'

A Schlenk tube was charged with $[Cp^*RhCl_2]_2$ (3.1 mg, 0.0050 mmol, 2.5 mol%), $Cu(OAc)_2 \cdot H_2O$ (8.0 mg, 0.040 mmol, 20 mol%), **1b** (43.2 mg, 0.20 mmol), **2a** (71.3 mg, 0.40 mmol), and DMF (2.0 mL). The reaction mixture was stirred at 110 °C for 19 h under air atmosphere using an oil bath. Then, the mixture was cooled to room temperature and filtered through a pad of Celite® with $CHCl_3$ to remove the metal salts. After concentration in vacuo, the residue was purified by silica gel column chromatography (Hexane/EtOAc = 3:1) to give **3ba'** (38.1 mg, 54%) as a yellow solid (mp 217.2–218.1 °C).

Analytical data for 3ba': ^{1}H NMR (400 MHz, $CDCl_3$) δ 9.18 (br s, 1H), 7.84-7.77 (m, 1H), 7.51-7.40 (m, 4H), 7.31-7.20 (m, 9H), 3.72 (s, 3H); ^{13}C NMR (100 MHz, $CDCl_3$) δ 159.9, 137.6, 134.0, 133.9, 132.5, 131.9, 131.0 (2C), 128.4 (2C), 127.9 (2C), 127.6 (2C), 127.3, 126.6, 121.7, 121.2, 120.6, 115.2, 113.62, 113.58, 29.0, One C*sp*^{2} signal is missing due to overlapping; IR (neat) 3178, 1615, 1575 cm^{-1}; HRMS (ESI) *m/z*: $[M+Na]^+$ calcd for $C_{24}H_{18}N_2NaO$ 373.1317, found 373.1303.

5. Isolation and Characterization of C5-Functionalized 2-Quinolone Derivatives 4 and 5

(*E*)-*N*-(5-(1,2-Diphenylvinyl)-1-methyl-2-oxo-1,2-dihydroquinolin-4-yl)acetamide (4ba)

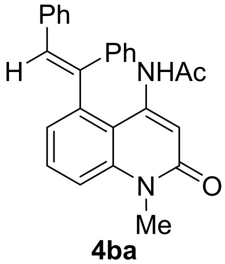

4ba

This product was observed when Cp*Rh(OAc)$_2$·H$_2$O (3.7 mg, 0.010 mmol, 5 mol%) was used instead of [Cp*RhCl$_2$]$_2$ in the representative procedure (Table 1, entry 5). Purified by silica gel column chromatography (Hexane/EtOAc = 3:1 to 1:1) afforded **4ba** (3.9 mg, 5%) as a yellow solid along with **3ba** (51.0 mg, 65%). Colorless crystals (mp 123.0–123.6 °C) for X-ray crystallographic analysis were obtained by recrystallization from toluene. **Analytical data for 4ba:** ^{1}H NMR (400 MHz, CDCl$_3$) δ 9.32 (br s, 1H), 7.72 (br s, 1H), 7.54 (dd, *J* = 8.5, 7.6 Hz, 1H), 7.42 (dd, *J* = 8.5, 1.1 Hz, 1H), 7.30-7.22 (m, 8H), 7.19-7.15 (m, 2H), 7.01 (dd, *J* = 7.3, 0.9 Hz, 1H), 6.87 (s, 1H), 3.74 (s, 3H), 2.01 (s, 3H); ^{13}C NMR (100 MHz, CDCl$_3$) δ 168.1, 162.4, 142.3, 142.1, 141.5, 140.1, 138.1, 135.4, 132.2, 129.6, 129.3 (2C), 129.2 (2C), 128.8 (2C), 128.6 (2C), 128.3, 126.4, 114.8, 113.3, 109.9, 30.0, 25.5, One C*sp*^{2} signal is missing due to overlapping; IR (KBr) 3378, 1712, 1650 cm^{-1}; HRMS (ESI) *m/z*: [M+Na]$^+$ calcd for $C_{26}H_{22}N_2NaO_2$ 417.1579, found 417.1568.

tert-Butyl 1-methyl-2-oxo-5,6-diphenyl-1,2-dihydro-4*H*-benzo[*de*][1,6]naphthyridine-4-carboxylate (5aa)

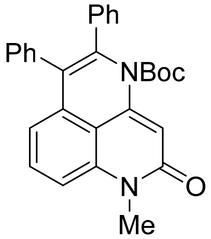

5aa

A Schlenk tube was charged with [Cp*RhCl$_2$]$_2$ (3.1 mg, 0.0050 mmol, 2.5 mol%), Cu(OAc)$_2$·H$_2$O (7.9 mg, 0.40 mmol, 20 mol%), **1a** (55.1 mg, 0.20 mmol), **2a** (71.2 mg, 0.40 mmol), and DCE (2.0 mL). The reaction mixture was stirred at 110 °C for 24 h under air atmosphere using an oil bath. Then, the mixture was cooled to room temperature and filtered through a pad of Celite® with CHCl$_3$ to remove the metal salts. After concentration in vacuo, the residue was purified by silica gel column chromatography (Hexane/EtOAc = 1:1) to give **5aa** (72.8 mg, 78%) as a yellow solid (mp 242.2–242.7 °C). **Analytical data for 5aa:** ^{1}H NMR (400 MHz, CDCl$_3$) δ 7.38 (dd, *J* = 8.0, 8.0 Hz, 1H), 7.25-7.13 (m, 8H), 7.06 (dd, *J* = 7.8, 1.4 Hz, 2H), 7.0 (d, *J* = 8.2 Hz, 1H), 6.54 (d, *J* = 7.8 Hz, 1H), 6.29 (s, 1H), 3.60 (s, 3H), 1.08 (s, 9H); ^{13}C NMR (100 MHz, CDCl$_3$) δ 163.2, 150.5, 144.9, 140.7, 136.8, 135.6, 135.1, 133.6, 131.7, 131.2 (2C), 130.2 (2C), 128.3 (2C), 127.7 (2C), 127.1, 120.0, 116.0, 113.5, 110.8, 93.4, 85.5, 29.2, 28.2, 26.8 (3C); IR (KBr) 2981, 1758, 1634 cm^{-1}; HRMS (ESI) *m/z*: [M+Na]$^+$ calcd for $C_{29}H_{26}N_2NaO_3$ 473.1841, found 473.1841.

4-Acetyl-1-methyl-5,6-diphenyl-1*H*-benzo[*de*][1,6]naphthyridin-2(4*H*)-one (5ba)

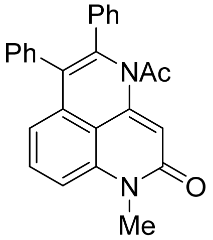

5ba

A Schlenk tube was charged with $[Cp^*RhCl_2]_2$ (3.1 mg, 0.0050 mmol, 2.5 mol%), $AgSbF_6$ (7.1 mg, 0.020 mmol, 10 mol%), $Cu(OAc)_2{\cdot}H_2O$ (79.8 mg, 0.40 mmol), **1b** (43.2 mg, 0.20 mmol), **2a** (71.3 mg, 0.40 mmol), and DCE (2.0 mL). The reaction mixture was stirred at 110 °C for 19 h under Ar atmosphere using an oil bath. Then, the mixture was cooled to room temperature and filtered through pad of Celite® with $CHCl_3$ to remove the metal salts. After concentration in vacuo, the residue was purified by silica gel column chromatography (Hexane/EtOAc = 4:1 to 2:1) to give **5ba** (37.2 mg, 47%) as a yellow solid (along with **3ba** (34.0 mg, 43%). The obtained solid was reprecipitated from $CHCl_3$ and hexane for melting point determination (mp 171.2–172.0 °C). **Analytical data for 5ba:** ^{1}H NMR (400 MHz, $CDCl_3$) δ 7.42 (t, *J* = 8.2 Hz, 1H), 7.34-7.22 (m, 4H), 7.20-7.05 (m, 7H), 6.72 (s, 1H), 6.67 (d, *J* = 7.8 Hz, 1 H), 3.64 (s, 3H), 1.95 (s, 3H); ^{13}C NMR (100 MHz, $CDCl_3$) δ 169.6, 161.6, 140.8, 139.2, 138.5, 136.2, 134.5, 132.8, 130.9, 129.5 (2C), 129.2 (2C), 128.5 (2C), 128.0 (2C), 127.7, 127.3, 126.2, 122.1, 118.1, 114.3, 30.0, 23.3, One Csp^2 signal is missing due to overlapping; IR (neat) 3001, 1625, 1592 cm^{-1}; HRMS (DART) *m/z*: $[M+H]^+$ calcd for $C_{26}H_{21}N_2O_2$ 393.1603, found 393.1615.

4-Acetyl-1,3-dimethyl-5,6-diphenyl-1*H*-benzo[*de*][1,6]naphthyridin-2(4*H*)-one (5ha)

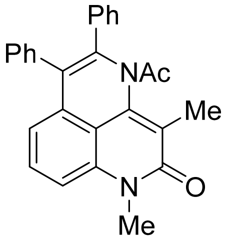

5ha

A Schlenk tube was charged with $Cp^*Rh(OAc)_2{\cdot}H_2O$ (3.7 mg, 0.010 mmol, 5 mol%), $Cu(OAc)_2{\cdot}H_2O$ (7.9 mg, 0.040 mmol), **1h** (46.1 mg, 0.20 mmol), **2a** (71.1 mg, 0.40 mmol), and $PhCF_3$ (2.0 mL). The reaction mixture was stirred at 110 °C for 43 h under Ar atmosphere using oil bath. Then, the mixture was cooled to room temperature and filtered through pad of Celite® with $CHCl_3$ to remove the metal salts. After concentration in vacuo, the residue was purified by silica gel column chromatography (Hexane/EtOAc = 2:1 to 1:1 to EtOAc only) to give **5ha** (29.4 mg, 36%) as a white solid along with **4ha** (6.5 mg, 8%). Colorless crystals (mp 213.1–213.5 °C) for X-ray crystallographic analysis were obtained by recrystallization from $CHCl_3$ and hexane. **Analytical data for 5ha:** ^{1}H NMR (400 MHz, $CDCl_3$) δ 7.42-7.33 (m, 4H), 7.25-7.20 (m, 3H), 7.20-7.14 (m, 5H), 6.85 (d, *J* = 7.8 Hz, 1H), 3.76 (s, 3H), 2.31 (s, 3H), 1.87 (s, 3H); ^{13}C NMR (100 MHz, $CDCl_3$) δ 169.2, 162.9, 139.8, 138.0, 137.0, 136.2, 136.0, 131.2, 130.4 (2C), 130.2, 129.5, 129.2 (2C), 128.7 (2C), 128.1 (2C), 127.9, 127.6, 124.9, 119.6, 116.7, 112.7, 30.0, 25.5, 15.6; IR (neat) 2922, 1695, 1641 cm^{-1}; HRMS (ESI) *m/z*: $[M+Na]^+$ calcd for $C_{27}H_{22}N_2NaO_2$ 429.1579, found 429.1589.

6. Synthesis and Characterization of 2-Quinolone substrates 1

Synthesis and Characterization of 1a and 1b

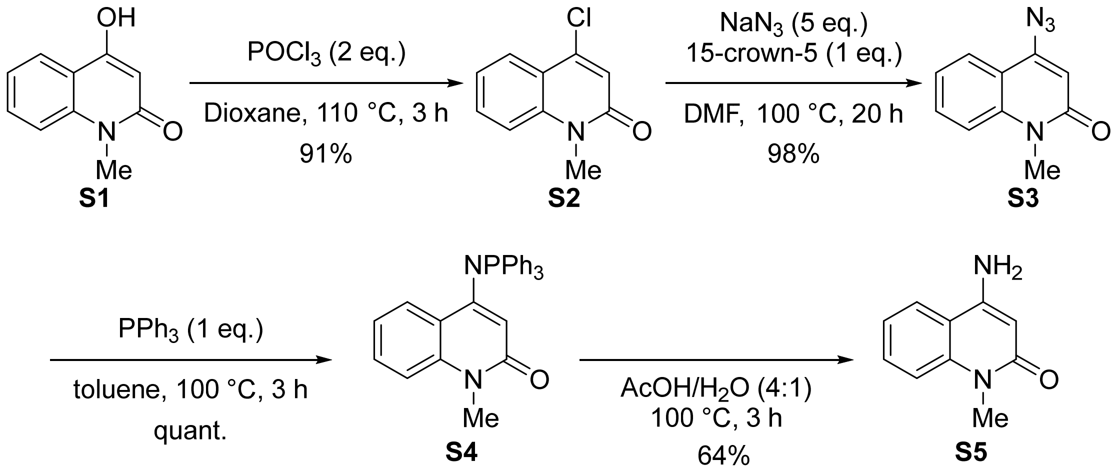

4-Chloro-1-methylquinolin-2(1*H*)-one (S2)[11]

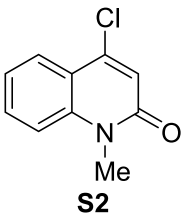

To a dioxane suspension (20 mL) of **S1** (3.50 g, 20.0 mmol) was added $POCl_3$ (6.13 g, 40.0 mmol) at room temperature in a 200 mL flask. The reaction mixture was stirred at 110 °C for 2 h. After cooling to room temperature, the mixture was poured into 40 mL of iced water. The resulting solution was neutralized with 0.5 M NaOH aq. The solution was extracted with diethyl ether (3 x 40 mL). The combined organic layer was washed with water (2 x 60 mL) and brine (40 mL), dried over $MgSO_4$, and concentrated in vacuo. Reprecipitation of the product from ethanol to give **S2** (3.53 g, 91%) as a white amorphous solid. **Analytical data for S2:** ^{1}H NMR (400 MHz, $CDCl_3$) δ 8.03 (dd, *J* = 8.2, 1.4 Hz, 1H), 7.65 (ddd, *J* = 8.6, 7.2, 1.4 Hz, 1H), 7.41 (d, *J* = 8.7 Hz, 1H), 7.33 (ddd, *J* = 7.6, 7.6, 0.9 Hz, 1H), 6.91 (s, 1H), 3.72 (s, 3H); ^{13}C NMR (100 MHz, $CDCl_3$) δ 160.9, 144.2, 139.7, 131.8, 126.2, 122.6, 121.0, 119.2, 114.3, 29.5; IR (neat) 3085, 1644 cm^{-1}; HRMS (DART) *m/z*: $[M+H]^+$ calcd for $C_{10}H_9ClNO$ 194.0373, found 194.0390.

4-Azido-1-methylquinolin-2(1*H*)-one (S3)

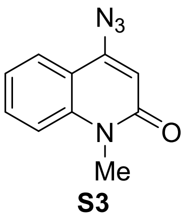

To a DMF suspension (50 mL) of **S2** (3.87 g, 20.0 mmol) and NaN_3 (6.61 g, 101.6 mmol) was added 15-crown-5 (4.0 mL, 20.0 mmol) at room temperature in a 200 mL flask. The reaction mixture was stirred at 100 °C for 20 h. After cooling to room temperature, the mixture was poured into water (100 mL). The precipitated solid was filtered, washed with water (50 ml) and hexane (20 ml). The product **S3** (3.92 g, 98%) was obtained as a yellow solid (mp 246.5–248.3 °C). **Analytical data for S3:** ^{1}H NMR (400 MHz, $CDCl_3$) δ 7.85 (dd, *J* = 8.2, 1.4 Hz, 1H), 7.63 (ddd, *J* = 8.5, 7.1, 1.4 Hz, 1H), 7.37 (d, *J* = 8.2 Hz, 1H), 7.25-7.23 (m, 1H), 6.48 (s, 1H), 3.71 (s, 3H); ^{13}C NMR (100 MHz, $CDCl_3$) δ 161.8, 148.4, 140.0, 131.9, 123.9, 122.0, 115.8, 114.2, 106.6, 29.3; IR (neat) 2128, 1639 cm^{-1}; HRMS (DART) *m/z*: $[M+H]^+$ calcd for $C_{10}H_9N_4O$ 201.0776, found 201.0775.

1-Methyl-4-((triphenyl-l^5-phosphaneylidene)amino)quinolin-2(1*H*)-one (S4)

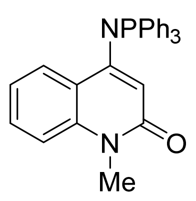

S4

A suspension of **S3** (2.00 g, 10.0 mmol) and PPh_3 (2.62 g, 10.0 mmol) in toluene (20 mL) was stirred at 100 °C for 3 h. After cooling to room temperature, the mixture was concentrated in vacuo. The residue was triturated with diethyl ether (50 mL) and filtered to give **S4** (4.34 g, quant.) as a white solid (mp 244.5–246.3 °C). **Analytical data for S4:** ^{1}H NMR (400 MHz, $CDCl_3$) δ 8.68 (dd, *J* = 8.0, 1.8 Hz, 1H), 7.83-7.78 (m, 6H), 7.59-7.54 (m, 3H), 7.52-7.47 (m, 7H), 7.28-7.23 (m, 2H), 5.58 (d, *J* = 0.9 Hz, 1H), 3.60 (s, 3H); ^{13}C NMR (100 MHz, $CDCl_3$) δ 163.9, 157.0, 140.3, 132.6 (d, *J* = 9.6 Hz, C6), 132.2 (d, *J* = 1.9 Hz, C3), 129.9, 129.0 (d, *J* = 100.6 Hz, C3), 128.9 (d, *J* = 12.5 Hz, C6), 126.2, 123.4 (d, *J* = 24.9 Hz), 120.6, 113.6, 103.2 (d, *J* = 11.5 Hz), 28.6; ^{31}P NMR (162 MHz, $CHCl_3$) δ 10.02; IR (neat) 1614, 1278 cm^{-1}; HRMS (ESI) *m/z*: $[M+Na]^+$ calcd for $C_{28}H_{23}N_2NaOP$ 457.1446, found 457.1428.

4-Amino-1-methylquinolin-2(1*H*)-one (S5)

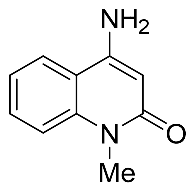

S5

A solution of **S4** (1.30 g, 3.00 mmol) in AcOH (22.5 mL) and H_2O (7.5 mL) was stirred at 100 °C for 3 h. After cooling to room temperature, the mixture was neutralized with 0.5 M NaOH aq. The mixture was extracted with DCM (3 x 20 mL). The combined organic layer was washed with water (2 x 20 mL) and brine (20 mL), dried over Na_2SO_4, and concentrated in vacuo. The residue was purified by silica gel column chromatography (EtOAc/MeOH = 9:1) to furnish **S5** (334.5 mg, 64%) as a white solid (mp 216.5–217.2 °C). **Analytical data for S5:** ^{1}H NMR (400 MHz, $CDCl_3$) δ 7.59 (td, *J* = 7.9, 1.1 Hz, 1H),7.55 (d, *J* = 7.8 Hz, 1H), 7.37 (d, *J* = 8.7 Hz, 1H), 7.23 (m, 1H), 5.93 (s, 1H), 4.48 (br s, 2H), 3.67 (s, 3H); ^{13}C NMR (100 MHz, DMSO-d_6) δ 162.1, 152.0, 140.1, 130.7, 123.0, 120.5, 114.72, 114.67, 93.3, 28.1; IR (neat) 3196, 2873, 1625 cm^{-1}; HRMS (DART) *m/z*: $[M+H]^+$ calcd for $C_{10}H_{11}N_2O$ 175.0871, found 175.0855.

During our study, we found that **S5** can also be prepared from **S3** as shown below.

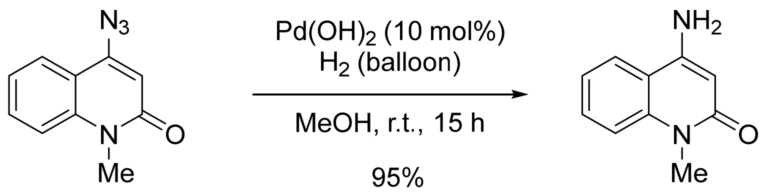

S3 **S5**

To a MeOH suspension (100 ml) of **S3** (3.92 g, 19.6 mmol) was added $Pd(OH)_2$ (280.8 mg, 2 mmol) under Ar atmosphere. Subject the vessel twice to evacuation followed by purging with a hydrogen balloon. After the reaction mixture was stirred at room temperature for 15 h, filtered through a pad of Celite® with MeOH to remove the metal salts, and concentrated in vacuo to afford **S5** (3.24 mg, 95%) as a yellow solid.

tert-Butyl (1-methyl-2-oxo-1,2-dihydroquinolin-4-yl)carbamate (1a)

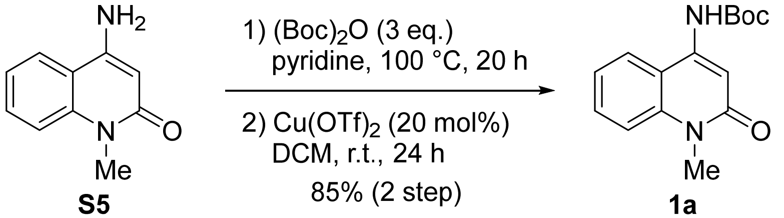

S5 **1a**

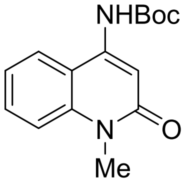

1a

To a pyridine solution (1.5 mL) of **S5** (541.5 mg, 3.11 mmol) was added Boc_2O (2.1 mL, 9.00 mmol) in a 100 mL flask. The reaction mixture was stirred at 80 °C for 20 h. After cooling to room temperature, the resulting mixture was concentrated in vacuo. The residue was dissolved in DCM (6 ml), to which $Cu(OTf)_2$ (228.0 mg, 0.630 mmol) was added, and the mixture was stirred at room temperature for 24 h. The reaction mixture was quenched with water (20 mL) and the whole mixture was extracted with DCM (3 x 20 mL). The combined organic layer was washed with water (2 x 20 mL), brine (20 mL), and dried over $MgSO_4$, and concentrated. The residue was purified by a silica gel column chromatography to furnish **1a** (724.2 mg, 85%) as a yellow amorphous solid. **Analytical data for 1a:** ^{1}H NMR (400 MHz, $CDCl_3$) δ 7.65-7.51 (m, 2H), 7.45-7.36 (m, 2H), 7.30-7.22 (m, 1H), 6.93 (br s, 1H), 3.69 (s, 3H), 1.56 (s, 9H); ^{13}C NMR (100 MHz, CD_3OD) δ 162.9, 151.9, 142.1, 140.1, 131.0, 121.8, 121.0, 115.1, 106.7, 82.1, 29.3, 28.7, 28.3 (3C); IR (neat) 3234, 2980, 1736, 1637, 1618, 1583, 1537, 1152 cm^{-1}; HRMS (DART) *m/z* $[M+H]^+$ calcd for $C_{15}H_{19}N_2O_4$ 275.1396, found 275.1403.

N-(1-Methyl-2-oxo-1,2-dihydroquinolin-4-yl)acetamide (1b)

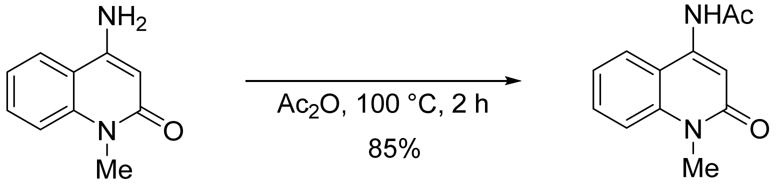

S5 **1b**

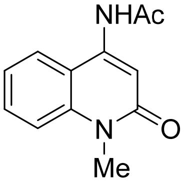

1b

An Ac_2O solution (5 mL) of **S5** (334.5 g, 1.92 mmol) was stirred at 100 °C for 2 h. After cooling to room temperature, the mixture was concentrated in vacuo. The residue was purified by silica gel column chromatography (EtOAc/MeOH = 19:1) to furnish **1b** (348.8 mg, 84%) as a white solid (mp 213.8–216.4 °C). **Analytical data for 1b:** ^{1}H NMR (400 MHz, $CDCl_3$) δ 7.66-7.46 (m, 4H), 7.42 (d, J = 8.7 Hz, 1H), 7.32-7.27 (m, 1H), 3.71 (s, 3H), 2.32 (s, 3H); ^{13}C NMR (100 MHz, $CDCl_3$) δ 171.7, 161.8, 146.7, 140.5, 131.9, 123.4, 123.0, 122.6, 118.4, 115.1, 29.7, 26.3; IR (neat) 3309, 1708, 1638, 1579 cm^{-1}; HRMS (DART) *m/z*: $[M+H]^+$ calcd for $C_{12}H_{13}N_2O_2$ 217.0977, found 217.0985.

***N*-(1-Methyl-2-oxo-1,2-dihydroquinolin-4-yl)pivalamide (1b')**

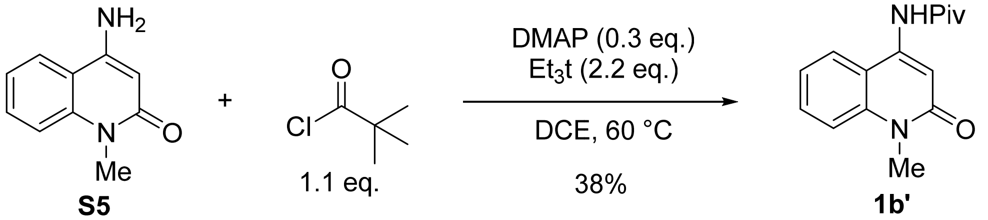

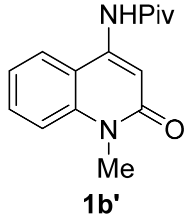

To a suspension of **S5** (88.8 mg, 0.51 mmol), Et_3N (153 μL, 1.10 mmol) and DMAP (18.4 mg, 0.15 mmol) in DCE (3 mL) was added pivaloyl chloride (67 μL, 0.55 mmol) at room temperature under Ar atmosphere. The reaction mixture was stirred at 60 °C for 96 h. After cooling to room temperature, the mixture was diluted with water and the aqueous layer was extracted with DCM (3 x 20 mL). The combined organic layer was washed with water (2 x 40 mL) and brine (20 mL), dried over Na_2SO_4, and concentrated in vacuo. The residue was purified by silica gel column chromatography (EtOAc only) to furnish **1b'** (50.4 mg, 38%) as a white solid (mp 208.8–210.5 °C). **Analytical data for 1b':** ^{1}H NMR (400 MHz, $CDCl_3$) δ 7.78 (br s, 1H), 7.64-7.59 (m, 2H), 7.48-7.41 (m, 2H), 7.31-7.26 (m, 1H), 3.71 (s, 3H), 1.41 (s, 9H); ^{13}C NMR (100 MHz, $CDCl_3$) δ 176.7, 162.7, 140.9, 140.2, 131.0, 121.8, 120.2, 115.6, 115.2, 109.9, 40.5, 29.3, 27.6 (3C); IR (neat) 3308, 1638, 1618, 1584 cm^{-1}; HRMS (DART) *m/z*: $[M+H]^+$ calcd for $C_{15}H_{19}N_2O_2$ 259.1447, found 259.1417.

Synthesis and Characterization of 1c-g

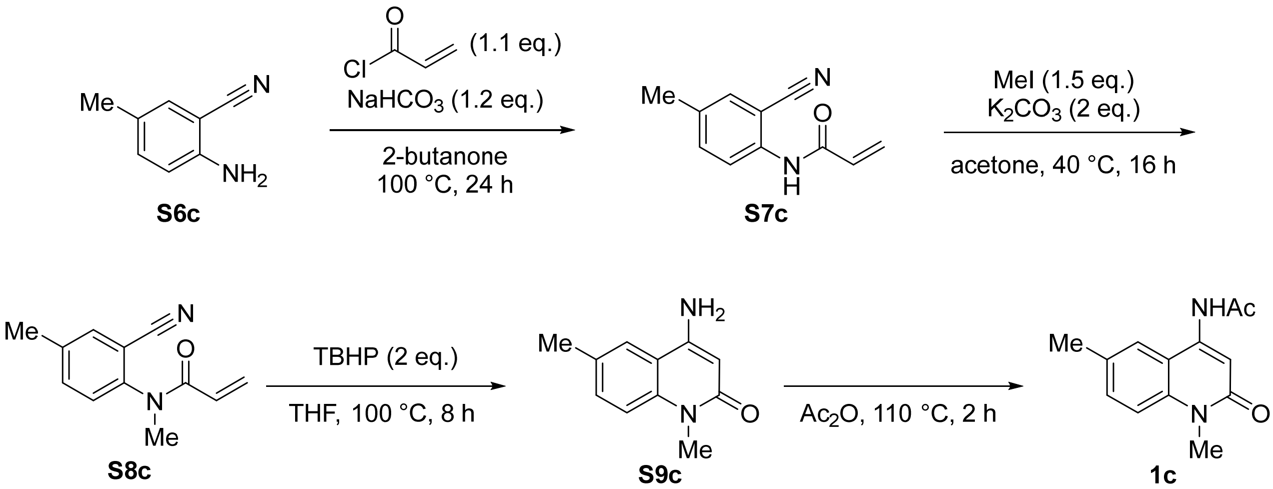

***N*-(2-Cyano-4-methylphenyl)acrylamide (S7c)**

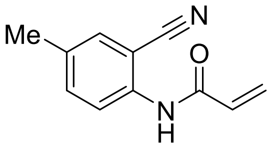

S7c

To a suspension of **S6c**[12] (1.18 g, 8.9 mmol) and $NaHCO_3$ (901.5 mg, 10.7 mmol) in 2-butanone (30 mL) was added acryloyl chloride (792 μL, 9.8 mmol) at room temperature under Ar atmosphere. The reaction mixture was stirred at 100 °C for 24 h. After cooling to room temperature, the mixture was diluted with water and the aqueous layer was extracted with DCM (3 x 20 mL). The combined organic layer was washed with water (2 x 40 mL) and brine (40 mL), dried over Na_2SO_4, and concentrated in vacuo. The residue was purified by silica gel column chromatography (Hexane/EtOAc = 8:1) to furnish **S7c** (1.30 g, 78%) as a white solid. The obtained solid was reprecipitated from $CHCl_3$ and hexane for melting point determination (mp 189.1–189.5 °C). **Analytical data for S7c:** ^1H NMR (400 MHz, $CDCl_3$) δ 8.37 (d, *J* = 8.2 Hz, 1H), 7.63 (br s, 1H), 7.46-7.36 (m, 2H), 6.49 (dd, *J* = 16.9, 0.9 Hz, 1H), 6.31 (ddd, *J* = 17.2, 10.3, 0.9 Hz, 1H), 5.87 (dd, *J* = 10.0, 0.9 Hz, 1H), 2.35 (s, 3H); ^{13}C NMR (100 MHz, $CDCl_3$) δ 163.5, 137.9, 135.1, 134.4, 132.2, 130.5, 129.2, 121.3, 116.5, 101.9, 20.5; IR (KBr) 3675, 2230, 1662 cm^{-1}; HRMS (DART) *m/z*: $[M+H]^+$ calcd for $C_{11}H_{11}N_2O$ 187.0871, found 187.0856.

***N*-(2-Cyano-4-methylphenyl)-*N*-methylacrylamide (S8c)**

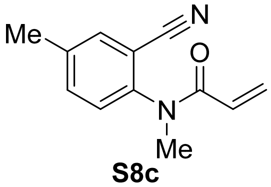

S8c

To a suspension of **S7c** (1.30 g, 7.0 mmol) and K_2CO_3 (2.13 g, 15.4 mmol) in acetone (35 mL) was added MeI (654 μL, 10.5 mmol) at room temperature under Ar atmosphere. The reaction mixture was stirred at 40 °C for 16 h. After cooling to room temperature, the mixture was concentrated in vacuo. The residue was purified by silica gel column chromatography (Hexane/EtOAc = 4:1) to give **S8c** (1.32 g, 94%) as a white amorphous solid. **Analytical data for S8c:** ^1H NMR (400 MHz, $CDCl_3$) δ 7.55 (s, 1H), 7.47 (dd, *J* = 8.2, 1.4 Hz, 1H), 7.20 (d, *J* = 7.8 Hz, 1H), 6.42 (d, *J* = 16.9 Hz, 1H), 5.90 (dd, *J* = 16.7, 10.3 Hz, 1H), 5.57 (d, *J* = 10.1 Hz, 1H), 3.37 (s, 3H), 2.44 (s, 3H); ^{13}C NMR (100 MHz, $CDCl_3$) δ 165.4, 143.4, 139.1, 135.0, 134.2, 129.2, 128.8, 127.4, 115.8, 112.2, 36.9, 20.8; IR (KBr) 3036, 2231, 1666 cm^{-1}; HRMS (DART) *m/z*: $[M+H]^+$ calcd for $C_{12}H_{13}N_2O$ 201.1028, found 201.1053.

***N*-(1,6-Dimethyl-2-oxo-1,2-dihydroquinolin-4-yl)acetamide (1c)**

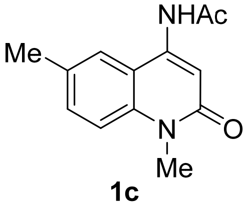

1c

S8c (801 mg, 4.0 mmol), TBHP (70% aqueous solution, 769 µL, 8.0 mmol), and THF (25 mL) were added in a sealed tube with a Teflon-lined cap. The mixture was stirred at 100 °C for 8 h. After cooling to room temperature, the reaction mixture was concentrated in vacuo. The residue was purified by silica gel column chromatography (EtOAc only) to give **S9c**, which still contained a small amount of inseparable impurities. This mixture was used for the next reaction without further purification. An Ac_2O solution (2 mL) of **S9c** was stirred at 100 °C for 2 h. After cooling to room temperature, the mixture was concentrated in vacuo. The residue was purified by silica gel column chromatography (EtOAc/MeOH = 19:1). A white solid (mp 189.9–190.1 °C) was obtained by reprecipitation from $CHCl_3$ and Et_2O to furnish **1c** (237 mg, 18%). **Analytical data for 1c:** ^{1}H NMR (400 MHz, DMSO-d_6) δ 9.79 (br s, 1H), 7.92 (s, 1H), 7.50-7.41 (m, 2H), 7.24 (s, 1H) 3.55 (s, 3H), 2.41 (s, 3H), 2.22 (s, 3H); ^{13}C NMR (100 MHz, DMSO-d_6) δ 170.0, 162.1, 161.5, 142.6, 141.6, 124.9, 109.1, 108.9, 106.0, 99.1, 55.6, 29.0, 24.3; IR (KBr) 3258, 2945, 1645 cm^{-1}; HRMS (DART) *m/z*: $[M+H]^+$ calcd for $C_{13}H_{15}N_2O_2$ 231.1134, found 231.1123.

***N*-(2-Cyano-4-(trifluoromethyl)phenyl)acrylamide (S7d)**

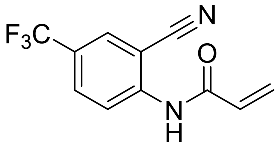

S7d

This compound was prepared from **S6d**[13] in 71% yield in a similar manner to the synthesis of **S7c** from **S6c**. **Analytical data for S7d:** white solid (mp 148.9–150.7 °C); ^{1}H NMR (400 MHz, $CDCl_3$) δ 8.77 (d, J = 8.2 Hz, 1H), 7.91 (br s, 1H), 7.88-7.82 (m, 2H), 6.55 (dd, J = 16.9, 0.9 Hz, 1H), 6.36 (dd, J = 16.9, 11.0 Hz, 1H), 5.96 (dd, J = 10.1, 0.9 Hz, 1H); ^{13}C NMR (100 MHz, $CDCl_3$) δ 163.6, 143.2, 131.2 (q, $^3J_{CF}$ = 3.8 Hz), 130.5, 130.0, 129.5 (q, $^3J_{CF}$ = 3.8 Hz), 126.4 (q, $^2J_{CF}$ = 34.5 Hz), 122.8 (q, $^1J_{CF}$ = 272.2 Hz), 121.1, 115.1, 101.7; ^{19}F NMR (376 MHz, $CDCl_3$): δ –63.6; IR (KBr) 3340, 2236, 1689 cm^{-1}; HRMS (DART) *m/z*: $[M+H]^+$ calcd for $C_{11}H_8F_3N_2O$ 241.0559, found 241.0586.

***N*-(2-Cyano-4-(trifluoromethyl)phenyl)-*N*-methylacrylamide (S8d)**

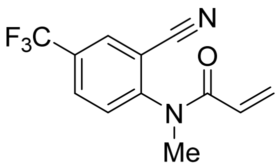

S8d

This compound was prepared from **S7d** in 82% yield in a similar manner to the synthesis of **S8c** from **S7c**. **Analytical data for S8d:** white amorphous solid; ^{1}H NMR (400 MHz, $CDCl_3$) δ 8.02 (d, J = 1.8 Hz, 1H), 7.93 (dd, J = 8.5, 2.0 Hz, 1H), 7.49 (d, J = 8.2 Hz, 1H), 6.75 (s, 1H), 6.49 (dd, J = 16.5, 1.4 Hz, 1H), 5.94 (br s, 1H), 5.70 (d, J = 10.1 Hz, 1H), 3.44 (s, 3H); ^{13}C NMR (100 MHz, $CDCl_3$) δ 165.1, 149.2, 131.1 (q, $^3J_{CF}$ = 2.8 Hz), 130.942 (q, $^3J_{CF}$ = 2.8 Hz), 130.942 (q, $^2J_{CF}$ = 35.5Hz), 130.2, 130.1, 126.9, 122.5 (q, $^1J_{CF}$ =273.2 Hz), 114.6, 113.3, 37.2; ^{19}F NMR (376 MHz, $CDCl_3$): δ –63.9; IR (KBr) 3079, 2233, 1668 cm^{-1}; HRMS (DART) *m/z*: $[M+H]^+$ calcd for $C_{12}H_{11}F_3N_2O$ 255.0745, found 255.0771.

N-(1-Methyl-2-oxo-6-(trifluoromethyl)-1,2-dihydroquinolin-4-yl)acetamide (1d)

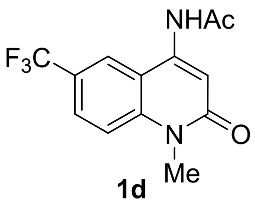

1d

This compound was prepared from **S8d** in 23% yield in a similar manner to the synthesis of **1c** from **S8c**. **Analytical data for 1d:** yellow solid (mp 196.4–198.4 °C); ^{1}H NMR (400 MHz, DMSO-d_6) δ 10.06 (s, 1H), 8.52 (s, 1H), 7.96 (d, *J* = 9.2 Hz, 1H), 7.73 (d, *J* = 8.7 Hz, 1H), 7.39 (s, 1H), 3.62 (s, 3H), 2.25 (s, 3H); ^{13}C NMR (100 MHz, DMSO-d_6) δ 170.3, 161.6, 142.1 (q, $^3J_{CF}$ = 3.8 Hz), 127.20, 127.17, 124.4 (q, $^1J_{CF}$ = 271.5 Hz), 121.9 (q, $^2J_{CF}$ = 32.6 Hz), 120.9, 116.2, 115.1, 109.6, 29.2, 24.4; ^{19}F NMR (376 MHz, DMSO-d_6): δ –62.6; IR (KBr) 3275, 2959, 1684 cm^{-1}; HRMS (DART) *m/z*: $[M+H]^+$ calcd for $C_{13}H_{12}F_3N_2O_2$ 285.0851, found 285.0865.

N-(2-Cyano-4-fluorophenyl)acrylamide (S7e)

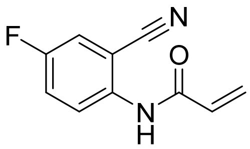

S7e

This compound was prepared from 2-amino-5-fluorobenzonitrile **S6e**[12, 14] in 75% yield in a similar manner to the synthesis of **S7c** from **S6c**. **Analytical data for S7e:** white solid (mp 161.0–162.3 °C); ^{1}H NMR (400 MHz, $CDCl_3$) δ 8.49 (dd, *J* = 9.4, 4.8 Hz, 1H), 7.69 (br s, 1H), 7.39-7.28 (m, 2H), 6.51 (dd, *J* = 16.5, 0.9 Hz, 1H), 6.32 (dd, *J* = 16.9, 10.1 Hz, 1H), 5.90 (dd, *J* = 10.1, 0.9 Hz, 1H); ^{13}C NMR (100 MHz, $CDCl_3$) δ 163.5, 158.0 (d, $^1J_{CF}$ = 248.2 Hz), 136.9 (d, $^4J_{CF}$ = 2.9 Hz), 130.2, 129.7, 123.6 (q, $^3J_{CF}$ = 7.7 Hz), 121.8 (d, $^2J_{CF}$ = 22.0 Hz), 118.5 (d, $^2J_{CF}$ = 25.9 Hz), 115.2 (d, $^4J_{CF}$ = 2.9 Hz), 103.2 (d, $^3J_{CF}$ = 8.6 Hz); ^{19}F NMR (376 MHz, $CDCl_3$): δ –116.4; IR (KBr) 3271, 2231, 1666 cm^{-1}; HRMS (DART) *m/z*: $[M+H]^+$ calcd for $C_{10}H_8FN_2O$ 191.0621, found 191.0629.

N-(2-Cyano-4-fluorophenyl)-N-methylacrylamide (S8e)

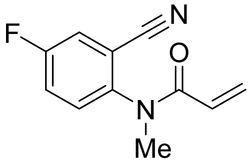

S8e

This compound was prepared from **S7e** in 88% yield in a similar manner to the synthesis of **S8c** from **S7c**. **Analytical data for S8e:** brown solid (mp 91.2–93.5 °C); ^{1}H NMR (400 MHz, $CDCl_3$) δ 7.47 (dd, *J* = 7.3, 2.3 Hz, 1H), 7.43-7.36 (m, 1H), 7.36-7.30 (m, 1H), 6.45 (d, *J* = 16.9 Hz, 1H), 5.87 (dd, *J* = 16.5, 10.5 Hz, 1H), 5.62 (d, *J* = 10.5 Hz, 1H), 3.38 (s, 3H); ^{13}C NMR (100 MHz, $CDCl_3$) δ 165.3, 161.0 (d, $^1J_{CF}$ = 253.0 Hz), 142.4, 131.5 (d, $^3J_{CF}$ = 8.6 Hz), 129.5, 127.0, 121.8 (d, $^2J_{CF}$ = 22.0 Hz), 120.7 (d, $^2J_{CF}$ = 25.9 Hz), 114.5, 114.1 (d, $^3J_{CF}$ = 9.6 Hz), 37.0; ^{19}F NMR (376 MHz, $CDCl_3$): δ –110.6; IR (KBr) 3073, 2235, 1664 cm^{-1}; HRMS (DART) *m/z*: $[M+H]^+$ calcd for $C_{11}H_{10}FN_2O$ 205.0777, found 205.0804.

***N*-(6-Fluoro-1-methyl-2-oxo-1,2-dihydroquinolin-4-yl)acetamide (1e)**

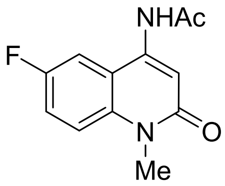
1e

This compound was prepared from **S8e** in 16% yield in a similar manner to the synthesis of **1c** from **S8c**. **Analytical data for 1e:** white solid (mp 225.1–227.5 °C); ^{1}H NMR (400 MHz, DMSO-d_6) δ 9.92 (br s, 1H), 8.04 (dd, J = 10.3, 2.5 Hz, 1H), 7.58-7.48 (m, 2H), 7.31 (s, 1H), 3.56 (s, 3H), 2.21 (s, 3H); ^{13}C NMR (100 MHz, DMSO-d_6) δ 170.2, 161.3, 157.0 (d, $^{1}J_{CF}$ = 238.7 Hz), 141.8 (d, $^{4}J_{CF}$ = 2.9 Hz), 136.5, 118.5 (d, $^{2}J_{CF}$ = 24.0 Hz), 117.1 (d, $^{3}J_{CF}$ = 7.7 Hz), 116.2 (d, $^{3}J_{CF}$ = 8.6 Hz), 109.5, 109.0 (d, $^{2}J_{CF}$ = 24.9 Hz), 29.1, 24.3; ^{19}F NMR (376 MHz, DMSO-d_6): δ –122.6; IR (KBr) 3323, 3061, 1642 cm^{-1}; HRMS (DART) *m/z*: [M+H]$^{+}$ calcd for $C_{12}H_{12}FN_2O_2$ 235.0883, found 235.0861.

***N*-(5-Bromo-2-cyanophenyl)acrylamide (S7f)**

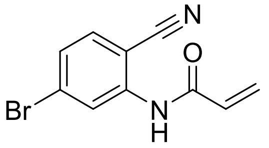
S7f

This compound was prepared from **S6f**[15] in 90% yield in a similar manner to the synthesis of **S7c** from **S6c**. **Analytical data for S7f:** white amorphous solid; ^{1}H NMR (400 MHz, $CDCl_3$) δ 8.48 (d, J = 9.6 Hz, 1H), 7.75-7.69 (m, 2H), 7.65 (br s, 1H), 6.51 (dd, J = 16.9, 0.9 Hz, 1H), 6.31 (dd, J = 16.9, 10.1 Hz, 1H), 5.91 (d, J = 10.5 Hz, 1H); ^{13}C NMR (100 MHz, $CDCl_3$) δ 163.5, 139.5, 137.4, 134.4, 130.2, 129.9, 122.6, 116.4, 115.0, 103.4; IR (KBr) 3342, 2229, 1683 cm^{-1}; HRMS (DART) *m/z*: [M+H]$^{+}$ calcd for $C_{10}H_8BrN_2O$ 250.9820, found 250.9829.

***N*-(5-Bromo-2-cyanophenyl)-*N*-methylacrylamide (S8f)**

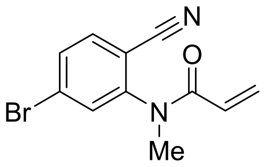
S8f

This compound was prepared from **S7f** in 83% yield in a similar manner to the synthesis of **S8c** from **S7c**. **Analytical data for S8f:** white solid (mp 98.7–99.7 °C); ^{1}H NMR (400 MHz, $CDCl_3$) δ 7.87 (d, J = 2.3 Hz, 1H), 7.79 (dd, J = 8.2, 2.3 Hz, 1H), 7.20 (d, J = 8.7 Hz, 1H), 6.45 (dd, J = 16.9, 1.4 Hz, 1H), 5.89 (br s, 1H), 5.63 (d, J = 8.7 Hz, 1H), 3.38 (s, 3H); ^{13}C NMR (100 MHz, $CDCl_3$) δ 165.1, 145.0, 137.4, 136.4, 130.8, 129.6, 127.0, 121.9, 114.4, 114.2, 36.9; IR (KBr) 2236, 1664 cm^{-1}; HRMS (DART) *m/z*: [M+H]$^{+}$ calcd for $C_{11}H_{10}BrN_2O$ 264.9977, found 264.9962.

***N*-(7-Bromo-1-methyl-2-oxo-1,2-dihydroquinolin-4-yl)acetamide (1f)**

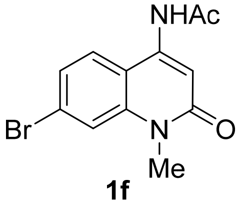
1f

This compound was prepared from **S8f** in 7% yield in a similar manner to the synthesis of **1c** from **S8c**. **Analytical data for 1f:** white amorphous solid; ^{1}H NMR (400 MHz, DMSO-d_6) δ 9.82 (s, 1H), 8.31 (d, J = 1.8 Hz, 1H), 7.75 (dd, J = 9.2, 2.3 Hz, 1H), 7.45 (d, J = 8.7 Hz, 1H), 7.27 (s, 1H), 3.51 (s, 3H), 2.17 (s, 3H); ^{13}C NMR (100 MHz, DMSO-d_6) δ 170.2, 161.3, 141.5, 138.9, 133.5, 125.3, 117.4, 116.8, 113.9, 109.4, 29.0, 24.4; IR (KBr) 3060, 1635, 1612 cm^{-1}; HRMS (DART) *m/z*: [M+H]$^{+}$ calcd for $C_{12}H_{12}BrN_2O_2$ 295.0082, found 295.0105.

***N*-(2-Cyano-5-methoxyphenyl)acrylamide (S7g)**

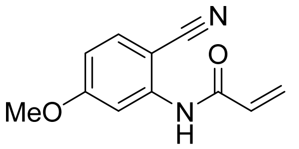

S7g

This compound was prepared from **S6g**[12, 14] in 75% yield in a similar manner to the synthesis of **S7c** from **S6c**. **Analytical data for S7g:** white solid (mp 141.5–143.7 °C); ^{1}H NMR (400 MHz, $CDCl_3$) δ 8.22 (d, *J* = 2.3 Hz, 1H), 7.69 (br s, 1H), 7.49 (d, *J* = 8.7 Hz, 1H), 6.71 (dd, *J* = 8.7, 2.8 Hz, 1H), 6.50 (dd, *J* = 16.9, 0.9 Hz, 1H), 6.31 (dd, *J* = 16.9, 10.1 Hz, 1H), 5.89 (dd, *J* = 10.1, 0.9 Hz, 1H), 3.89 (s, 3H); ^{13}C NMR (100 MHz, $CDCl_3$) δ 164.1, 163.7, 142.3, 133.3, 130.5, 129.5, 116.8, 111.6, 105.5, 93.1, 55.7; IR (KBr) 3240, 2222, 1672 cm^{-1}; HRMS (DART) *m/z*: $[M+H]^+$ calcd for $C_{11}H_{11}N_2O_2$ 203.0821, found 203.0848.

***N*-(2-Cyano-5-methoxyphenyl)-*N*-methylacrylamide (S8g)**

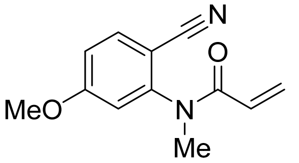

S8g

This compound was prepared from **S7g** in 95% yield in a similar manner to the synthesis of **S8c** from **S7c**. **Analytical data for S8g:** white solid (mp 81.5–82.7 °C); ^{1}H NMR (400 MHz, $CDCl_3$) δ 7.66 (d, *J* = 8.7 Hz, 1H), 6.97 (dd, *J* = 8.7, 2.3 Hz, 1H), 6.80 (d, *J* = 2.3 Hz, 1H), 6.45 (dd, *J* = 16.7, 1.6 Hz, 1H), 5.94 (dd, *J* = 16.3, 10.3 Hz, 1H), 5.61 (d, *J* = 10.1 Hz, 1H), 3.88 (s, 3H), 3.39 (s, 3H); ^{13}C NMR (100 MHz, $CDCl_3$) δ 165.1, 163.7, 147.6, 135.1, 128.8, 127.3, 116.0, 115.2, 114.2, 103.8, 55.9, 36.7; IR (KBr) 2228, 1664 cm^{-1}; HRMS (DART) *m/z*: $[M+H]^+$ calcd for $C_{12}H_{13}N_2O_2$ 217.0977, found 217.0961.

***N*-(7-Methoxy-1-methyl-2-oxo-1,2-dihydroquinolin-4-yl)acetamide (1g)**

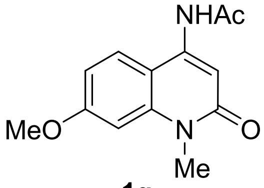

1g

This compound was prepared from **S8g** in 36% yield in a similar manner to the synthesis of **1c** from **S8c**. **Analytical data for 1g:** white solid (mp 251.3–253.4 °C); ^{1}H NMR (400 MHz, DMSO-d_6) δ 9.77 (s, 1H), 8.01 (d, *J* = 8.7 Hz, 1H), 7.10 (s, 1H), 6.96-6.89 (m, 2H), 3.90 (s, 3H), 3.56 (s, 3H), 2.20 (s, 3H); ^{13}C NMR (100 MHz, DMSO-d_6) δ 170.0, 162.0, 161.5, 142.6, 141.6, 124.9, 109.1, 108.9, 106.0, 99.1, 55.6, 28.9, 24.3; IR (KBr) 2956, 1645, 1614 cm^{-1}; HRMS (DART) *m/z*: $[M+H]^+$ calcd for $C_{13}H_{15}N_2O_3$ 247.1083, found 247.1076.

Synthesis and Characterization of 1h

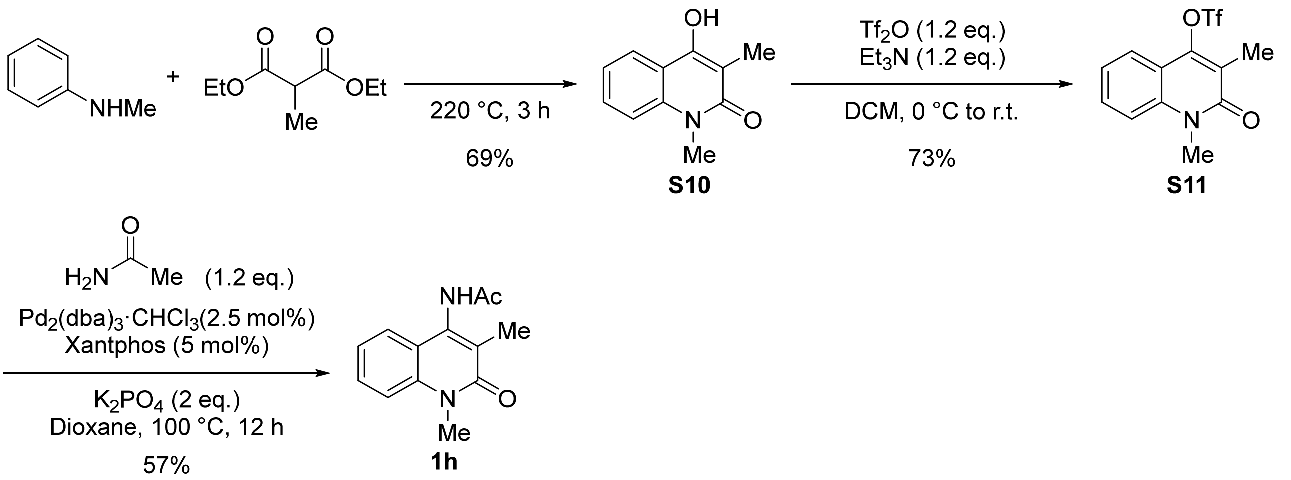

4-Hydroxy-1,3-dimethylquinolin-2(1*H*)-one (S10)[16]

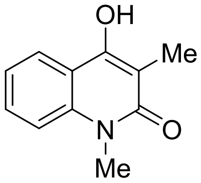

S10

A mixture of diethyl methylmalonate (3.42 mL, 20.0 mmol) and *N*-methylaniline (2.16 mL, 20.0 mmol) was heated to 220 °C in a 10 mL round bottomed flask topped with a short path distillation head. After finishing the generation of EtOH, the mixture solidified upon cooling to room temperature. The crude product was washed with H_2O (40 mL) and DCM (40 mL) and dried in vacuo to afford **S10** (2.59 g, 69%) as a white solid (mp 194.1–195.4 ℃). **Analytical data for S10[17]:** ^{1}H NMR (400 MHz, DMSO-d_6) δ 10.08 (s, 1H), 7.95 (dd, J = 8.2, 1.4 Hz, 1H), 7.53 (t, J = 7.8 Hz, 1H), 7.42 (d, J = 8.2 Hz, 1H), 7.21 (t, J = 7.6 Hz, 1H), 3.56 (s, 3H), 2.03 (s, 3H); ^{13}C NMR (100 MHz, DMSO-d_6) δ 163.0, 155.9, 138.1, 130.0, 122.8, 121.2, 116.2, 114.1, 106.4, 29.1, 10.3; IR (KBr) 3165, 3090, 1644 cm^{-1}; HRMS (DART) *m/z*: $[M+H]^+$ calcd for $C_{11}H_{12}NO_2$ 190.0868, found 190.0887.

1,3-Dimethyl-2-oxo-1,2-dihydroquinolin-4-yl trifluoromethanesulfonate (S11)[18]

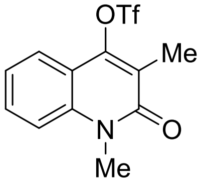

S11

To a solution of **S10** (1.89 g, 10.0 mmol) and Et_3N (1.7 mL, 12.0 mmol) in dry DCM (100 mL) was slowly added Tf_2O (2.0 mL, 12.0 mmol) at 0 °C under Ar atmosphere. The reaction mixture was gradually warmed to room temperature and kept stirring for 5 h. After concentration in vacuo, the residue was purified by silica gel column chromatography (Hexane/EtOAc = 1:1) to give **S11** (2.36g, 73%) as a yellow amorphous solid. **Analytical data for S11[18]:** ^{1}H NMR (400 MHz, $CDCl_3$) δ 7.81 (dd, J = 8.0, 1.1 Hz, 1H), 7.65 (ddd, J = 8.5, 7.1, 1.4 Hz, 1H), 7.44 (d, J = 8.7 Hz, 1H), 7.35 (dd, J = 7.3, 0.9 Hz, 1H), 3.78 (s, 3H), 2.34 (s, 3H); ^{13}C NMR (100 MHz, $CDCl_3$) δ 162.4, 149.6, 138.1, 131.3, 123.3, 122.9, 122.7, 118.5 (q, $^3J_{CF}$ = 320.1 Hz), 115.7, 114.3, 30.4, 12.3; ^{19}F NMR (376 MHz, $CDCl_3$) δ –73.7; IR (KBr) 1639 cm^{-1}; HRMS (DART) *m/z*: $[M+H]^+$ calcd for $C_{12}H_{11}F_3NO_4S$ 322.0361, found 322.0337.

***N*-(1,3-Dimethyl-2-oxo-1,2-dihydroquinolin-4-yl)acetamide (1h)**

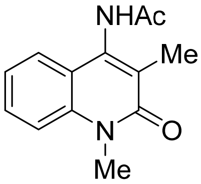
1h

A Schlenk tube was charged with $Pd_2(dba)_3·CHCl_3$ (14.1 mg, 0.025 mmol, 5.0 mol%), Xantphos (14.2 mg, 0.025 mmol, 5.0 mol%), **S11** (160.5 mg, 0.50 mmol), K_3PO_4 (212.5 mg, 1.0 mmol), acetamide (36.1 mg, 0.60 mmol) and dry dioxane (8 mL) under Ar atmosphere. The mixture was stirred at 100 °C for 12 h. After cooling to room temperature, the mixture was diluted with water and the aqueous layer was extracted with DCM (3 x 20 mL). The combined organic layer was washed with water (2 x 40 mL), dried over $MgSO_4$, and concentrated in vacuo. The residue was purified by silica gel column chromatography (EtOAc only) to give **1h** (65.9 mg, 57%) as a white amorphous solid; **Analytical data for 1h:** This compound was observed as a mixture of rotamers (ratio 3.75:1) at 25 ºC. ^{1}H NMR (400 MHz, $CDCl_3$) discernible data for major rotamer: δ 7.60 (d, *J* = 8.2 Hz, 1H), 7.54 (t, *J* = 7.8 Hz, 1H), 7.38 (d, *J* = 8.7 Hz, 1H), 7.26-7.21 (m, 1H), 7.05 (br s, 1H), 3.75 (s, 3H), 2.34 (s, 3H), 2.20 (s, 3H); discernable data for minor rotamer: δ 7.75 (d, *J* = 7.3 Hz, 1H), 7.43 (d, *J* = 8.7 Hz, 1H), 7.32 (t, *J* = 7.3 Hz, 1H), 6.80 (br s, 1H), 3.79 (s, 3H), 2.29 (s, 3H), 1.84 (s, 3H); ^{13}C NMR (100 MHz, DMSO-d_6) δ 168.2, 161.9, 139.6, 138.0, 130.0, 125.2, 124.4, 121.7, 118.5, 114.5, 29.6, 22.8, 13.3; IR (KBr) 3258, 1638, 1597 cm^{-1}; HRMS (DART) *m/z*: $[M+H]^+$ calcd for $C_{13}H_{15}N_2O_2$ 231.1134, found 231.1122.

7. Mechanistic Studies

7-1. H/D Exchange Experiments

*H/D exchange experiment with **1a** in the presence of CD3OD at 110 °C.*

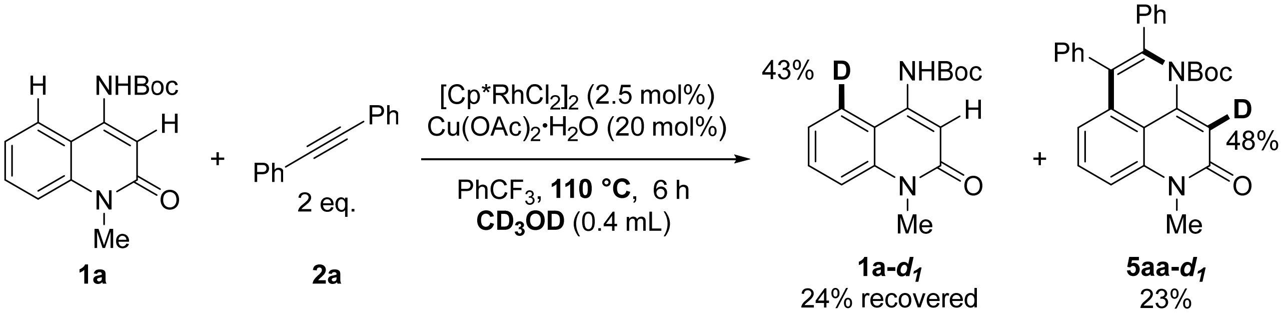

A Schlenk tube was charged with $[Cp^*RhCl_2]_2$ (3.0 mg, 0.0050 mmol, 2.5 mol%), $Cu(OAc)_2{\cdot}H_2O$ (8.0 mg, 0.040 mmol, 20 mol%), **1a** (54.7 mg, 0.20 mmol), and **2a** (71.5 mg, 0.40 mmol) . $PhCF_3$ (2.0 mL) and CD_3OD (0.40 mL) were added, and the mixture was stirred at 110 °C for 6 h under air atmosphere using an oil bath. Then, the mixture was cooled to room temperature and filtered through a pad of Celite® with $CHCl_3$ to remove the metal salts. After evaporation of the solvents under reduced pressure, the residue was purified by a silica gel column chromatography (Hexane/EtOAc = 1:1) to afford a mixture of **1a-*d*$_2$** and **5aa-*d*$_1$**. This mixture was further purified by preparative HPLC to give pure **1a-*d*$_2$** (24%) and **5aa-*d*$_1$** (23%). ^{1}H NMR analysis (400 MHz, CD_3OD or $CDCl_3$) showed that only 43% deuterium incorporation at the C5 position was observed in **1a**. In **5aa**, 48% deuterium incorporation at the C3 position was observed.

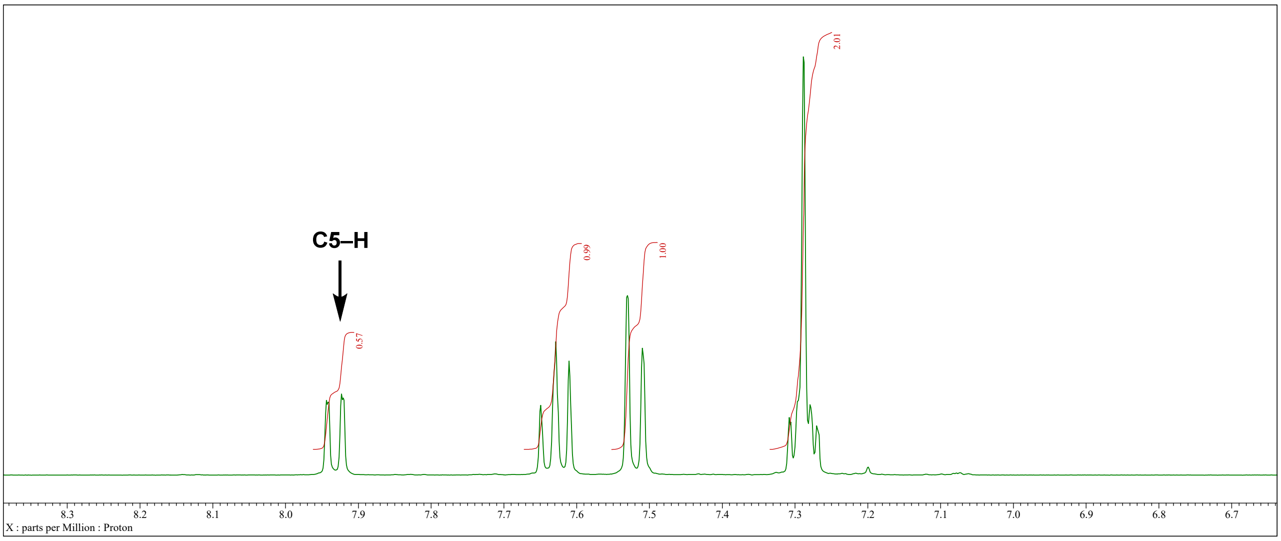

Figure S1. ^{1}H NMR spectrum (CD_3OD) of **1a** after the H/D exchange experiment.

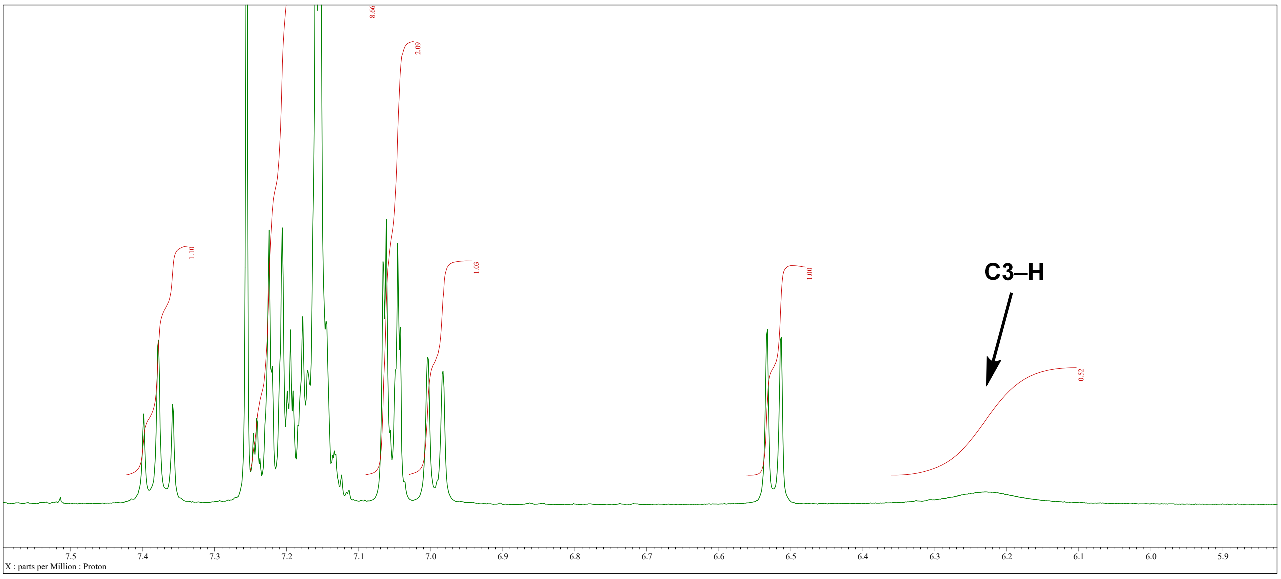

Figure S2. ^{1}H NMR spectrum ($CDCl_3$) of **5aa** after the H/D exchange experiment.

*H/D exchange experiment with **1b** in the presence of D_2O or AcOD at 80 °C.*

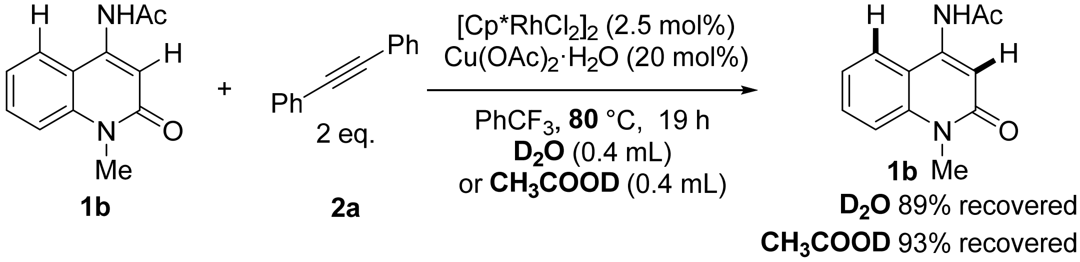

A Schlenk tube was charged with $[Cp^*RhCl_2]_2$ (3.1 mg, 0.0050 mmol, 2.5 mol%), $Cu(OAc)_2{\cdot}H_2O$ (8.0 mg, 0.040 mmol, 20 mol%), **1b** (43.2 mg, 0.20 mmol), and **2a** (71.3 mg, 0.40 mmol). $PhCF_3$ (2.0 mL) and D_2O (or AcOD) (0.40 mL) were added, and the mixture was stirred at 80 °C for 19 h under air atmosphere using an oil bath. Then, the mixture was cooled to room temperature and filtered through a pad of Celite® with $CHCl_3$ to remove the metal salts. After evaporation of the solvents under reduced pressure, the residue was purified by silica gel column chromatography (EtOAc only) to recover **1b** in 89% (or 93%) yield. ^{1}H NMR analysis (400 MHz, CD_3OD) showed that the deuterium incorporation at the C3- and C5 positions was not observed.

*H/D exchange experiment with **1b** in the presence of CD_3OD at 110 °C.*

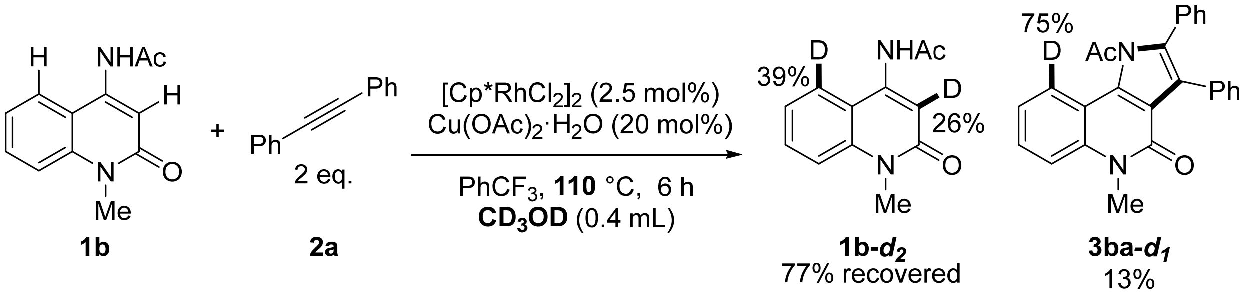

A Schlenk tube was charged with $[Cp^*RhCl_2]_2$ (3.1 mg, 0.0050 mmol, 2.5 mol%), $Cu(OAc)_2{\cdot}H_2O$ (8.0 mg, 0.040 mmol, 20 mol%), **1b** (43.2 mg, 0.20 mmol), and **2a** (71.3 mg, 0.40 mmol). $PhCF_3$ (2.0 mL) and CD_3OD (0.40 mL) were added, and the mixture was stirred at 110 °C for 6 h under air atmosphere using an oil bath. Then, the mixture was cooled to room temperature and filtered through a pad of Celite® with $CHCl_3$ to remove the metal salts. After evaporation of the solvents under reduced pressure, the residue was purified by a silica gel column chromatography (Hexane/EtOAc = 3:1 to EtOAc only) to afford **1b-*d*$_2$** in 77% yield and **3ba-*d*$_1$** in 13% yield. ^1H NMR analysis (400 MHz, CD_3OD) showed that 26% deuterium incorporation at the C3 position and 39% deuterium incorporation at the C5 position were observed in **1b**. In **3ba**, 75% deuterium incorporation at the C5 position was observed.

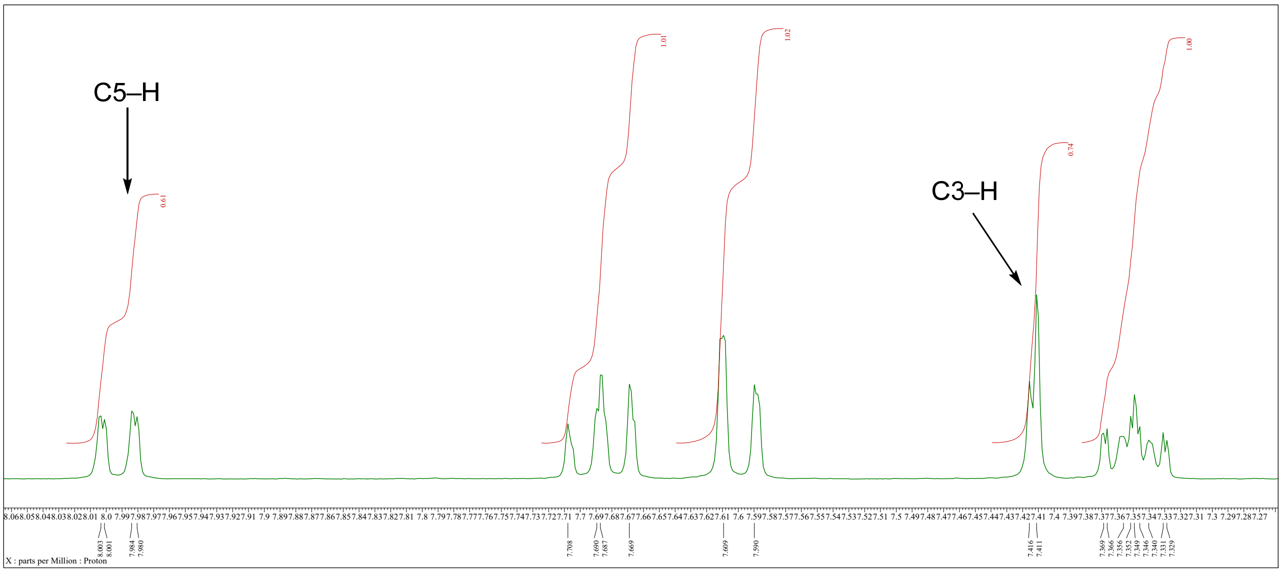

Figure S3. ^1H NMR spectrum of **1b** after the H/D exchange experiment.

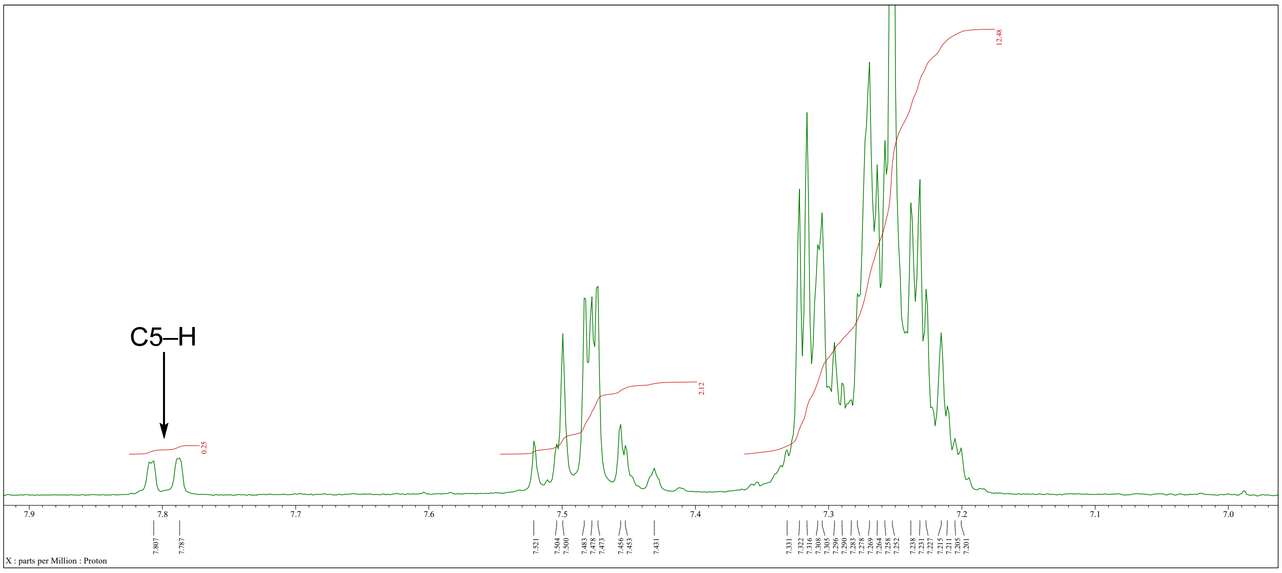

Figure S4. ^1H NMR spectrum of **3ba** after the H/D exchange experiment.

*H/D exchange experiment with **1a** in the presence of CD_3OD at 80 °C.*

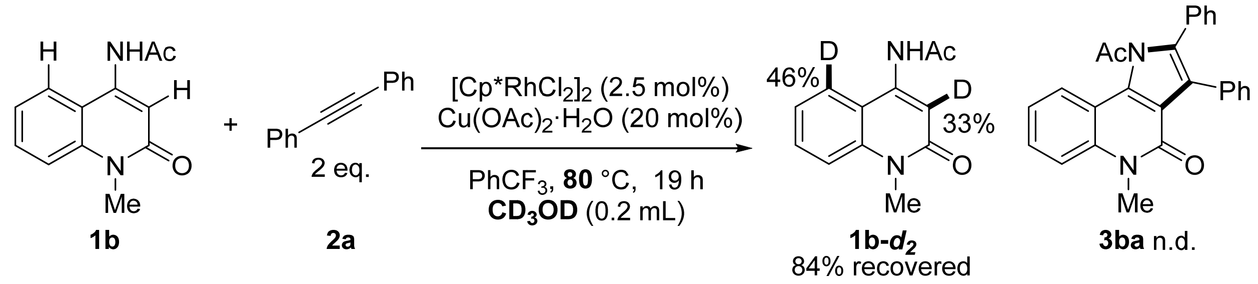

A Schlenk tube was charged with $[Cp^*RhCl_2]_2$ (1.5 mg, 0.0025 mmol, 2.5 mol%), $Cu(OAc)_2{\cdot}H_2O$ (4.0 mg, 0.020 mmol, 20 mol%), **1b** (21.3 mg, 0.10 mmol), and **2a** (36.4 mg, 0.20 mmol). $PhCF_3$ (1.0 mL) and CD_3OD (0.20 mL) were added, and the mixture was stirred at 80 °C for 19 h under air atmosphere using an oil bath. Then, the mixture was cooled to room temperature and filtered through a pad of Celite® with $CHCl_3$ to remove the metal salts. After evaporation of the solvents under reduced pressure, the residue was purified by silica gel column chromatography (EtOAc only) to afford **1b-*d*$_2$** in 84% yield. ^1H NMR analysis (400 MHz, CD_3OD)showed that the deuterium incorporation at both the C3 position (33%) and the C5 position (46%) were observed.

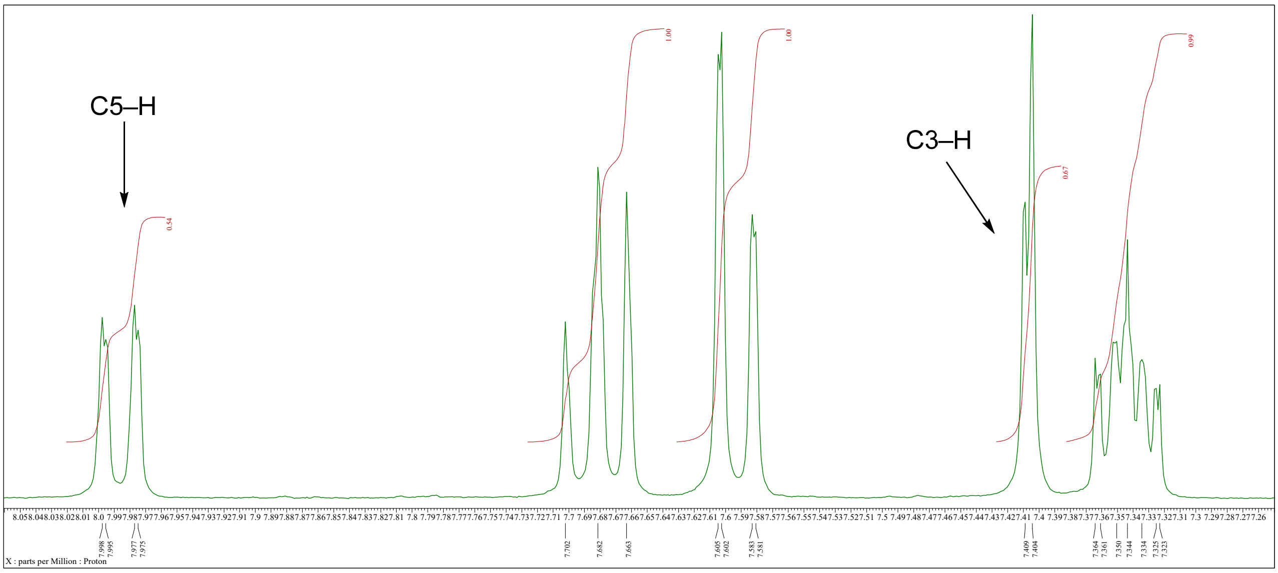

Figure S5. ^1H NMR spectrum of **1b** after the H/D exchange experiment.

7-2. ^{1}H NMR Monitoring of the Reaction

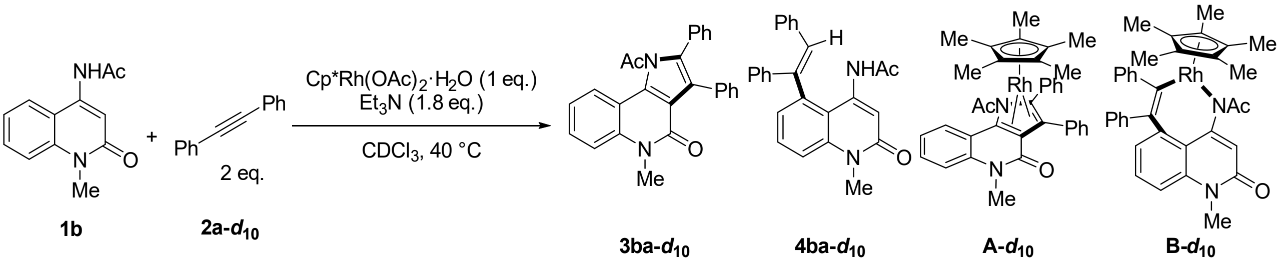

A J.Young. NMR tube was charged with Cp*Rh(OAc)$_2$·H$_2$O (3.8 mg, 0.010 mmol), **1b** (2.1 mg, 0.010 mmol), **2a** (3.7 mg, 0.020 mmol), Et$_3$N (2.5 μL (1.8 mg), 0.18 mmol), and CDCl$_3$ (0.50 mL) under Ar atmosphere. The reaction mixture was stirred at 40 °C using an oil bath and the reaction was monitored by ^{1}H NMR.

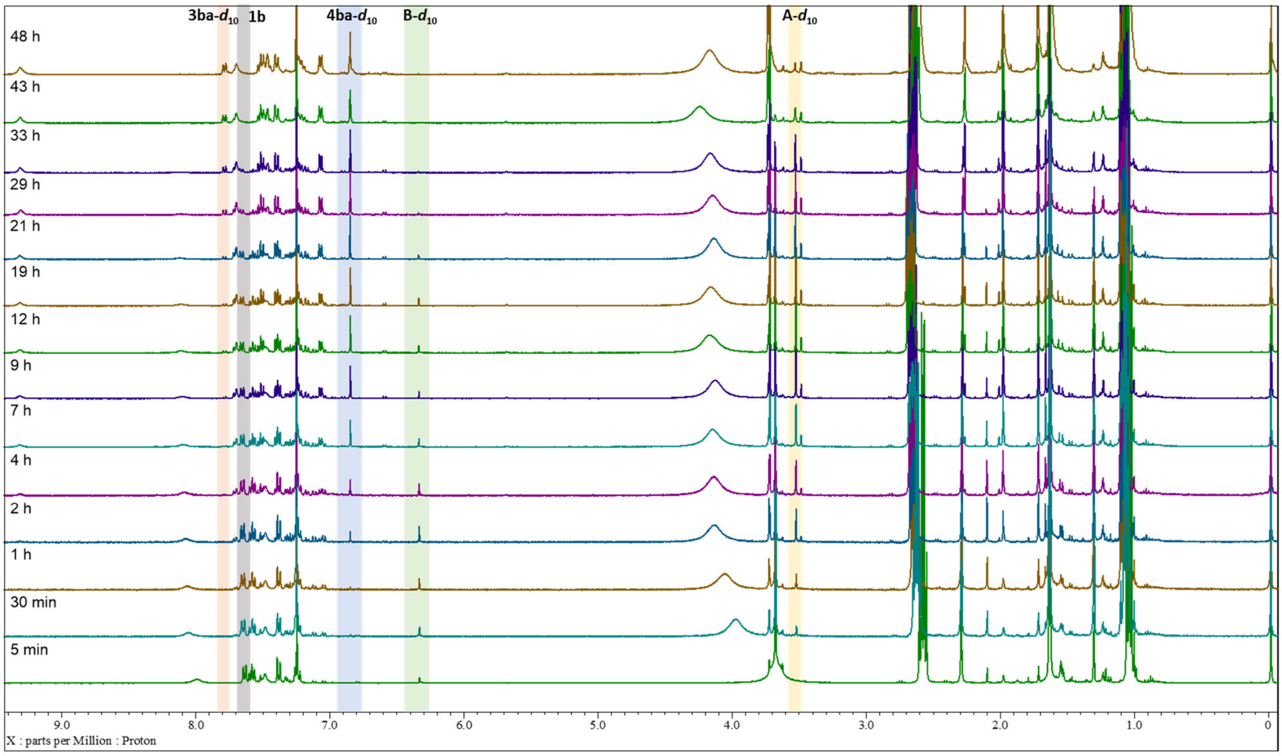

Figure S6. Monitoring of the reaction.

7-3. Isolation of Complex A

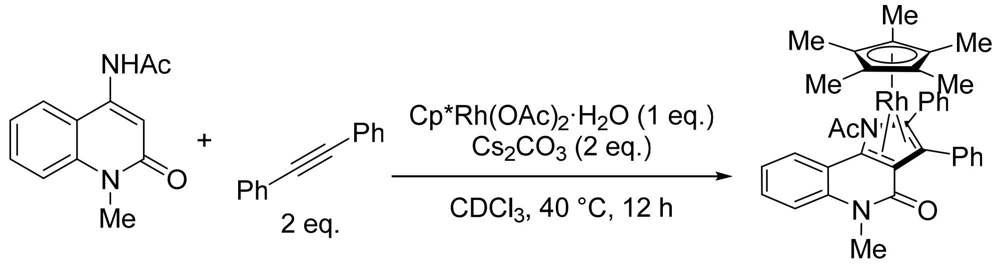

1b **2a** **A**

A J. Young. NMR tube was charged with Cp*Rh(OAc)$_2$·H$_2$O (11.2 mg, 0.030 mol), **1b** (6.5 mg, 0.030 mmol), **2a** (10.7 mg, 0.060 mmol), Cs$_2$CO$_3$ (19.5 mg, 0.060 mmol), and CDCl$_3$ (0.50 mL) under Ar atmosphere. The reaction mixture was stirred at 40 °C using an oil bath and the reaction was monitored by ^{1}H NMR. After 12 h, CDCl$_3$ was removed under reduced pressure. The residue was purified by silica gel column chromatography (Hexane/EtOAc = 1:1). Red crystals were obtained by recrystallization from CHCl$_3$ and hexane. The structure of complex **A** was determined by X-Ray diffraction analysis. Other than that, only ^{1}H NMR analysis was conducted using a mixture of **A** and **3ba** because **A** was immediately converted to **3ba** in CDCl$_3$.

^{1}H NMR (400 MHz, CDCl$_3$) δ 7.77-7.65 (m, 3H), 7.53-7.37 (m, 3H), 7.35-7.28 (m, 3H), 7.20 (d, *J* = 7.8 Hz, 1H), 7.17-7.10 (m, 2H), 7.06-6.95 (m, 2H), 3.55 (s, 3H), 1.68 (s, 3H), 1.13 (s, 15H).

7-4. Preparation and Characterization of Complex B-Me

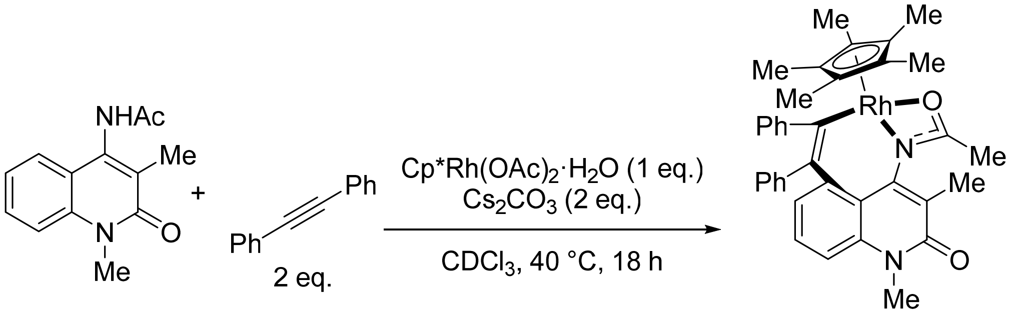

1h **2a** **B-Me** 66%

A Schlenk tube was charged with Cp*Rh(OAc)$_2$·H$_2$O (11.1 mg, 0.030 mmol), **1h** (6.5 mg, 0.030 mmol), **2a** (10.7 mg, 0.060 mmol), Cs$_2$CO$_3$ (19.5 mg, 0.060 mmol), and CDCl$_3$ (0.50 mL) under Ar atmosphere. The reaction mixture was stirred at 40 °C using an oil bath for 12 h. The solvent was removed under reduced pressure. This crude product was treated with DCM and hexane at room temperature to form a reddish-orange solid, which was collected by filtration (12.7 mg, 66%). The obtained solid was recrystallized from diethyl ether/hexane. **Analytical data for B-Me:** ^{1}H NMR (400 MHz, CDCl$_3$) δ 7.31-7.26 (m, 1H), 7.14 (dd, *J* = 7.6, 6.2 Hz, 2H), 7.07-6.98 (m, 4H), 6.91-6.85 (m, 3H), 6.82-6.77(m, 1H), 6.74-6.70 (m, 2H), 3.80 (s, 3H), 2.28 (s, 3H), 1.89 (s, 3H), 1.29 (s, 15H); ^{13}C NMR data could not be obtained due to the instability of the complex over the timescale required for the experiment; HRMS (ESI) *m/z*: [M+Na]$^{+}$ calcd for $C_{37}H_{37}N_2NaO_2Rh$ 667.1808, found 667.1817.

Comparison of ^{1}H NMR spectra of **B** and **B-Me**.

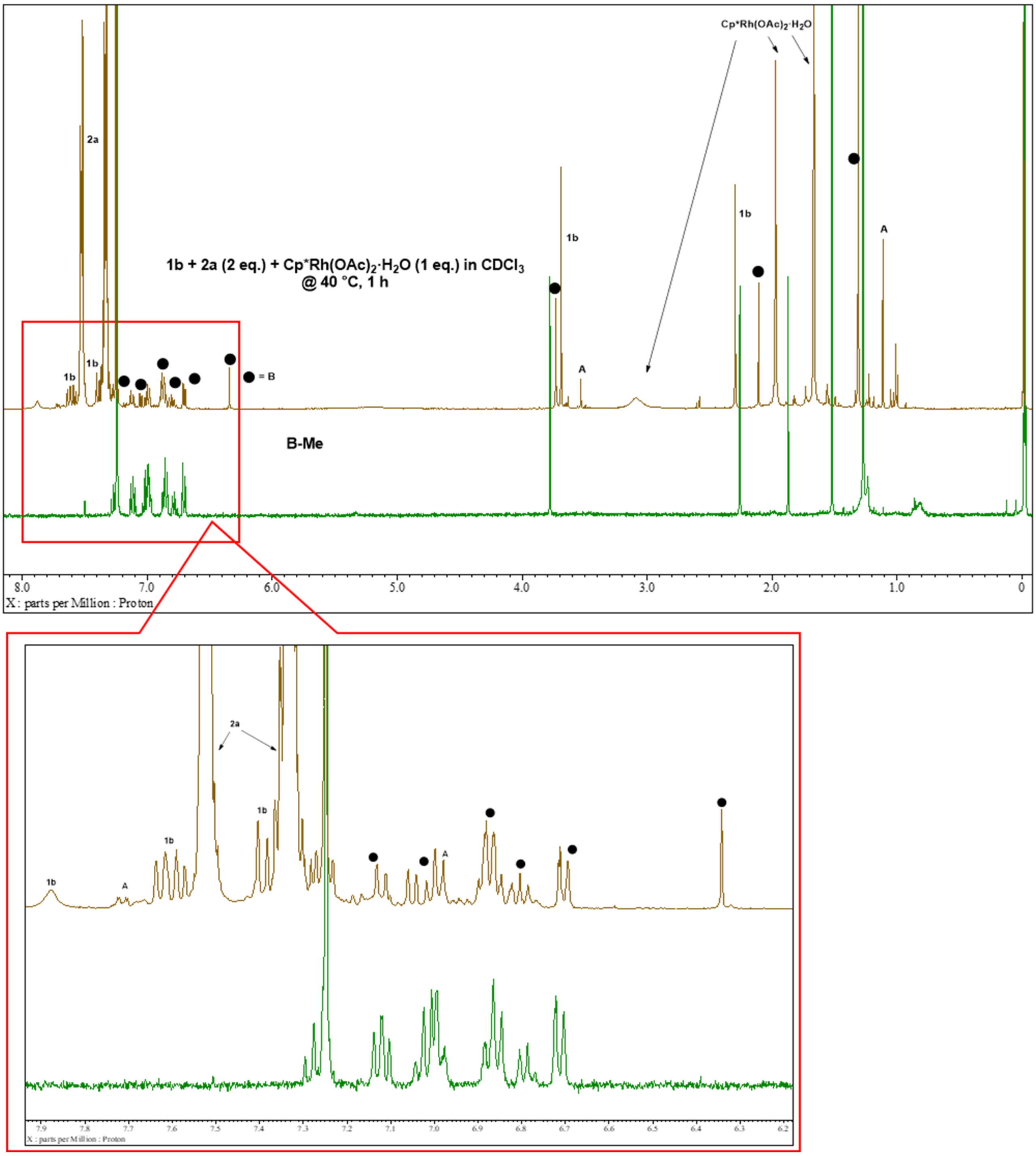

Figure S7. ^{1}H NMR spectra of **B** (reaction mixture) and **B-Me** (purified).

7-5. Catalytic Reaction with 1h

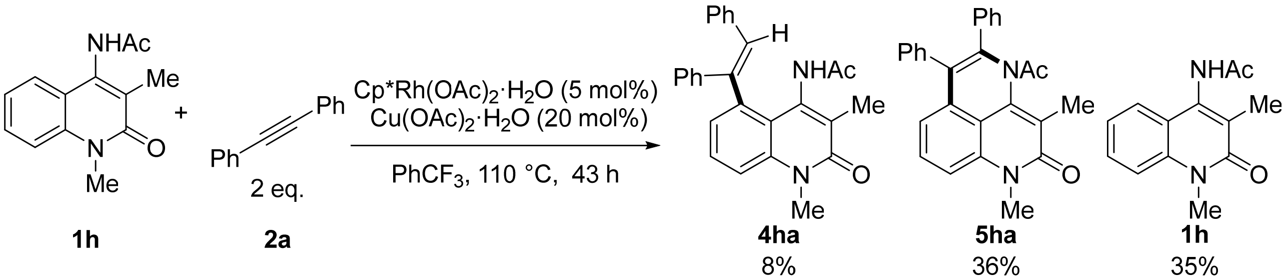

A Schlenk tube was charged with $Cp^*Rh(OAc)_2 \cdot H_2O$ (3.7 mg, 0.010 mmol, 5 mol%), $Cu(OAc)_2 \cdot H_2O$ (7.9 mg, 0.040 mmol, 20 mol%), **1h** (46.1 mg, 0.20 mmol), **2a** (71.1 mg, 0.40 mmol), and $PhCF_3$ (2.0 mL). The reaction mixture was stirred at 110 °C for 43 h under air atmosphere using an oil bath. Then, the mixture was cooled to room temperature and filtered through a pad of Celite® with $CHCl_3$ to remove the metal salts. After concentration in vacuo, the residue was purified by silica gel column chromatography (Hexane/EtOAc = 2:1 to 1:1 to EtOAc only).

7-6. Experiments with B-Me

Table S3. Screening some additives to promote the *β*-carbon elimination.

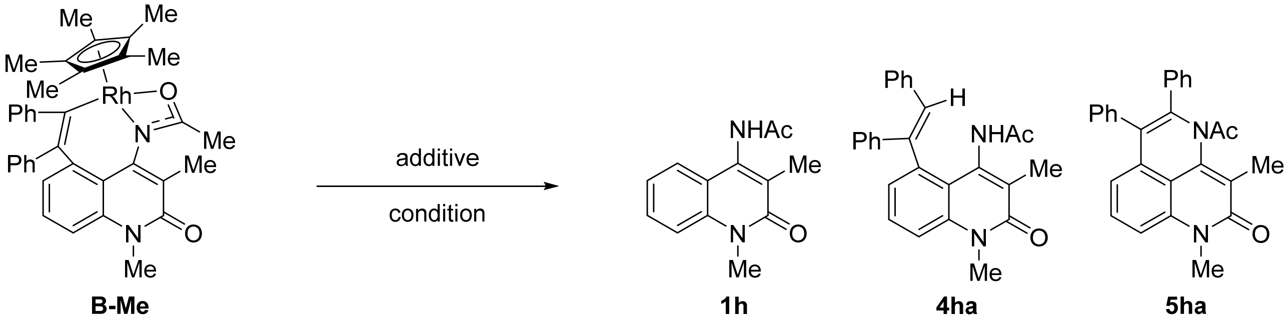

additive	condition	**1h**	**4ha**	**5ha**
-	$PhCF_3$, 110 °C, 13 h	n.d.	n.d.	major
AcOH-d_4 (1 eq.)	$CDCl_3$, 60 °C, 3 h	n.d.	major	n.d.
MeOH-d_4 (1 eq.)	$CDCl_3$, 60 °C, 4 days	n.d.	trace	major
**1b** (10 eq.)	$CDCl_3$, 60 °C, 4 days	n.d.	major (1 : 1)	
$Cu(OAc)_2 \cdot H_2O$ (1 eq.)	$PhCF_3$, 110 °C, 13 h	n.d.	n.d.	major
NaOAc (1 eq.)	$PhCF_3$, 110 °C, 13 h	n.d.	n.d.	major
**3ba** (1 eq.)	$PhCF_3$, 110 °C, 13 h	n.d.	n.d.	major
$Cp^*Rh(OAc)_2 \cdot H_2O$ (1 eq.)	$PhCF_3$, 110 °C, 13 h	major (1 : 1.2 : 1.2)		

*Heating **B-Me** at 110 °C.*

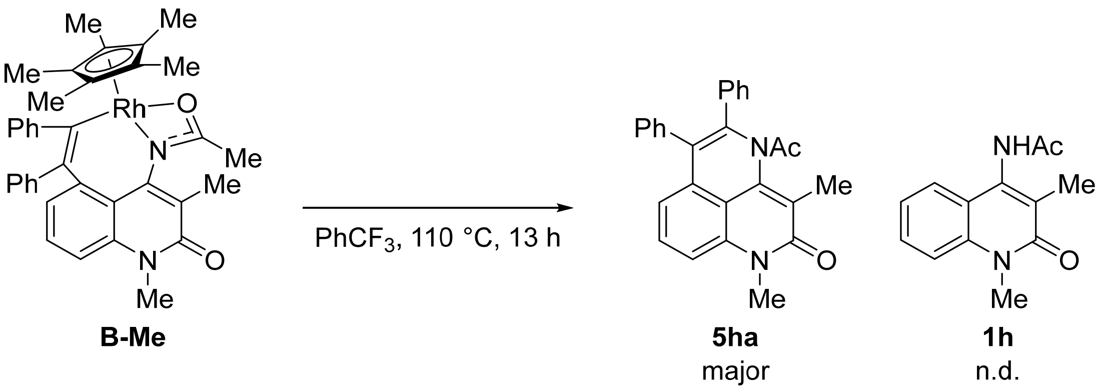

A Schlenk tube was charged with **B-Me** (0.6 mg, 0.0010 mmol) and $PhCF_3$ (1.0 mL) under Ar atmosphere. The reaction mixture was stirred at 110 °C for 13 h under Ar atmosphere using an oil bath. The resulting solution was concentrated. ^{1}H NMR analysis of the crude product mixture showed that **5ha** was mainly observed while **1h** was not detected.

Addition of acetic acid (1 eq.) at 60 °C.

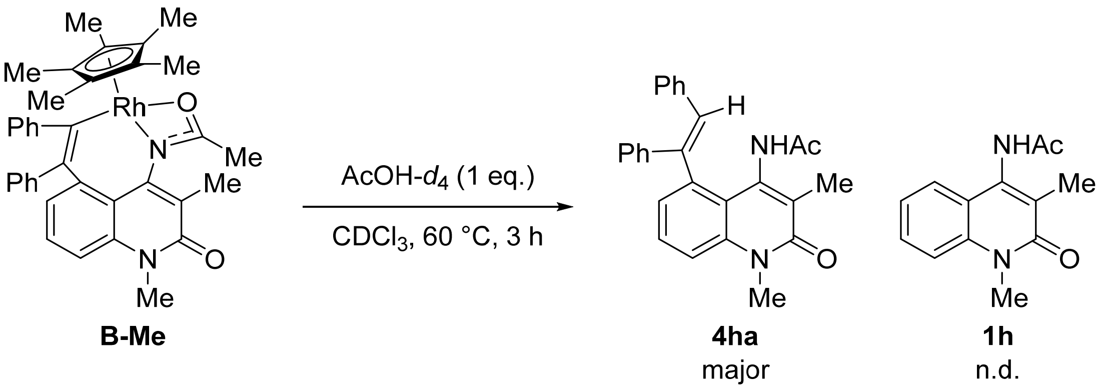

A crimp vial was loaded with AcOH-d_4 (6.4 mg, 0.010 mmol) and dissolved in 5 mL of $CDCl_3$ to obtain a 0.002 M AcOH-d_4 solution in $CDCl_3$. A J. Young NMR tube was charged with **B-Me** (0.6 mg, 0.0010 mmol) and the prepared solution (0.50 mL) under Ar atmosphere. The reaction mixture was stirred at 60 °C using an oil bath and the reaction was monitored by ^{1}H NMR. After 3h at this temperature, **B-Me** was completely consumed and **4ha** was mainly observed while **1h** was not detected by ^{1}H NMR.

Addition of methanol (1 eq.) as a proton source at 60 °C.

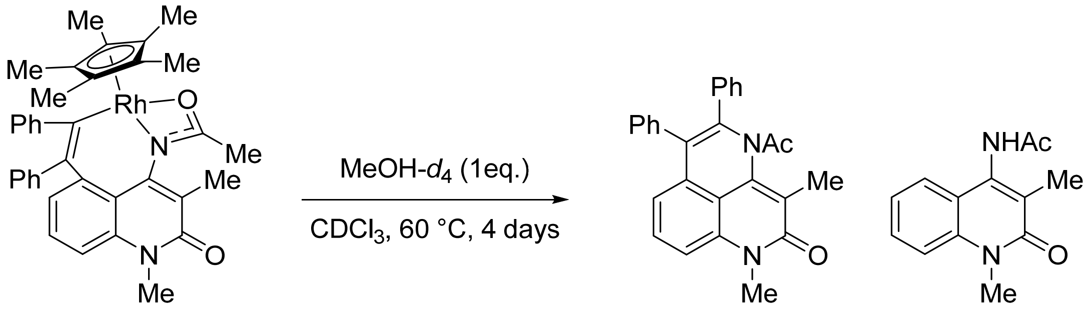

B-Me **5ha** major **1h** n.d.

A crimp vial was loaded with MeOH-d_4 (3.6 mg, 0.010 mmol) and dissolved in 5 mL of $CDCl_3$ to obtain a 0.002 M MeOH-d_4 solution in $CDCl_3$. A J. Young NMR tube was charged with **B-Me** (0.6 mg, 0.0010 mmol) and the prepared solution (0.50 mL) under Ar atmosphere. The reaction mixture was stirred at 60 °C using an oil bath and the reaction was monitored by ^{1}H NMR. After 4 days at this temperature, **B-Me** was completely consumed and **5ha** was mainly observed while **1h** was not detected by ^{1}H NMR.

*Addition of **1b** (10 eq.) at 60 °C.*

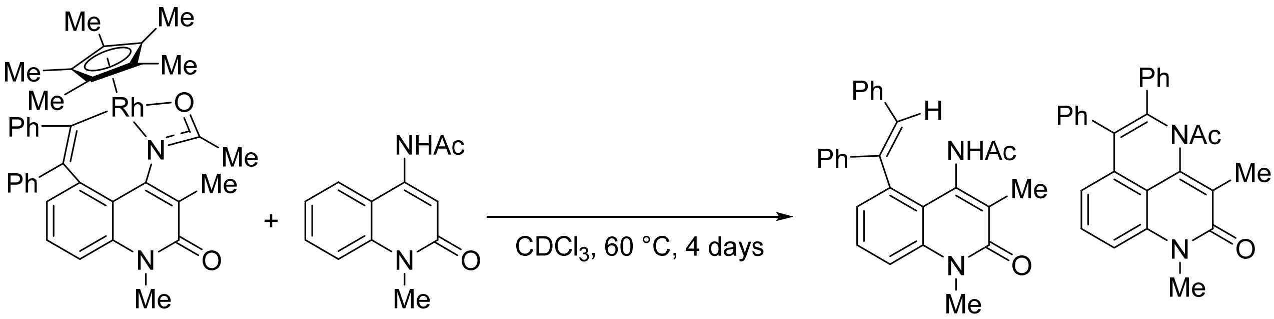

B-Me **1b** 10 eq. **4ha** **5ha** (1 : 1)

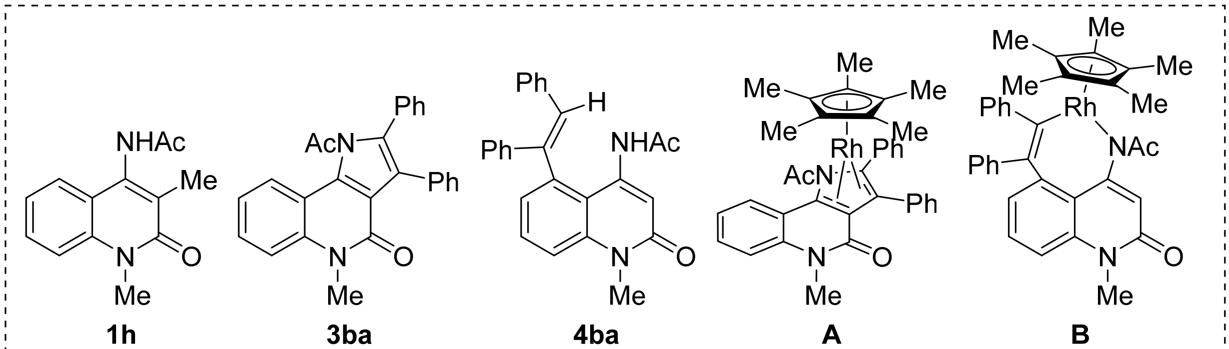

1h **3ba** **4ba** **A** **B** **n.d.**

A J. Young NMR tube was charged with **B-Me** (0.6 mg, 0.0010 mmol), **1b** (2.2 mg, 0.010 mmol), $CDCl_3$ (0.5 mL) under Ar atmosphere. The reaction mixture was stirred at 60 °C using an oil bath and the reaction was monitored by ^{1}H NMR. After 4 days at this temperature, **4ha** and **5ha** were observed as the major products, while other products such as **1h**, **3ba**, **4ba**, **A**, and **B** were not detected in this experiment.

*Heating **B-Me** with Cu(OAc)$_2$·H$_2$O (1 eq) at 110 °C.*

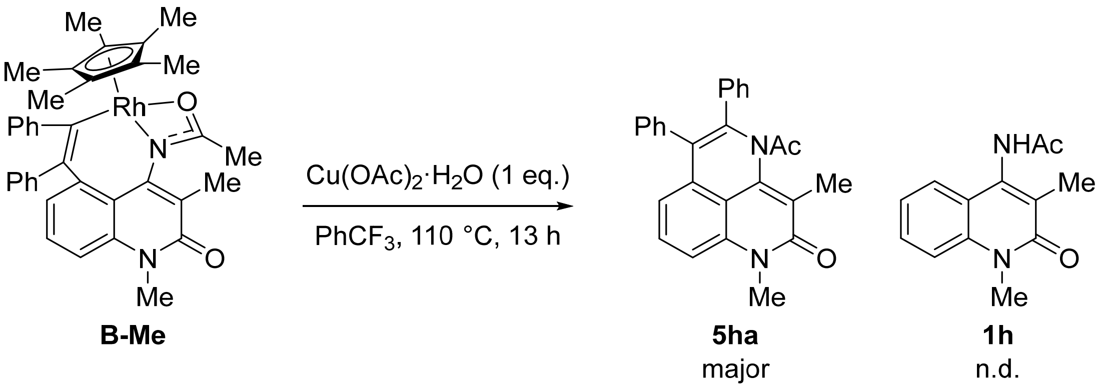

A Schlenk tube was charged with **B-Me** (0.6 mg, 0.0010 mmol), Cu(OAc)$_2$·H$_2$O (0.2 mg, 0.0010 mmol) and PhCF$_3$ (1.0 mL) under Ar atmosphere. The reaction mixture was stirred at 110 °C for 13 h under Ar atmosphere using an oil bath. The resulting solution was concentrated, and the ^{1}H NMR analysis of the crude product mixture showed that **5ha** was mainly observed while **1h** was not detected.

*Heating **B-Me** with NaOAc (1 eq) at 110 °C.*

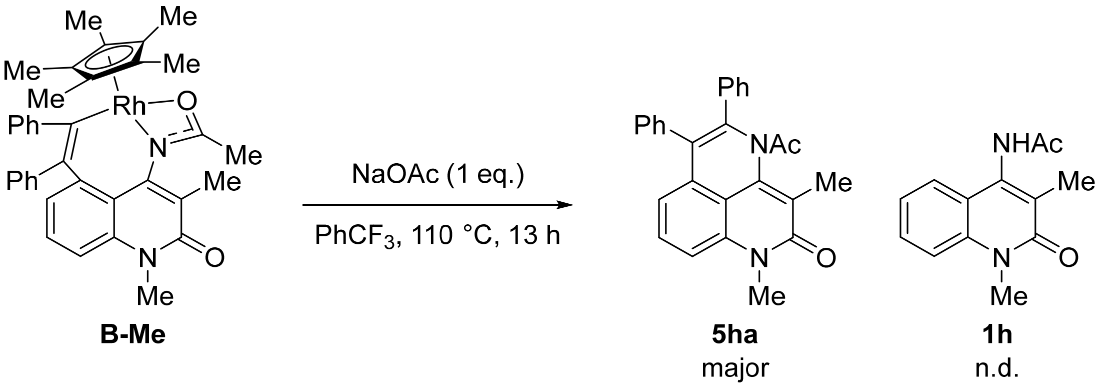

A Schlenk tube was charged with **B-Me** (0.6 mg, 0.0010 mmol), NaOAc (0.08 mg, 0.0010 mmol) and PhCF$_3$ (1.0 mL) under Ar atmosphere. The reaction mixture was stirred at 110 °C for 13 h under Ar atmosphere using an oil bath. The resulting solution was concentrated, and the ^{1}H NMR analysis of the crude product mixture showed that **5ha** was mainly observed while **1h** was not detected.

*Heating **B-Me** with **3ba** (1 eq.) at 110 °C.*

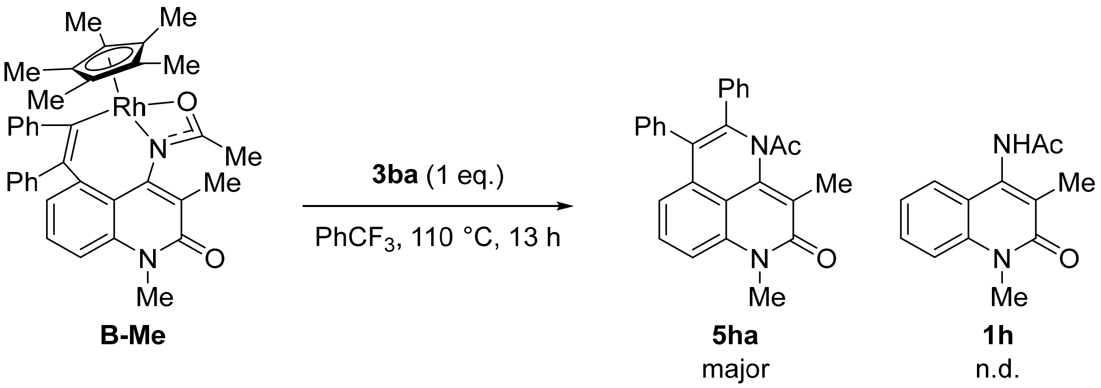

A Schlenk tube was charged with **B-Me** (0.6 mg, 0.0010 mmol), **3ba** (0.4 mg, 0.0010 mmol) and $PhCF_3$ (1.0 mL) under Ar atmosphere. The reaction mixture was stirred at 110 °C for 6 h under Ar atmosphere using an oil bath. The resulting solution was concentrated, and the ^{1}H NMR analysis of the crude product mixture showed that **5ha** was mainly observed while **1h** was not detected.

*Heating **B-Me** with Cp*Rh(OAc)$_2$·H$_2$O (1 eq.) at 110 °C.*

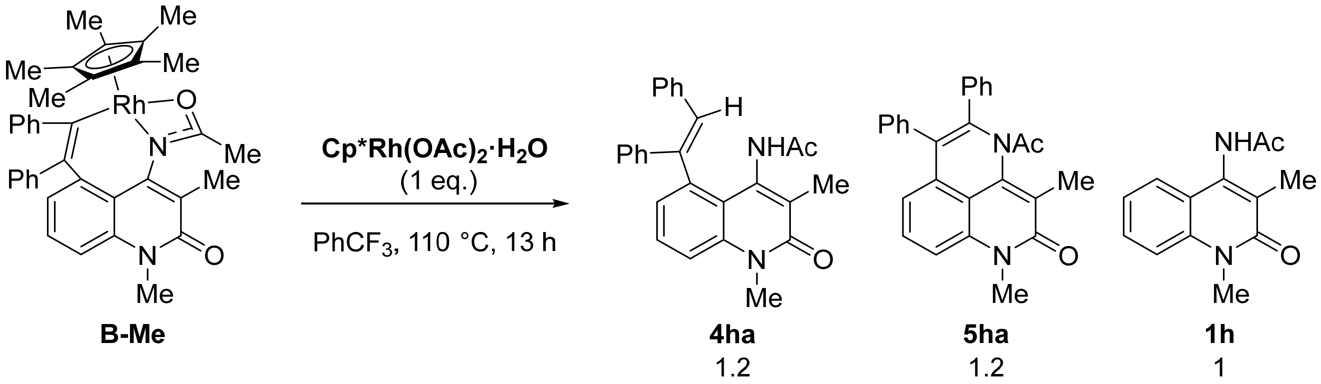

A Schlenk tube was charged with **B-Me** (0.6 mg, 0.0010 mmol), Rh(OAc)$_2$·H$_2$O (0.4 mg, 0.0010 mmol) and $PhCF_3$ (1.0 mL) under Ar atmosphere. The reaction mixture was stirred at 110 °C for 13 h under Ar atmosphere using an oil bath. The resulting solution was concentrated, and the ^{1}H NMR analysis of the crude product mixture showed that **4ha**, **5ha**, and **1h** were obtained (**4ha**/**5ha**/**1h** = 1.2:1.2:1).

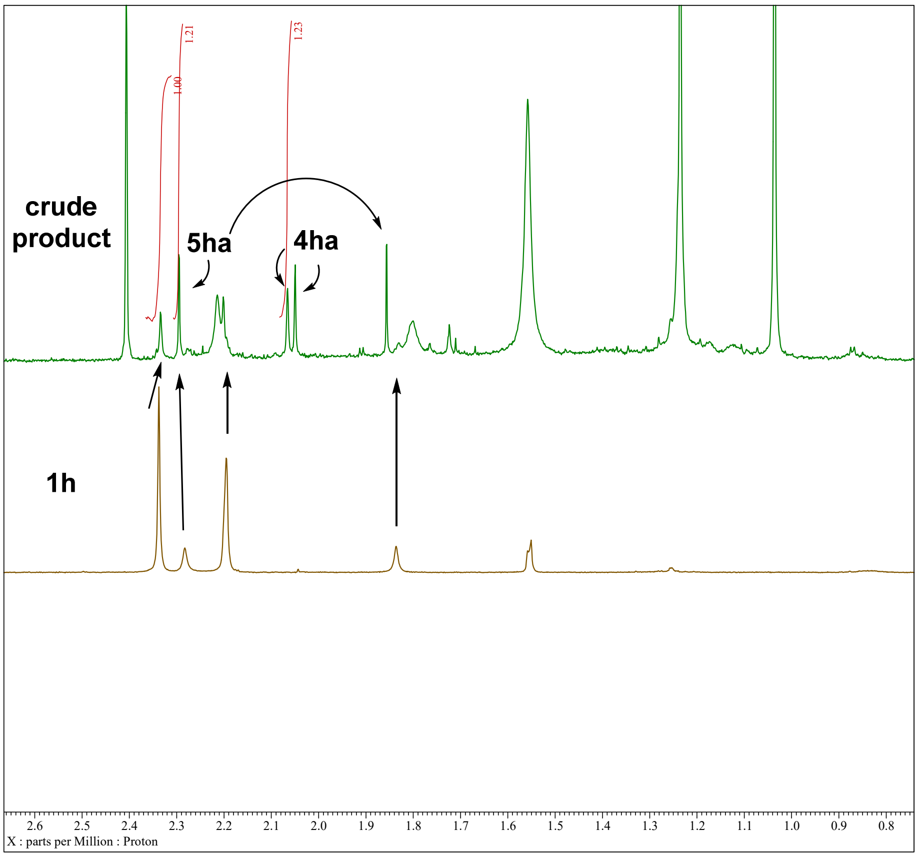

Figure S8. ^{1}H NMR spectra of the crude product.

*Isolated of **1h** from the reaction of **B-Me** with Cp*Rh(OAc)$_2$·H$_2$O.*

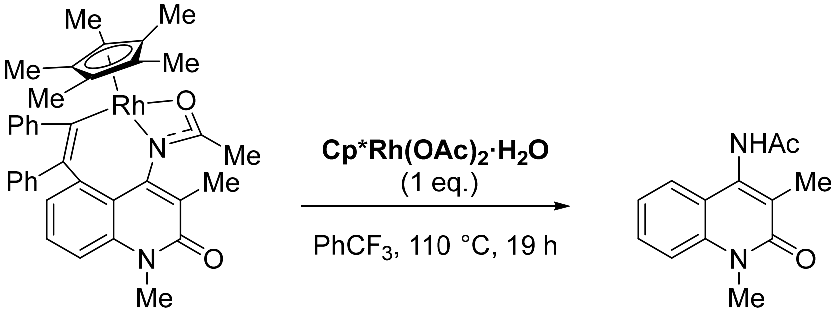

A Schlenk tube was charged with **B-Me** (19.3 mg, 0.030 mmol), Rh(OAc)$_2$·H$_2$O (11.2 mg, 0.030 mmol) and PhCF$_3$ (30 mL) under Ar atmosphere. The reaction mixture was stirred at 110 °C for 19 h under Ar atmosphere using an oil bath. The resulting solution was concentrated and the ^{1}H NMR analysis of the crude product mixture showed that **4ha**, **5ha**, and **1h** were obtained (**4ha**/**5ha**/**1h** =29%:38%:19%). The crude product purified by preparative TLC (Hexane/EtOAc = 1:1 to EtOAc only) to give **1h** (0.50 mg, 14%).

8. X-Ray Diffraction Analysis

X-Ray Diffraction Analysis of 3ba

Diffraction data were collected in θ ranges specified in Table S4 at 123 K on a Rigaku R-AXIS Rapid diffractometer with graphite monochromatized Cu-Ka radiation (λ = 1.54187 Å). The Lorenz polarization absorption correction was applied. The structure was solved by direct methods and refined by the full-matrix least-squares on F^2. All non-hydrogen atoms were refined with anisotropic displacement parameters. All hydrogen atoms were refined using the riding model. Final refinement details are compiled in Table S4. The supplementary crystallographic data for this paper (CCDC2278040) can also be obtained free of charge via www.ccdc.cam.ac.uk/conts/retrieving.html (or from the Cambridge Crystallographic Data Centre, 12, Union Road, Cambridge CB2 1EZ, UK; fax: +44 1223 336033; or deposit@ccdc.cam.ac.uk).

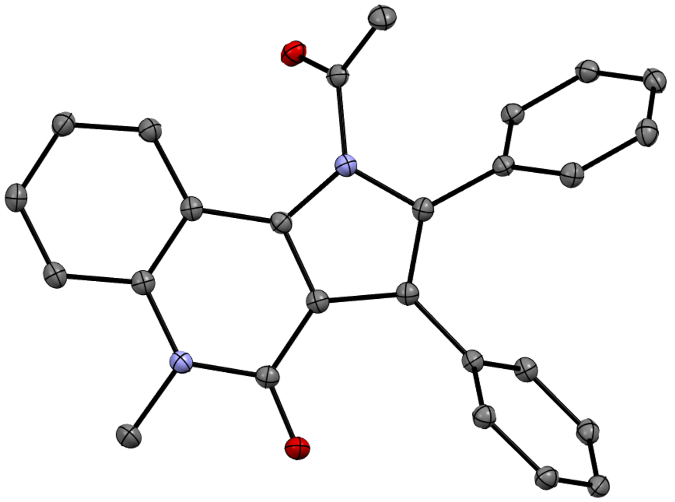

Figure S9. ORTEP plot of 3ba

Table S4. Selected crystallographic data and collection parameters for **3ba**.

formula	$C_{26}H_{20}N_2O_2$	crystal size, mm	0.2 x 0.15 x 0.1
FW	392.46	maximum 2θ, deg	136.3
crystal system	monoclinic	reflections collected	22156
space group	P21/n (#14)	independent reflections [*R*(int)]	3536 [R(int) = 0.0272]
a, Å	10.3174(4)	max. and min. transmission	0.935/0.699
b, Å	18.8450(7)	goodness-of-fit on F^2	1.044
c, Å	10.3316(4)	R_1 [$I > 2\sigma(I)$]	0.0406
volume, Å^3	1956.84(14)	*R*, wR_2 (all data)	0.0433, 0.1085
β, °	103.056(7)	Weighting scheme	$R_1 = \Sigma \|\|Fo\| - \|Fc\|\| / \Sigma \|Fo\|$
Z	4		$wR_2 = [\Sigma(w(Fo^2 - Fc^2)^2)/\Sigma w(Fo^2)^2]^{1/2}$
D (calcd), Mg m^{-3}	1.332	largest diff. peak and hole, e Å^{-3}	0.19 and –0.26
μ, cm^{-1}	6.763		
F(000)	824.00		

X-Ray Diffraction Analysis of 3bf'·$CHCl_3$

Diffraction data were collected in θ ranges specified in Table S5 at 123 K on a Rigaku R-AXIS Rapid diffractometer with graphite monochromatized Cu-Ka radiation (λ = 1.54187 Å). The Lorenz polarization absorption correction was applied. The structure was solved by direct methods and refined by the full-matrix least-squares on F^2. All non-hydrogen atoms were refined with anisotropic displacement parameters. All hydrogen atoms were refined using the riding model. Final refinement details are compiled in Table S5. The supplementary crystallographic data for this paper (CCDC2278041) can also be obtained free of charge via www.ccdc.cam.ac.uk/conts/retrieving.html (or from the Cambridge Crystallographic Data Centre, 12, Union Road, Cambridge CB2 1EZ, UK; fax: +44 1223 336033; or deposit@ccdc.cam.ac.uk).

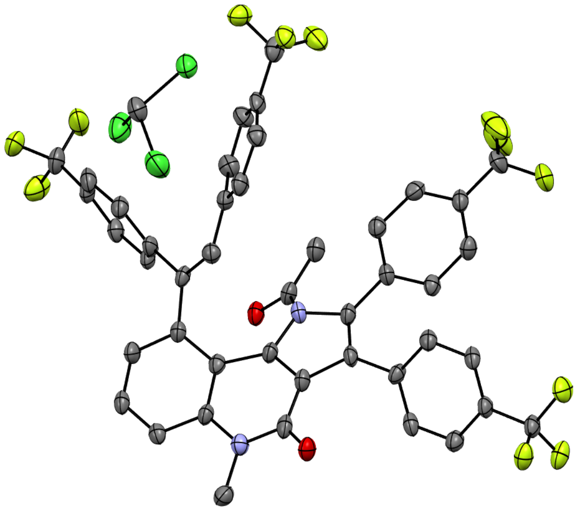

Figure S10. ORTEP plot of 3bf'·$CHCl_3$

Table S5. Selected crystallographic data and collection parameters for **3bf'·$CHCl_3$**.

formula	$C_{45}H_{27}Cl_3\text{-}F_{12}N_2O_2$	μ, cm^{-1}	29.459
FW	962.06	F(000)	972.00
crystal system	triclinic	crystal size, mm	0.3 x 0.1 x 0.05
		maximum 2θ, deg	136.4
space group	P-1 (#2)	reflections collected	23137
a, Å	10.1061(11)	independent reflections [R(int)]	7190 [R(int) = 0.1528]
b, Å	14.1090(14)	max. and min. transmission	0.863/0.329
c, Å	14.8443(15)	goodness-of-fit on F^2	1.072
volume, Å^3	2017.9(4)	R_1 [I >2σ(I)]	0.1199
α, °	85.453(6)	R, wR_2 (all data)	0.2328, 0.4302
β, °	83.081(6)	Weighting scheme	$R_1 = \Sigma$ ||Fo| - |Fc|| / Σ |Fo|
γ, °	74.047(5)		$wR_2 = [\Sigma(w(Fo^2 - Fc^2)^2)/\Sigma w(Fo^2)^2]^{1/2}$
Z	2	largest diff. peak and hole, e Å^{-3}	0.52 and –0.40
D (calcd), Mg m^{-3}	1.583		

X-Ray Diffraction Analysis of 4ba

Diffraction data were collected in θ ranges specified in Table S6 at 123 K on a Rigaku R-AXIS Rapid diffractometer with graphite monochromatized Cu-Ka radiation (λ = 1.54187 Å). The Lorenz polarization absorption correction was applied. The structure was solved by direct methods and refined by the full-matrix least-squares on F^2. All non-hydrogen atoms were refined with anisotropic displacement parameters. All hydrogen atoms were refined using the riding model. Final refinement details are compiled in Table S6. The supplementary crystallographic data for this paper (CCDC2278042) can also be obtained free of charge via www.ccdc.cam.ac.uk/conts/retrieving.html (or from the Cambridge Crystallographic Data Centre, 12, Union Road, Cambridge CB2 1EZ, UK; fax: +44 1223 336033; or deposit@ccdc.cam.ac.uk).

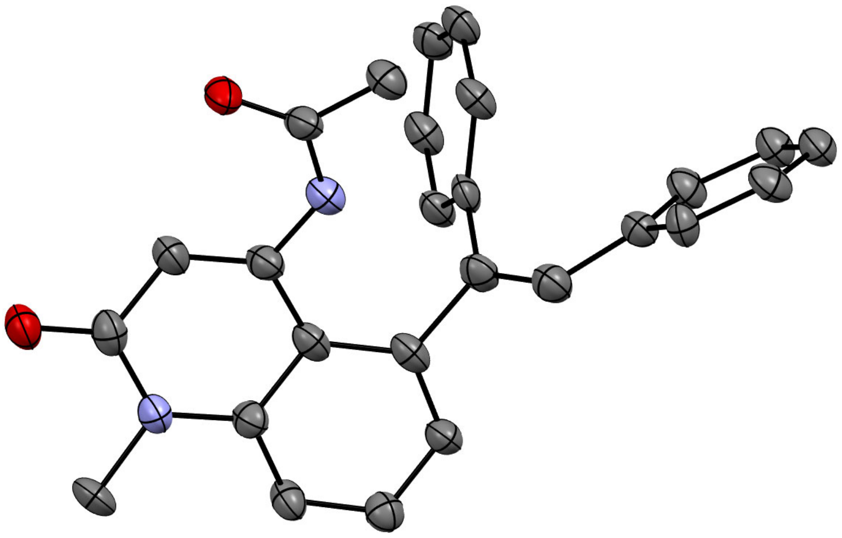

Figure S11. ORTEP plot of 4ba

Table S6. Selected crystallographic data and collection parameters for **4ba**.

formula	$C_{26}H_{22}N_2O_2$	crystal size, mm	0.1 x 0.1 x 0.1
FW	394.47	maximum 2θ, deg	136.3
crystal system	monoclinic	reflections collected	10978
space group	P21 (#4)	independent reflections [*R*(int)]	3580 [R(int) = 0.1214]
a, Å	11.2867(14)	max. and min. transmission	0.937/0.538
b, Å	7.6954(9)	goodness-of-fit on F^2	0.991
c, Å	11.8213(14)	R_1 [$I > 2\sigma(I)$]	0.0904
volume, Å^3	1014.9(2)	R, wR_2 (all data)	0.1934, 0.2730
β, °	98.725(7)	Weighting scheme	$R_1 = \Sigma \|\|Fo\| - \|Fc\|\| / \Sigma \|Fo\|$
Z	2		$wR_2 = [\Sigma(w(Fo^2 - Fc^2)^2)/\Sigma w(Fo^2)^2]^{1/2}$
D (calcd), Mg m^{-3}	1.291	largest diff. peak and hole, e Å^{-3}	0.18 and –0.18
μ, cm^{-1}	6.523		
F(000)	416.00		

X-Ray Diffraction Analysis of 5ha

Diffraction data were collected in θ ranges specified in Table S7 at 123 K on a Rigaku PILATUS200K diffractometer with graphite monochromatized MoKa radiation (λ =0.71075 Å). The Lorenz polarization absorption correction was applied. The structure was solved by direct methods and refined by the full-matrix least-squares on F^2. All non-hydrogen atoms were refined with anisotropic displacement parameters. All hydrogen atoms were refined using the riding model. Final refinement details are compiled in Table S7. The supplementary crystallographic data for this paper (CCDC2278043) can also be obtained free of charge via www.ccdc.cam.ac.uk/conts/retrieving.html (or from the Cambridge Crystallographic Data Centre, 12, Union Road, Cambridge CB2 1EZ, UK; fax: +44 1223 336033; or deposit@ccdc.cam.ac.uk).

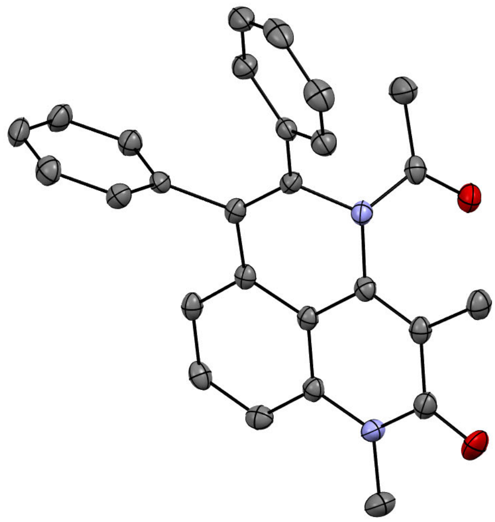

Figure S12. ORTEP plot of 5ha

Table S7. Selected crystallographic data and collection parameters for **5ha**.

formula	$C_{27}H_{22}N_2O_2$	crystal size, mm	0.15 x 0.1 x 0.075
FW	406.48	maximum 2θ, deg	55.0
crystal system	monoclinic	reflections collected	17132
space group	P21/n (#14)	independent reflections [R(int)]	4658 [R(int) = 0.1149]
a, Å	9.0396(15) Å	max. and min. transmission	0.994/0.569
b, Å	21.882(4) Å	goodness-of-fit on F^2	0.824
c, Å	10.4133(18) Å	R_1 [$I > 2\sigma(I)$]	0.0524
volume, $Å^3$	2043.5(6)	R, wR_2 (all data)	0.0706, 0.1152
β, °	97.221(4)	Weighting scheme	$R_1 = \Sigma \|\|Fo\| - \|Fc\|\| / \Sigma \|Fo\|$
Z	4		$wR_2 = [\Sigma(w(Fo^2 - Fc^2)^2)/\Sigma w(Fo^2)^2]^{1/2}$
D (calcd), Mg m^{-3}	1.321	largest diff. peak and hole, e $Å^{-3}$	0.26 and –0.27
μ, cm^{-1}	0.838		
F(000)	856.00		

X-Ray Diffraction Analysis of A

Diffraction data were collected in θ ranges specified in Table S8 at 123 K on a Rigaku R-AXIS Rapid diffractometer with graphite monochromatized Cu-Ka radiation (λ = 1.54187 Å). The Lorenz polarization absorption correction was applied. The structure was solved by direct methods and refined by the full-matrix least-squares on F^2. All non-hydrogen atoms were refined with anisotropic displacement parameters. All hydrogen atoms were refined using the riding model. Final refinement details are compiled in Table S8. The supplementary crystallographic data for this paper (CCDC2278044) can also be obtained free of charge via www.ccdc.cam.ac.uk/conts/retrieving.html (or from the Cambridge Crystallographic Data Centre, 12, Union Road, Cambridge CB2 1EZ, UK; fax: +44 1223 336033; or deposit@ccdc.cam.ac.uk).

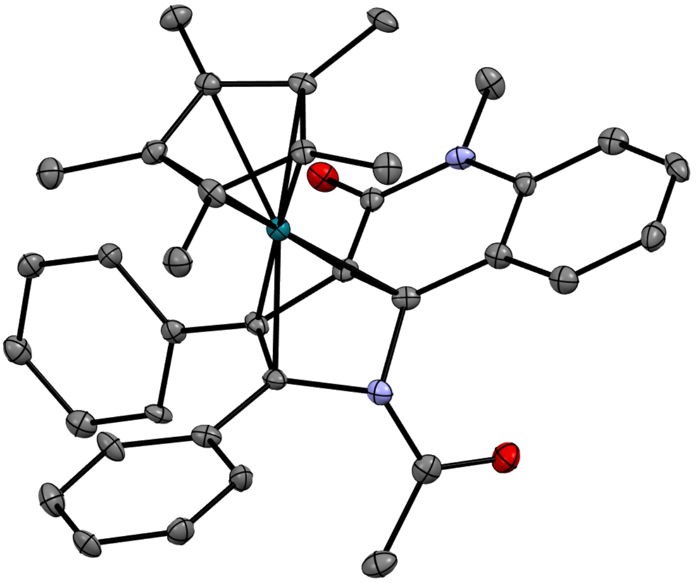

Figure S13. ORTEP plot of A

Table S8. Selected crystallographic data and collection parameters for **A**.

formula	$C_{72}H_{70}N_4O_4Rh_2$	crystal size, mm	0.6 x 0.1 x 0.1
FW	1261.18	maximum 2θ, deg	136.3
crystal system	orthorhombic	reflections collected	51511
space group	Pna21 (#33)	independent reflections [R(int)]	10423 [R(int) = 0.0926]
a, Å	21.2877(5)	max. and min. transmission	0.600/0.262
b, Å	9.8400(2)	goodness-of-fit on F^2	1.029
c, Å	27.3069(6)	R_1 [$I > 2\sigma(I)$]	0.0570
volume, Å^3	5720.0(2)	R, wR_2 (all data)	0.0728, 0.1341
Z	4	Weighting scheme	$R_1 = \Sigma \|\|Fo\| - \|Fc\|\| / \Sigma \|Fo\|$ $wR_2 = [\Sigma(w(Fo^2 - Fc^2)^2)/\Sigma w(Fo^2)^2]^{1/2}$
D (calcd), Mg m^{-3}	1.464		
μ, cm^{-1}	51.065	largest diff. peak and hole, e Å^{-3}	1.22 and –0.68
F(000)	2608.00		

X-Ray Diffraction Analysis of B-Me

Diffraction data were collected in θ ranges specified in Table S9 at 123 K on a Rigaku R-AXIS Rapid diffractometer with graphite monochromatized Cu-Ka radiation (λ = 1.54187 Å). The Lorenz polarization absorption correction was applied. The structure was solved by direct methods and refined by the full-matrix least-squares on F^2. All non-hydrogen atoms were refined with anisotropic displacement parameters. All hydrogen atoms were refined using the riding model. Final refinement details are compiled in Table S9. The supplementary crystallographic data for this paper (CCDC2278045) can also be obtained free of charge via www.ccdc.cam.ac.uk/conts/retrieving.html (or from the Cambridge Crystallographic Data Centre, 12, Union Road, Cambridge CB2 1EZ, UK; fax: +44 1223 336033; or deposit@ccdc.cam.ac.uk).

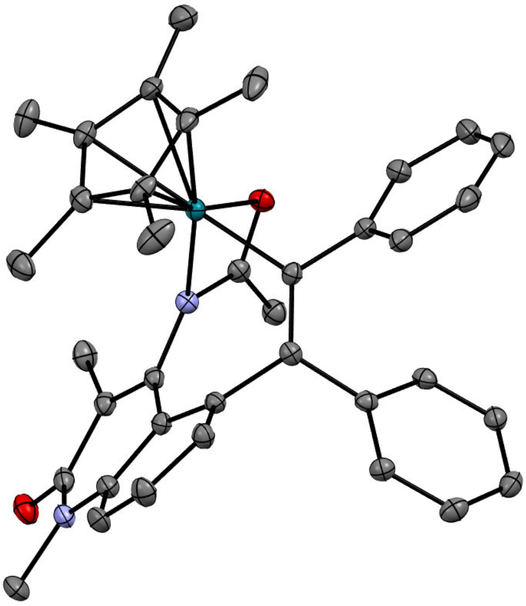

Figure S14. ORTEP plot of B-Me

Table S9. Selected crystallographic data and collection parameters for **B-Me**.

formula	$C_{37}H_{37}N_2O_2Rh$	crystal size, mm	0.3 x 0.1 x 0.1
FW	644.62	maximum 2θ, deg	136.5
crystal system	orthorhombic	reflections collected	63038
space group	Pbca (#61)	independent reflections [R(int)]	5468 [R(int) = 0.0759]
a, Å	17.6227(4)	max. and min. transmission	0.613/0.369
b, Å	18.3009(4)	goodness-of-fit on F^2	1.115
c, Å	18.5577(4)	R_1 [$I > 2\sigma(I)$]	0.0511
volume, $Å^3$	5985.1(2)	R, wR_2 (all data)	0.0632, 0.1233
Z	8	Weighting scheme	$R_1 = \Sigma \|\|Fo\| - \|Fc\|\| / \Sigma \|Fo\|$
D (calcd), Mg m^{-3}	1.431		$wR_2 = [\Sigma(w(Fo^2 - Fc^2)^2)/\Sigma w(Fo^2)^2]^{1/2}$
μ, cm^{-1}	48.925	largest diff. peak and hole, e $Å^{-3}$	0.98 and –0.37
F(000)	2672.00		

9. DFT Calculations

The Gaussian 16 program package was used for all geometry optimizations.[19] Geometry optimization and energy calculations were performed with B3LYP[20] with GD3BJ[21] empirical dispersion along with a combined basis set. The Lanl2DZ basis set[22] with ECP was used for Rh, and the 6-31G(d) basis set[23] was used for other atoms. Thermal correction to Gibbs free energy (TCGFE) including zero-point energy were calculated at the same level of theory. Harmonic frequency calculations were performed at the same level for each stationary point to ensure that it is either an energy minimum (no imaginary frequency) or a transition state (only one imaginary frequency). The connectivity of each step was also confirmed using intrinsic reaction coordinate (IRC)[24] calculation from the transition states, followed by optimization of the resultant geometries. Final energies were obtained using the more extended 6-311+G(d,p)[25] basis set for all atoms except Rh for which SDD[26] was used. To examine the solvent effect, the above single-point energy calculations were performed using the SMD model[27] with dichloroethane as the solvent because the ε value of DiChloroEthane (10.36) is similar to that of $CF_3C_6H_5$ (9.18). The obtained energies, TCGFEs, and IF are summarized in Table S10.

Table S10. Summary of theoretical calculations.

Model	TCGFE/au	Energy/au	imaginary frequency /cm^{-1}
**B**	0.551908	-1764.175154	
**C**	0.551336	-1764.166298	
**TS$_{CD}$**	0.548677	-1764.126033	288.73i
**D**	0.549077	-1764.148695	
**TS$_{BE}$**	0.550221	-1764.131682	302.52i
**E**	0.550802	-1764.135307	

Cartesian coordinates

B

Rh	1.93549	4.15741	8.45946
O	3.91833	5.19347	9.00998
O	-0.41838	8.10964	13.09314
N	2.11672	5.26961	10.23063
N	-1.07716	5.92287	12.79251
C	2.55771	2.64536	9.65069
C	0.38702	4.35022	11.64007
C	2.82901	1.76246	11.96937
C	2.0679	2.48995	10.90322
C	3.57198	1.7773	9.02533
C	4.22257	-0.47351	8.34057
H	4.02813	-1.54285	8.33959
C	1.47134	4.87776	6.29133
C	0.5634	2.94099	7.23738
C	1.20603	5.48624	11.24449
C	4.23627	1.85121	11.9734
H	4.72838	2.43186	11.2026
C	2.22745	1.05781	13.02751
H	1.14862	0.99938	13.09614
C	0.40674	5.28731	7.10623

C	-0.186	7.00401	12.60591
C	4.38729	0.4964	13.97091
H	4.98345	0.00727	14.73612
C	4.70457	2.27653	8.353
H	4.88929	3.34437	8.36835
C	0.71212	3.00719	11.31253
C	5.00283	1.22042	12.94759
H	6.08556	1.30796	12.91483
C	-1.7806	3.61728	12.50688
H	-2.72498	3.82745	12.99061
C	1.61391	3.42167	6.39888
C	3.39194	5.58802	10.10638
C	4.21556	6.28123	11.15548
H	4.4614	7.29694	10.82593
H	5.15575	5.73639	11.2812
H	3.68953	6.33916	12.11083
C	2.99542	0.42886	14.0076
H	2.49689	-0.11455	14.80609
C	-0.12147	4.09411	7.76851
C	-0.82945	4.639	12.32005
C	3.35014	0.38555	9.00619
H	2.48469	-0.01012	9.52852
C	-0.27478	2.03014	11.4777
H	-0.05365	1.01001	11.181
C	5.34278	0.03613	7.6809
H	6.02398	-0.63185	7.16127
C	-2.29308	6.20134	13.54943
H	-2.35541	5.55569	14.43143
H	-3.18219	6.04703	12.92826
H	-2.23829	7.24315	13.85881
C	-1.51267	2.33716	12.04903
H	-2.26268	1.55982	12.16502
C	5.58024	1.41326	7.69875
H	6.45469	1.81916	7.19645
C	-1.32956	4.08808	8.64959
H	-1.37209	3.19346	9.27218
H	-2.24263	4.128	8.03963
H	-1.33712	4.95508	9.31543
C	2.58245	2.5904	5.61676
H	3.54824	3.09445	5.52144
H	2.20443	2.39411	4.60375
H	2.76643	1.63076	6.10518
C	0.23179	1.51061	7.52264
H	-0.22923	1.40314	8.50758
H	1.12695	0.88436	7.50096
H	-0.47187	1.12568	6.77268
C	2.39523	5.73814	5.48906

H	2.26817	5.54854	4.41543
H	3.43974	5.52436	5.7399
H	2.21848	6.80082	5.67009
C	-0.08645	6.67712	7.36686
H	-0.16578	6.87037	8.44231
H	-1.08134	6.83368	6.92991
H	0.58649	7.42756	6.94449
C	0.93899	6.72928	11.73758
H	1.50789	7.59366	11.41698

C

Rh	0.57209	0.89394	0.14567
C	2.13739	2.275	-0.3795
C	3.12405	2.12769	-1.48948
C	0.90246	3.07274	-0.46032
C	0.36622	3.64232	-1.73505
C	0.32454	3.10122	0.8131
C	-0.98651	3.69938	1.21811
C	1.19163	2.33094	1.71947
C	0.91164	2.1392	3.17604
C	2.34885	1.9179	1.00765
C	3.55881	1.22968	1.55392
H	-2.31079	-0.17264	4.28009
H	-0.44955	-1.35337	3.1248
H	2.61399	1.85741	-2.41563
H	3.85418	1.3473	-1.26628
H	3.66511	3.07332	-1.63579
H	0.51606	2.93444	-2.55445
H	0.88543	4.57508	-1.99193
H	-0.70261	3.85878	-1.66041
H	-1.57884	2.98917	1.80659
H	-1.5801	3.98506	0.34626
H	-0.83925	4.59517	1.83575
H	-0.13359	1.86107	3.34256
H	1.10358	3.06755	3.73055
H	1.53976	1.35352	3.60256
H	4.35105	1.9599	1.76509
H	3.95254	0.49685	0.84633
H	3.33271	0.69942	2.48251
H	-4.09504	0.89473	2.97172
N	-0.58423	0.21381	-1.48919
C	-1.88623	0.23703	-1.03894
C	-2.96072	0.72395	-1.73282
C	-4.21081	1.08299	-1.10767
C	-2.24721	-0.16526	3.19571
C	-1.19424	-0.80807	2.55448
C	-1.0934	-0.81529	1.15185
C	-2.04882	-0.06971	0.39065

C	-3.18964	0.47828	1.05238
C	-3.25535	0.44718	2.45681
H	-2.87811	1.00582	-2.77391
C	-0.16257	0.06296	-2.77016
O	0.96448	0.43147	-3.13132
C	-1.05889	-0.65793	-3.77027
H	-1.89079	-1.19229	-3.3077
H	-1.46071	0.05773	-4.49631
H	-0.42778	-1.35984	-4.32208
N	-4.22556	1.0224	0.31079
O	-5.18707	1.51181	-1.7209
C	-5.41915	1.52575	0.97872
H	-5.88833	0.74079	1.58175
H	-5.17529	2.37647	1.62532
H	-6.1036	1.84503	0.1952
C	1.0966	-1.06827	0.20479
C	-0.01605	-1.7255	0.58118
C	2.43133	-1.56977	-0.10781
C	3.05464	-1.29894	-1.33976
C	3.14698	-2.2883	0.86908
C	4.35242	-1.74752	-1.58324
H	2.5074	-0.74106	-2.09308
C	4.44634	-2.72519	0.62246
H	2.66613	-2.49959	1.81963
C	5.05591	-2.45623	-0.60567
H	4.81729	-1.53949	-2.54341
H	4.98412	-3.27657	1.38919
H	6.06948	-2.7964	-0.79873
C	-0.29104	-3.18007	0.53635
C	0.47235	-4.03172	-0.28801
C	-1.35383	-3.75199	1.25978
C	0.2035	-5.3946	-0.35513
H	1.27116	-3.61631	-0.8893
C	-1.62403	-5.11799	1.18628
H	-1.9768	-3.12442	1.88747
C	-0.84353	-5.95027	0.38516
H	0.80907	-6.02545	-1.00035
H	-2.45059	-5.53009	1.75913
H	-1.05302	-7.01467	0.32849

TS_{CD}

Rh	0.72332	-0.11015	0.05469
C	2.4948	1.33808	0.00925
C	3.49826	1.64032	-1.05792
C	1.25523	2.07725	0.21915
C	0.72824	3.10472	-0.73196
C	0.70109	1.69311	1.46931

C	-0.53853	2.23042	2.11452
C	1.54314	0.64389	2.00252
C	1.39777	-0.00394	3.34601
C	2.66227	0.46107	1.11678
C	3.80306	-0.48277	1.34468
H	-3.17012	-1.38001	3.36257
H	-0.78554	-1.48605	2.72239
H	3.01783	1.69906	-2.03483
H	4.27747	0.87471	-1.09972
H	3.98988	2.60013	-0.84606
H	0.89152	2.77667	-1.76081
H	1.24307	4.06381	-0.58534
H	-0.34261	3.27047	-0.59015
H	-1.15365	1.42511	2.52808
H	-1.15247	2.77452	1.39222
H	-0.29152	2.92172	2.93109
H	0.39554	0.14325	3.75282
H	2.11296	0.43069	4.05709
H	1.59283	-1.08036	3.30188
H	4.60739	0.00286	1.91307
H	4.22116	-0.83725	0.39935
H	3.48009	-1.36001	1.91318
H	-4.91018	-1.01344	1.66806
N	-0.38112	-0.06017	-1.76811
C	-1.73589	-0.27073	-1.63376
C	-2.72111	0.00189	-2.55344
C	-4.12841	-0.14867	-2.272
C	-2.87964	-1.19925	2.33107
C	-1.52896	-1.21838	1.98178
C	-1.14729	-0.99372	0.65439
C	-2.13918	-0.70729	-0.31226
C	-3.51283	-0.7467	0.03181
C	-3.86875	-0.99541	1.37386
H	-2.50709	0.40709	-3.53144
C	0.26942	0.07824	-2.96742
O	1.35615	0.66003	-3.04418
C	-0.27781	-0.58842	-4.22438
H	-1.0435	-1.33961	-4.02423
H	-0.6908	0.16005	-4.90941
H	0.57805	-1.05042	-4.72415
N	-4.4678	-0.5165	-0.94204
O	-5.01704	0.06053	-3.09964
C	-5.88484	-0.5927	-0.61482
H	-6.13774	-1.58213	-0.2184
H	-6.15889	0.16856	0.125
H	-6.42876	-0.41364	-1.54057
C	1.14889	-2.00812	-0.59536

C	0.17313	-2.39565	0.16322
C	2.17741	-2.59034	-1.43332
C	2.29049	-3.99201	-1.54215
C	3.07586	-1.78036	-2.14623
C	3.28939	-4.55861	-2.32633
H	1.59401	-4.62598	-1.00366
C	4.07477	-2.35485	-2.93013
H	2.94495	-0.70817	-2.11516
C	4.1898	-3.74278	-3.01929
H	3.36657	-5.63993	-2.39951
H	4.75662	-1.71385	-3.48188
H	4.96865	-4.18899	-3.63128
C	-0.20753	-3.57912	0.92757
C	0.77483	-4.25791	1.66811
C	-1.51608	-4.08953	0.89444
C	0.45624	-5.4283	2.35625
H	1.78487	-3.85974	1.68937
C	-1.82741	-5.26089	1.57648
H	-2.27672	-3.55657	0.33416
C	-0.84452	-5.93194	2.31151
H	1.22274	-5.94383	2.9279
H	-2.8395	-5.6532	1.53758
H	-1.09381	-6.84336	2.84732

D

Rh	0.6622	0.48886	0.28483
C	2.64441	1.80444	0.10701
C	3.74438	1.70379	-0.9001
C	1.50061	2.62156	-0.00212
C	1.14162	3.50933	-1.14939
C	0.70952	2.47095	1.20782
C	-0.51896	3.25829	1.54408
C	1.45425	1.62286	2.11213
C	1.14821	1.39795	3.55788
C	2.5912	1.14695	1.40691
C	3.65488	0.25625	1.96597
H	-2.64571	-1.09441	4.03805
H	-0.37605	-0.81402	3.10448
H	3.33272	1.62205	-1.90881
H	4.37608	0.83072	-0.72706
H	4.38634	2.59424	-0.86006
H	1.59658	3.1583	-2.07528
H	1.47703	4.53622	-0.94949
H	0.05884	3.53581	-1.29813
H	-1.12565	2.74101	2.29092
H	-1.14462	3.40636	0.65973
H	-0.25407	4.24804	1.93972

H	0.07545	1.43849	3.75428
H	1.63323	2.17402	4.16583
H	1.51863	0.42941	3.9061
H	4.43555	0.85238	2.45771
H	4.13476	-0.33204	1.18152
H	3.24651	-0.43456	2.70921
H	-4.64056	-0.46117	2.75601
N	-0.70546	0.66017	-1.29239
C	-2.03362	0.72043	-0.89574
C	-3.13	1.13243	-1.6101
C	-4.47498	1.06372	-1.07614
C	-2.52502	-0.6788	3.04053
C	-1.23505	-0.50796	2.51793
C	-1.07947	0.00812	1.23824
C	-2.21984	0.31171	0.47766
C	-3.52181	0.17722	1.0105
C	-3.65999	-0.32877	2.31657
H	-3.07461	1.52395	-2.61419
C	-0.23525	0.66381	-2.57269
O	0.96416	0.86682	-2.8085
C	-1.15408	0.33912	-3.74317
H	-1.99939	-0.2946	-3.46667
H	-1.55064	1.25851	-4.18837
H	-0.54145	-0.15611	-4.5
N	-4.61186	0.53716	0.23215
O	-5.47037	1.41984	-1.71093
C	-5.96086	0.42017	0.76711
H	-6.18884	-0.6222	1.01702
H	-6.07684	1.03378	1.66775
H	-6.63916	0.77264	-0.00785
C	1.65779	-1.41079	-0.64834
C	0.65189	-1.82318	-0.04837
C	2.9431	-1.53637	-1.28034
C	3.95821	-2.20924	-0.56902
C	3.20957	-1.06895	-2.57823
C	5.21574	-2.39162	-1.13726
H	3.74147	-2.59014	0.42389
C	4.46906	-1.26658	-3.14011
H	2.43314	-0.53476	-3.11106
C	5.4767	-1.91743	-2.42543
H	5.98897	-2.91016	-0.57752
H	4.66574	-0.90097	-4.14397
H	6.45793	-2.05912	-2.86934
C	-0.36196	-2.71453	0.43602
C	-0.09925	-3.52832	1.54979
C	-1.62554	-2.766	-0.17616
C	-1.08702	-4.375	2.04577

H	0.87682	-3.47944	2.02259
C	-2.60954	-3.60797	0.33054
H	-1.82662	-2.12843	-1.02902
C	-2.346	-4.411	1.44321
H	-0.87675	-5.00118	2.90814
H	-3.58801	-3.63177	-0.13986
H	-3.1197	-5.06278	1.83859

TS_{BE}

Rh	0.88832	1.03578	-1.52004
C	1.36173	3.23048	-2.2542
C	1.49665	3.63739	-3.68836
C	0.23758	3.30716	-1.43824
C	-1.13551	3.7987	-1.77299
C	0.58852	2.69639	-0.14532
C	-0.30078	2.72068	1.05602
C	1.99917	2.40465	-0.14193
C	2.78324	1.93067	1.04027
C	2.44717	2.59439	-1.47678
C	3.83714	2.39572	-1.99811
H	-2.87094	0.26624	3.96326
H	-0.69144	-0.603	3.22355
H	1.91082	2.82334	-4.29347
H	2.17144	4.49756	-3.79386
H	0.53091	3.91295	-4.11967
H	-1.39892	4.67736	-1.16963
H	-1.88895	3.02784	-1.57205
H	-1.21925	4.07697	-2.82661
H	0.00399	1.98439	1.80068
H	-1.34062	2.51211	0.79385
H	-0.26613	3.71485	1.52377
H	3.70055	1.41899	0.73485
H	2.19734	1.23233	1.64545
H	3.07124	2.77125	1.68626
H	3.82543	1.97325	-3.00808
H	4.41189	1.72059	-1.35789
H	4.3786	3.35059	-2.04738
H	-4.60929	0.88565	2.34166
N	-0.6293	-0.46157	-1.70562
C	-1.91165	-0.13716	-1.24722
C	-2.89156	0.22088	-2.13689
C	-4.20509	0.63366	-1.71499
C	-2.66893	0.15714	2.90098
C	-1.43889	-0.32964	2.48906
C	-1.12749	-0.45665	1.11789
C	-2.12812	-0.06919	0.17041
C	-3.40423	0.39234	0.61071

C	-3.65781	0.5164	1.98562
H	-2.71024	0.26724	-3.20267
C	-0.3215	-0.84549	-3.00596
O	0.73476	-0.35904	-3.44784
C	-1.11219	-1.89005	-3.73725
H	-2.00229	-2.19124	-3.18379
H	-1.40506	-1.5187	-4.72419
H	-0.45665	-2.75578	-3.88622
N	-4.39053	0.71406	-0.31653
O	-5.11144	0.9346	-2.49431
C	-5.69719	1.16869	0.14315
H	-6.17015	0.40789	0.77315
H	-5.60482	2.09797	0.71614
H	-6.3004	1.34227	-0.74565
C	0.65564	-0.82218	-0.59467
C	0.13558	-1.01727	0.69508
C	1.56494	-1.87282	-1.1535
C	1.09316	-3.1881	-1.26891
C	2.87063	-1.58549	-1.56498
C	1.91044	-4.19423	-1.78124
H	0.08525	-3.41487	-0.93383
C	3.69813	-2.59297	-2.05696
H	3.22841	-0.56341	-1.49076
C	3.21824	-3.89933	-2.17217
H	1.53148	-5.20906	-1.86486
H	4.71496	-2.35761	-2.35878
H	3.85966	-4.68322	-2.56502
C	0.87276	-1.90838	1.62887
C	0.20905	-2.93702	2.32417
C	2.25842	-1.77901	1.82551
C	0.90109	-3.80061	3.16907
H	-0.8609	-3.05966	2.18581
C	2.95534	-2.64829	2.66299
H	2.79124	-0.99131	1.30524
C	2.28109	-3.6638	3.34156
H	0.36353	-4.59155	3.6857
H	4.02777	-2.52597	2.79044
H	2.82195	-4.33977	3.99786

E

Rh	0.91764	0.95325	-0.82584
C	1.02789	3.25709	-1.31072
C	0.71436	3.88348	-2.63436
C	0.18068	3.07603	-0.21715
C	-1.26716	3.42728	-0.09295
C	0.93909	2.36602	0.83578
C	0.4211	2.10785	2.21567

C	2.30122	2.24656	0.41441
C	3.48051	1.84579	1.24555
C	2.31232	2.62781	-0.96486
C	3.50739	2.61246	-1.86718
H	-3.50104	0.0286	4.25581
H	-1.24446	-0.78222	3.75676
H	1.07677	3.26662	-3.46391
H	1.18682	4.87053	-2.73429
H	-0.36284	4.01548	-2.7682
H	-1.42047	4.20861	0.66328
H	-1.85451	2.55645	0.22109
H	-1.68053	3.78371	-1.03952
H	0.89455	1.23129	2.66486
H	-0.65607	1.93126	2.21533
H	0.6202	2.97284	2.86366
H	4.16347	1.18517	0.70092
H	3.16826	1.32667	2.15459
H	4.05892	2.72874	1.55432
H	3.22369	2.37697	-2.89767
H	4.2434	1.87243	-1.54058
H	3.99971	3.59487	-1.87639
H	-5.05055	0.71202	2.47436
N	-0.63109	-0.60409	-1.08824
C	-1.9465	-0.19114	-0.79134
C	-2.7576	0.29123	-1.78746
C	-4.09584	0.74548	-1.51685
C	-3.18023	-0.04883	3.22012
C	-1.90822	-0.51703	2.94447
C	-1.44842	-0.62464	1.60741
C	-2.34416	-0.19211	0.57417
C	-3.6525	0.28836	0.87602
C	-4.06806	0.34535	2.21204
H	-2.42412	0.39854	-2.81165
C	-0.24877	-0.91715	-2.43529
O	0.77104	-0.35717	-2.82677
C	-0.99814	-1.95791	-3.20658
H	-1.99674	-2.12475	-2.80289
H	-1.05814	-1.67377	-4.26011
H	-0.41905	-2.88751	-3.13326
N	-4.48726	0.7039	-0.15625
O	-4.86715	1.1637	-2.38302
C	-5.82957	1.17901	0.15309
H	-6.40394	0.39832	0.66305
H	-5.78858	2.06609	0.79539
H	-6.30159	1.43235	-0.79399
C	0.35598	-1.03541	-0.0587
C	-0.15294	-1.12285	1.26848

C	1.25133	-2.16138	-0.52342
C	0.72528	-3.46081	-0.53608
C	2.57456	-1.96382	-0.92362
C	1.50338	-4.53909	-0.95544
H	-0.29531	-3.6184	-0.19767
C	3.36067	-3.04152	-1.33117
H	2.97339	-0.95391	-0.91178
C	2.82607	-4.3305	-1.35292
H	1.08235	-5.54064	-0.96113
H	4.39073	-2.87379	-1.633
H	3.43754	-5.16955	-1.67283
C	0.72355	-1.74748	2.28004
C	0.24928	-2.72492	3.17506
C	2.08828	-1.41149	2.34367
C	1.09885	-3.32511	4.10123
H	-0.79279	-3.02498	3.12617
C	2.94069	-2.016	3.265
H	2.47193	-0.67207	1.65208
C	2.45022	-2.97345	4.15397
H	0.70716	-4.08186	4.77591
H	3.9905	-1.73512	3.28985
H	3.11181	-3.44435	4.8755

10. References

(1) Shibata, Y.; Tanaka, K. Catalytic [2+2+1] Cross-cyclotrimerization of Silylacetylenes and Two Alkynyl Esters to Produce Substituted Silylfulvenes. *Angew. Chem. Int. Ed.* **2011**, *50*, 10917–10921.

(2) Mio, M. J.; Kopel, L. C.; Braun, J. B.; Gadzikwa, T. L.; Hull, K. L.; Brisbois, R. G.; Markworth, C. J.; Grieco, P. A. One-pot Synthesis of Symmetrical and Unsymmetrical Bisarylethynes by a Modification of the Sonogashira Coupling Reaction.*Org. Lett.* **2002**, *4*, 3199–3202.

(3) Yan, Q.; Shen, X.; Zi, G.; Hou, G. Rh-catalyzed Asymmetric Hydrogenation of α,β- and β,β-disubstituted Unsaturated Boronate Esters. *Chem. – Eur. J.* **2020**, *26*, 5961–5964.

(4) Davison, R. T.; Parker, P. D.; Hou, X.; Chung, C. P.; Augustine, S. A.; Dong, V. M. Enantioselective Addition of A-nitroesters to Alkynes. *Angew. Chem. Int. Ed.* **2021**, *60*, 4599–4603.

(5) Okuno, Y.; Yamashita, M.; Nozaki, K. One-pot Carboboration of Alkynes Using Lithium Borylcyanocuprate and the Subsequent Suzuki-miyaura Cross-coupling of the Resulting Tetrasubstituted Alkenylborane. *Eur. J. Org. Chem.* **2011**, *2011*, 3951–3958.

(6) Zubar, V.; Sklyaruk, J.; Brzozowska, A.; Rueping, M. Chemoselective Hydrogenation of Alkynes to (*Z*)-Alkenes Using an Air-Stable Base Metal Catalyst. *Org. Lett.* **2020**, *22*, 5423–5428.

(7) Wang, Y.-L.; Zhang, W.-M.; Dai, J.-J.; Feng, Y.-S.; Xu, H.-J. Cu-catalyzed intramolecular hydroarylation of alkynes. *RCS Adv.* **2014**, *4*, 61706–61710.

(8) Wong, V. H. L.; White, A. J. P.; Hor, T. S. A.; Hii, K. K. M. Silver-Catalyzed Cyclization of Propargylic Amides to Oxazolines. *Adv. Synth. Catal.* **2015**, *357*, 3943–3948.

(9) Boyer, P. M.; Roy, C. P.; Bielski, J. M.; Merola, J. S. Pentamethylcyclopentadienylrhodium bis-carboxylates: monohapto carboxylate coordination, dihapto carboxylate coordination, and water coordination to Cp*Rh. *Inorg. Chim. Acta.* **1996**, *245*, 7–15.

(10) Kang, J. W.; Moseley, K.; Maitlis, P. M. Pentamethylcyclopentadienylrhodium and -iridium Halides. I. Synthesis and Properties. *J. Am. Chem. Soc.* **1969**, *91*, 5970–5977.

(11) Glasnov, T. N.; Stadlbauer, W.; Kappe, C. O. Microwave-Assisted Multistep Synthesis of Functionalized 4-Arylquinolin-2(1*H*)-ones Using Palladium-Catalyzed Cross-Coupling Chemistry. *J. Org. Chem.* **2005**, *70*, 3864–3870.

(12) Yuan, H.; Yoo, W.-J.; Miyamura, H.; Kobayashi, S. A Cooperative Catalytic System of Platinum/Iridium Alloyed Nanoclusters and a Dimeric Catechol Derivative: An Efficient Synthesis of Quinazolines Through a Sequential Aerobic Oxidative Process. *Adv. Synth. Catal.* **2012**, *354*, 2899–2904.

(13) Smith, C. J.; Ali, A.; Hammond, M. L.; Li, H.; Lu, Z.; Napolitano, J.; Taylor, G. E.; Thompson, C. F.; Anderson, M. S.; Chen, Y.; Eveland, S. S.; Guo, Q.; Hyland, S. A.; Milot, D. P.; Sparrow, C. P.; Wright, S. D.; Cumiskey, A.-M.; Latham, M.; Peterson, L. B.; Rosa, R.; Pivnichny, J. V.; Tong, X.; Xu, S. S.; Sinclair, P. J. Biphenyl-Substituted Oxazolidinones as Cholesteryl Ester Transfer Protein Inhibitors: Modifications of the Oxazolidinone Ring Leading to the Discovery of Anacetrapib. *J. Med. Chem.* **2011**, *54*, 4880–4895.

(14) Adepu, R.; Rajitha, A.; Ahuja, D.; Sharma, A. K.; Ramudu, B.; Kapavarapu, R.; Parsa, K. V. L.; Pal, M. A direct access to bioactive fused N-heterocyclic acetic acid derivatives. *Org. Biomol. Chem.* **2014**, *12*, 2514–2518.

(15) Bagheri, M.; Azizi, N.; Saidi, M. R. An intriguing effect of lithium perchlorate dispersed on silica gel in the bromination of aromatic compounds by N-bromosuccinimide. *Can. J. Chem.* **2005**, *83*, 146–149.

(16) Dooley, J. D.; Reddy Chidipudi, S.; Lam, H. W. Catalyst-Controlled Divergent C–H Functionalization of Unsymmetrical 2-Aryl Cyclic 1,3-Dicarbonyl Compounds with Alkynes and Alkenes. *J. Am. Chem. Soc.* **2013**, *135*, 10829–10836.

(17) Dittmer, D. C.; Li, Q.; Avilov, D. V. Synthesis of Coumarins, 4-Hydroxycoumarins, and 4-Hydroxyquinolinones by Tellurium-Triggered Cyclizations. *J. Org. Chem.* **2005**, *70*, 4682–4686.

(18) Yamamoto, Y.; Hirako, N.; Yasui, T. A Combined Experimental and Computational Study on the Palladium-Catalyzed Sequential [2+2+1] Spirocyclization/Arene C–H Activation of 4-Iodo-2-quinolones with Diphenylacetylene. *Bull. Chem. Soc. Jpn.* **2021**, *94*, 623–631.

(19) Gaussian 16, Revision C.02, Frisch, M. J.; Trucks, G. W.; Schlegel, H. B.; Scuseria, G. E.; Robb, M. A.; Cheeseman, J. R.; Scalmani, G.; Barone, V.; Petersson, G. A.; Nakatsuji, H.; Li, X.; Caricato, M.; Marenich, A. V.; Bloino, J.; Janesko, B. G.; Gomperts, R.; Mennucci, B.; Hratchian, H. P.; Ortiz, J. V.; Izmaylov, A. F.; Sonnenberg, J. L.; Williams-Young, D.; Ding, F.; Lipparini, F.; Egidi, F.; Goings, J.; Peng, B.; Petrone, A.; Henderson, T.; Ranasinghe, D.; Zakrzewski, V. G.; Gao, J.; Rega, N.; Zheng, G.; Liang, W.; Hada, M.; Ehara, M.; Toyota, K.; Fukuda, R.; Hasegawa, J.; Ishida, M.; Nakajima, T.; Honda, Y.; Kitao, O.; Nakai, H.; Vreven, T.; Throssell, K.; Montgomery, J. A., Jr.; Peralta, J. E.; Ogliaro, F.; Bearpark, M. J.; Heyd, J. J.; Brothers, E. N.; Kudin, K. N.; Staroverov, V. N.; Keith, T. A.; Kobayashi, R.; Normand, J.; Raghavachari, K.; Rendell, A. P.; Burant, J. C.; Iyengar, S. S.; Tomasi, J.; Cossi, M.; Millam, J. M.; Klene, M.; Adamo, C.; Cammi, R.; Ochterski, J. W.; Martin, R. L.; Morokuma, K.; Farkas, O.; Foresman, J. B.; Fox, D. J. Gaussian, Inc., Wallingford CT, 2016.

(20) a) Lee, C.; Yang, W.; Parr, R. G. Development of the Colle-Salvetti correlation-energy formula into a functional of the electron density. *Phys. Rev. B* **1988**, *37*, 785–789. b) Becke, A. D. Density-functional thermochemistry. III. The role of exact exchange. *J. Chem. Phys.* **1993**, *98*, 5648–5652. c) Stephens, P. J.; Devlin, F. J.; Chabalowski, C. F.; Frisch, M. J. *Ab Initio* Calculation of Vibrational Absorption and Circular Dichroism Spectra Using Density Functional Force Fields. *J. Phys. Chem.* **1994**, *98*, 11623–11627. d) Kohn, W.; Becke, A. D.; Parr, R. G. Density Functional Theory of Electronic Structure. *J. Phys. Chem.* **1996**, *100*, 12974–12980.

(21) Grimme, S.; Ehrlich, S.; Goerigk, L. Effect of the Damping Function in Dispersion Corrected Density Functional Theory. *J. Comput. Chem.* **2011**, *32*, 1456–1465.

(22) Hay, P. J.; Wadt, W. R. *Ab initio* effective core potentials for molecular calculations. Potentials for K to Au including the outermost core orbitals *J. Chem. Phys.* **1985**, *82*, 299–310.

(23) a) Hehre, W. J.; Ditchfield, R.; Pople, J. A. Self—Consistent Molecular Orbital Methods. XII. Further Extensions of Gaussian—Type Basis Sets for Use in Molecular Orbital Studies of Organic Molecules. *J. Chem. Phys.* **1972**, *56*, 2257–2261. b) Hariharan, P. C.; Pople, J. A. The Influence of Polarization

Functions on Molecular Orbital Hydrogenation Energies. *Theor. Chim. Acta* **1973**, *28*, 213–222. c) Fracl, M. M.; Pietro, W. J.; Hehre, W. J.; Binkley, J. S.; Gordon, M. S.; DeFrees, D. J.; Pople, J. A. Self-consistent molecular orbital methods. XXIII. A polarization-type basis set for second-row elements. *J. Chem. Phys.* **1982**, *77*, 3654–3665.

(24) a) Fukui, K. The Path of Chemical Reactions - The IRC Approach. *Acc. Chem. Res.* **1981**, *14*, 363–368. b) Gonzalez, C.; Schlegel, H. B. An improved algorithm for reaction path following. *J. Chem. Phys.* **1989**, *90*, 2154–2161. c) Gonzalez, C.; Schlegel, H. B. Reaction Path Following in Mass-Weighted Internal Coordinates. *J. Phys. Chem.* **1990**, *94*, 5523–5527.

(25) a) Krishnan, R.; Binkley, J. S.; Seeger, R.; Pople, J. A. Self-consistent molecular orbital methods. XX. A basis set for correlated wave functions. *J. Chem. Phys.* **1980**, *72*, 650–654. b) McLean, A. D.; Chandler, G. S. Contracted Gaussian basis sets for molecular calculations. I. Second row atoms, Z=11–18. *J. Chem. Phys.* **1980**, *72*, 5639–5648. c) Frisch, M. J.; Pople, J. A.; Binkley, J. S. Self-consistent molecular orbital methods 25. Supplementary functions for Gaussian basis sets. *J. Chem. Phys.* **1984**, *80*, 3265–3269. d) Clark, T.; Chandrasekhar, J.; Spitznagel, G. W.; Schleyer, P. V. R. Efficient Diffuse Function-Augmented Basis Sets for Anion Calculations. III. The 3-21+G Basis Set for First-row Elements, Li-f. *J. Comput. Chem.* **1983**, *4*, 294–301.

(26) a) Andrae, D.; Häussermann, U.; Dolg, M.; Stoll, H.; Preuß, H. Energy-adjusted*ab initio* pseudopotentials for the second and third row transition elements *Theor. Chim. Acta.* **1990**, *77*, 123–141. b) Martin, J. M. L.; Sundermann, A. Correlation consistent valence basis sets for use with the Stuttgart–Dresden–Bonn relativistic effective core potentials: the atoms Ga–kr and In–xe. *J. Chem. Phys.* **2001**, *114*, 3408–3420.

(27) Marenich, A. V.; Cramer, C. J.; Truhlar, D. G. Universal Solvation Model Based on Solute Electron Density and on a Continuum Model of the Solvent Defined by the Bulk Dielectric Constant and Atomic Surface Tensions. *J. Phys. Chem. B* **2009**, *113*, 6378–6396.

12. NMR Spectra

^{1}H NMR (400 MHz, $CDCl_3$) of S2

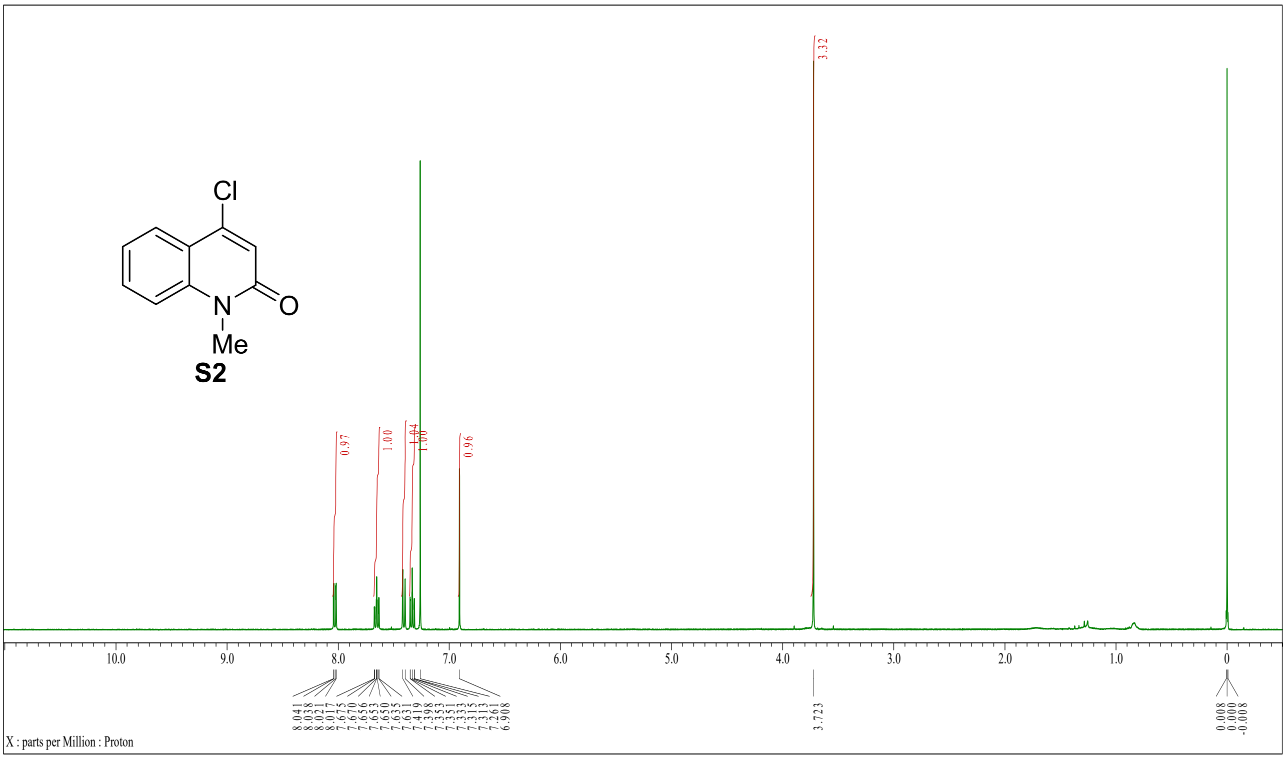

^{13}C NMR (100 MHz, $CDCl_3$) of S2

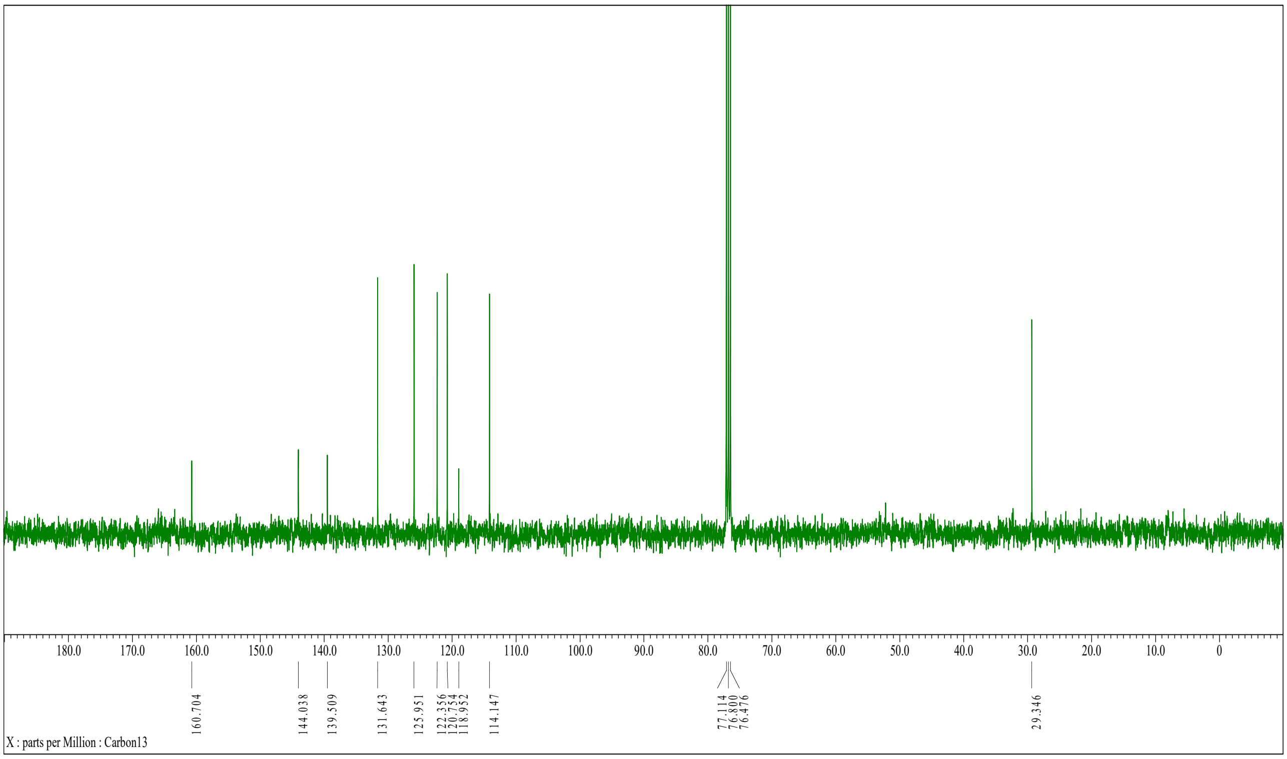

^{1}H NMR (400 MHz, $CDCl_3$) of S3

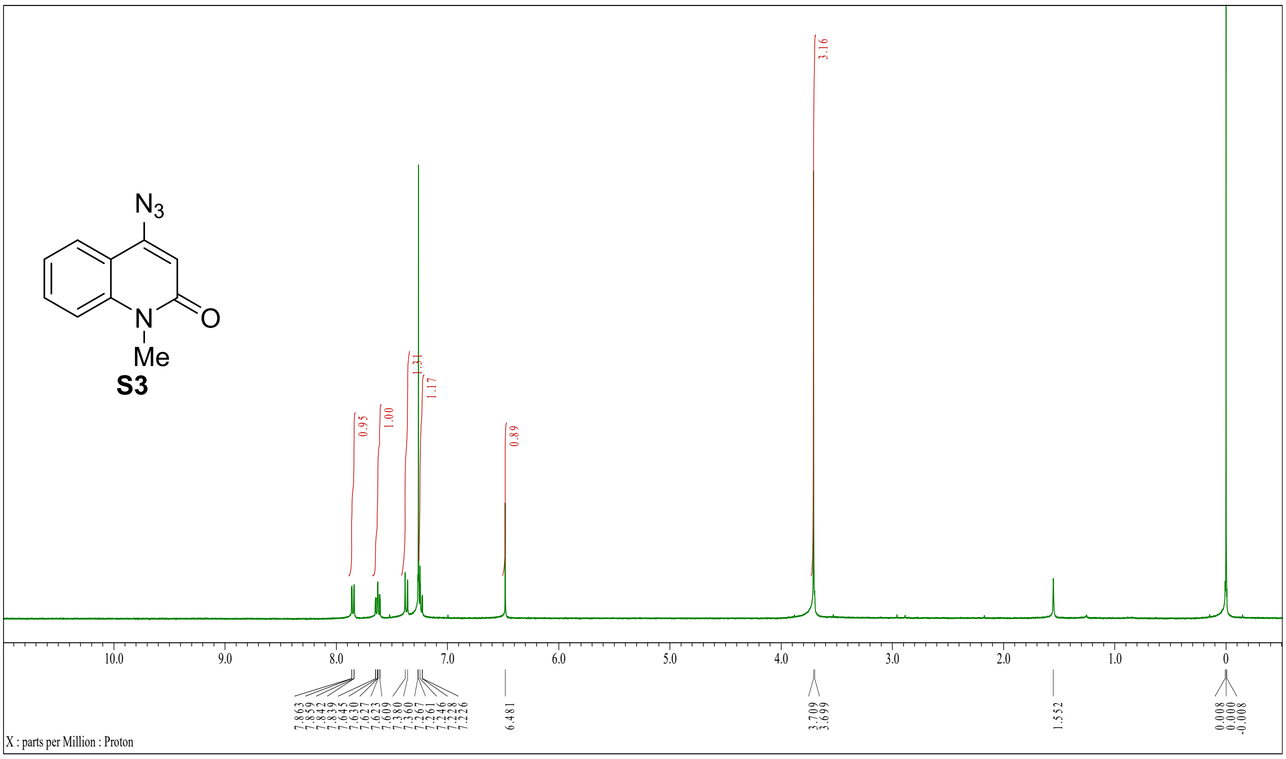

^{13}C NMR (100 MHz, $CDCl_3$) of S3

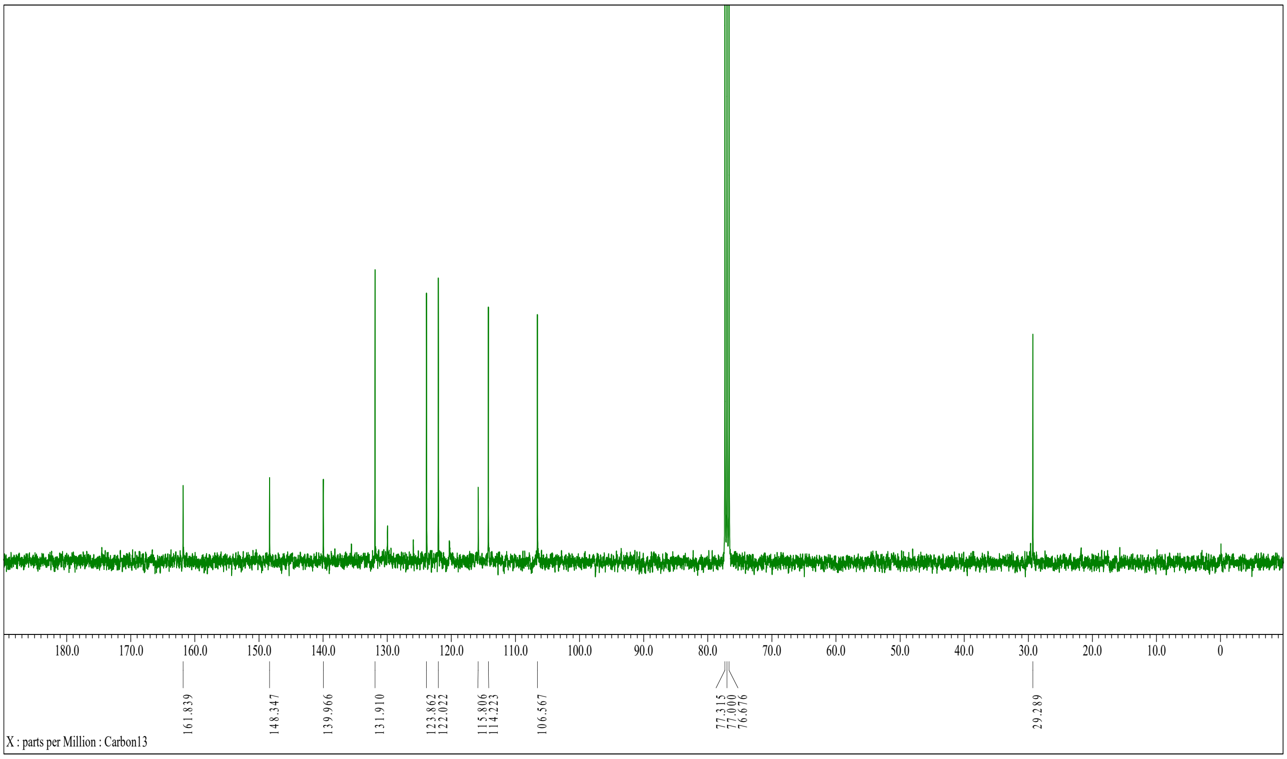

^{1}H NMR (400 MHz, $CDCl_3$) of S4

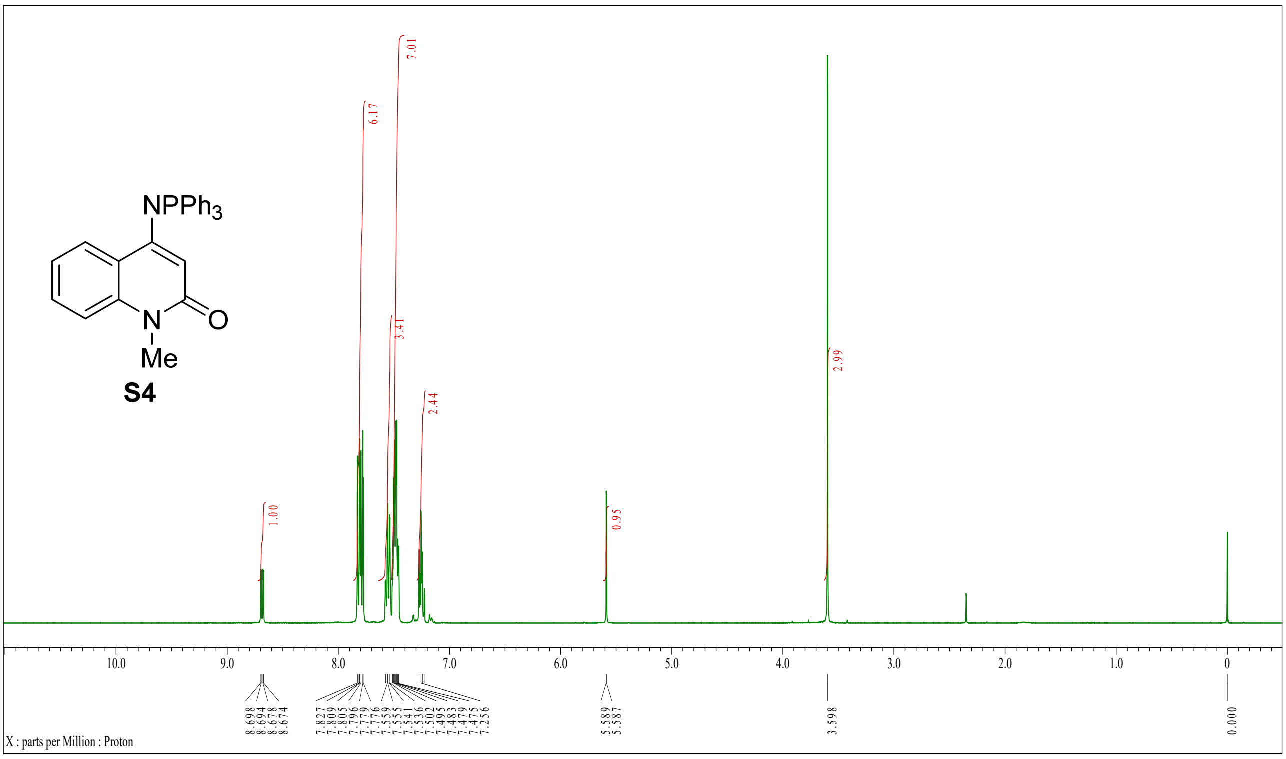

^{13}C NMR (100 MHz, $CDCl_3$) of S4

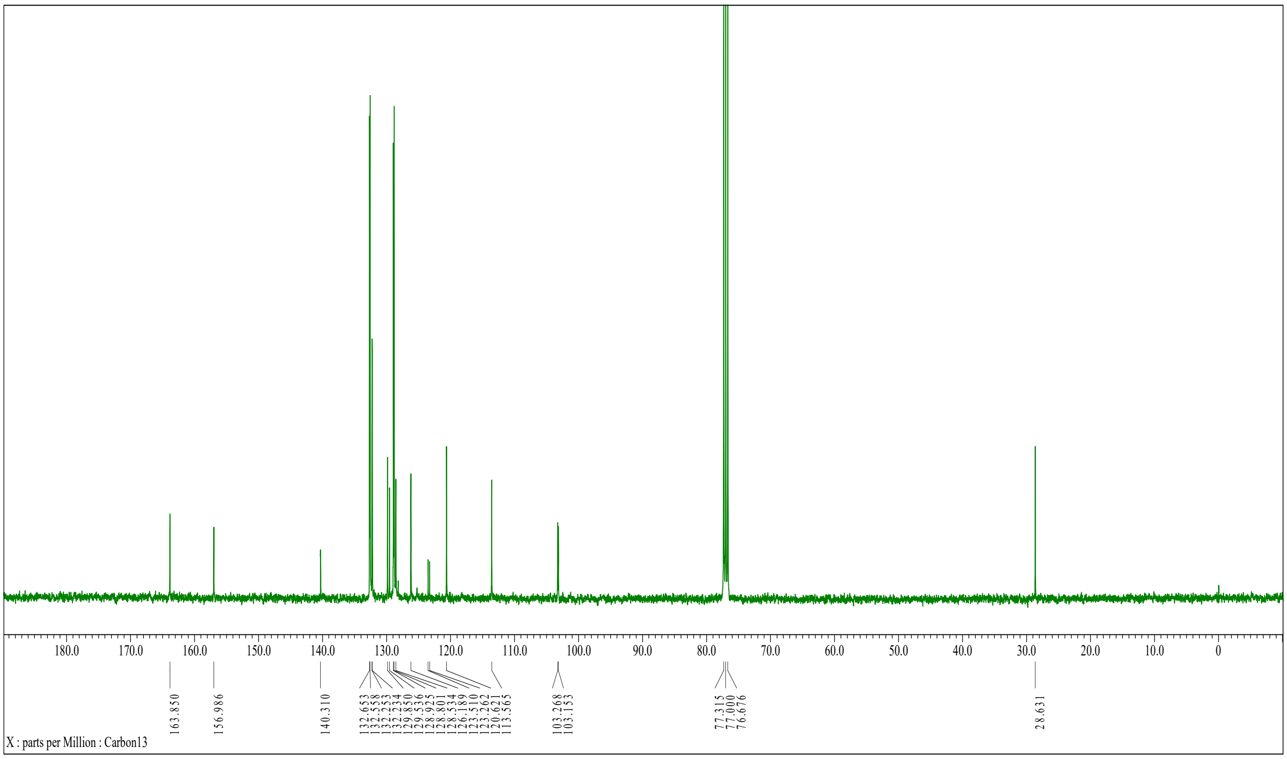

^{31}P NMR (162 MHz, $CHCl_3$) of S4

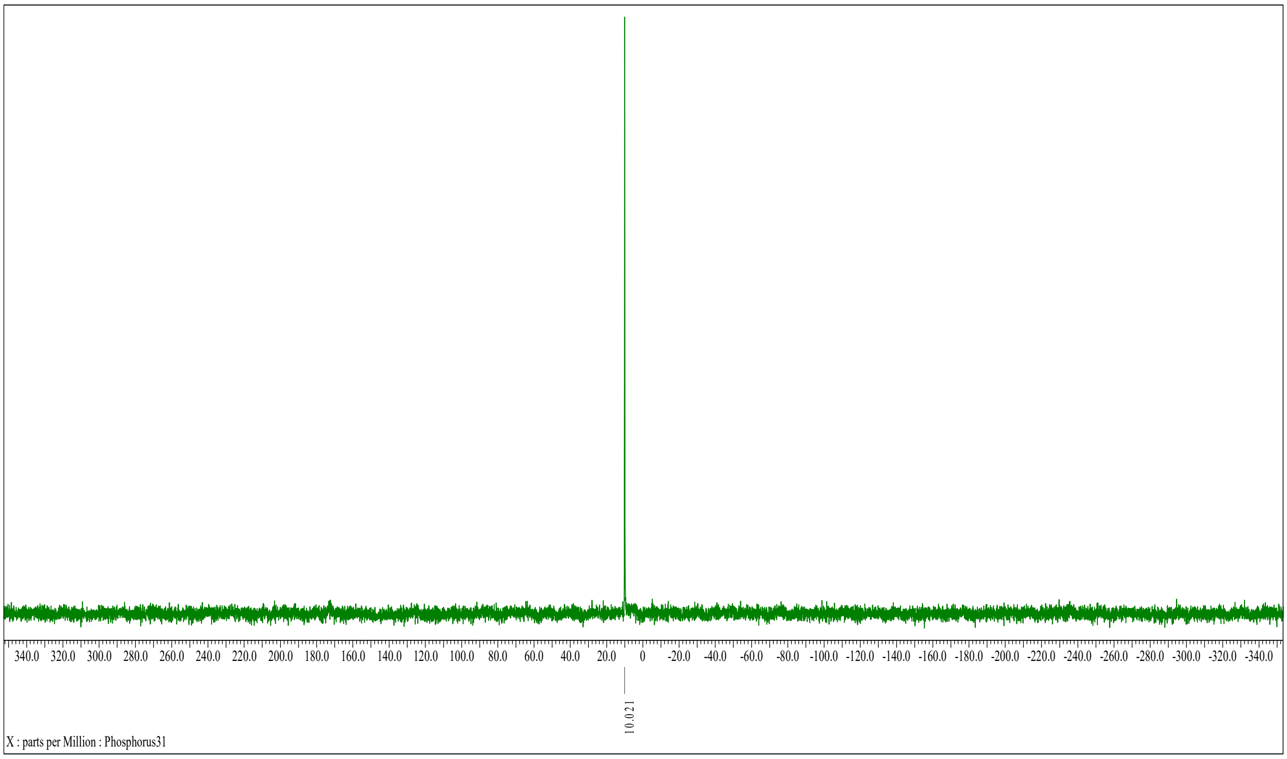

^{1}H NMR (400 MHz, $CDCl_3$) of S5

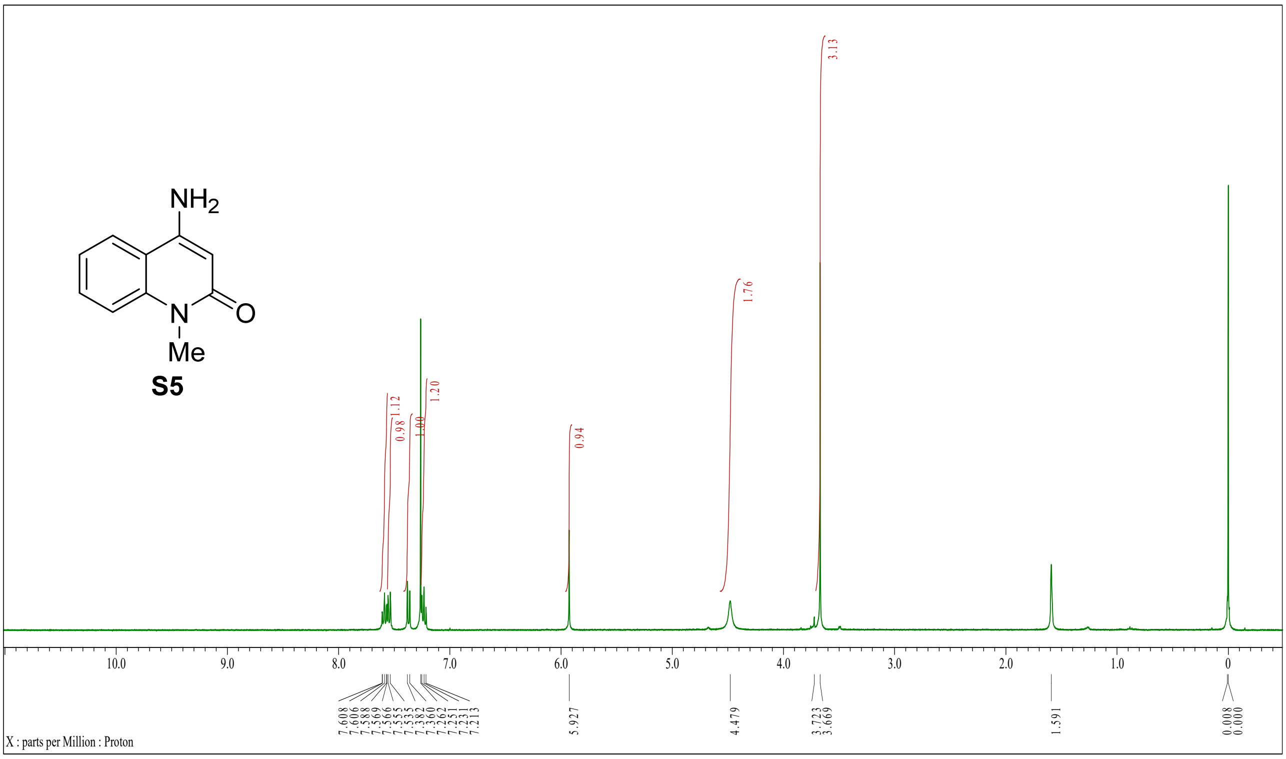

^{13}C NMR (100 MHz, DMSO-d_6) of S5

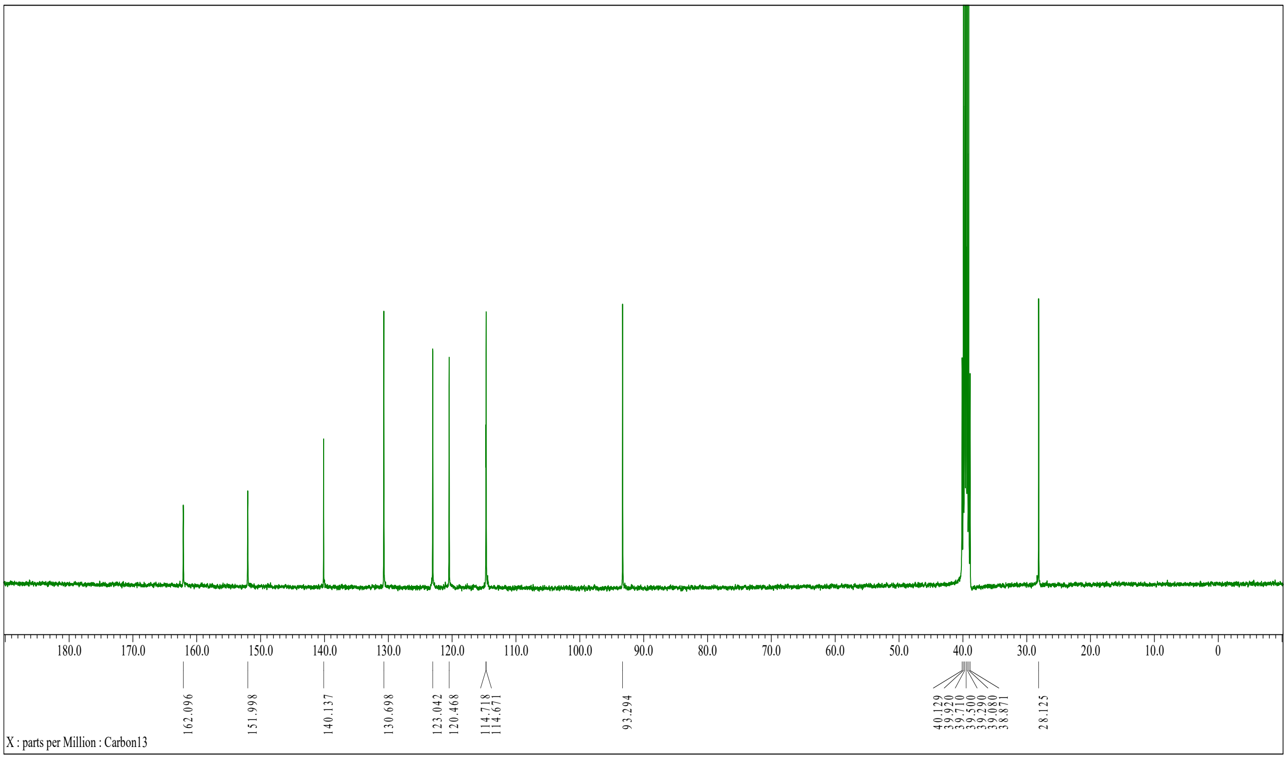

^{1}H NMR (400 MHz, $CDCl_3$) of 1a

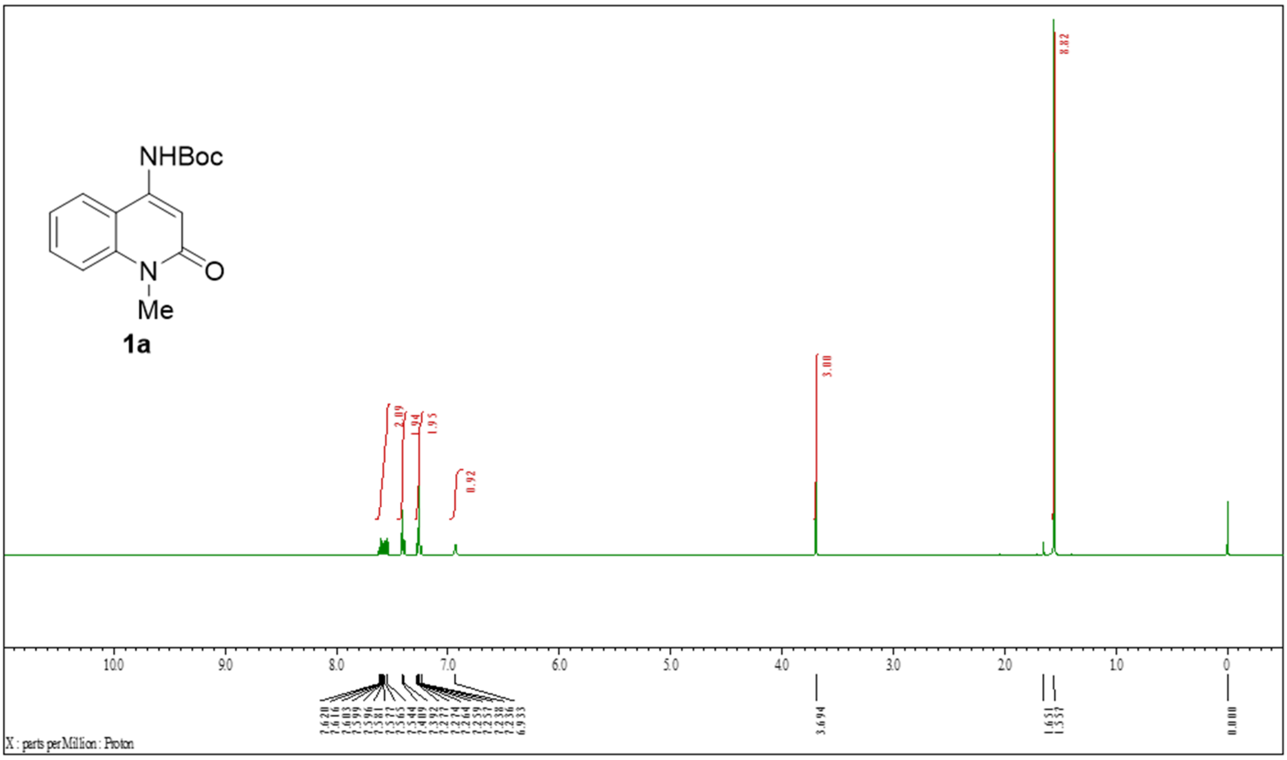

^{13}C NMR (100 MHz, $CDCl_3$) of 1a

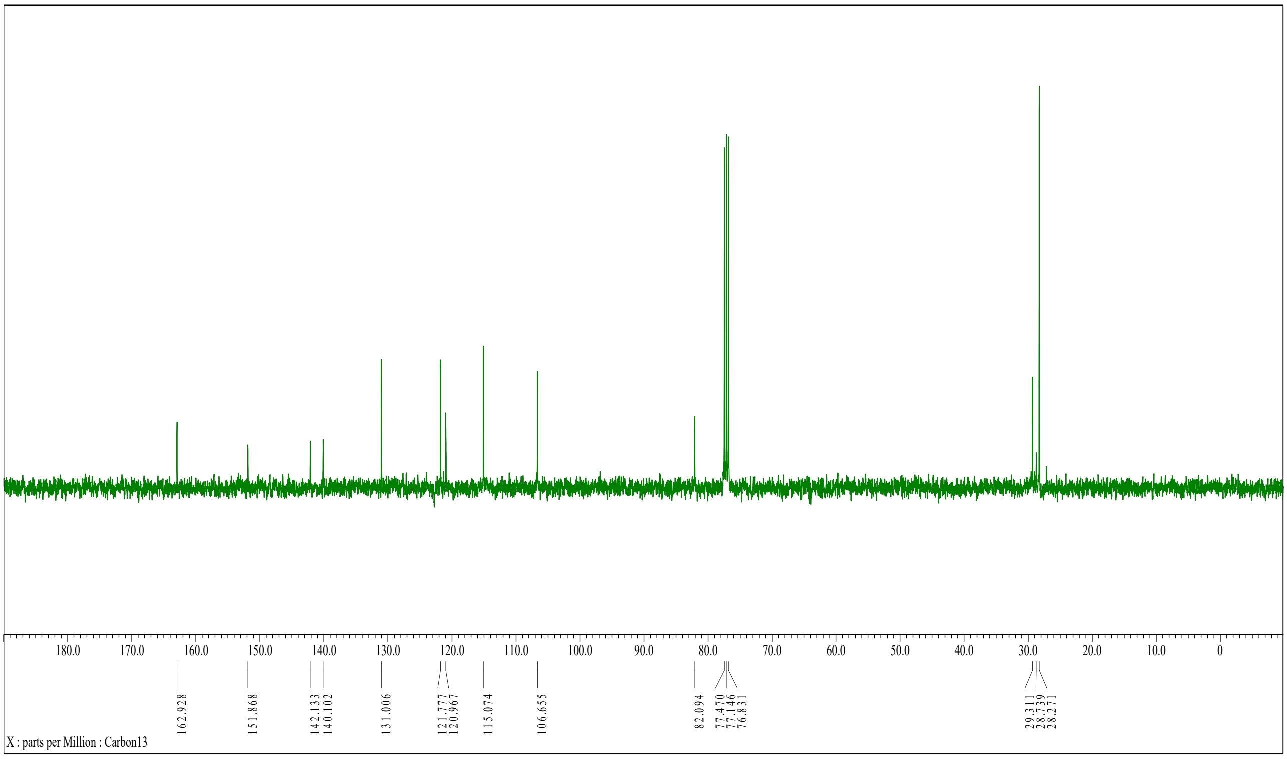

^{1}H NMR (400 MHz, $CDCl_3$) of 1b

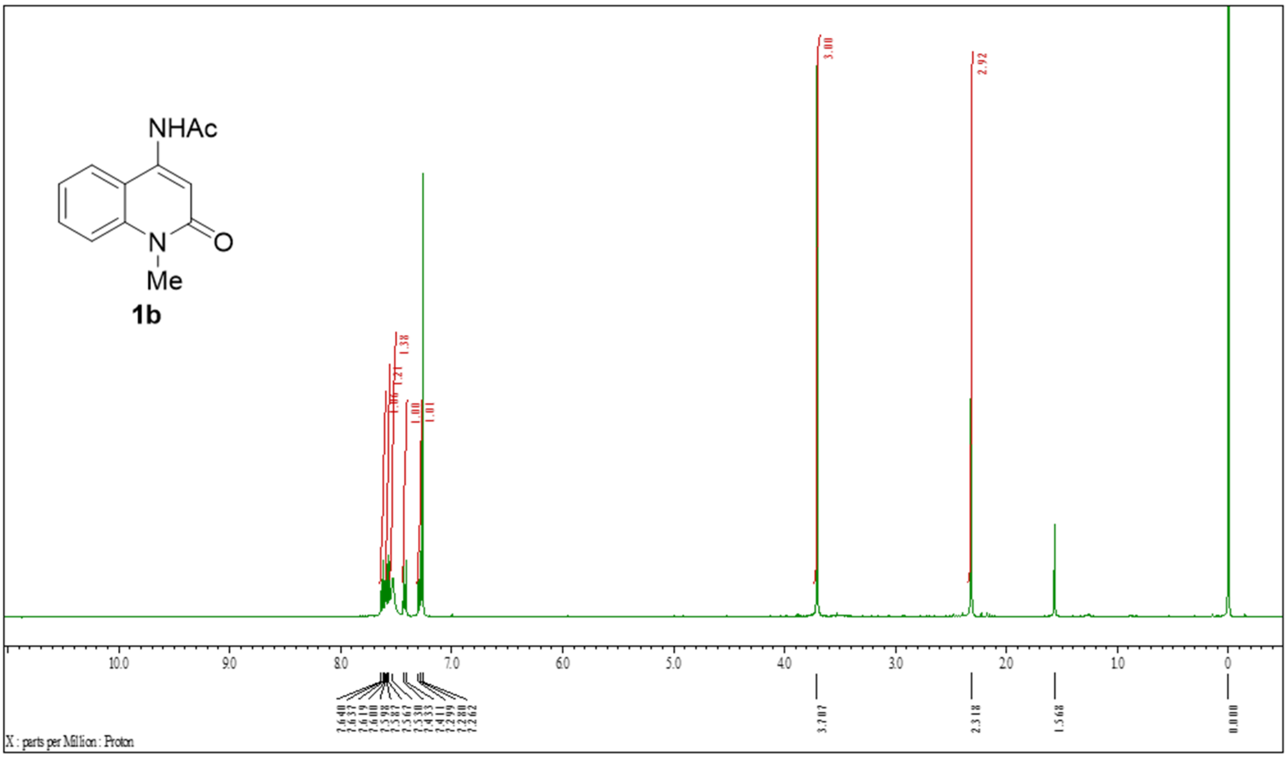

^{13}C NMR (100 MHz, $CDCl_3$) of 1b

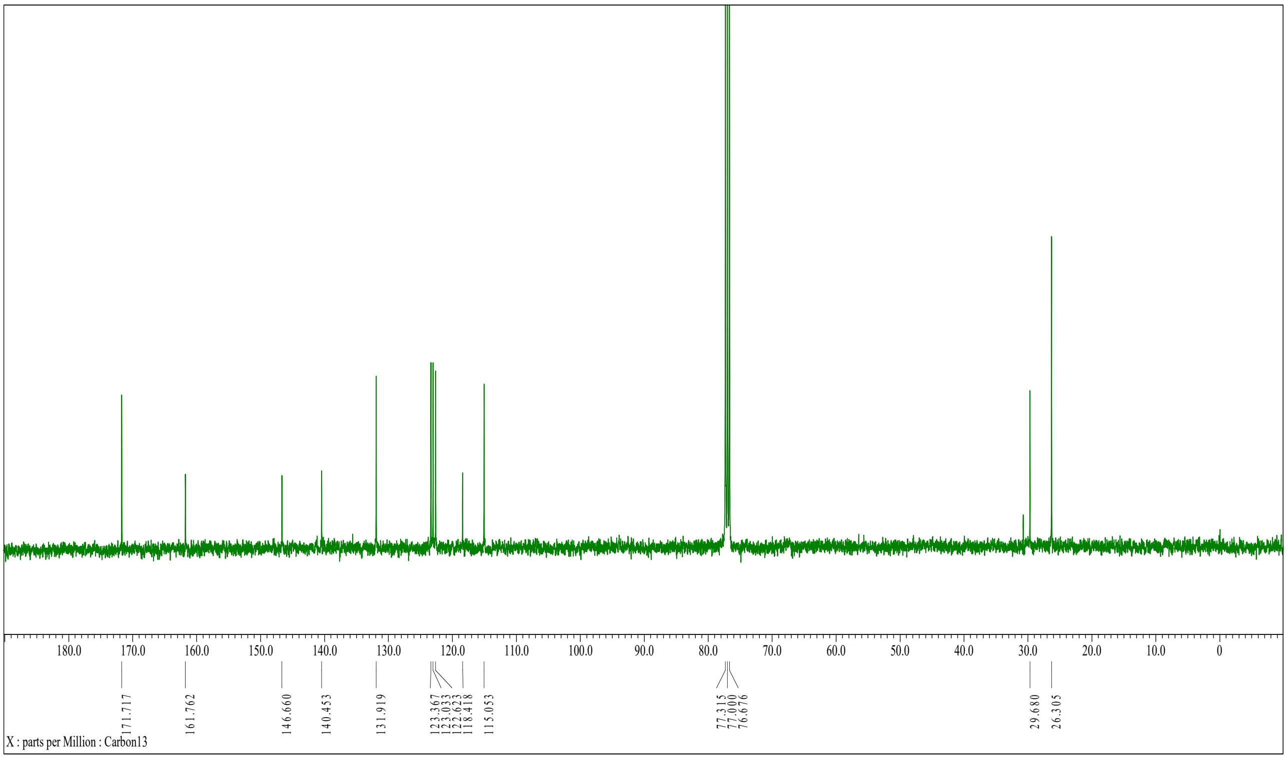

^{1}H NMR (400 MHz, $CDCl_3$) of 1b’

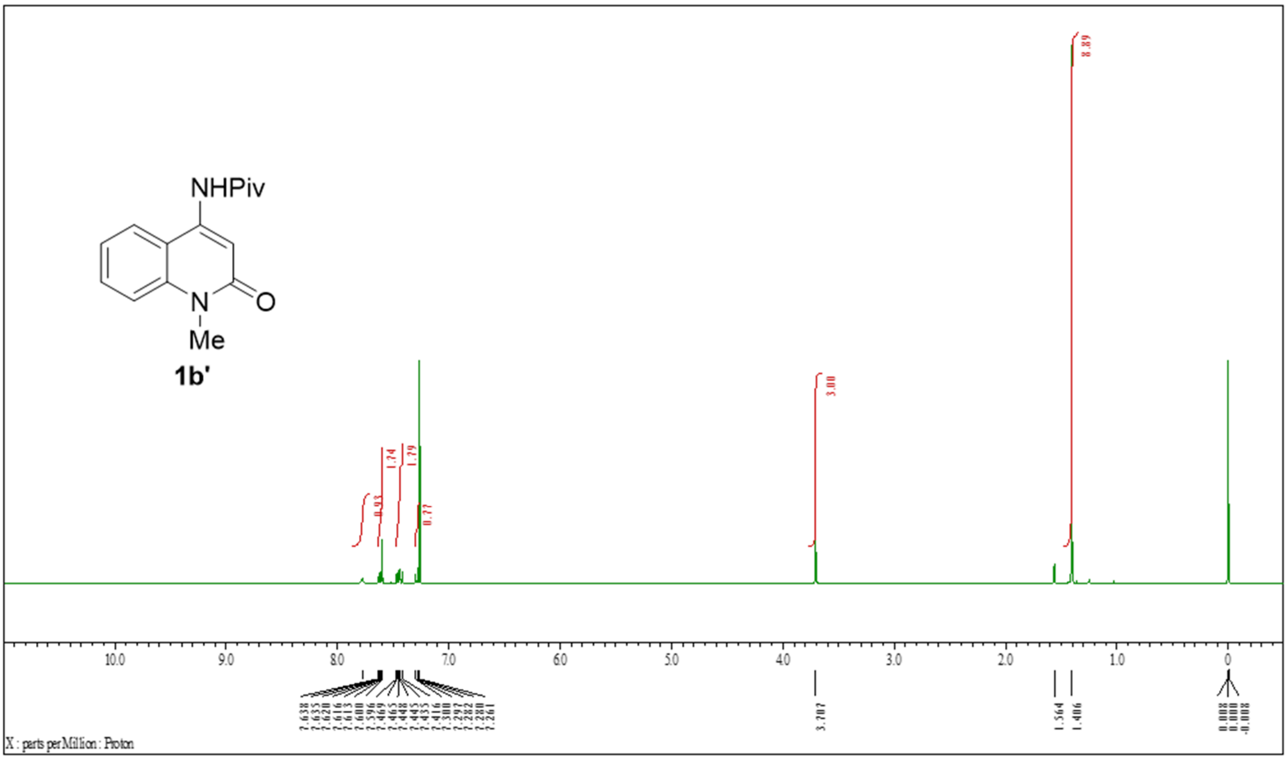

^{13}C NMR (100 MHz, $CDCl_3$) of 1b’

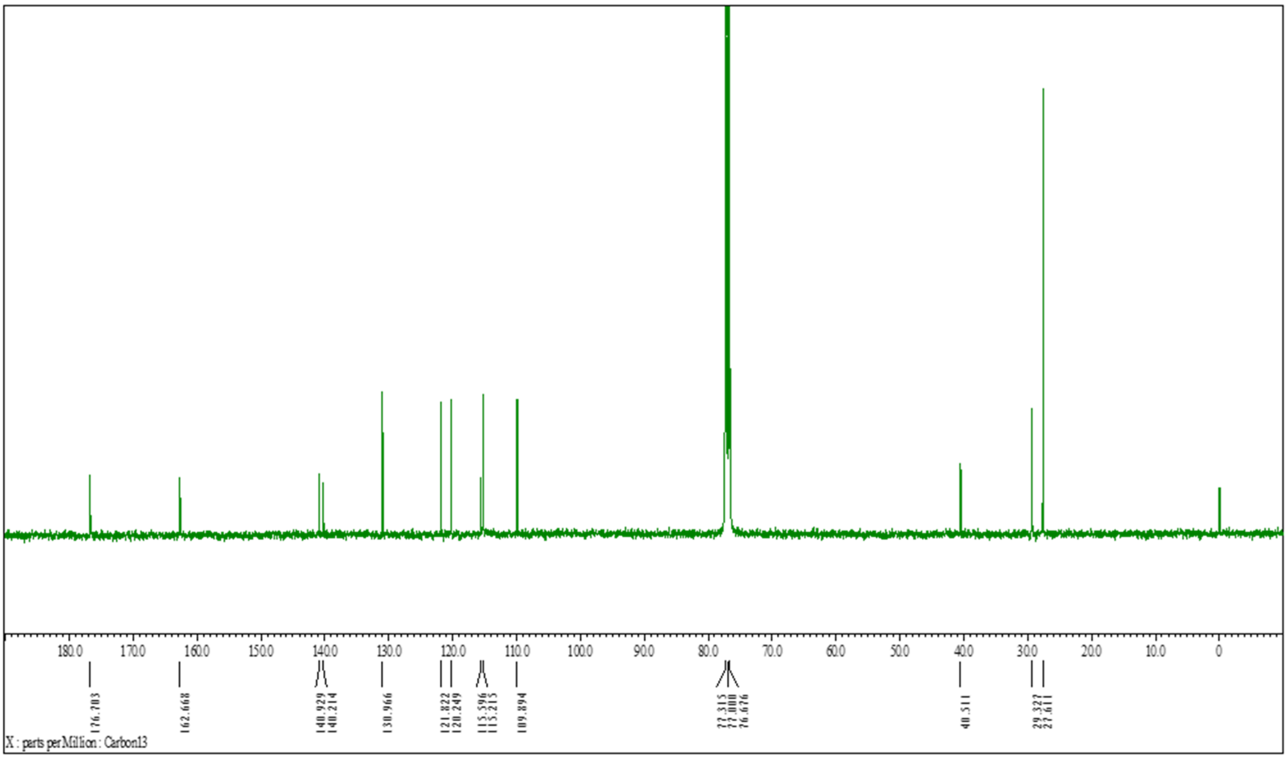

^{1}H NMR (400 MHz, $CDCl_3$) of S7c

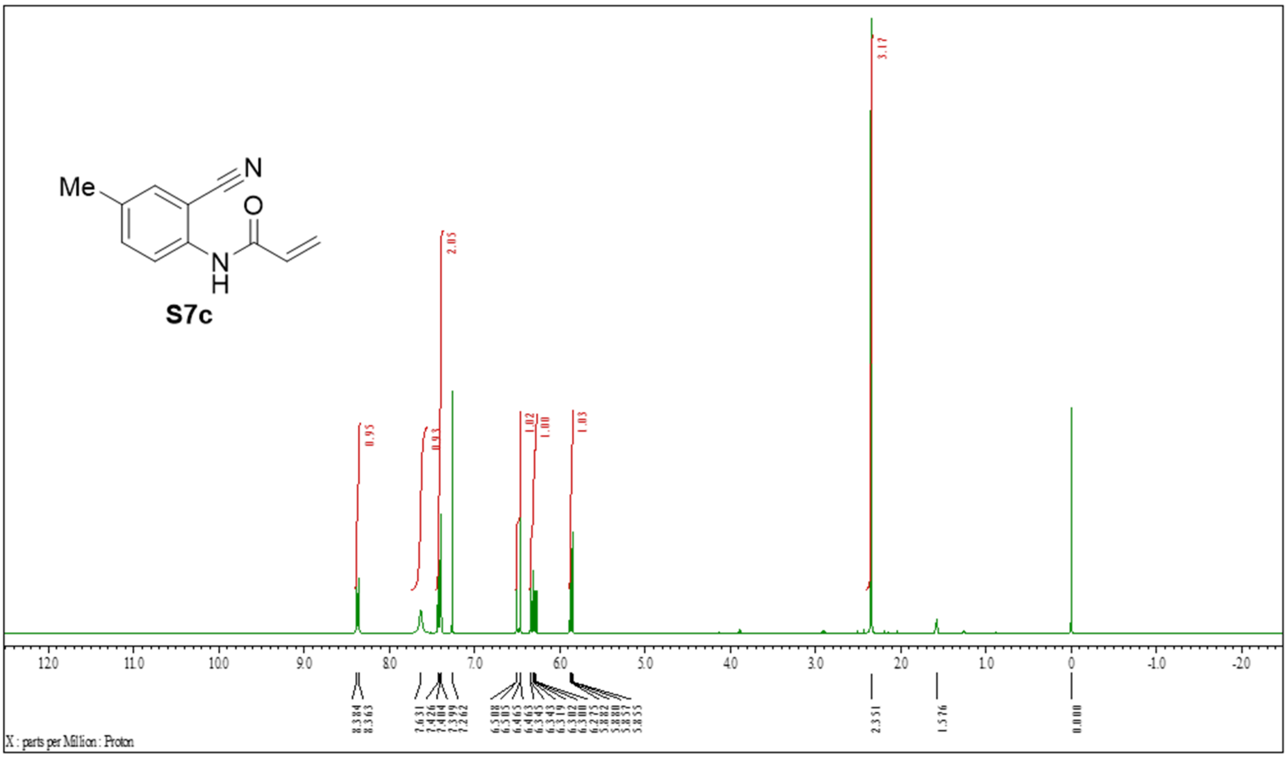

^{13}C NMR (100 MHz, $CDCl_3$) of S7c

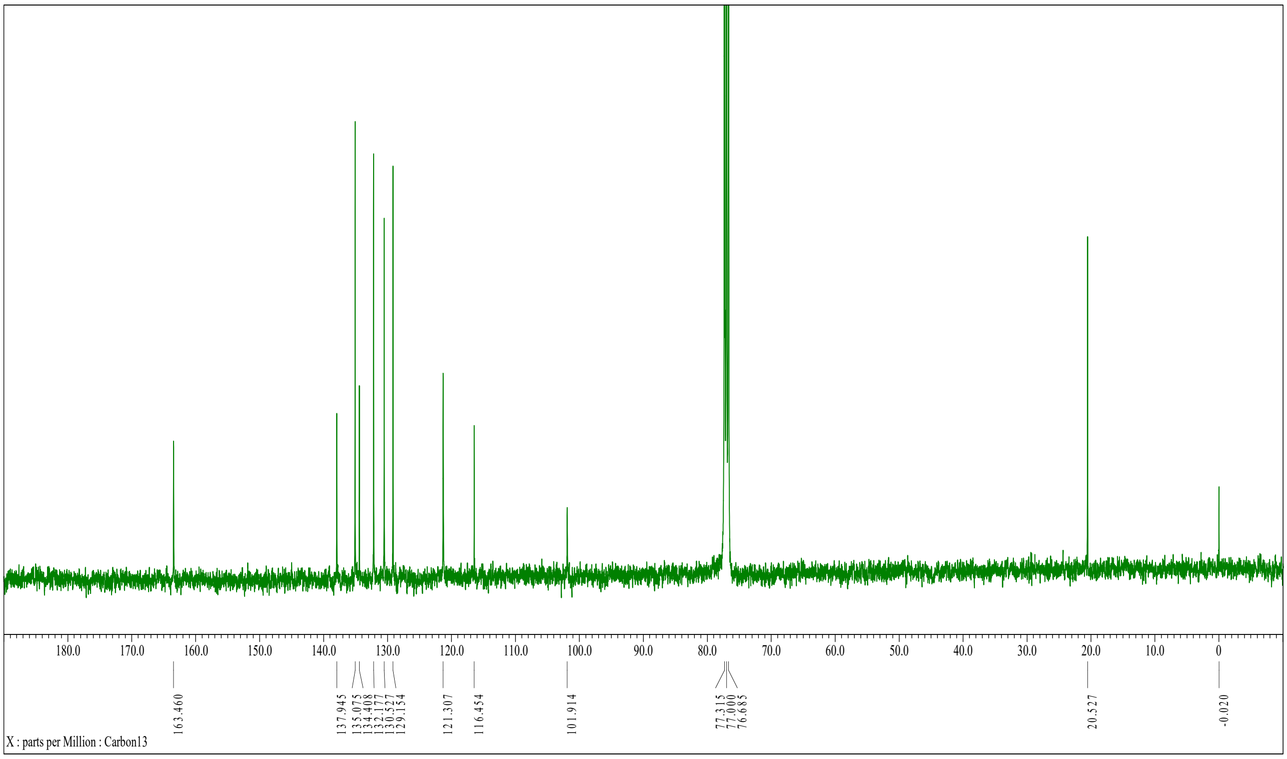

^{1}H NMR (400 MHz, $CDCl_3$) of S8c

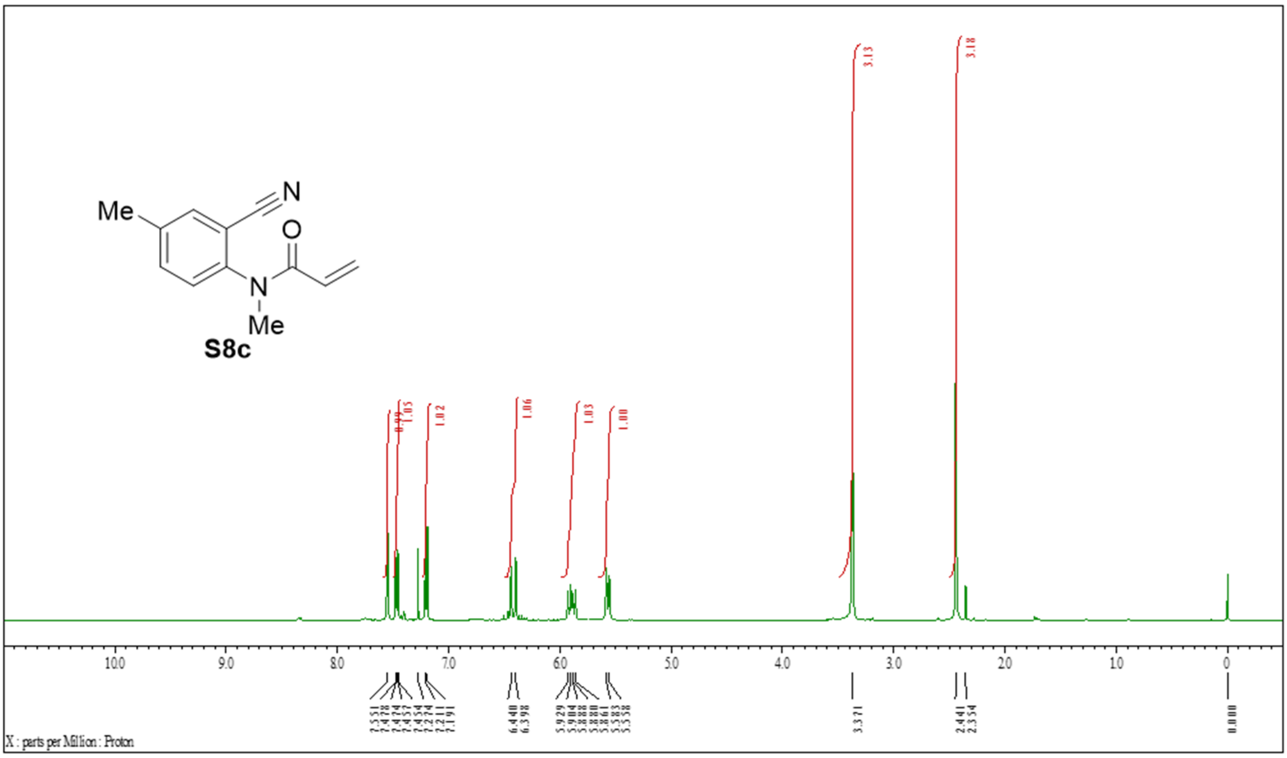

^{13}C NMR (100 MHz, $CDCl_3$) of S8c

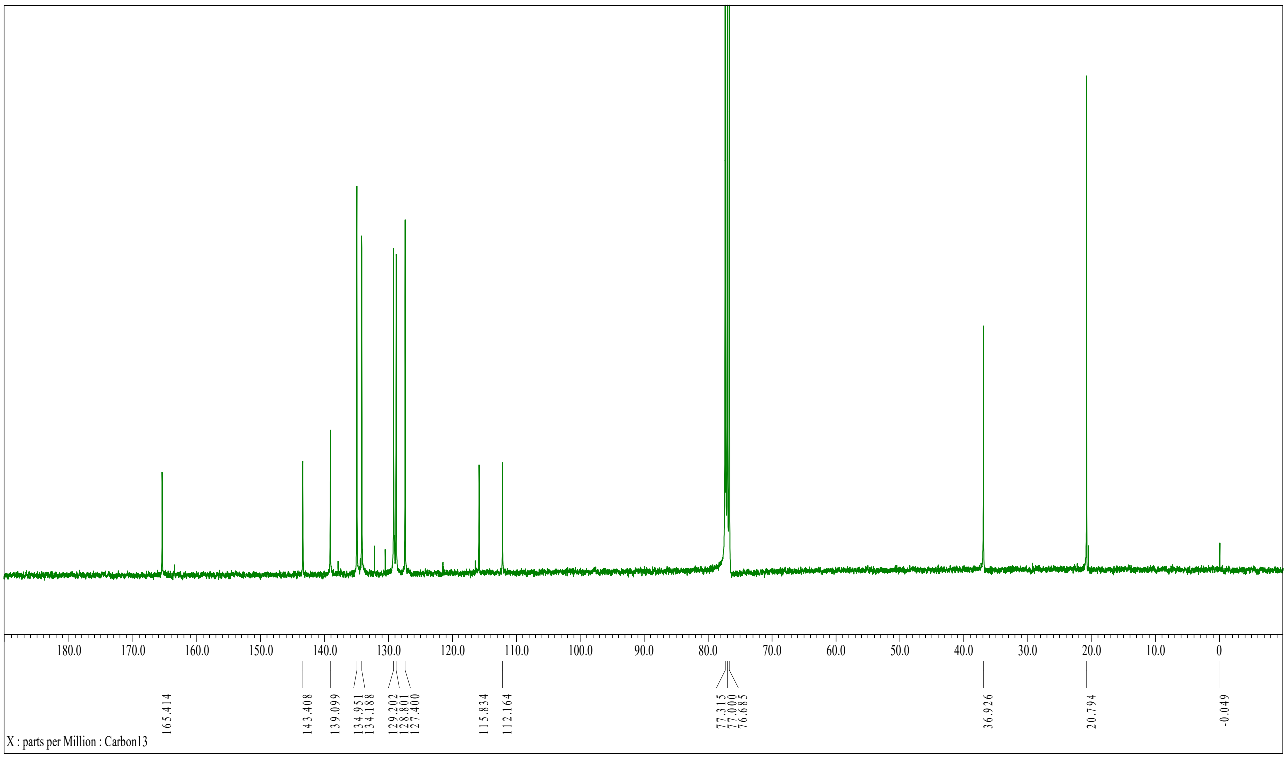

^{1}H NMR (400 MHz, DMSO-d_6) of 1c

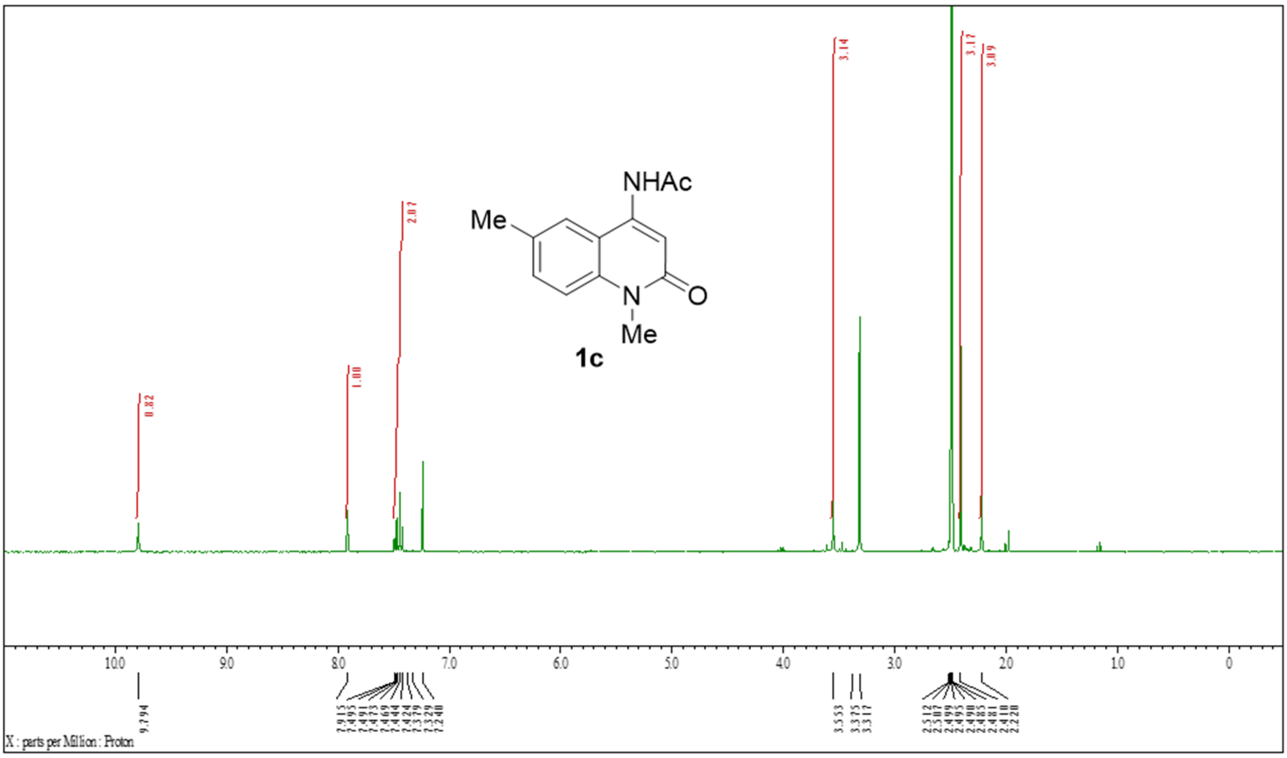

^{13}C NMR (100 MHz, DMSO-d_6) of 1c

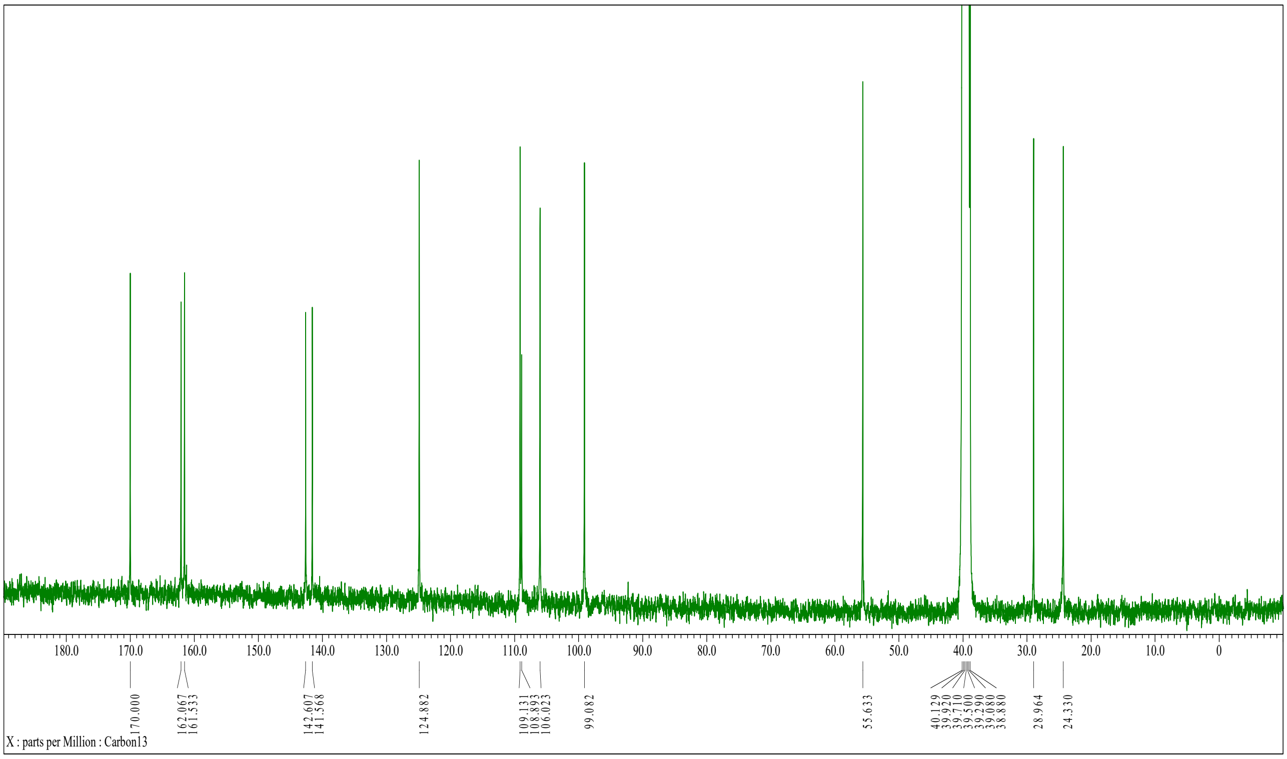

^{1}H NMR (400 MHz, $CDCl_3$) of S7d

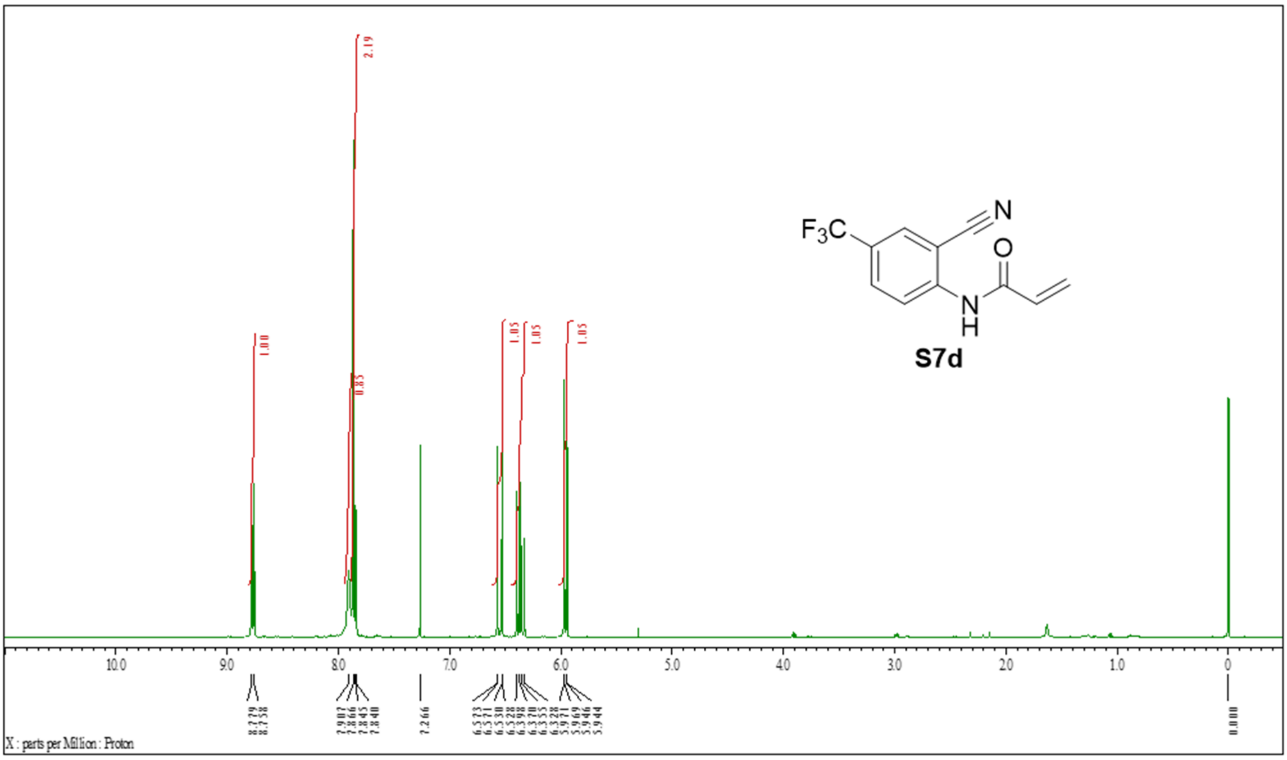

^{13}C NMR (100 MHz, $CDCl_3$) of S7d

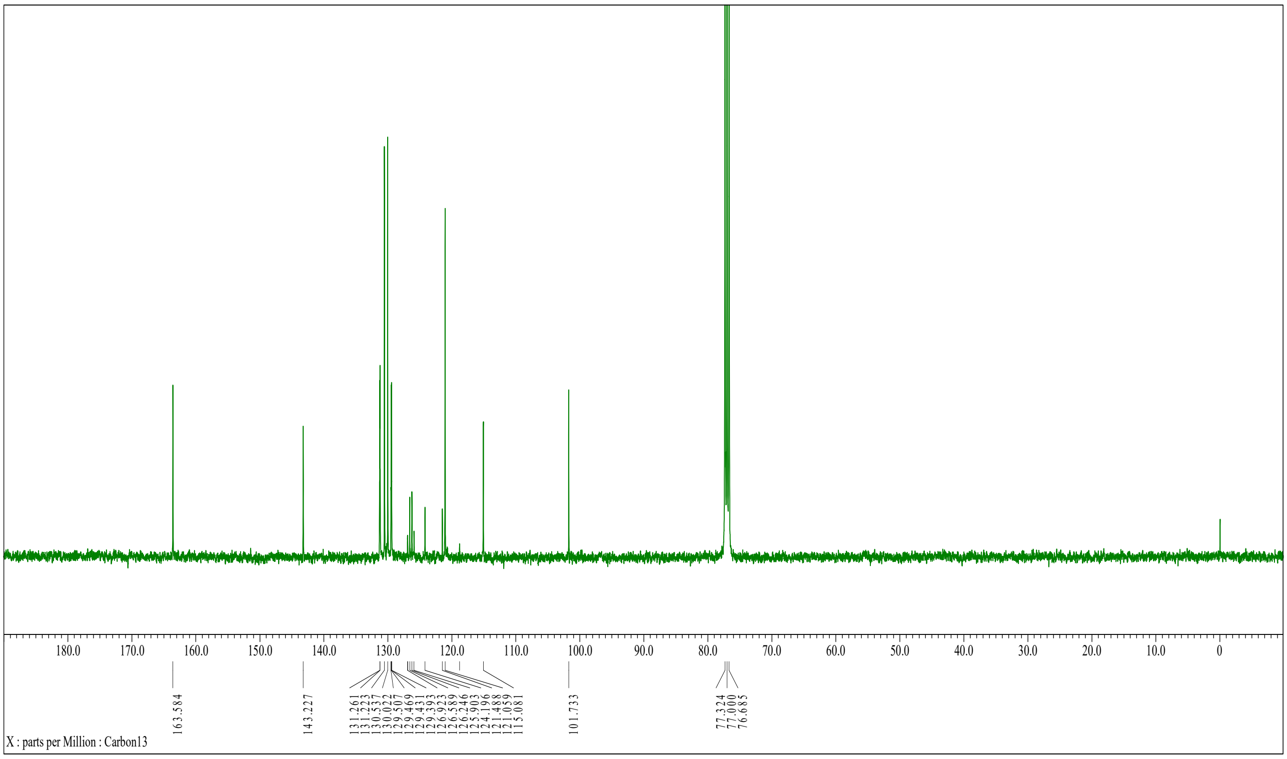

^{19}F NMR (376 MHz, $CDCl_3$) of S7d

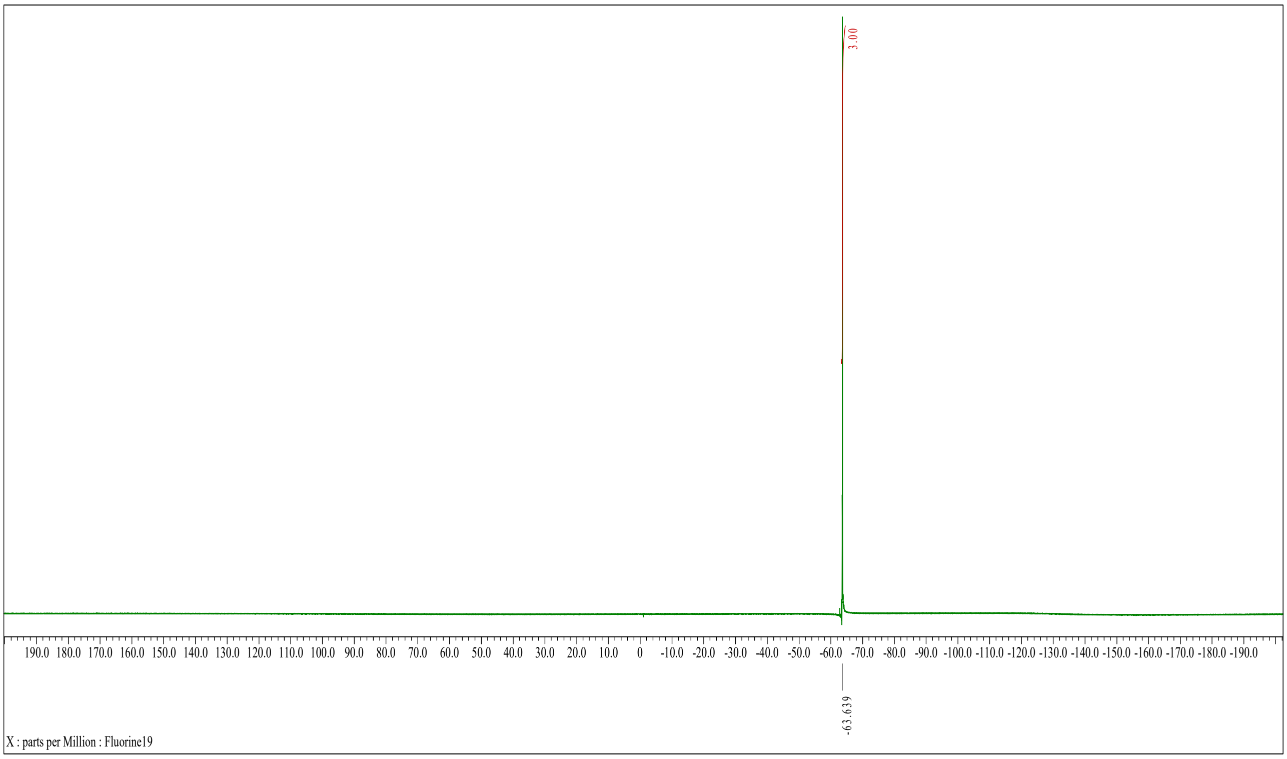

^{1}H NMR (400 MHz, $CDCl_3$) of S8d

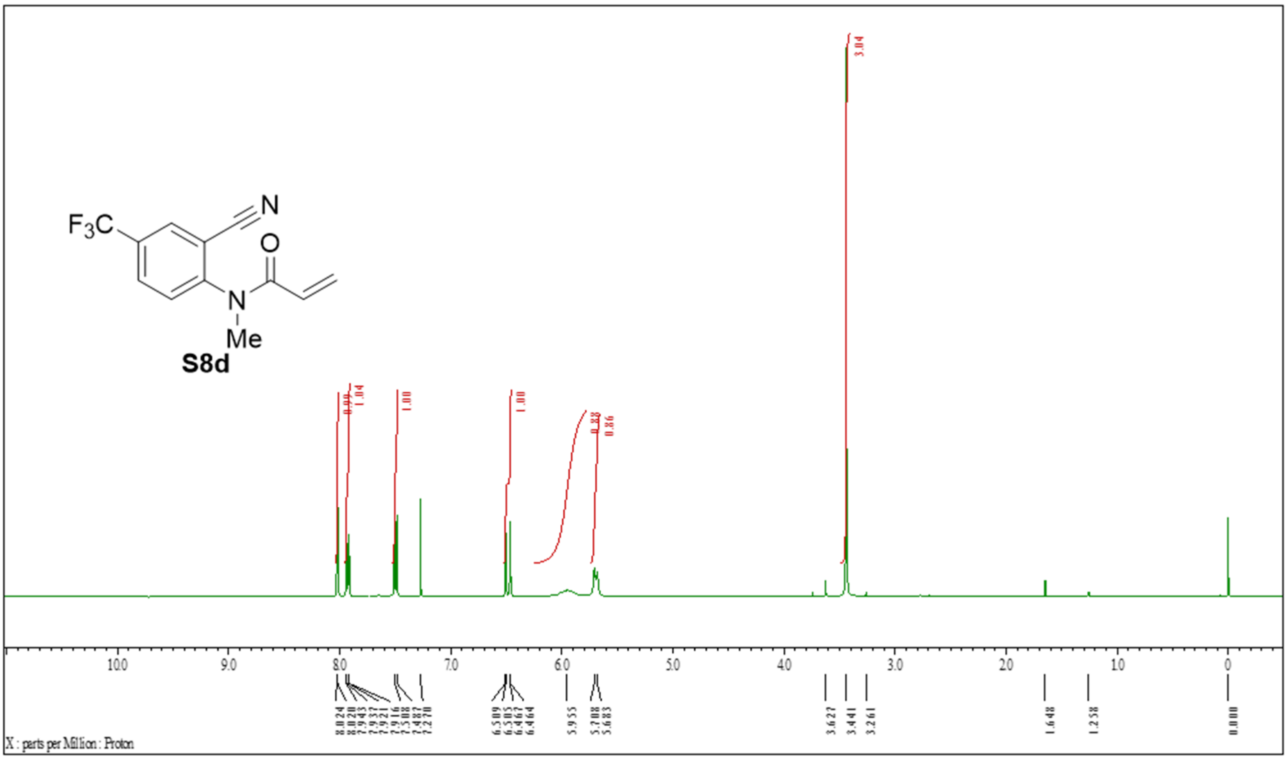

^{13}C NMR (100 MHz, $CDCl_3$) of S8d

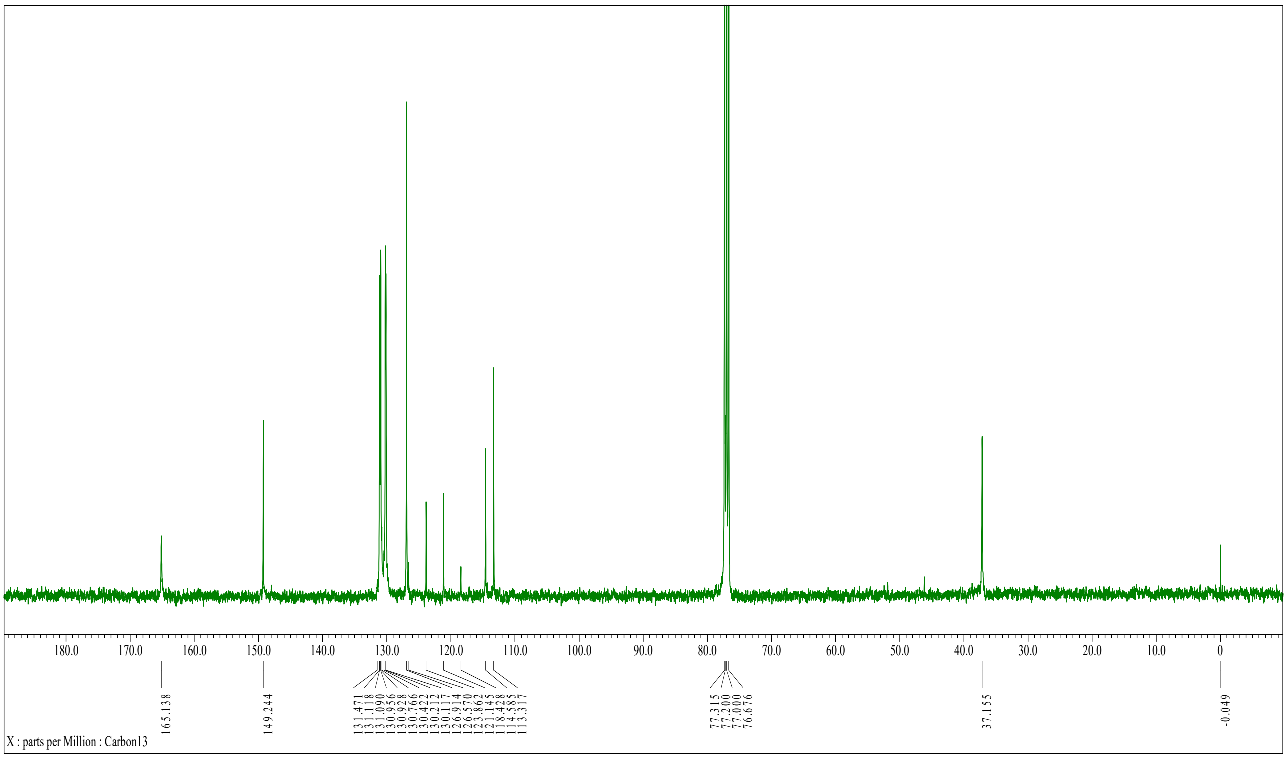

^{19}F NMR (376 MHz, $CDCl_3$) of S8d

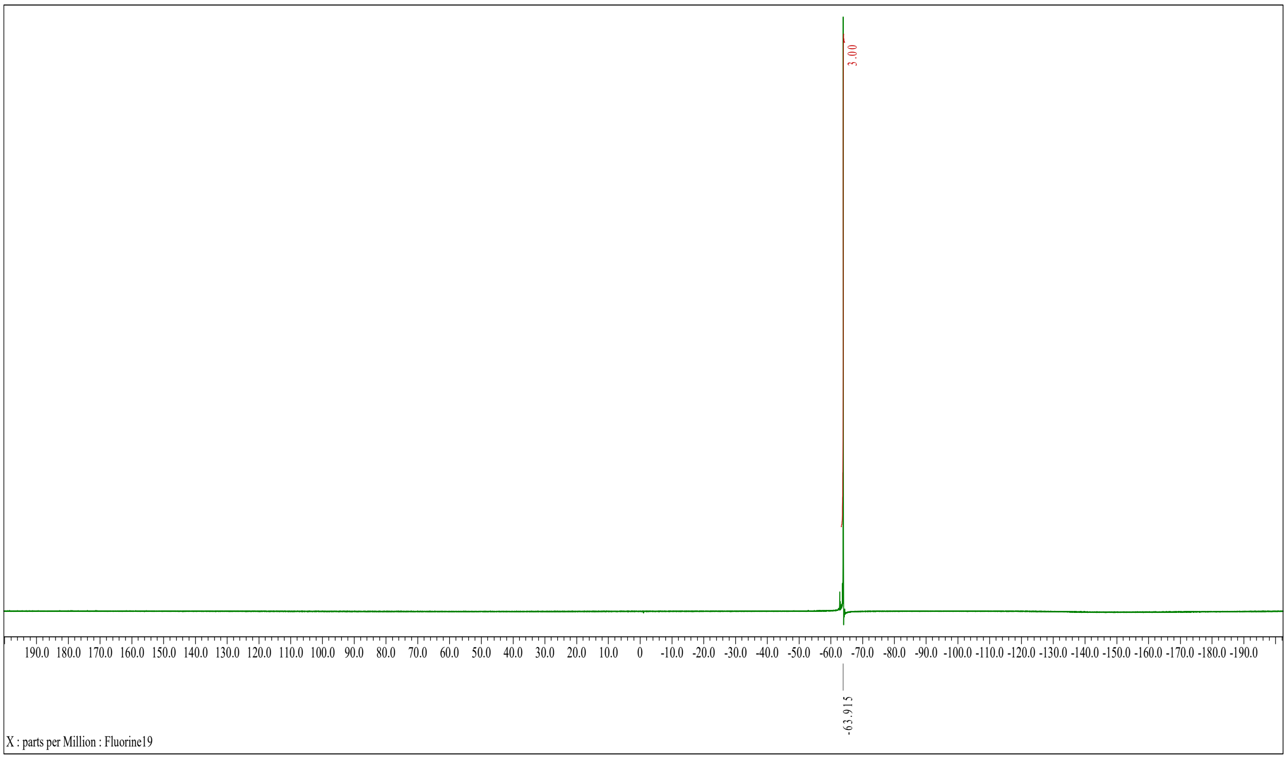

^{1}H NMR (400 MHz, DMSO-d_6) of 1d

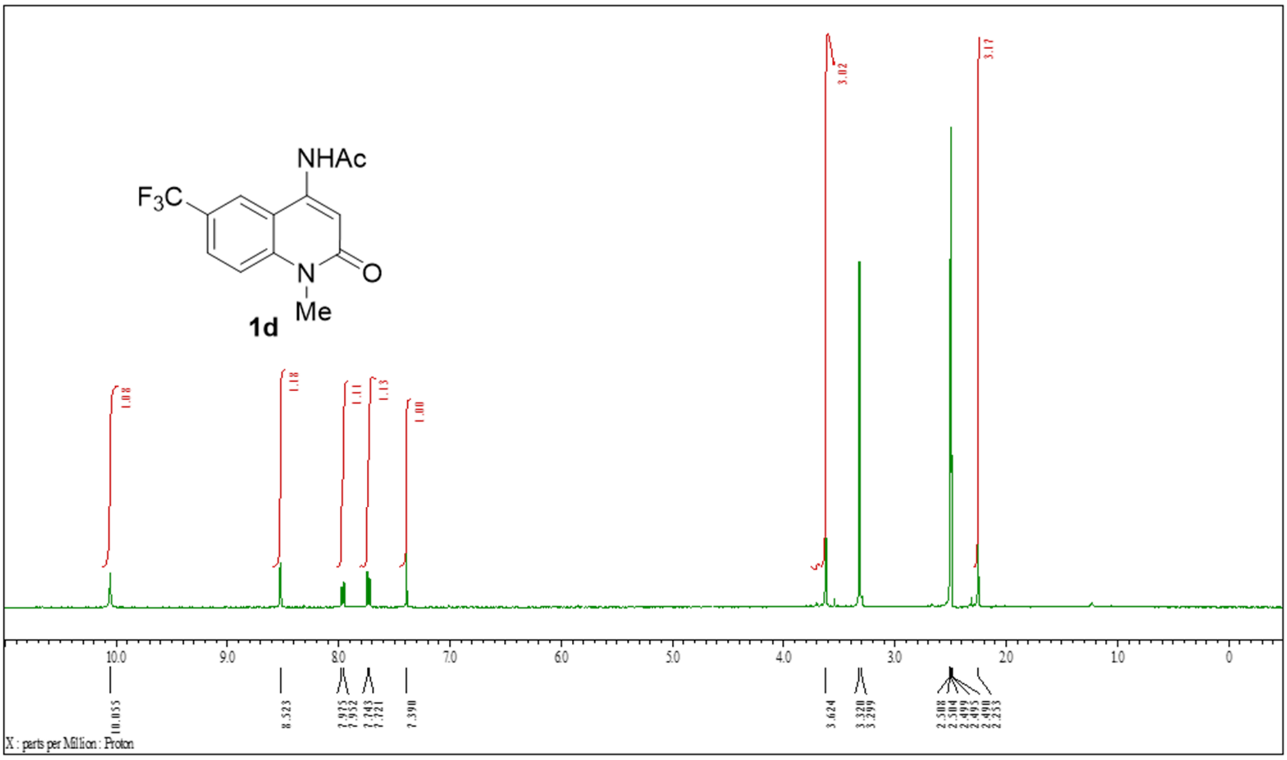

^{13}C NMR (100 MHz, DMSO-d_6) of 1d

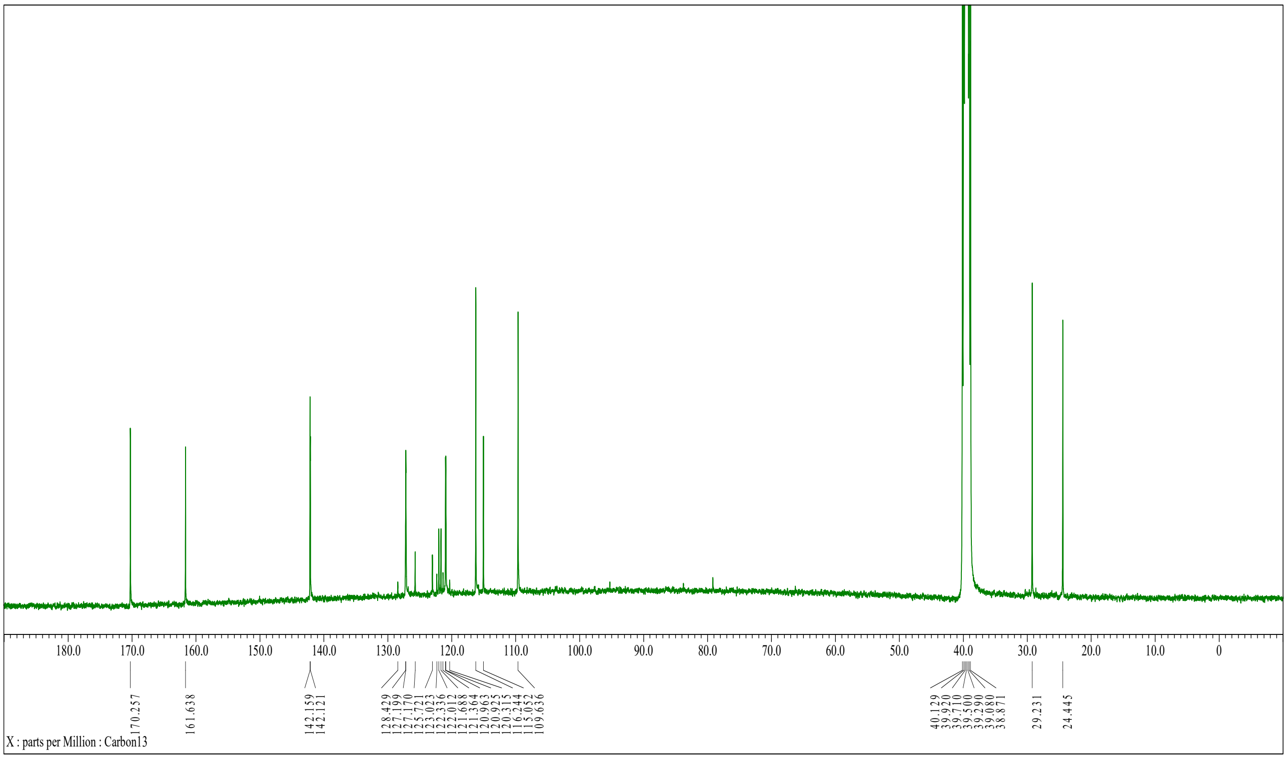

^{19}F NMR (376 MHz, $CDCl_3$) of 1d

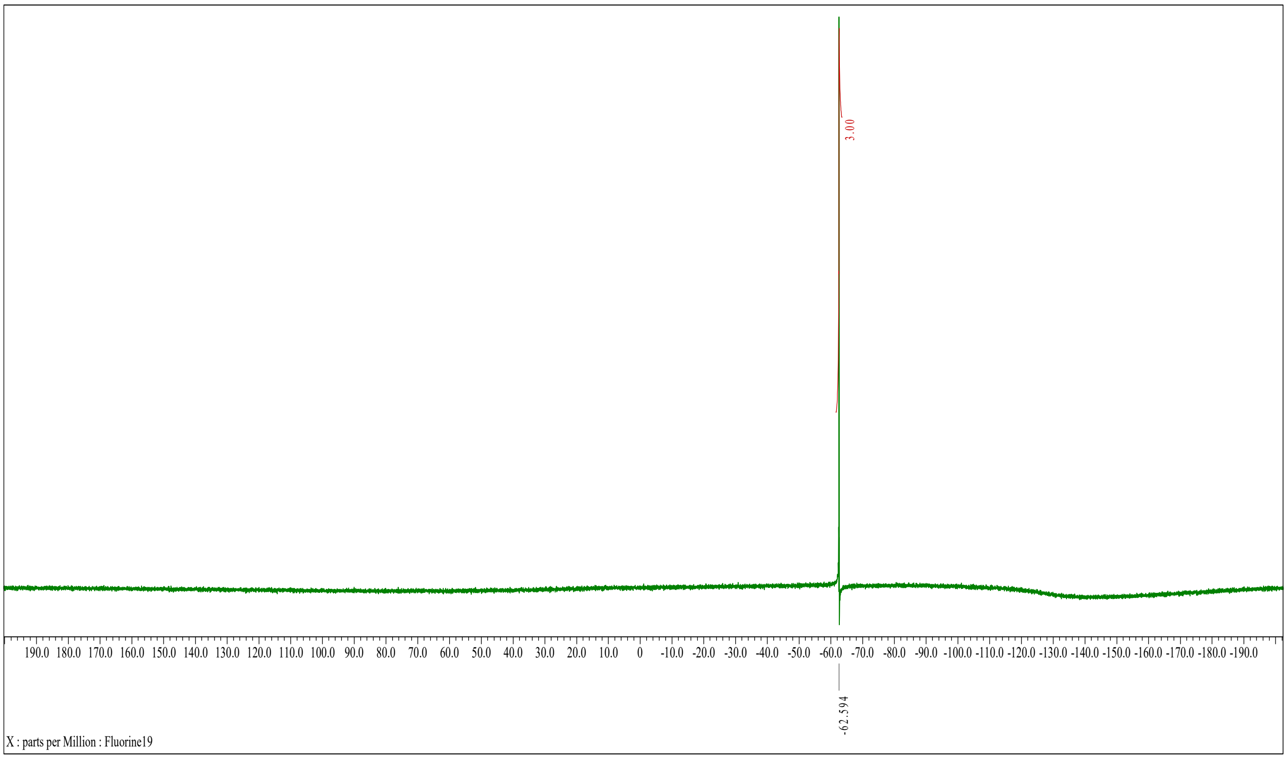

^{1}H NMR (400 MHz, $CDCl_3$) of S7e

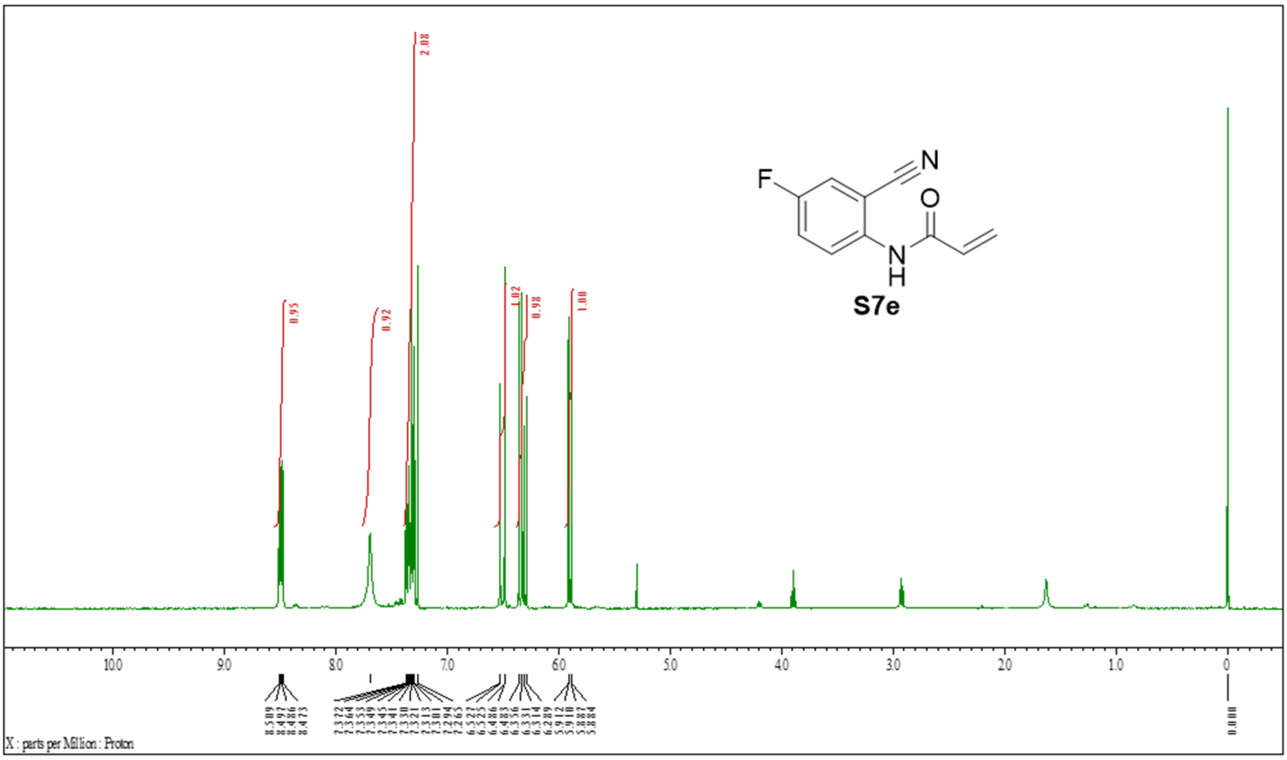

^{13}C NMR (100 MHz, $CDCl_3$) of S7e

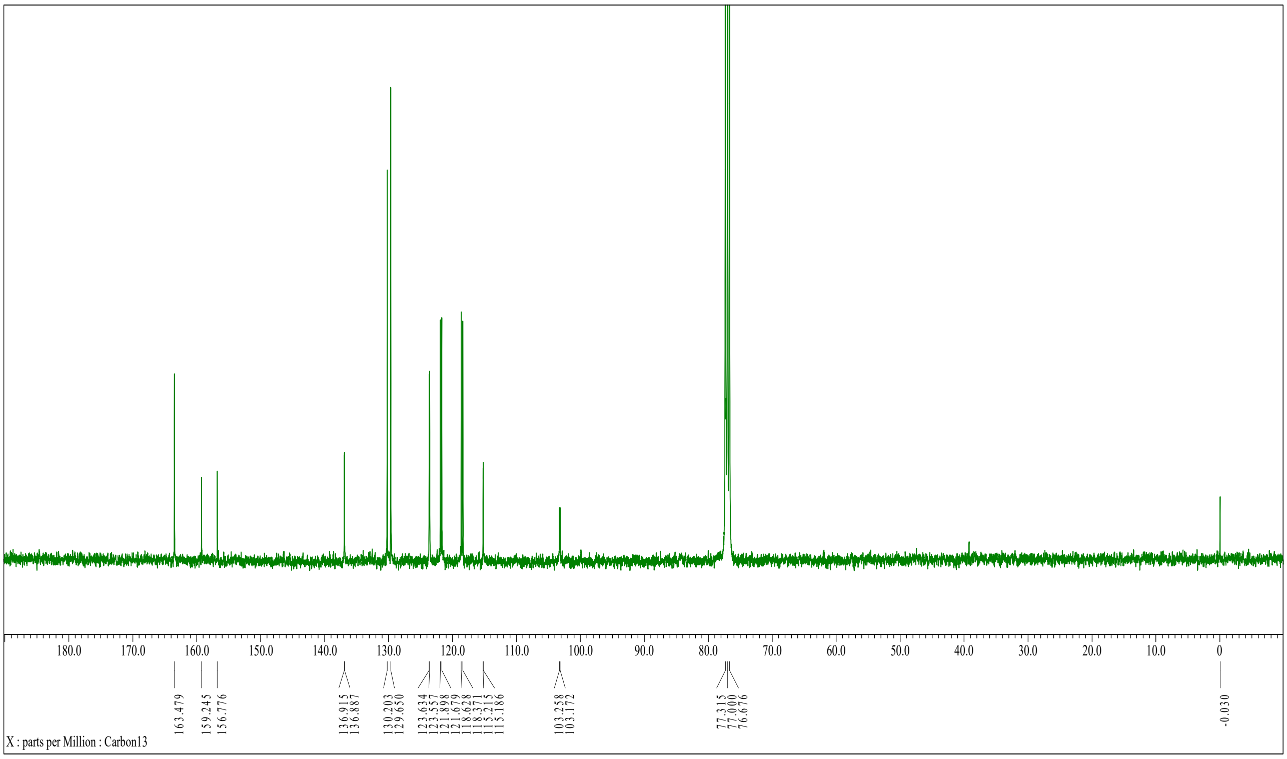

^{19}F NMR (376 MHz, $CDCl_3$) of S7e

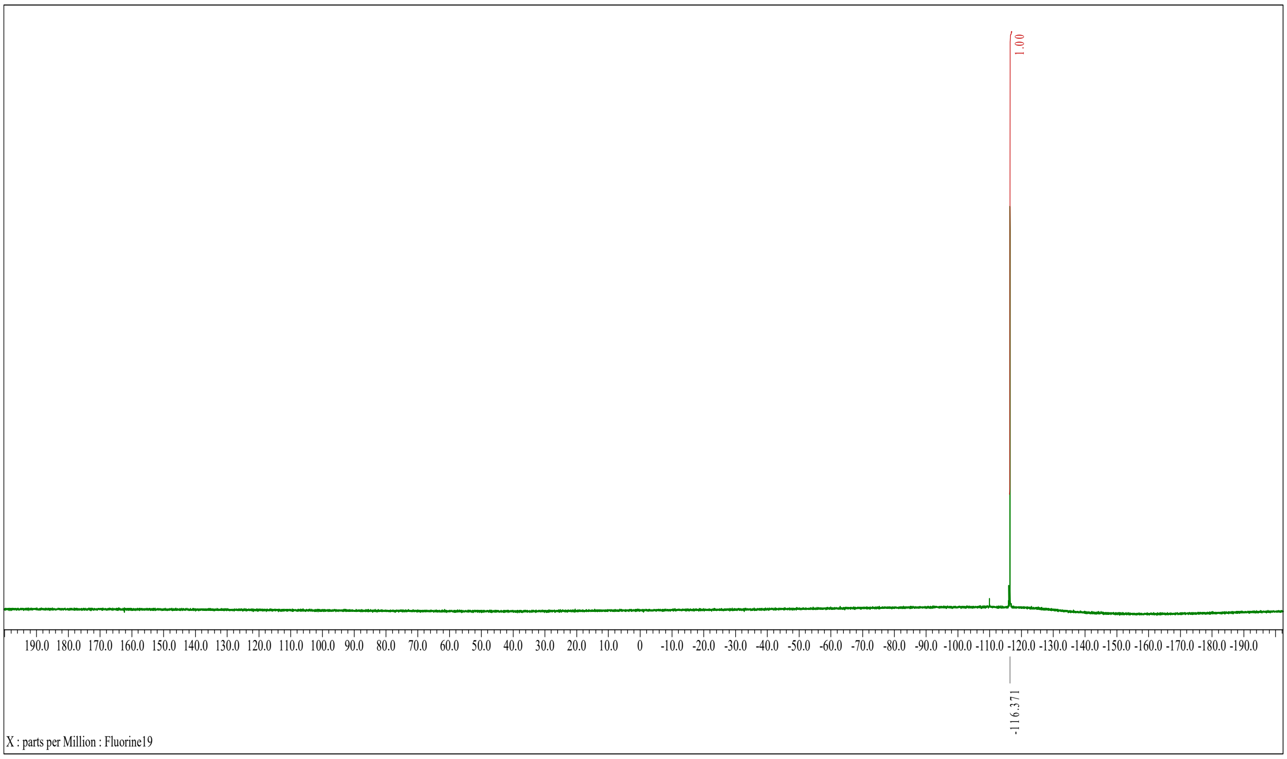

^{1}H NMR (400 MHz, $CDCl_3$) of S8e

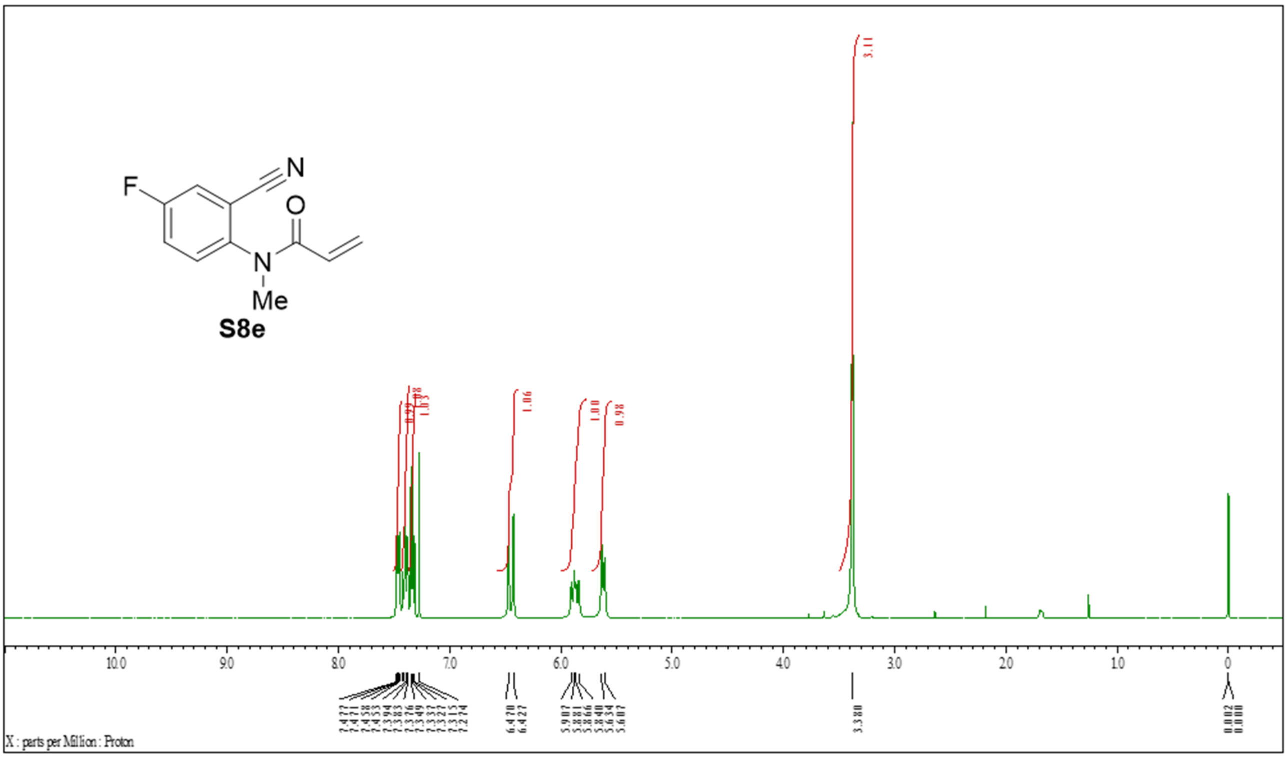

^{13}C NMR (100 MHz, $CDCl_3$) of S8e

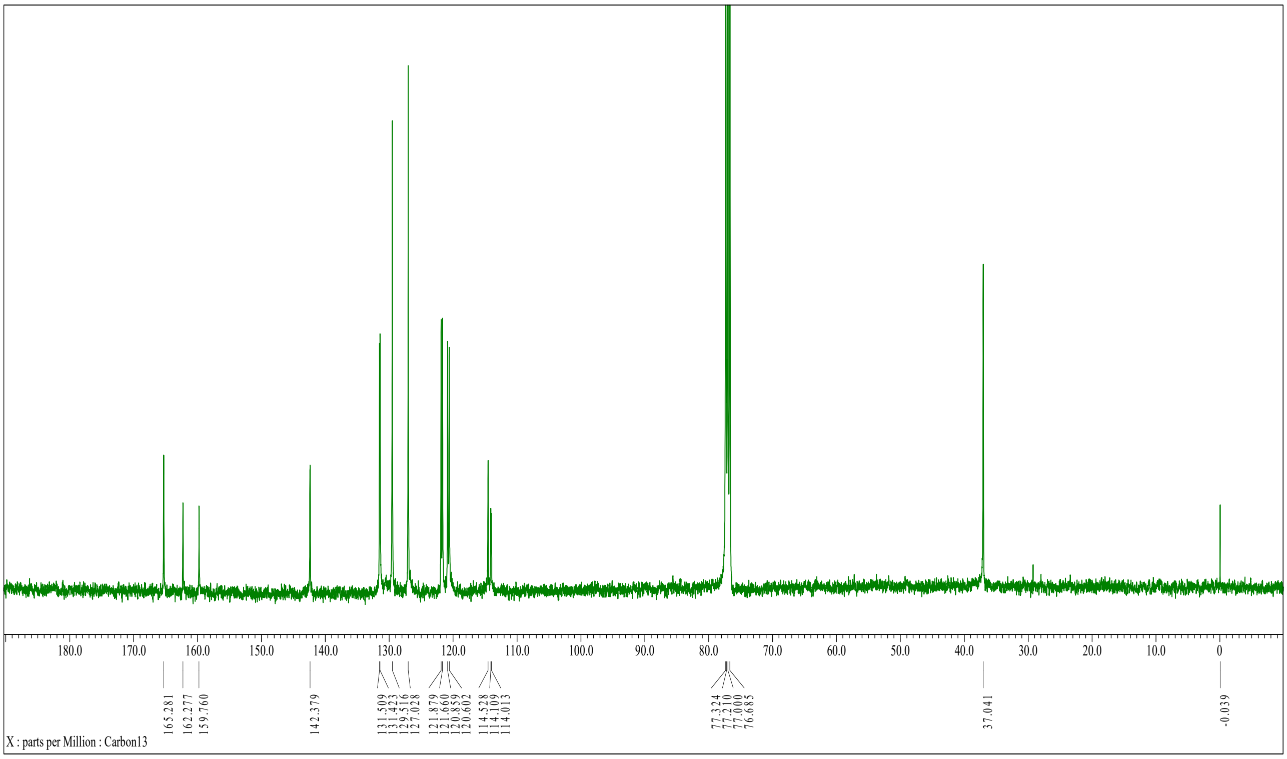

^{19}F NMR (376 MHz, $CDCl_3$) of S8e

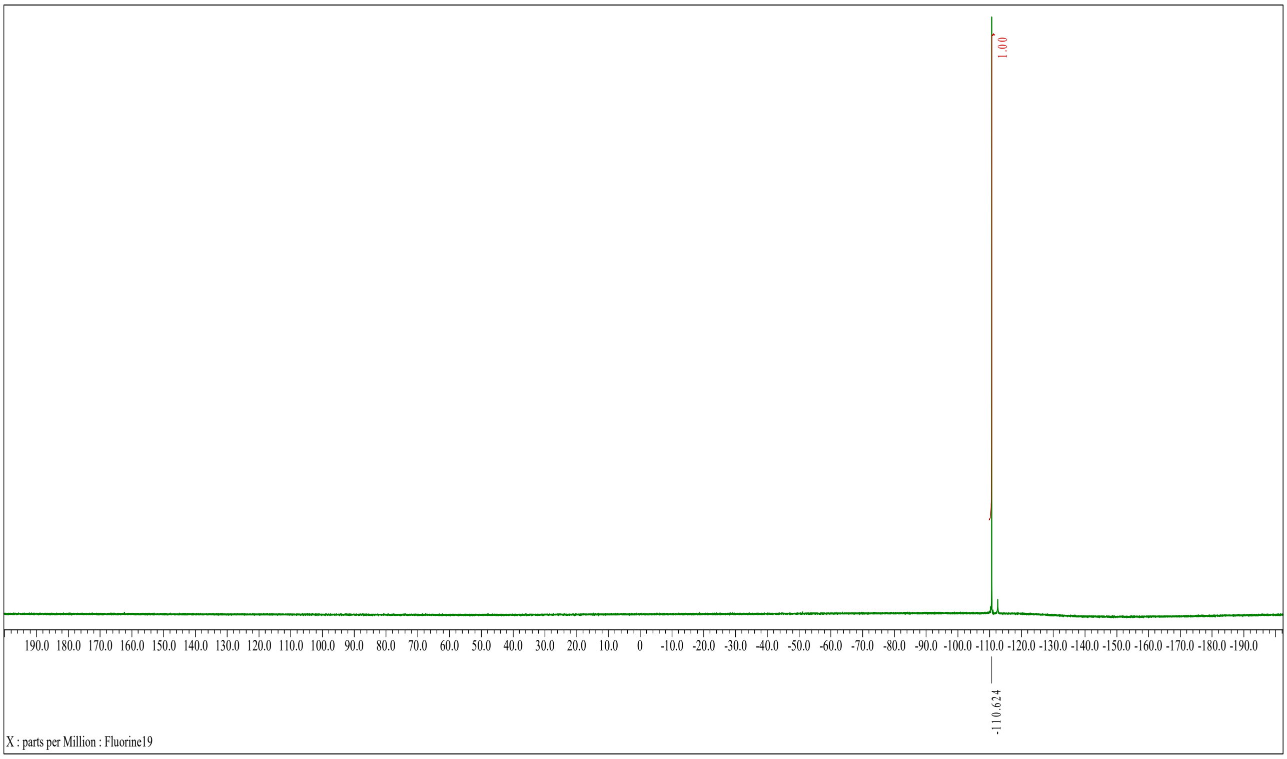

^{1}H NMR (400 MHz, DMSO-d_6) of 1e

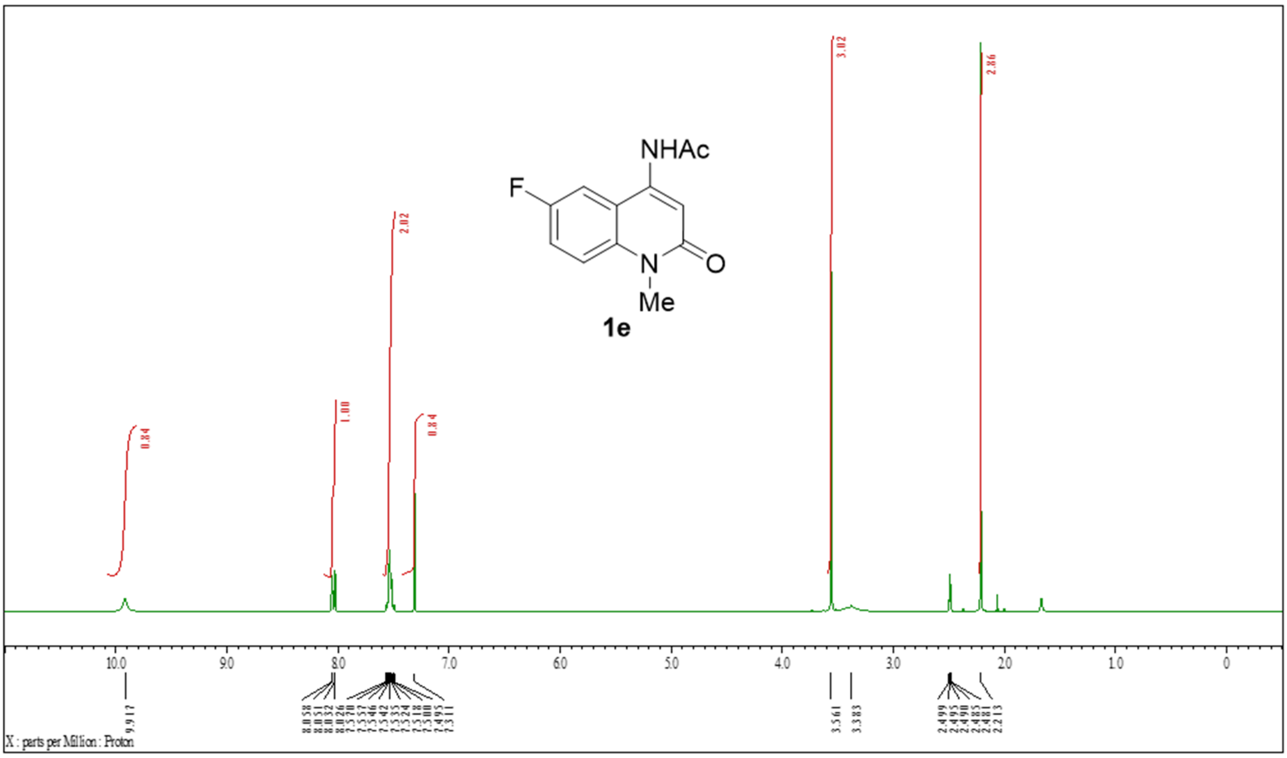

^{13}C NMR (100 MHz, DMSO-d_6) of 1e

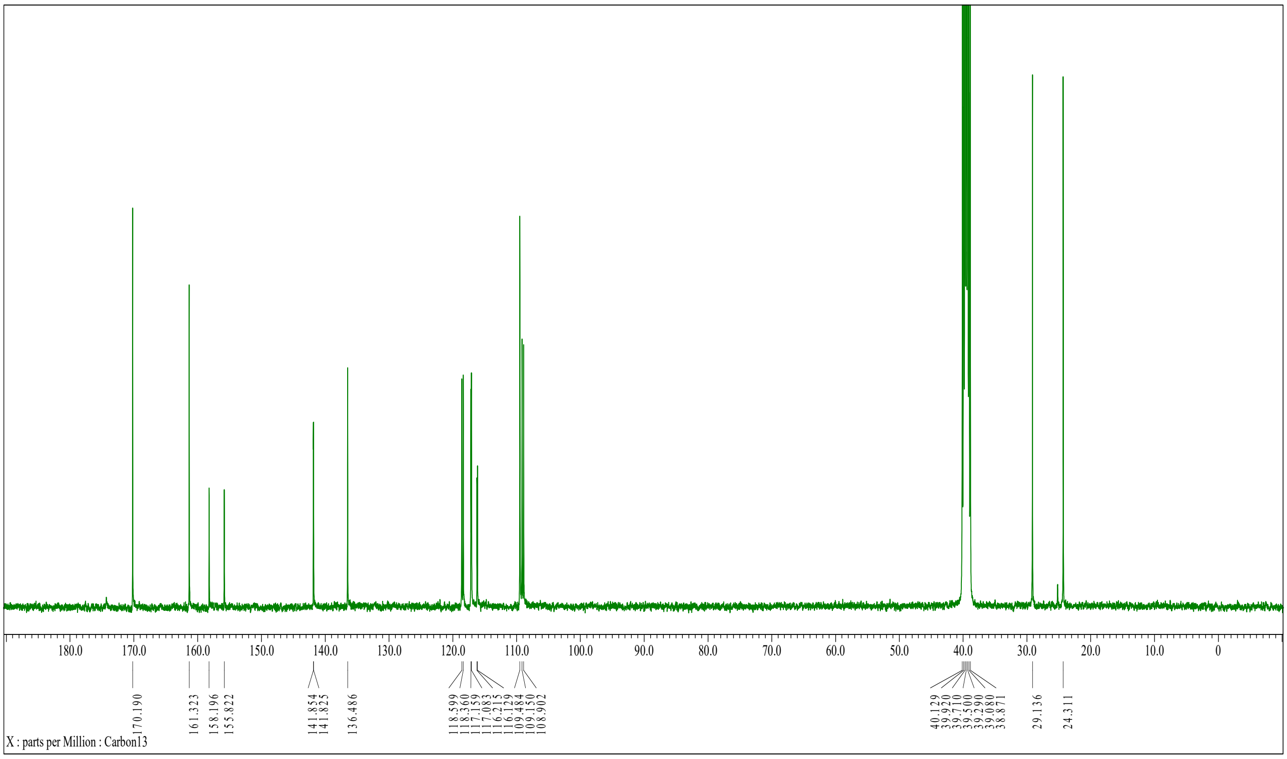

^{19}F NMR (376 MHz, $CDCl_3$) of 1e

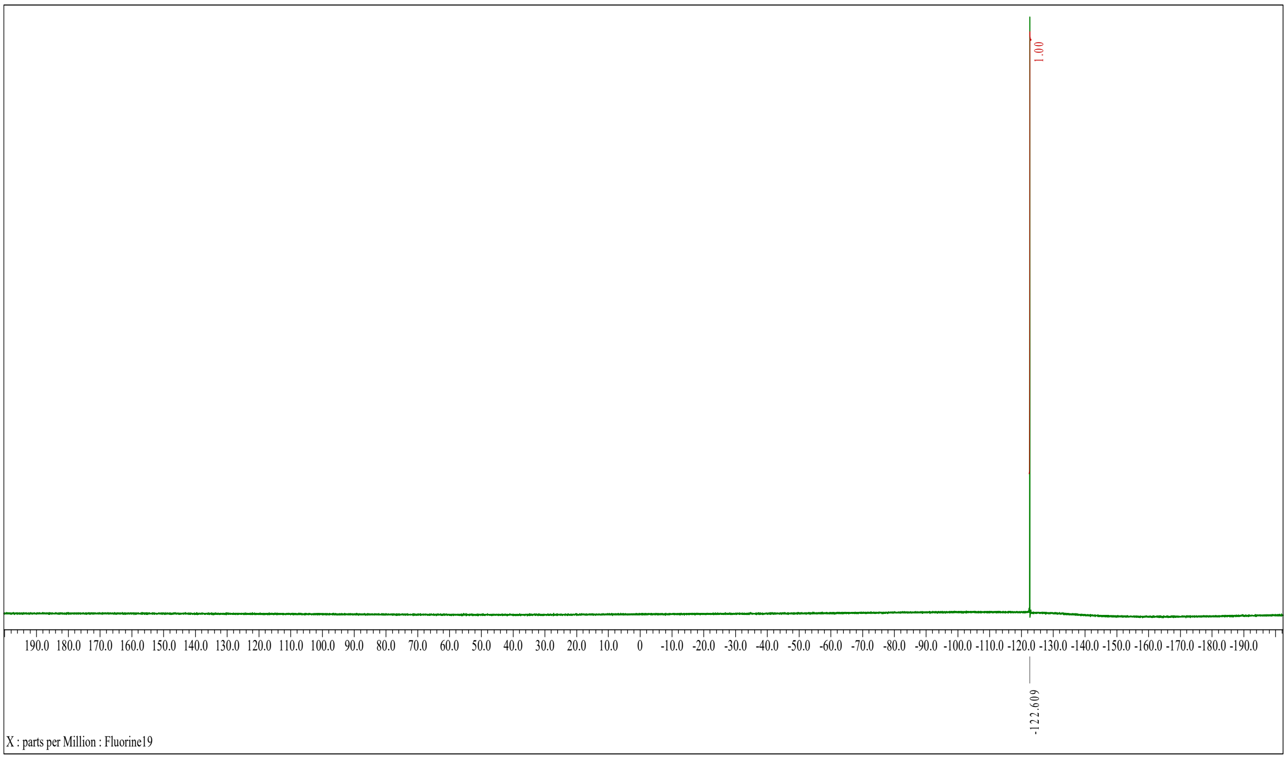

^{1}H NMR (400 MHz, $CDCl_3$) of S7f

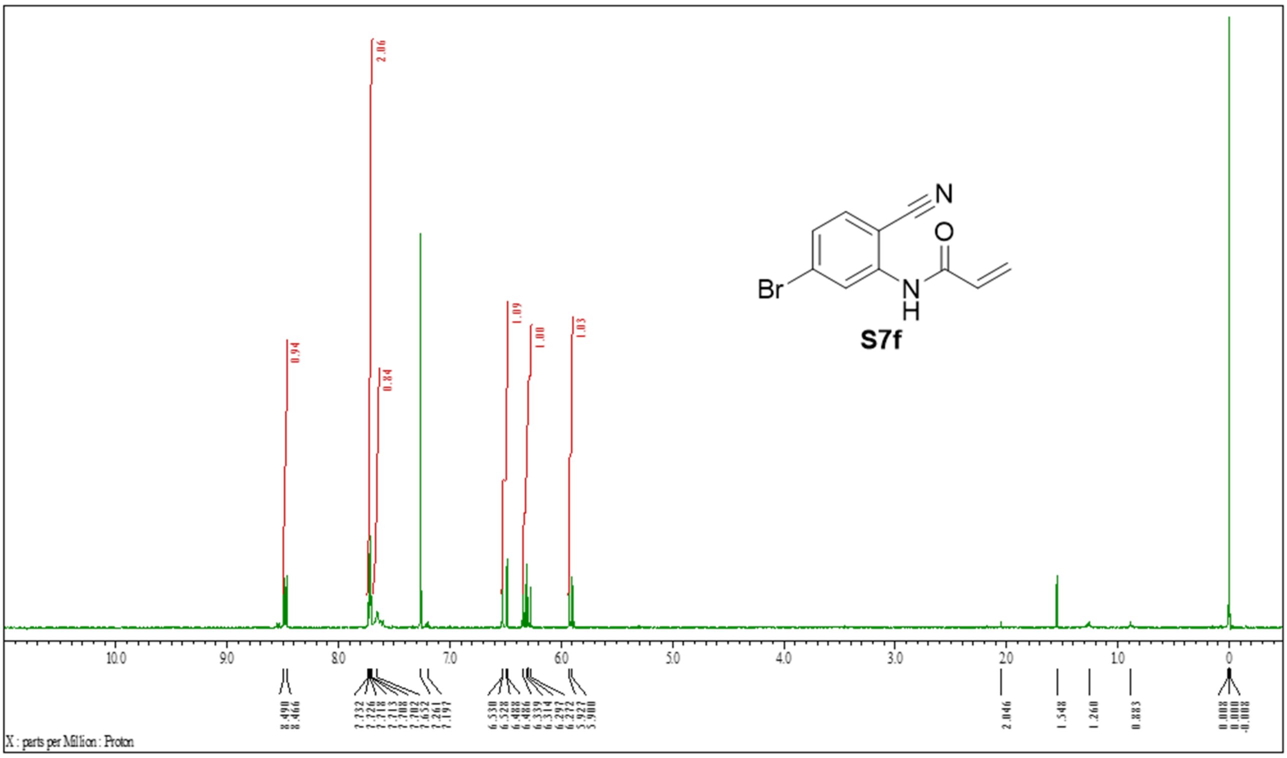

^{13}C NMR (100 MHz, $CDCl_3$) of S7f

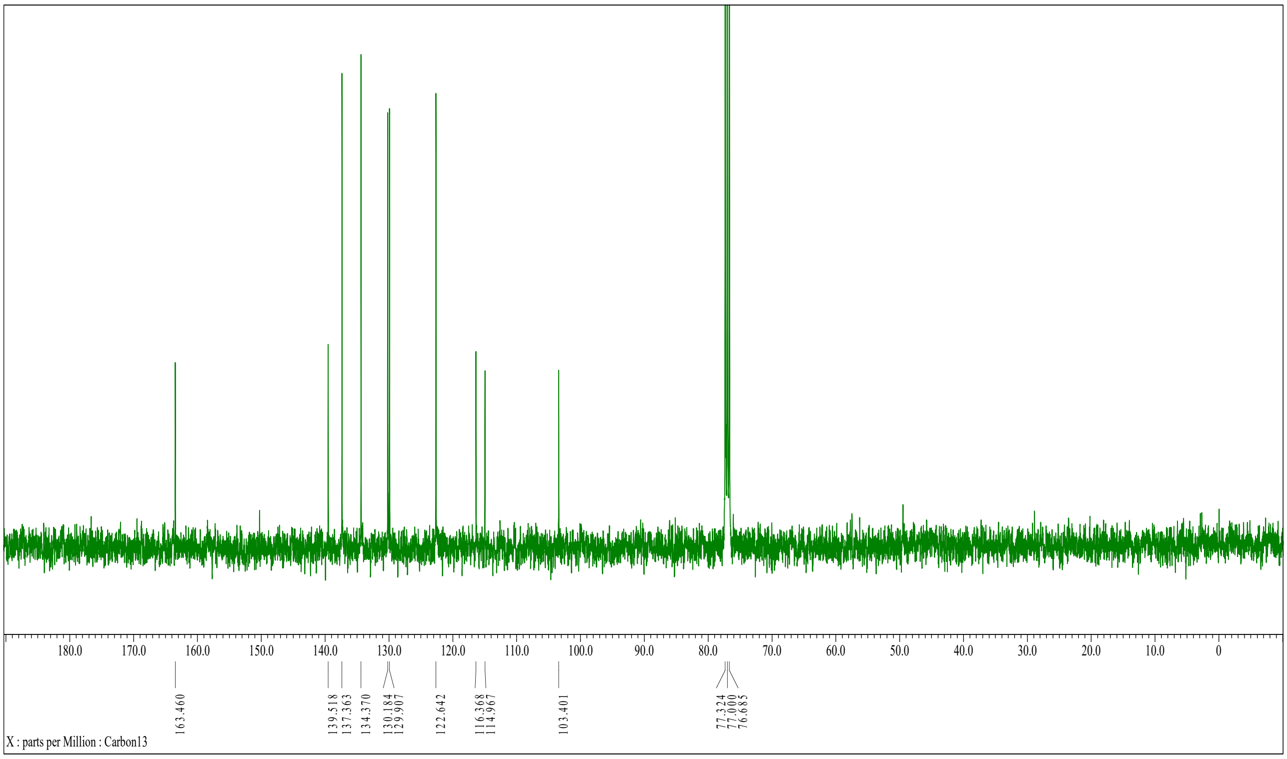

^{1}H NMR (400 MHz, $CDCl_3$) of S8f

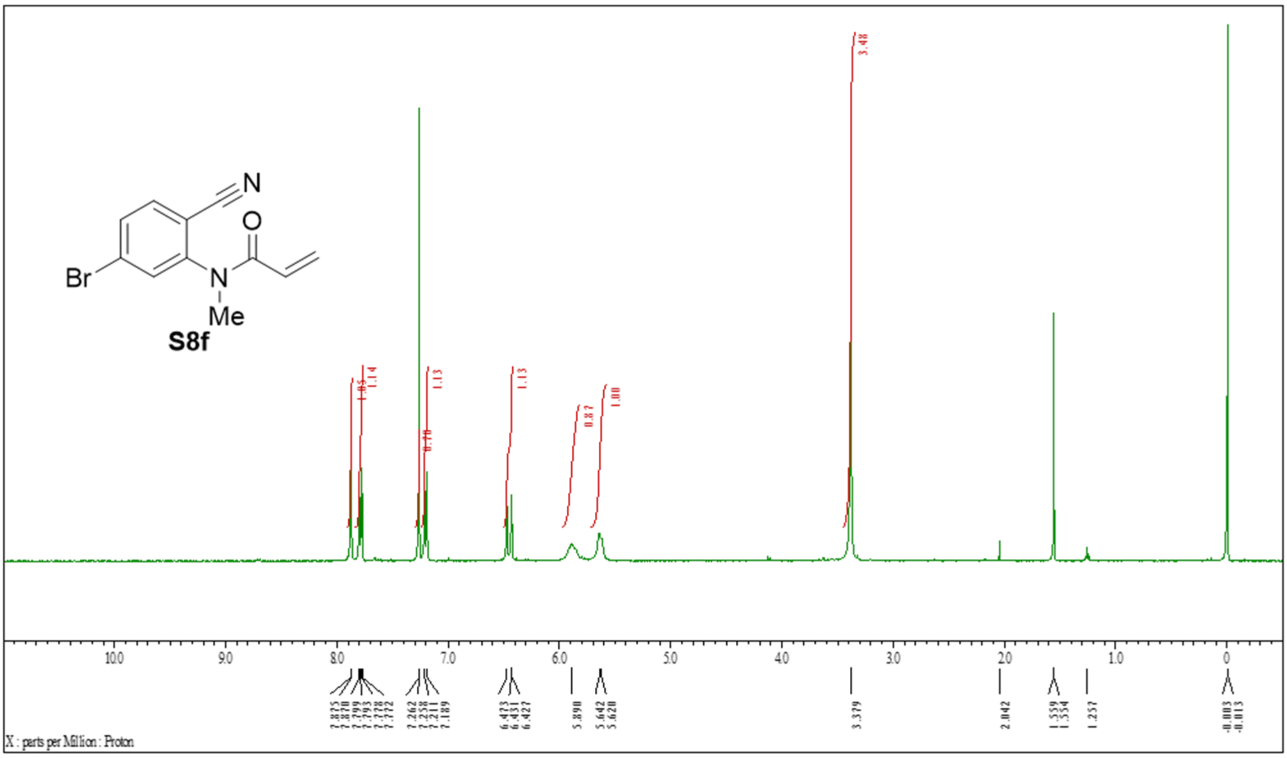

^{13}C NMR (100 MHz, $CDCl_3$) of S8f

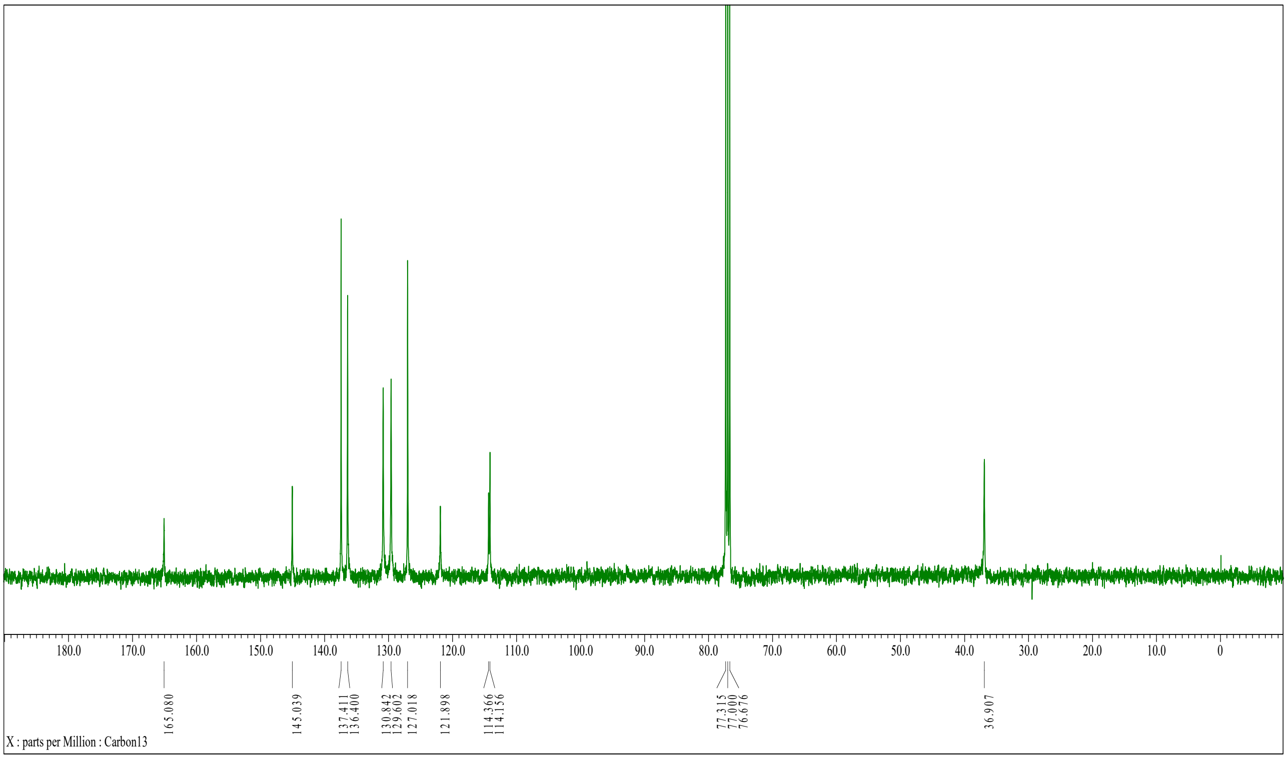

^{1}H NMR (400 MHz, DMSO-d_6) of 1f

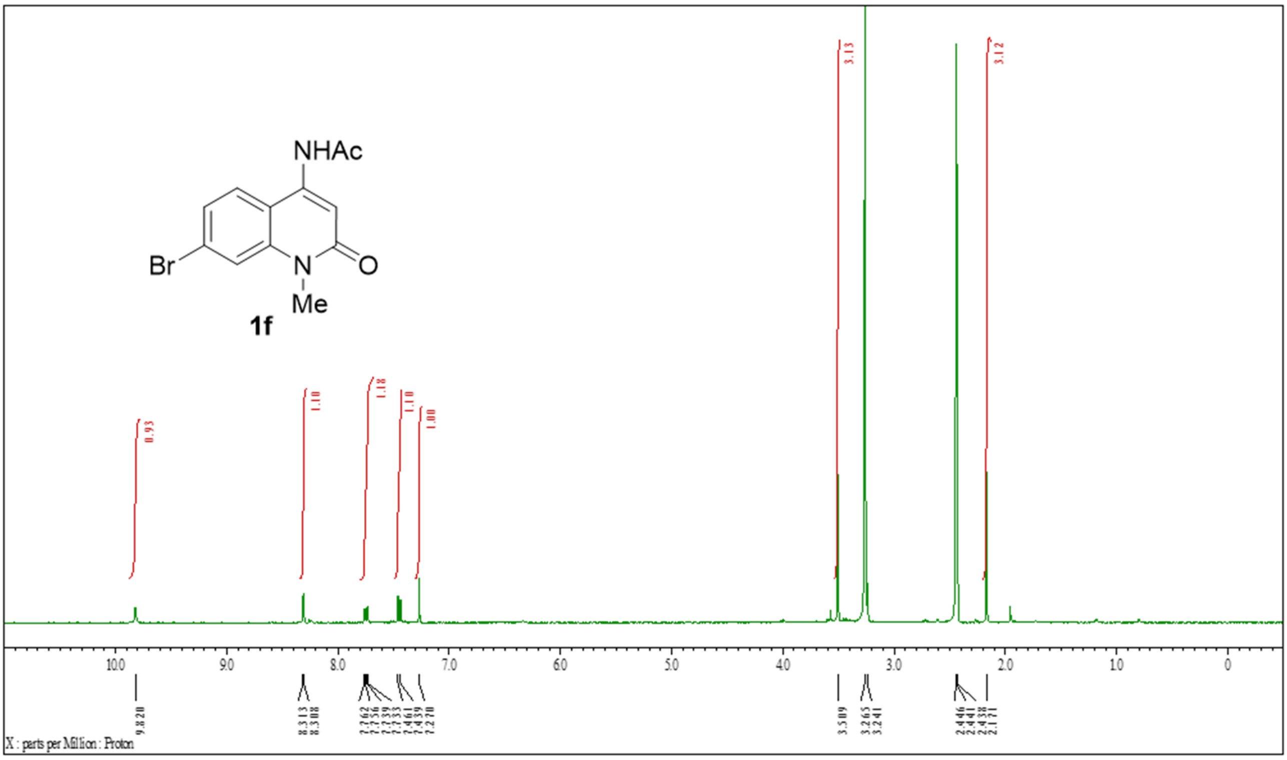

^{13}C NMR (100 MHz, DMSO-d_6) of 1e

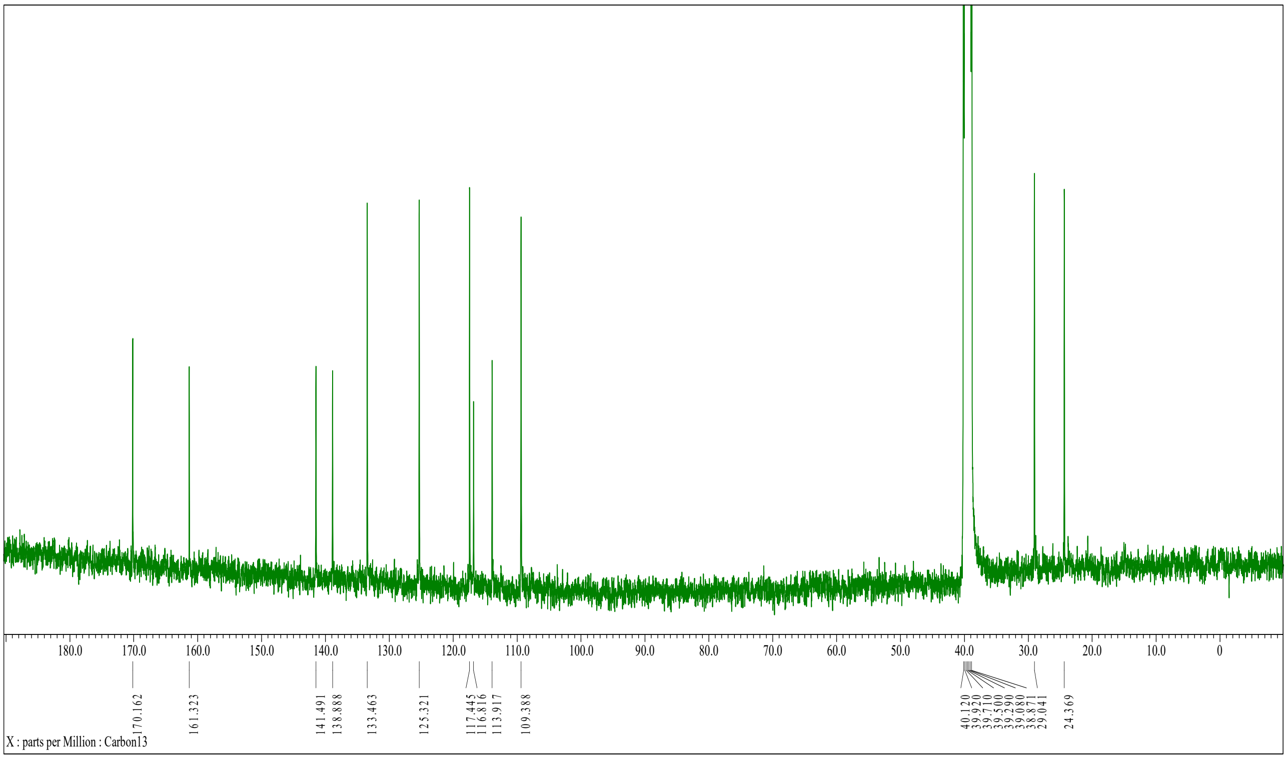

^{1}H NMR (400 MHz, $CDCl_3$) of S7g

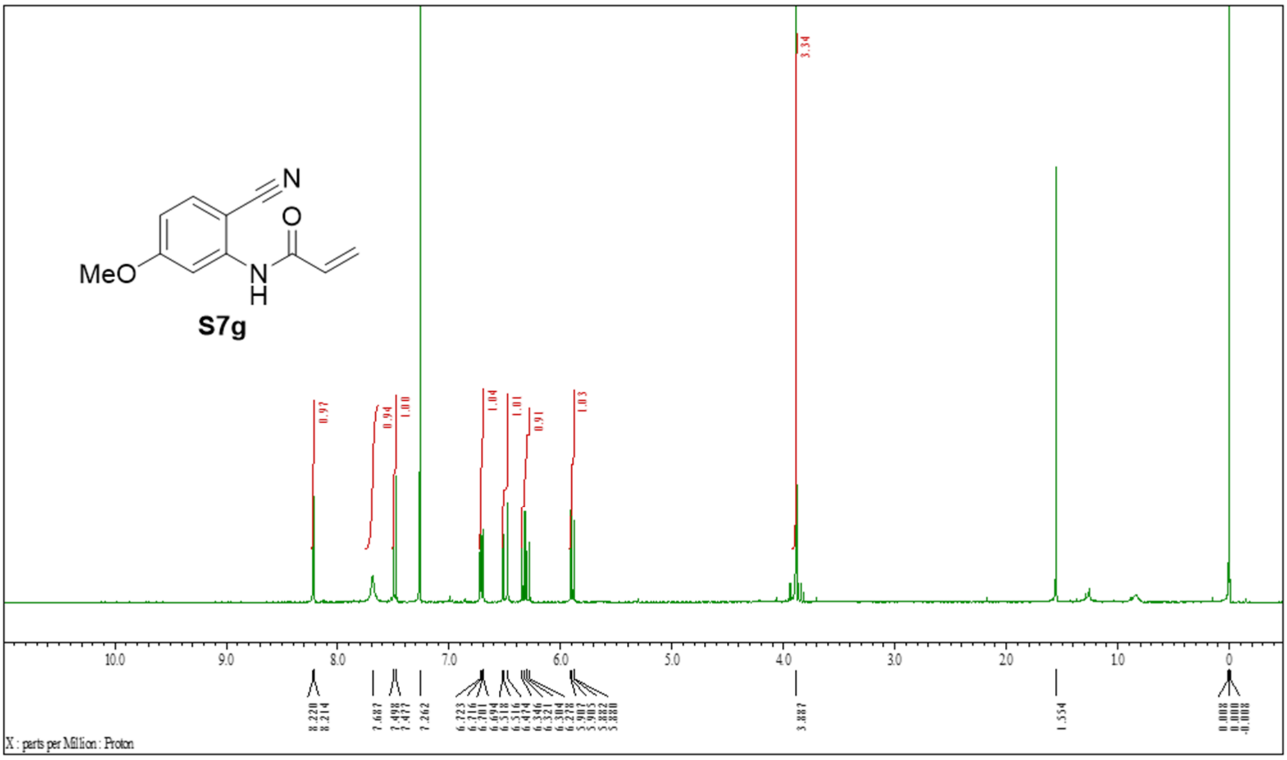

^{13}C NMR (100 MHz, $CDCl_3$) of S7g

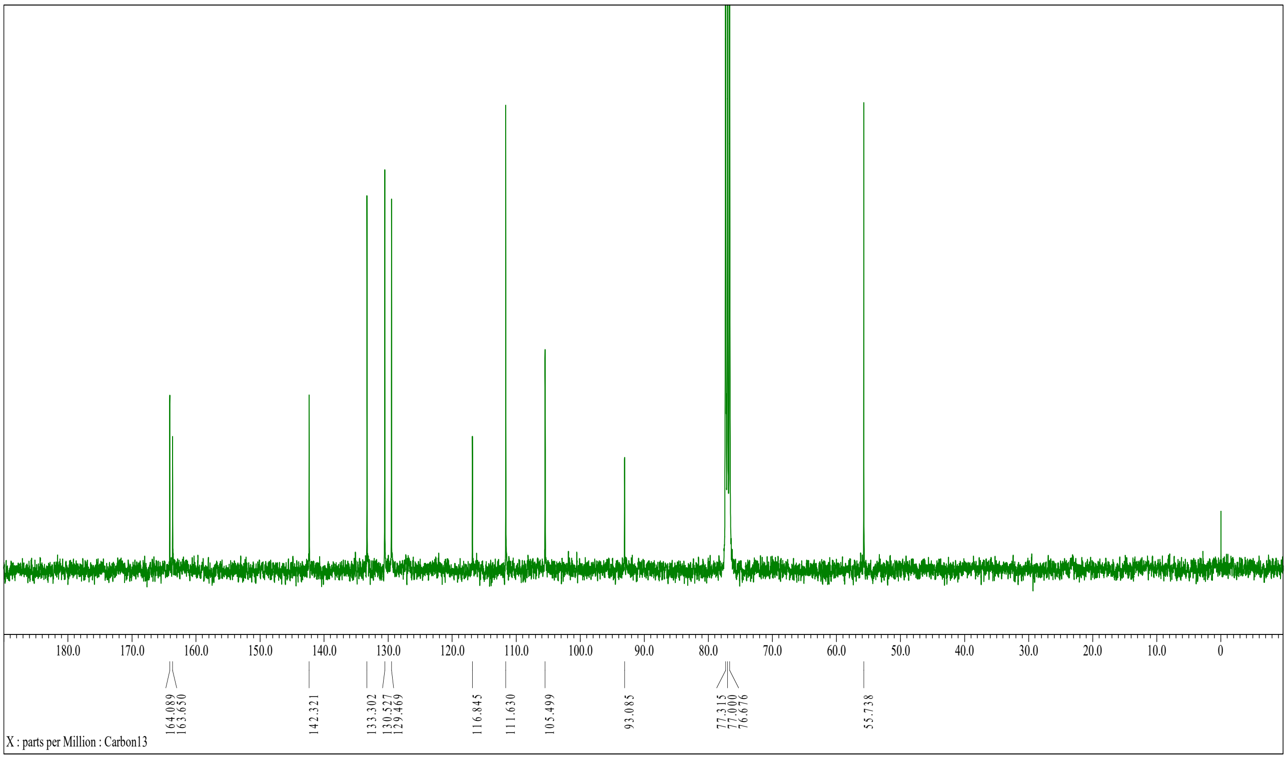

^{1}H NMR (400 MHz, $CDCl_3$) of S8g

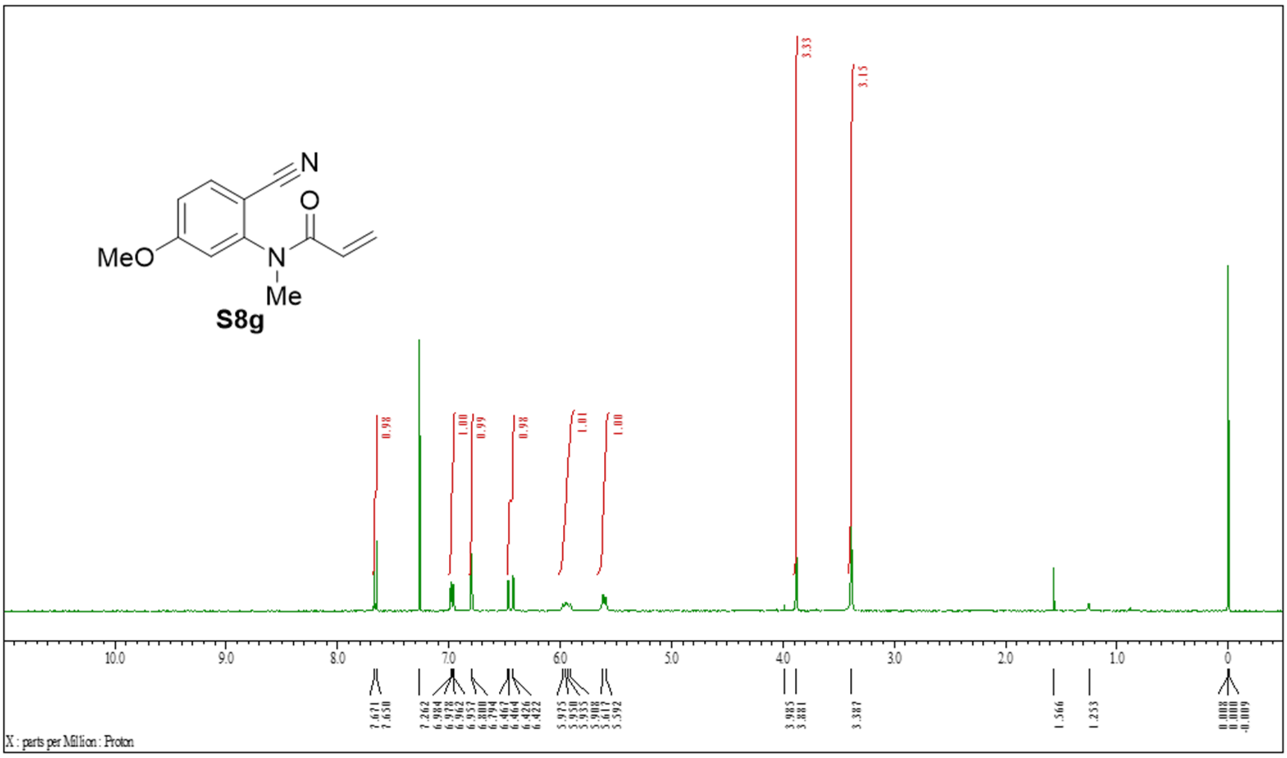

^{13}C NMR (100 MHz, $CDCl_3$) of S8g

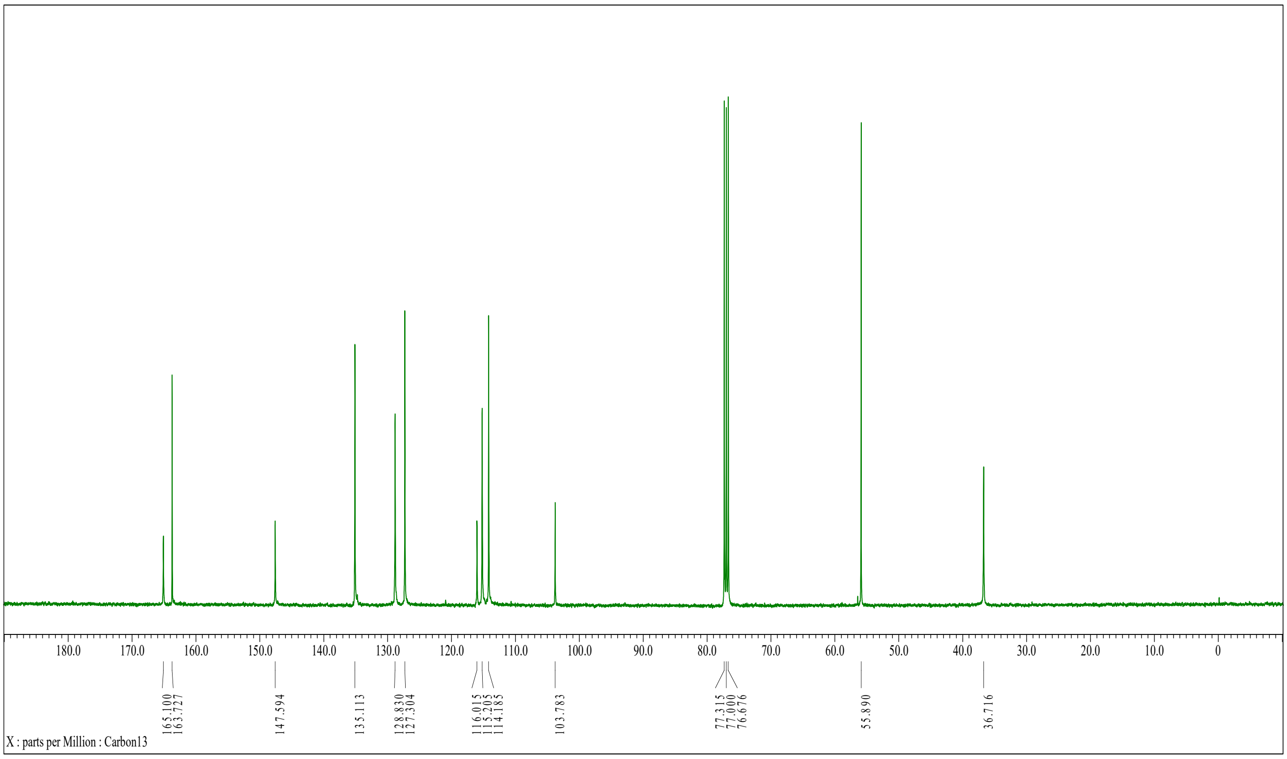

^{1}H NMR (400 MHz, DMSO-d_6) of 1g

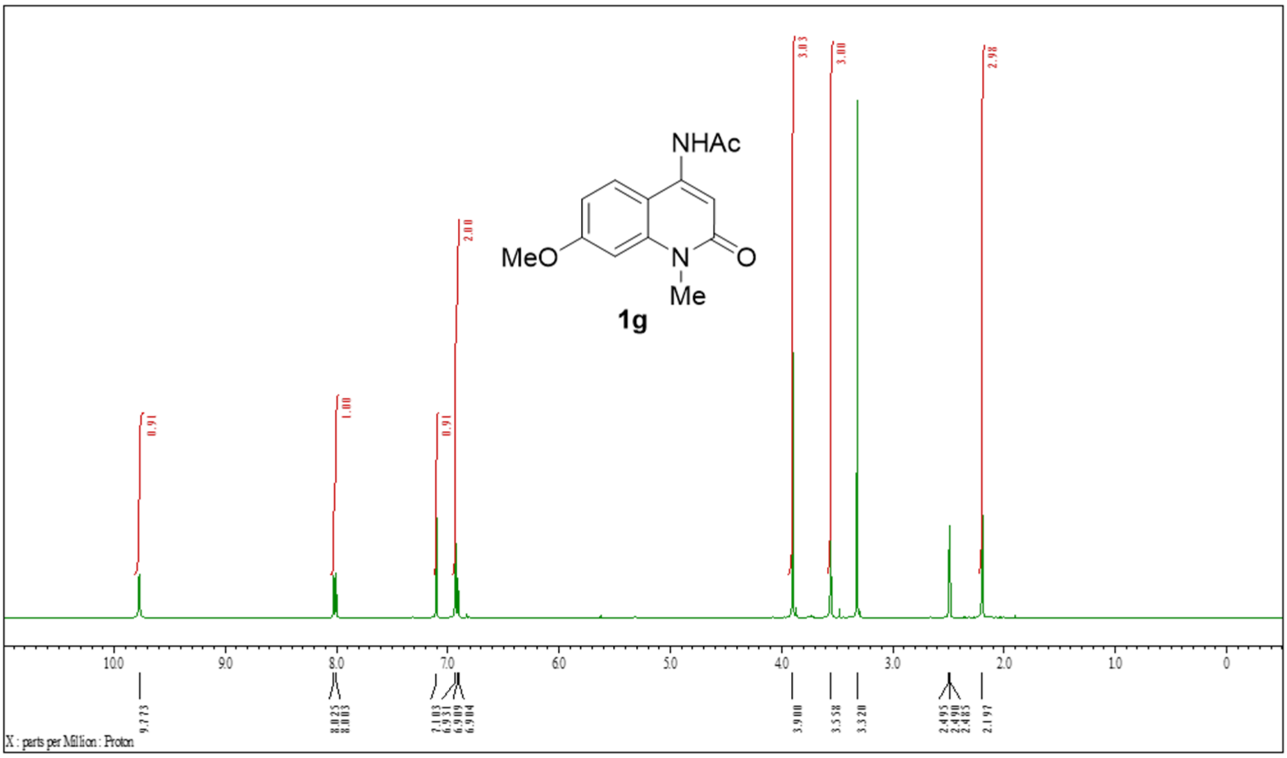

^{13}C NMR (100 MHz, DMSO-d_6) of 1g

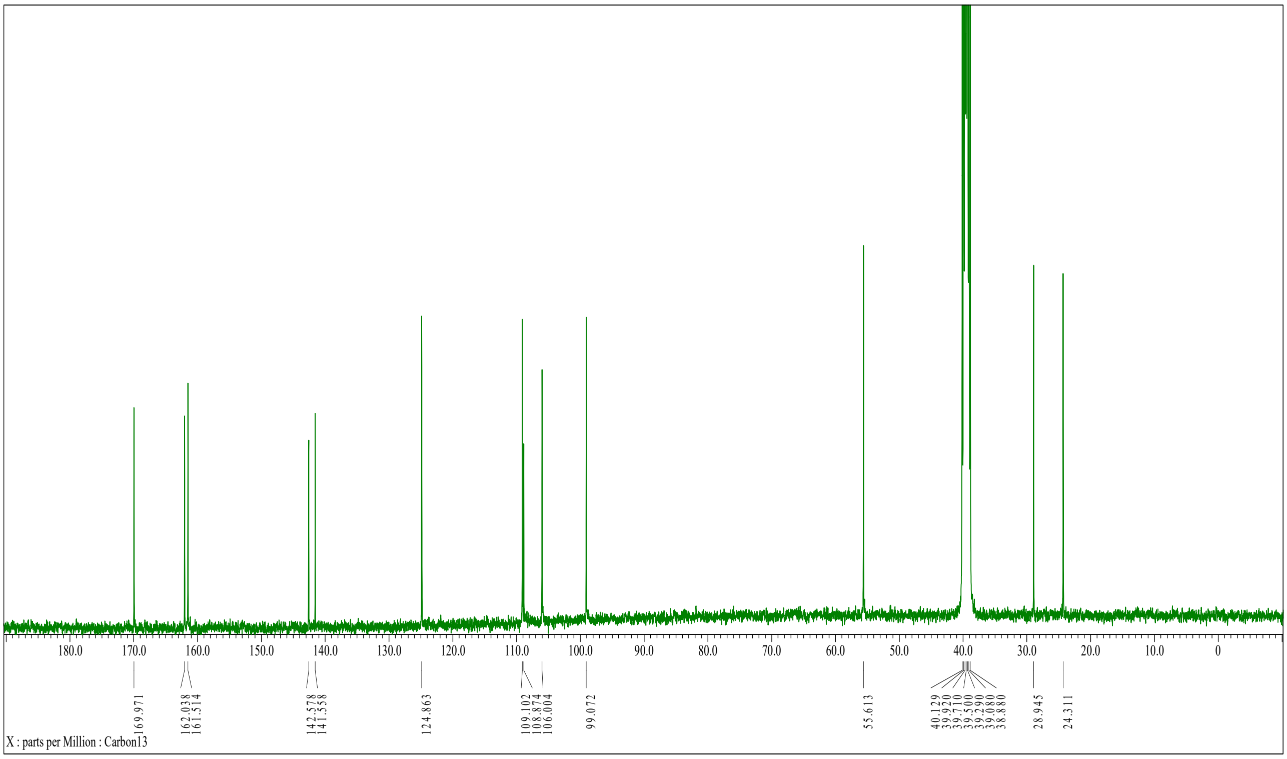

^{1}H NMR (400 MHz, DMSO-d_6) of S10

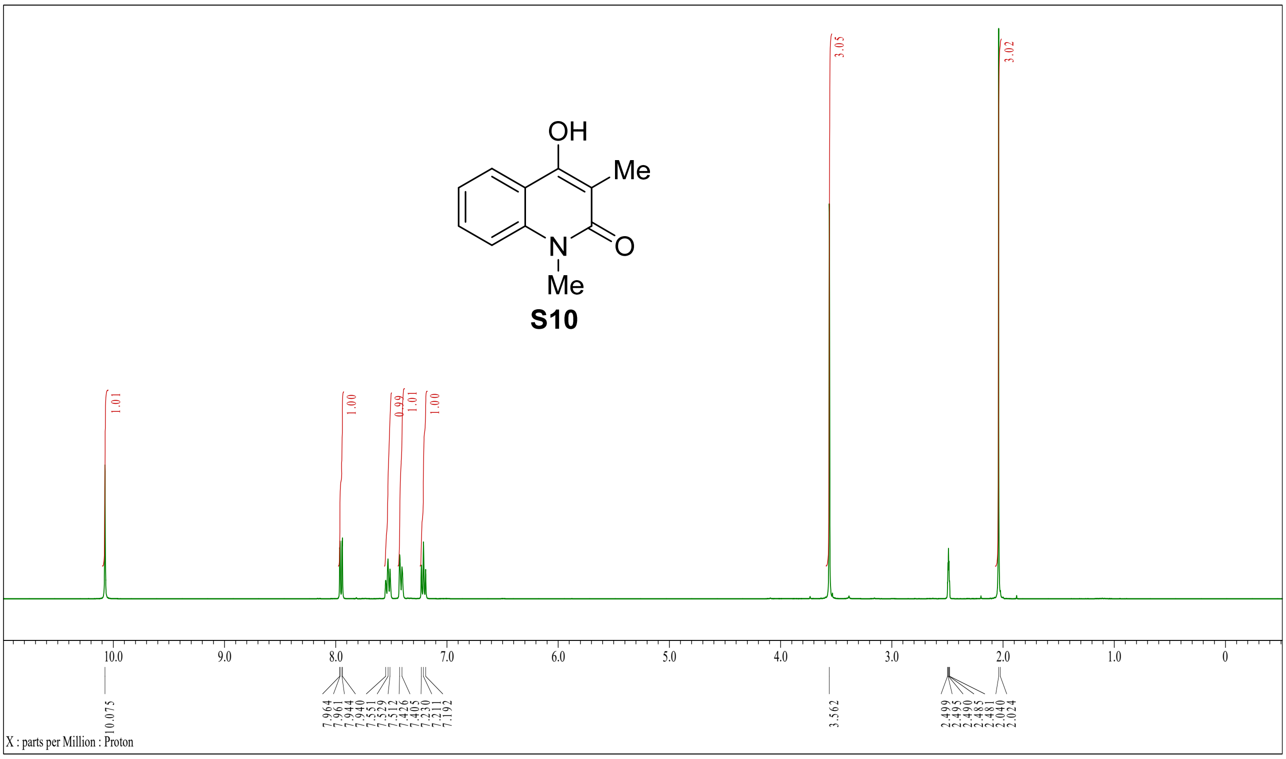

^{13}C NMR (100 MHz, DMSO-d_6) of S10

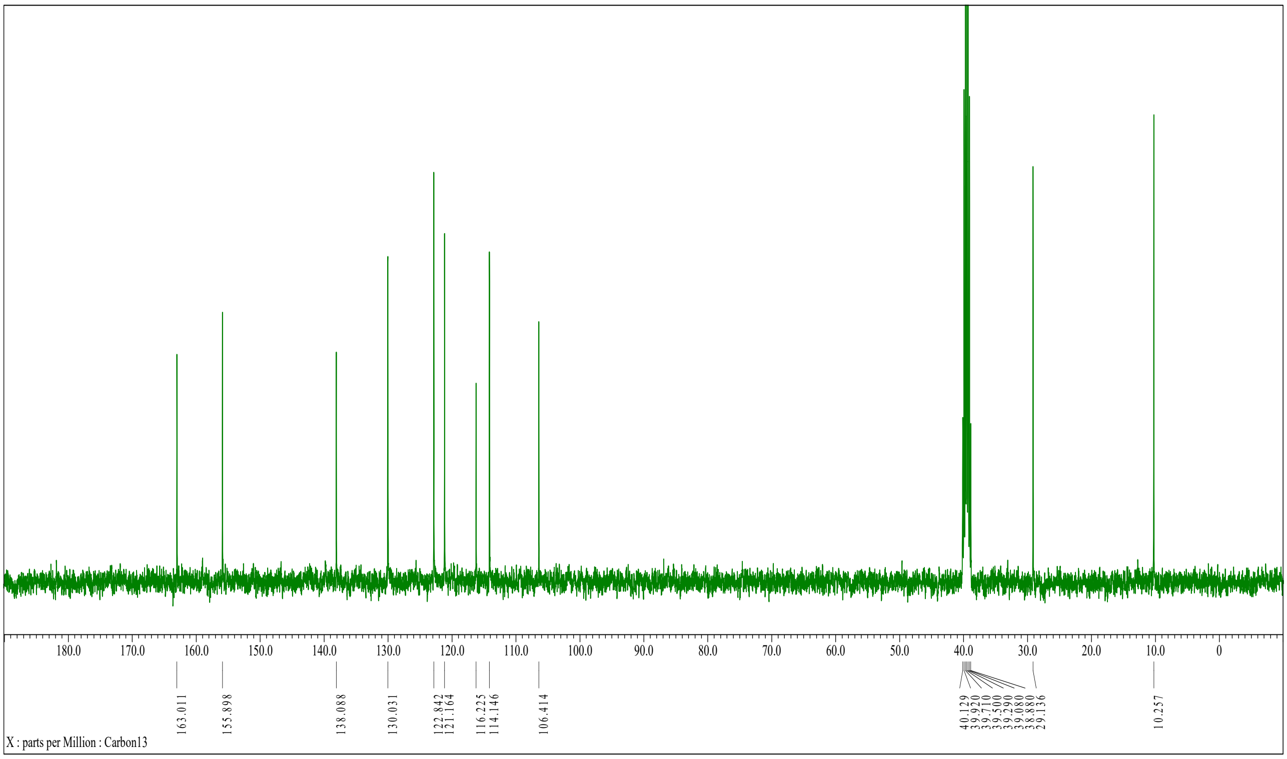

^{1}H NMR (400 MHz, $CDCl_3$) of S11

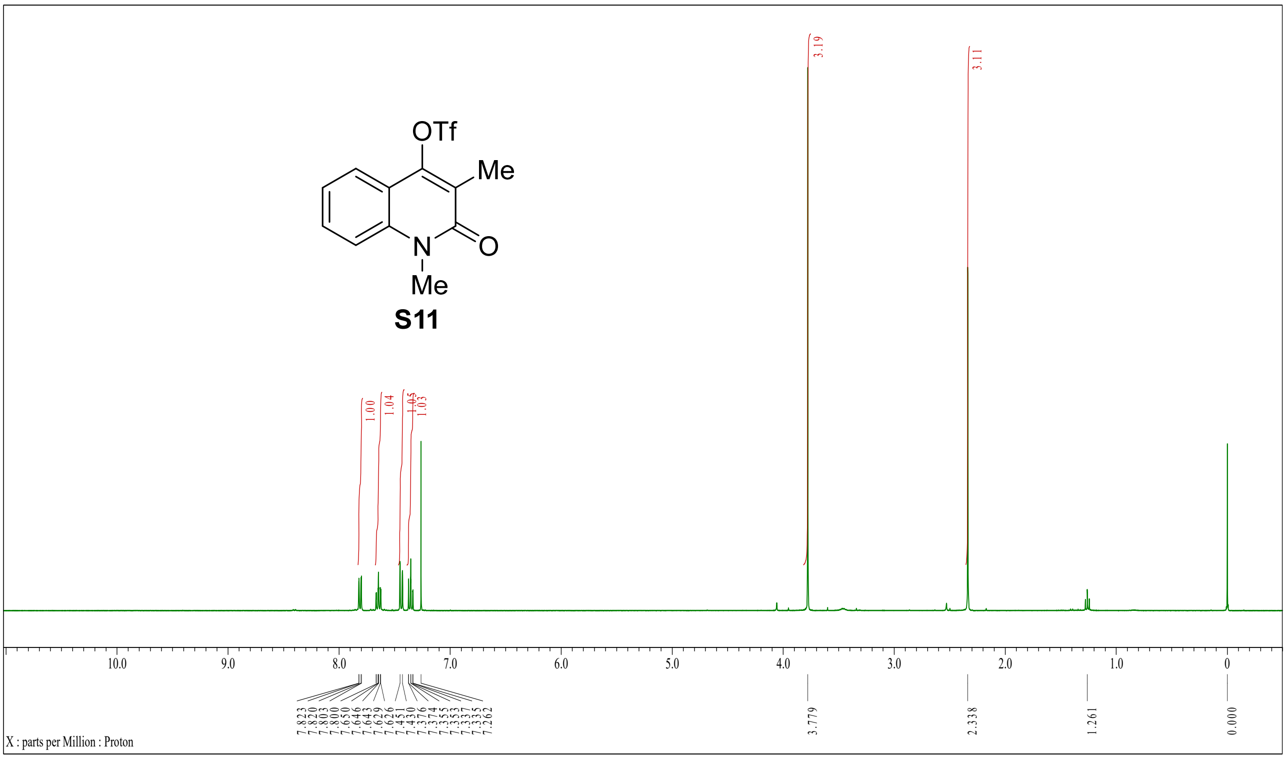

^{13}C NMR (100 MHz, $CDCl_3$) of S11

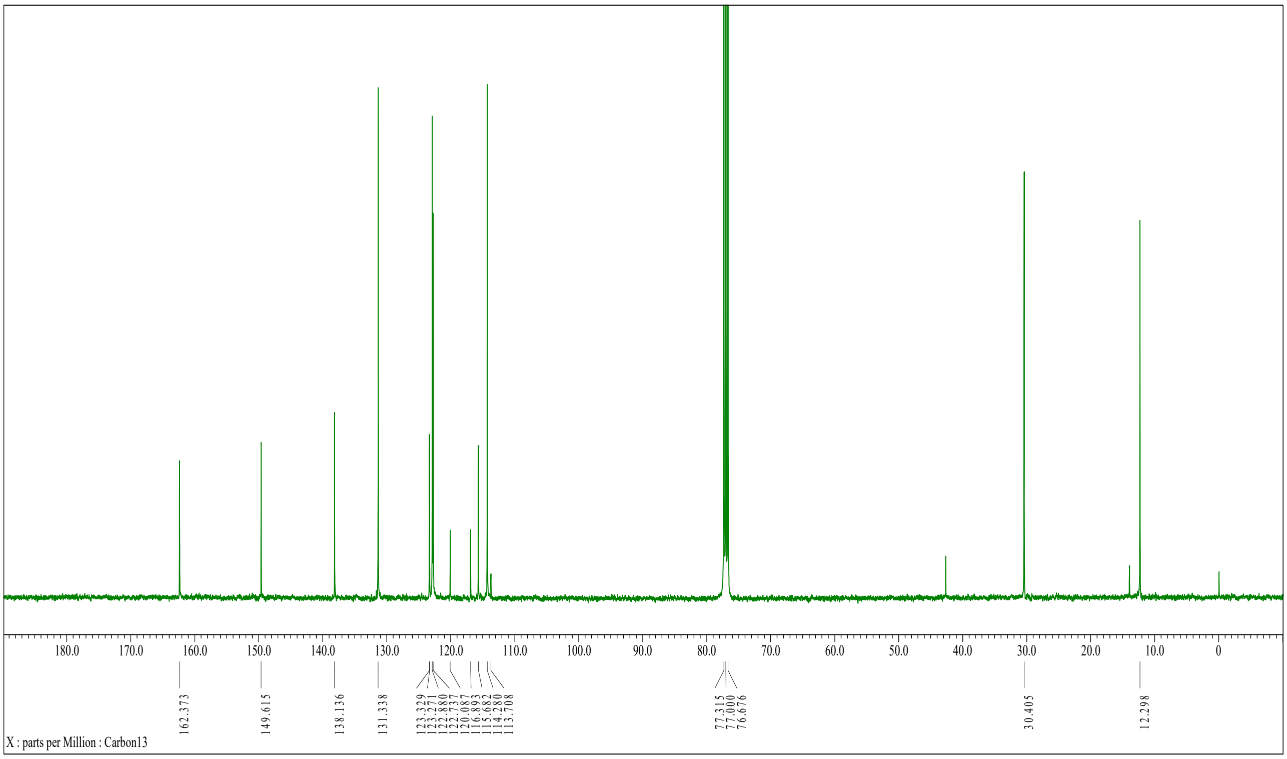

^{19}F NMR (376 MHz, $CDCl_3$) of S11

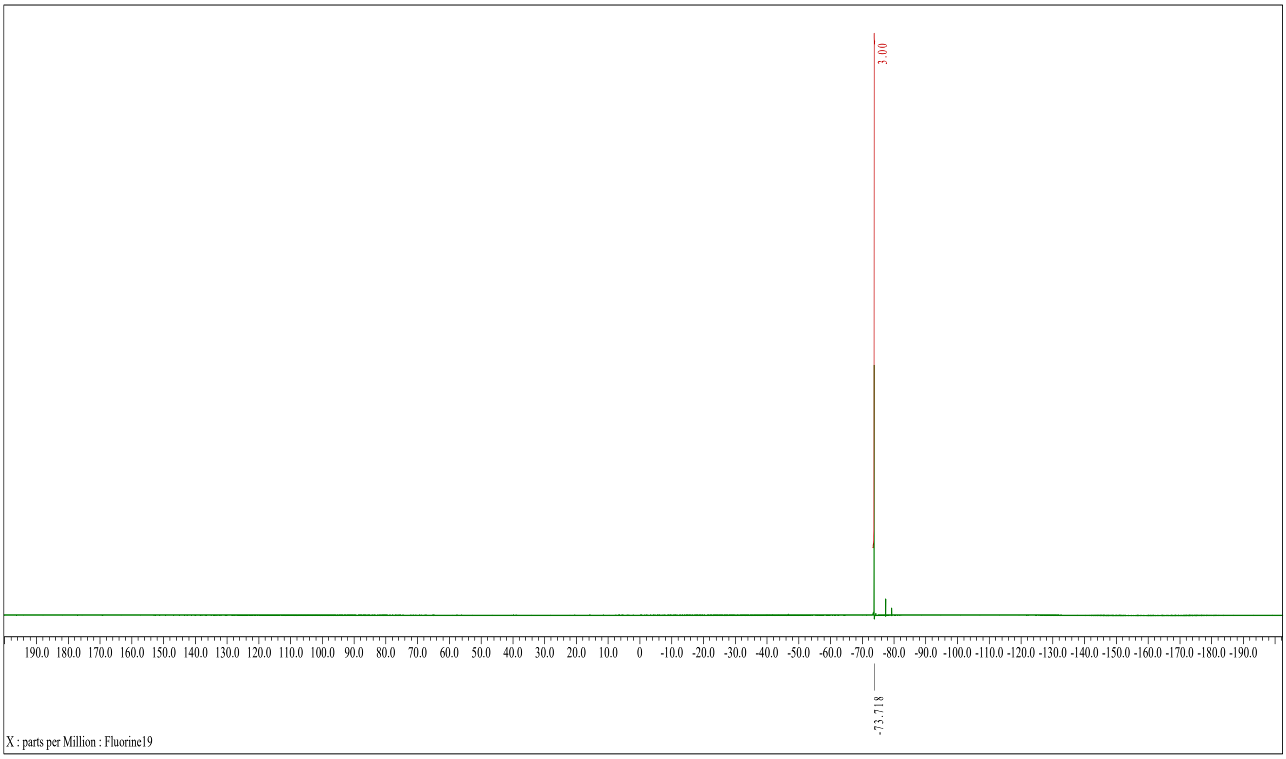

^{1}H NMR (400 MHz, $CDCl_3$) of 1h

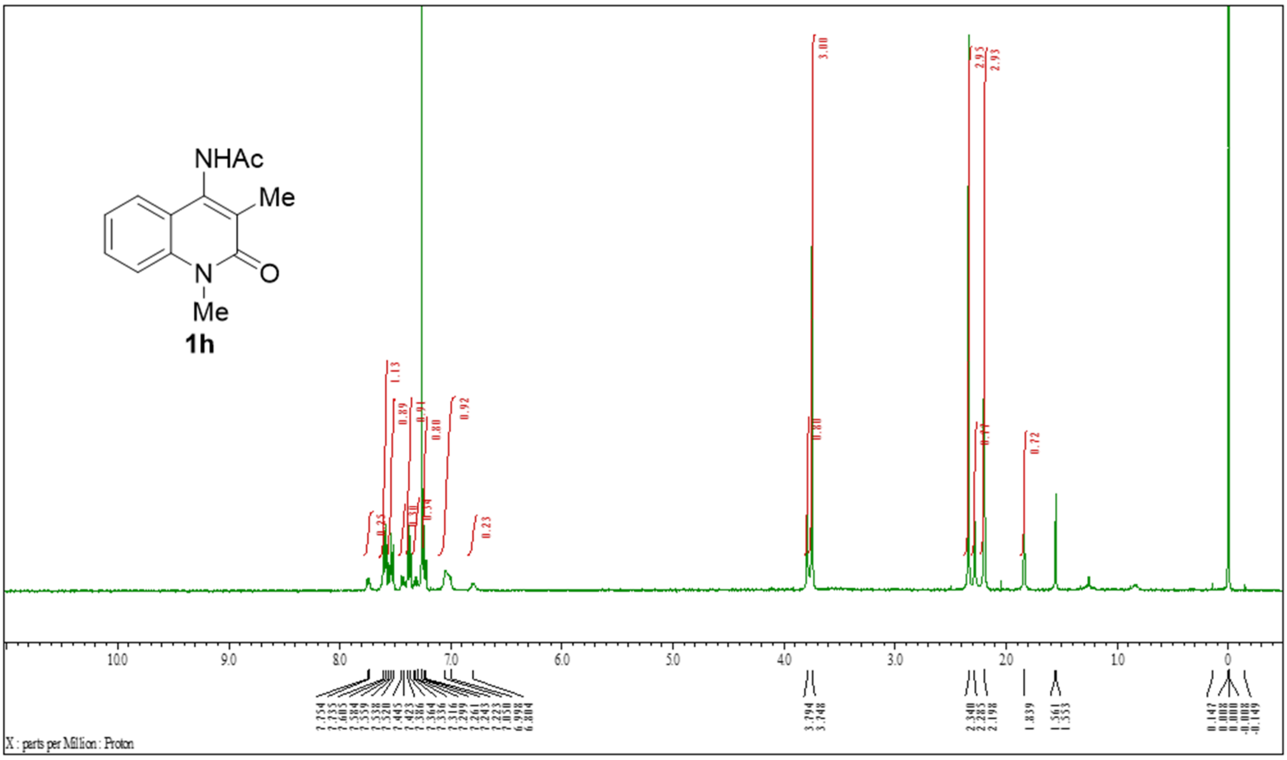

^{13}C NMR (100 MHz, DMSO-d_6) of 1h

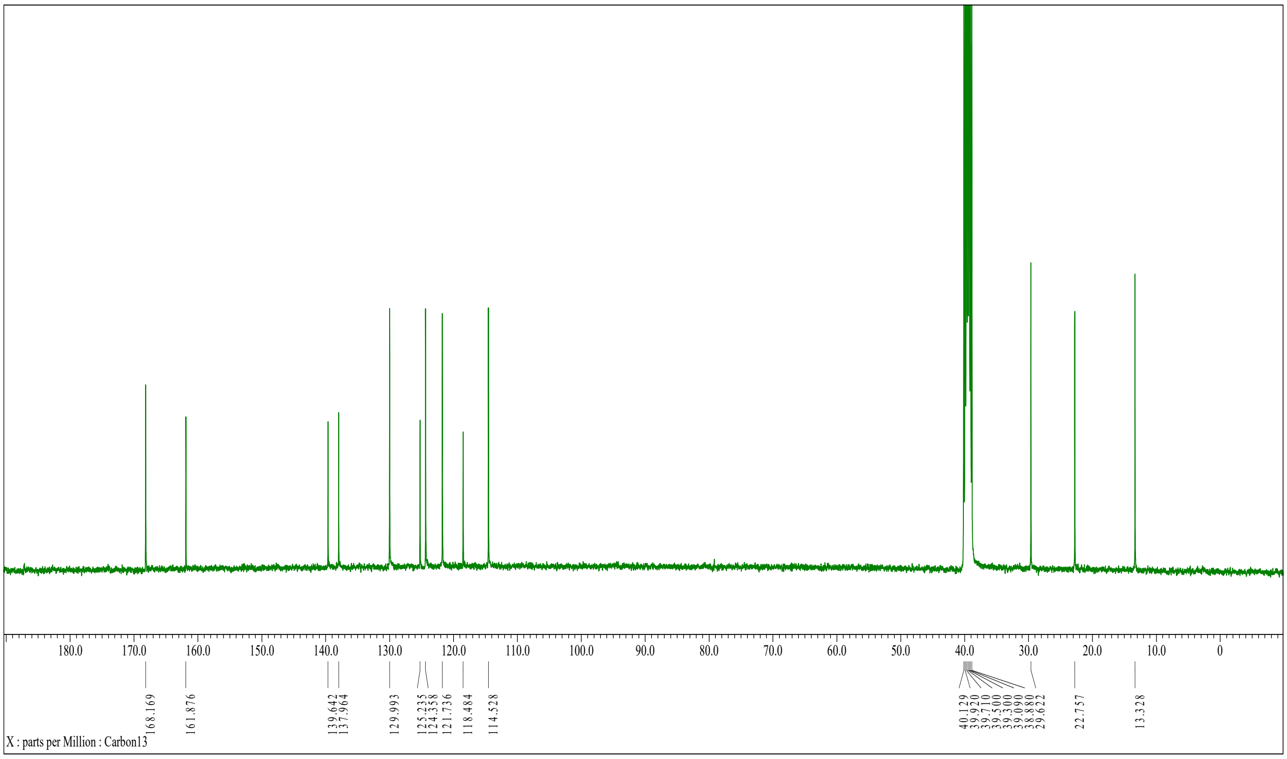

^{1}H NMR (400 MHz, $CDCl_3$) of 3ba

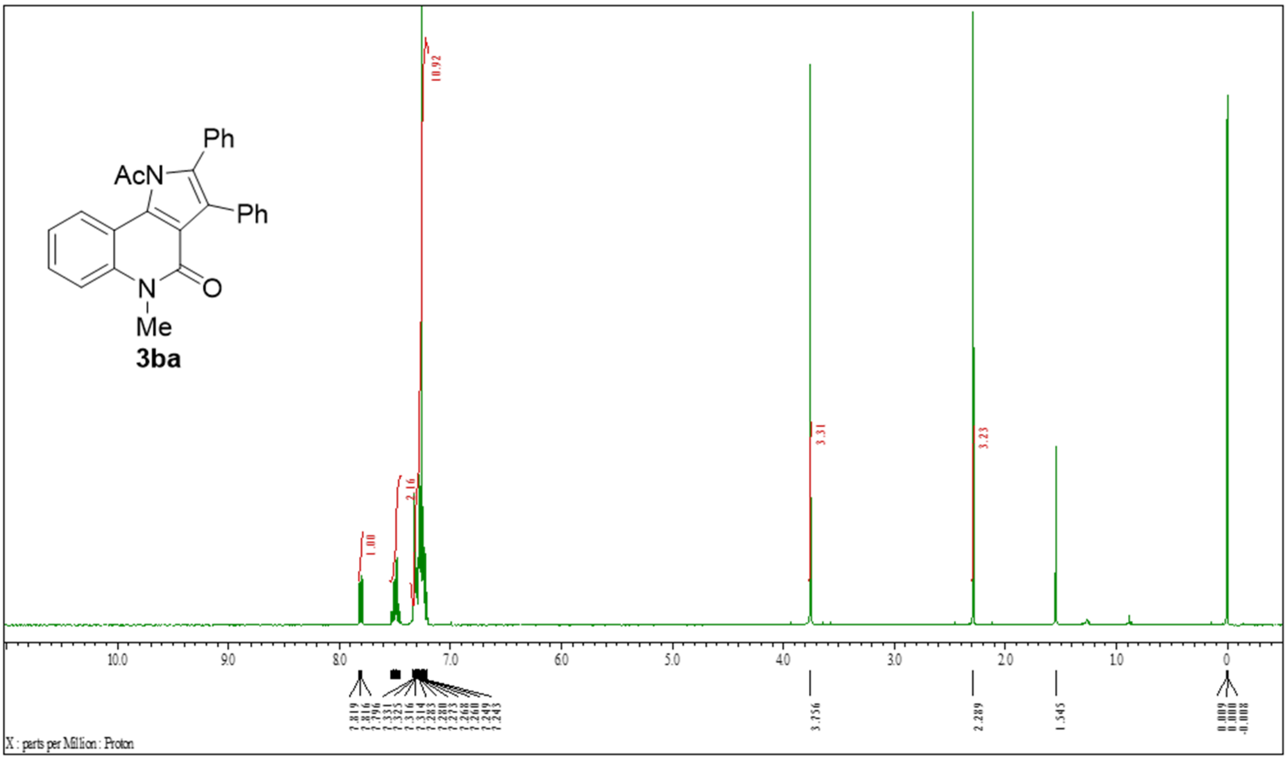

^{13}C NMR (100 MHz, $CDCl_3$) of 3ba

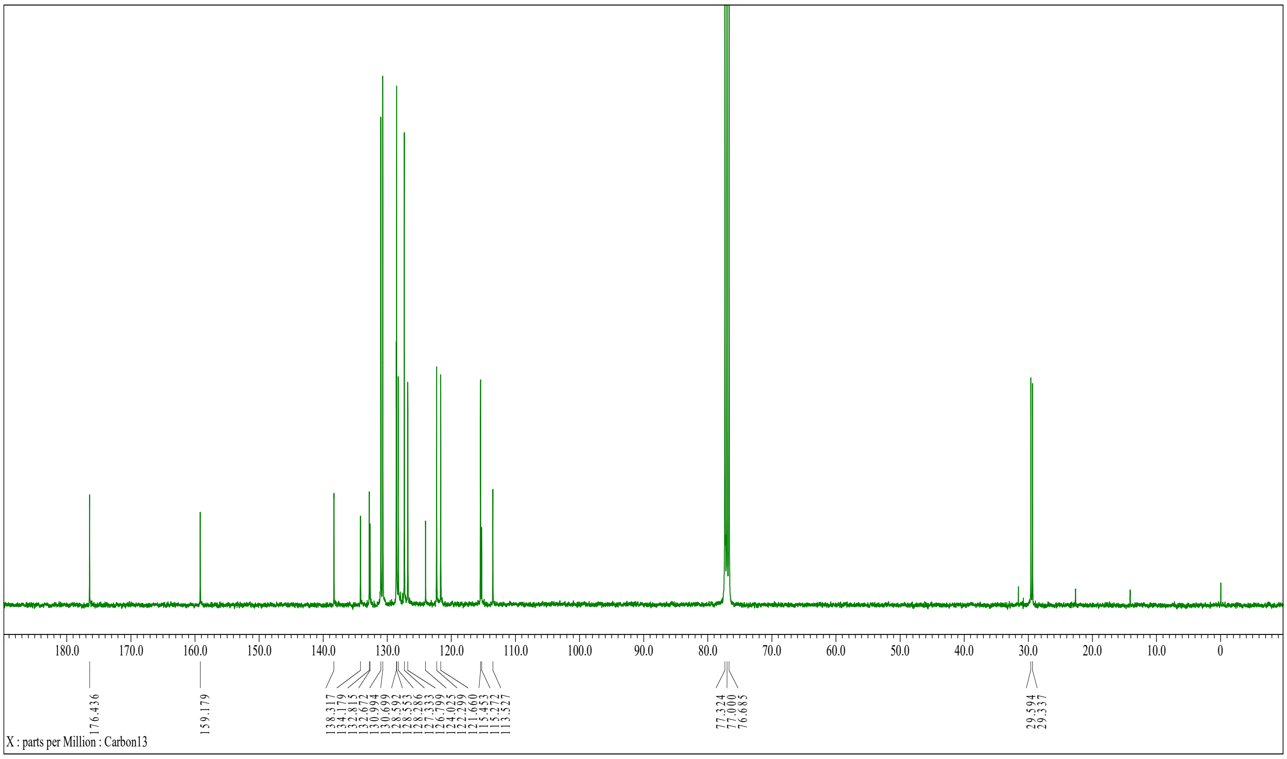

^{1}H NMR (400 MHz, $CDCl_3$) of 3bb

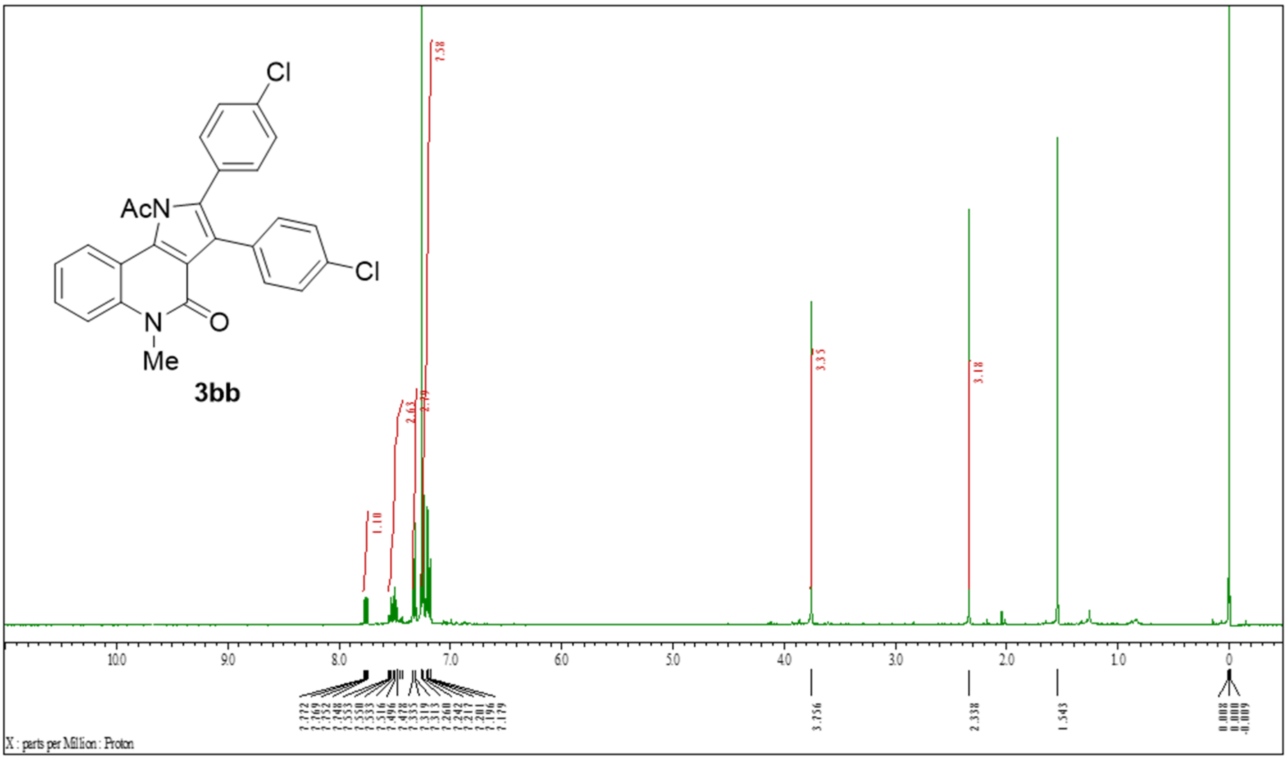

^{13}C NMR (100 MHz, $CDCl_3$) of 3bb

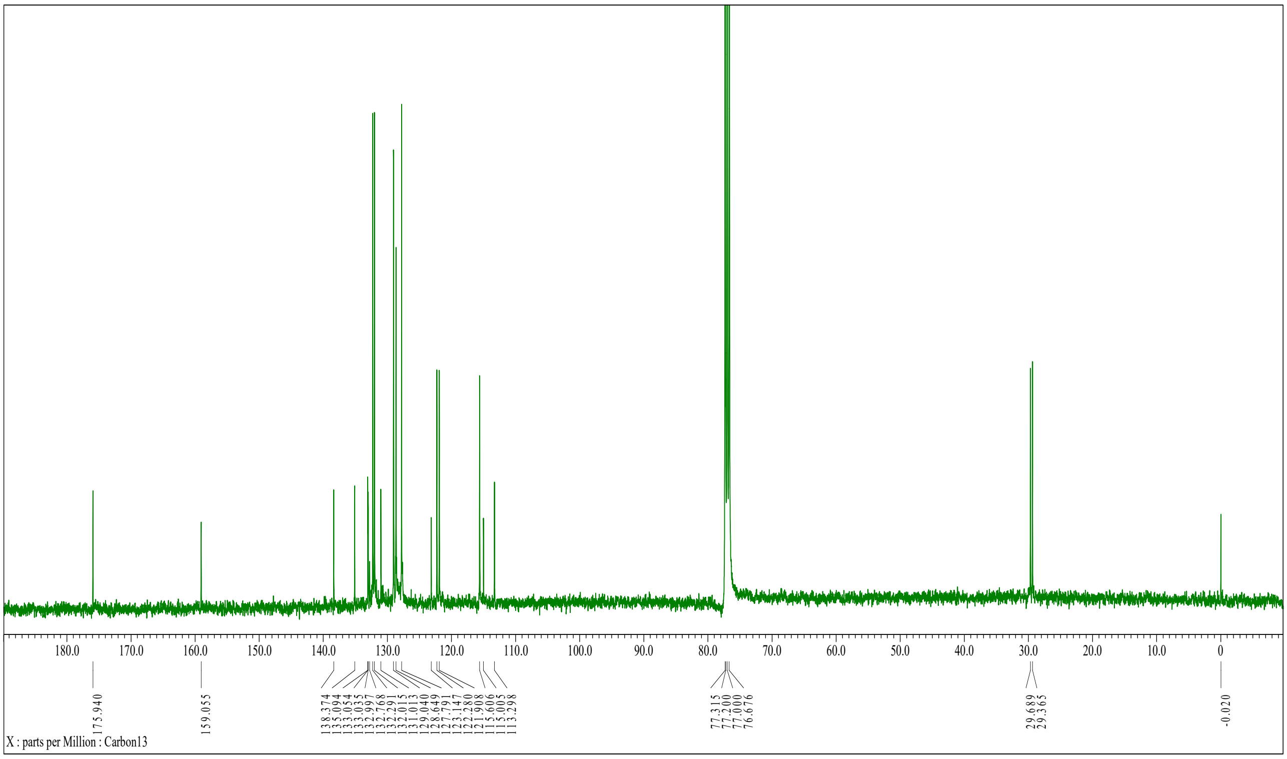

^{1}H NMR (400 MHz, $CDCl_3$) of 3bc

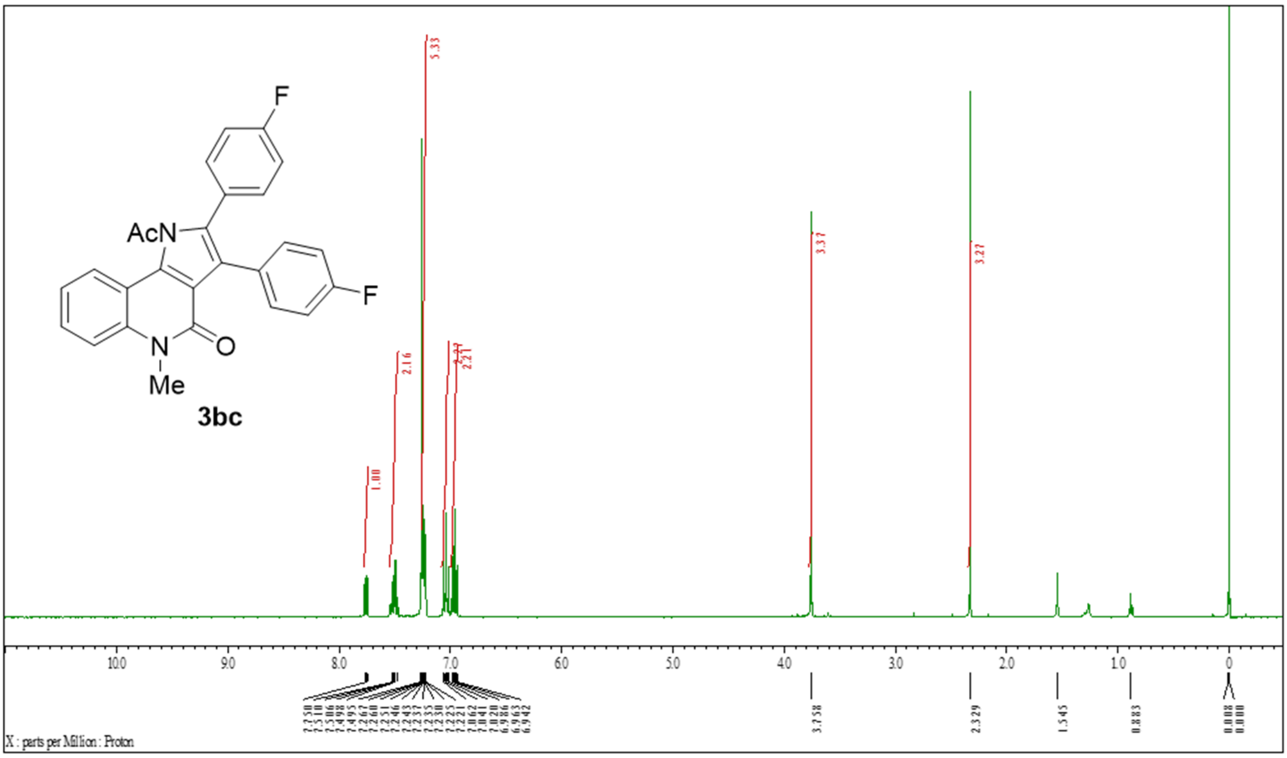

^{13}C NMR (100 MHz, $CDCl_3$) of 3bc

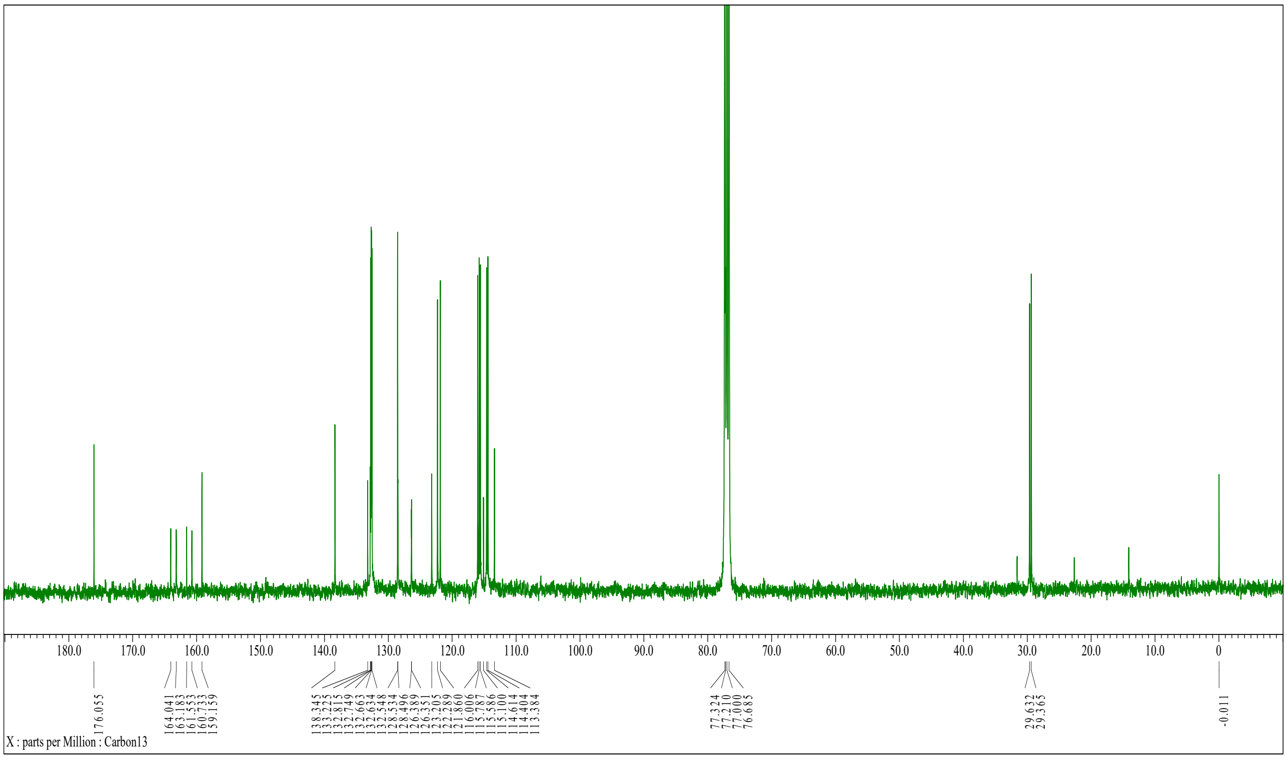

^{19}F NMR (376 MHz, $CDCl_3$) of 3bc

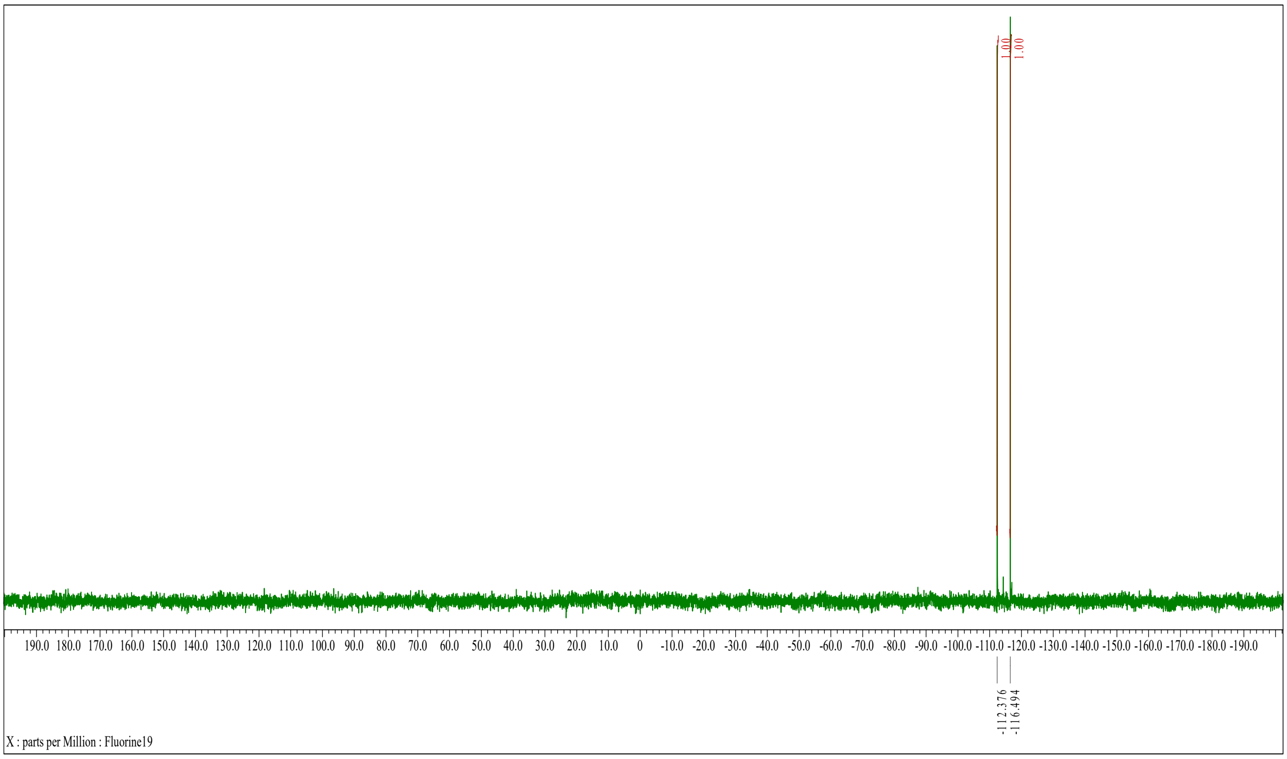

^{1}H NMR (400 MHz, $CDCl_3$) of 3bf

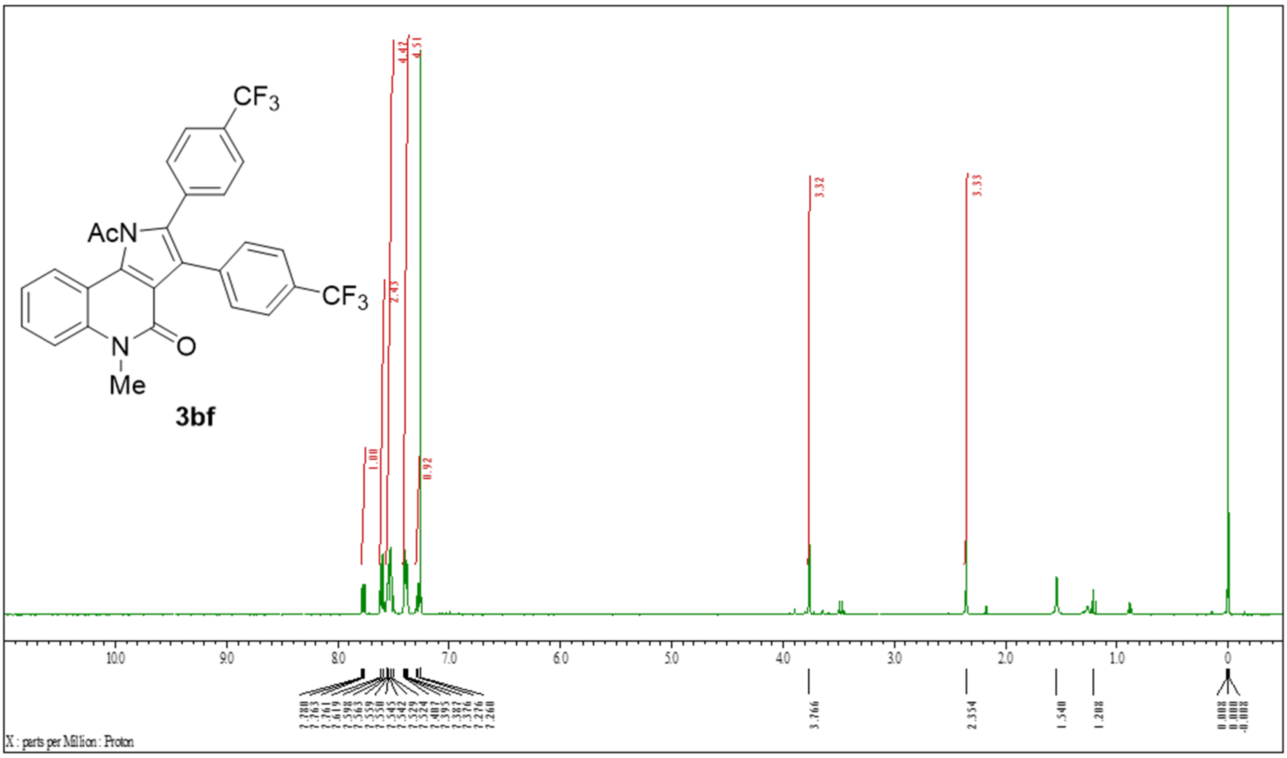

^{13}C NMR (100 MHz, $CDCl_3$) of 3bf

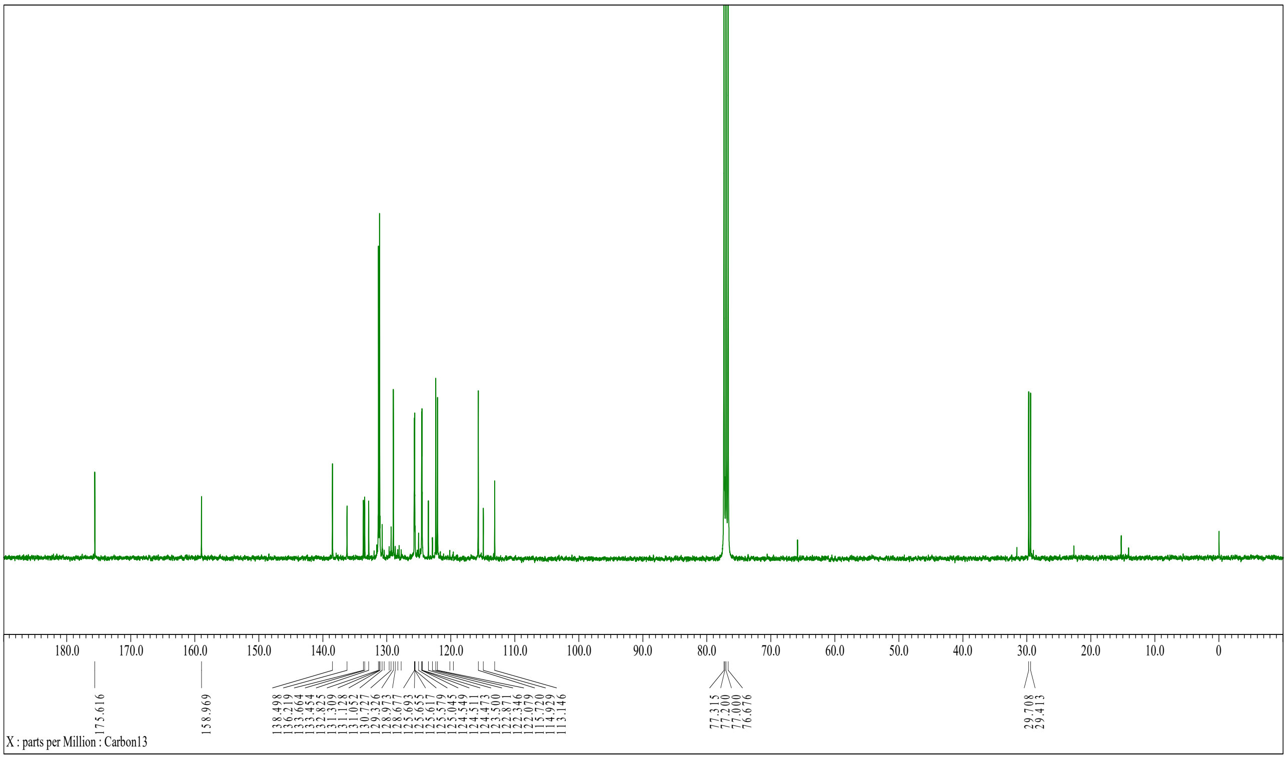

^{19}F NMR (376 MHz, $CDCl_3$) of 3bf

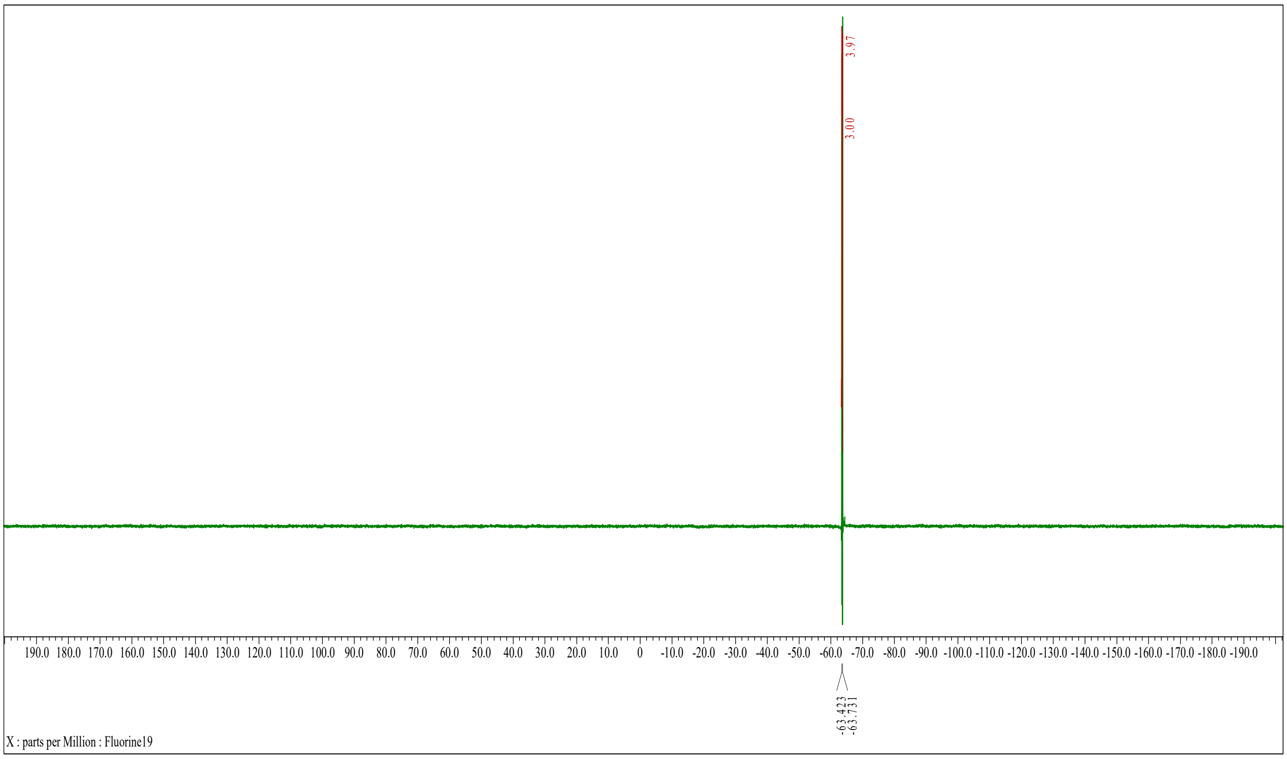

^{1}H NMR (400 MHz, $CDCl_3$) of 3bf'

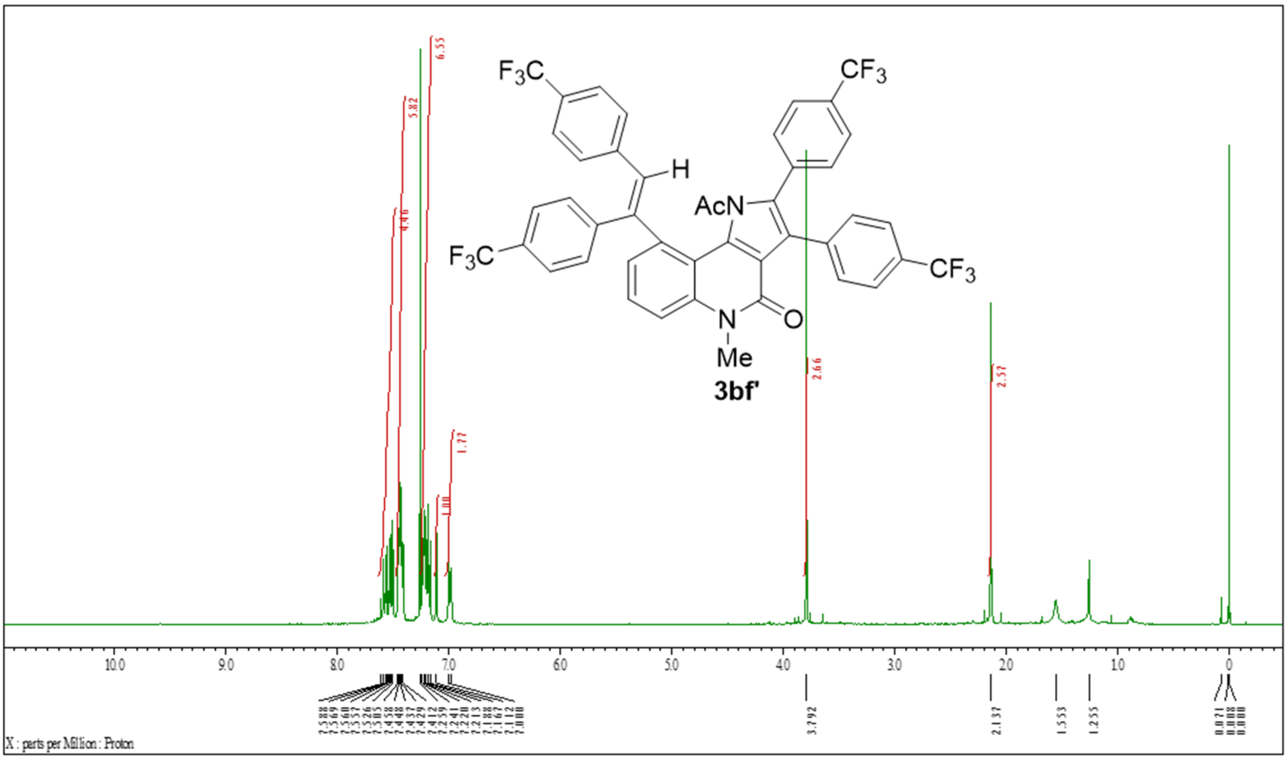

^{13}C NMR (100 MHz, $CDCl_3$) of 3bf'

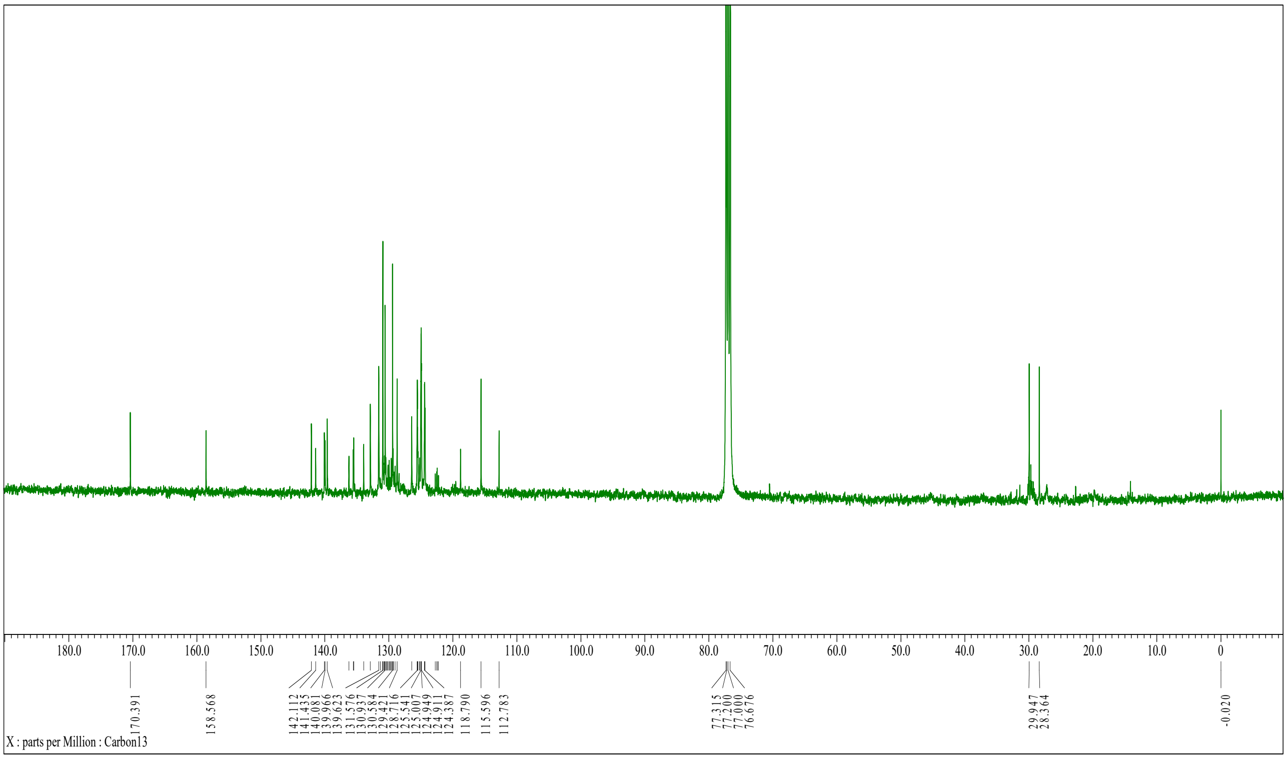

^{19}F NMR (376 MHz, $CDCl_3$) of 3bf’

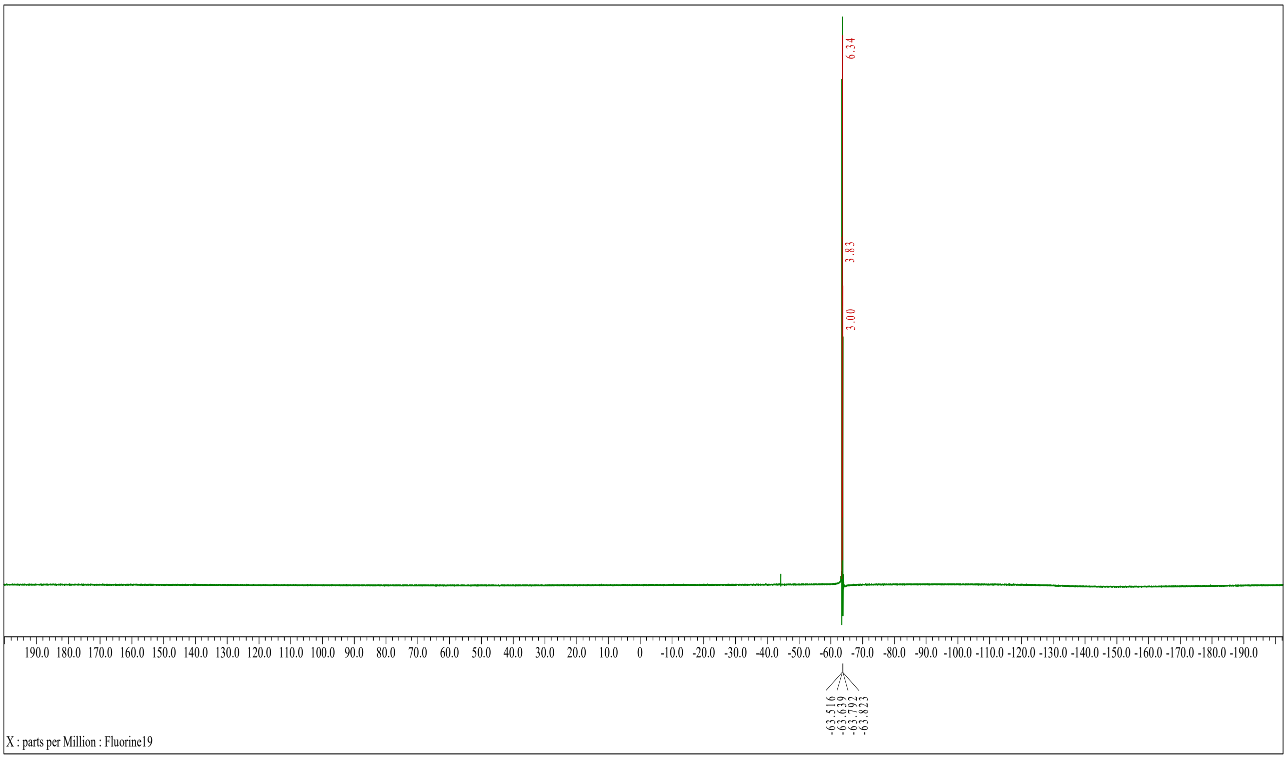

^{1}H NMR (400 MHz, $CDCl_3$) of 3bg

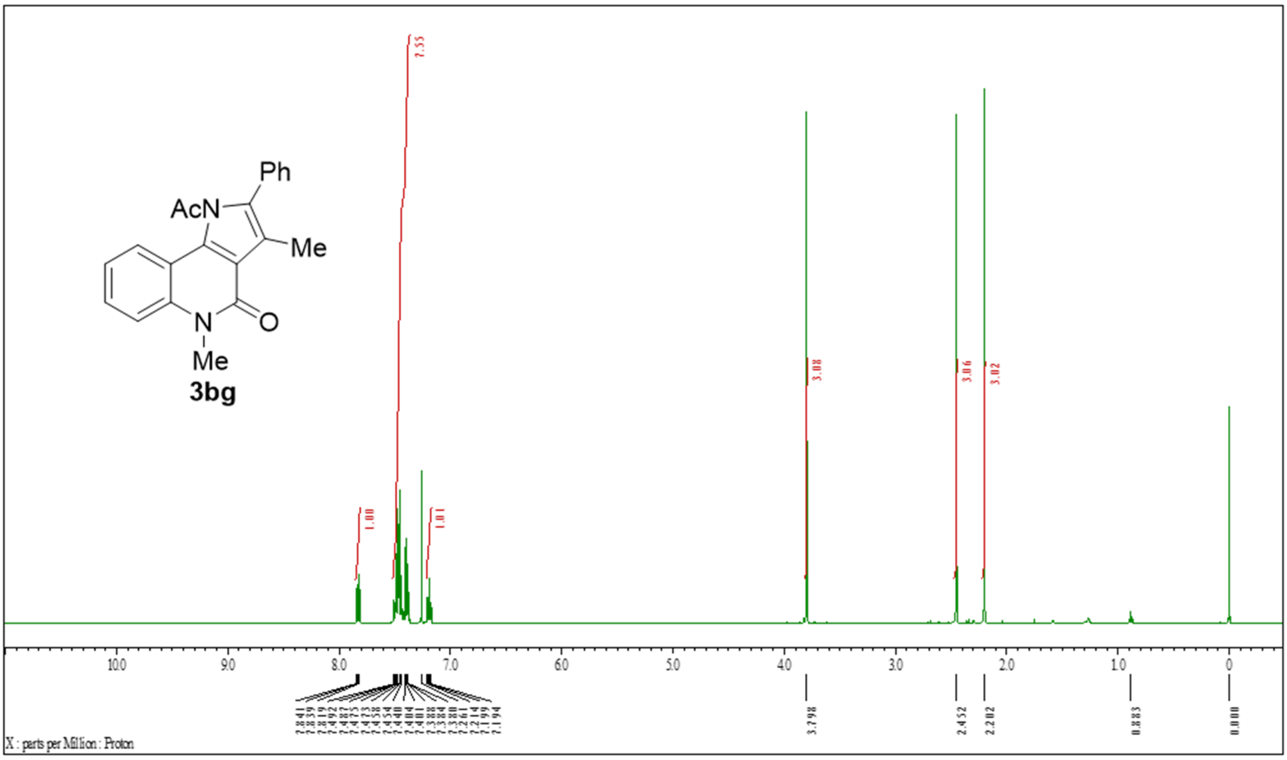

^{13}C NMR (100 MHz, $CDCl_3$) of 3bg

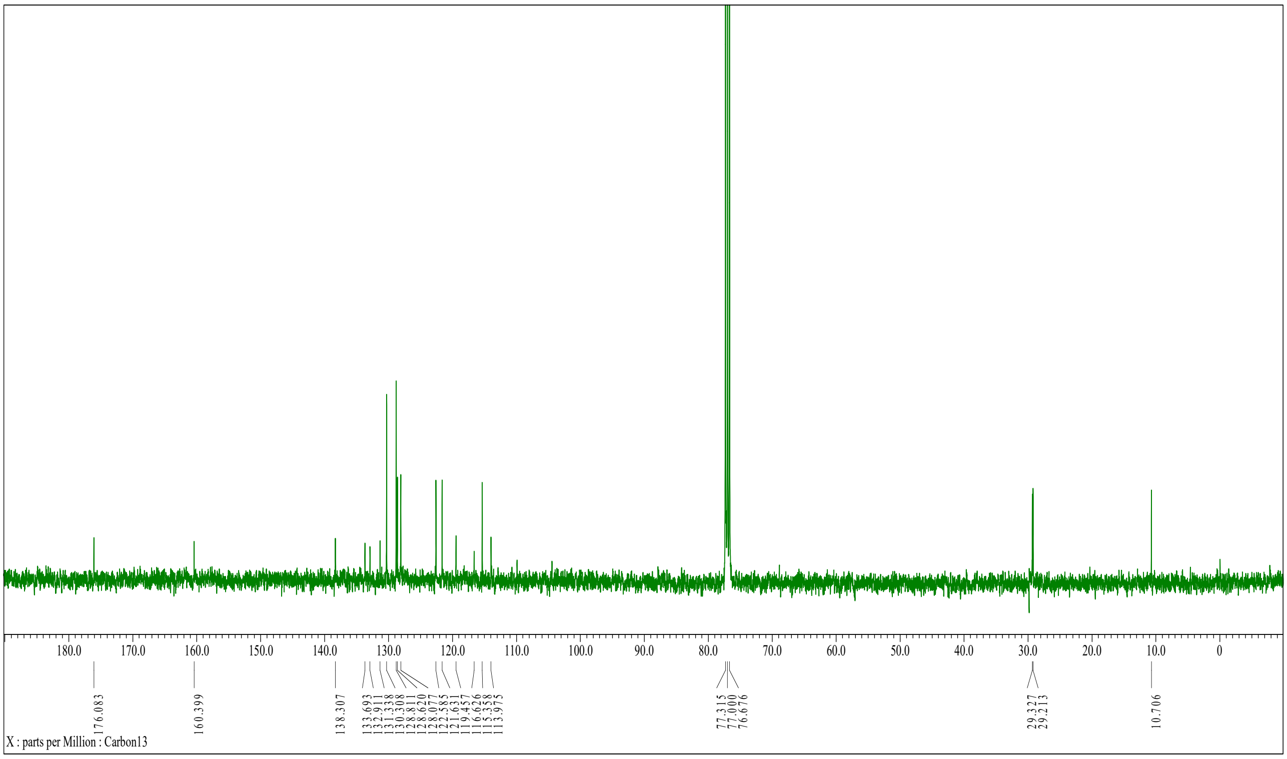

^{1}H NMR (400 MHz, $CDCl_3$) of 3b'g

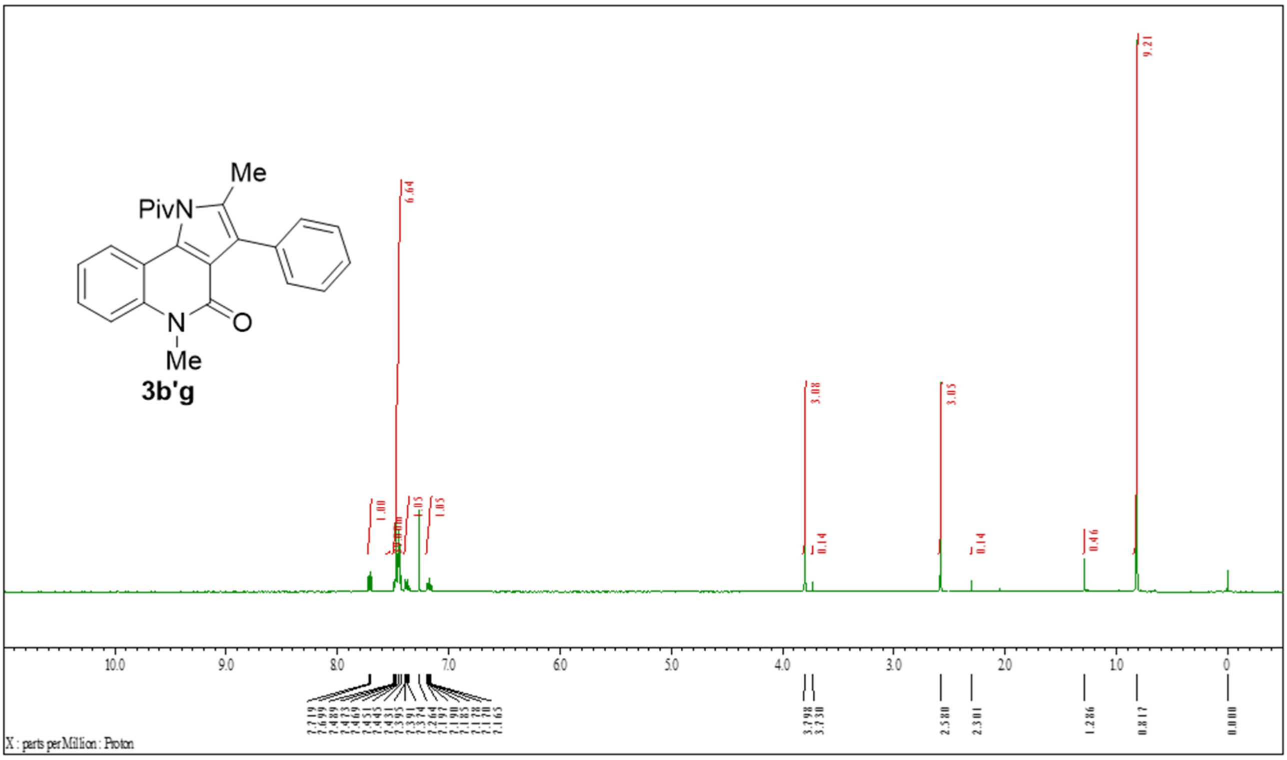

^{13}C NMR (100 MHz, $CDCl_3$) of 3b'g

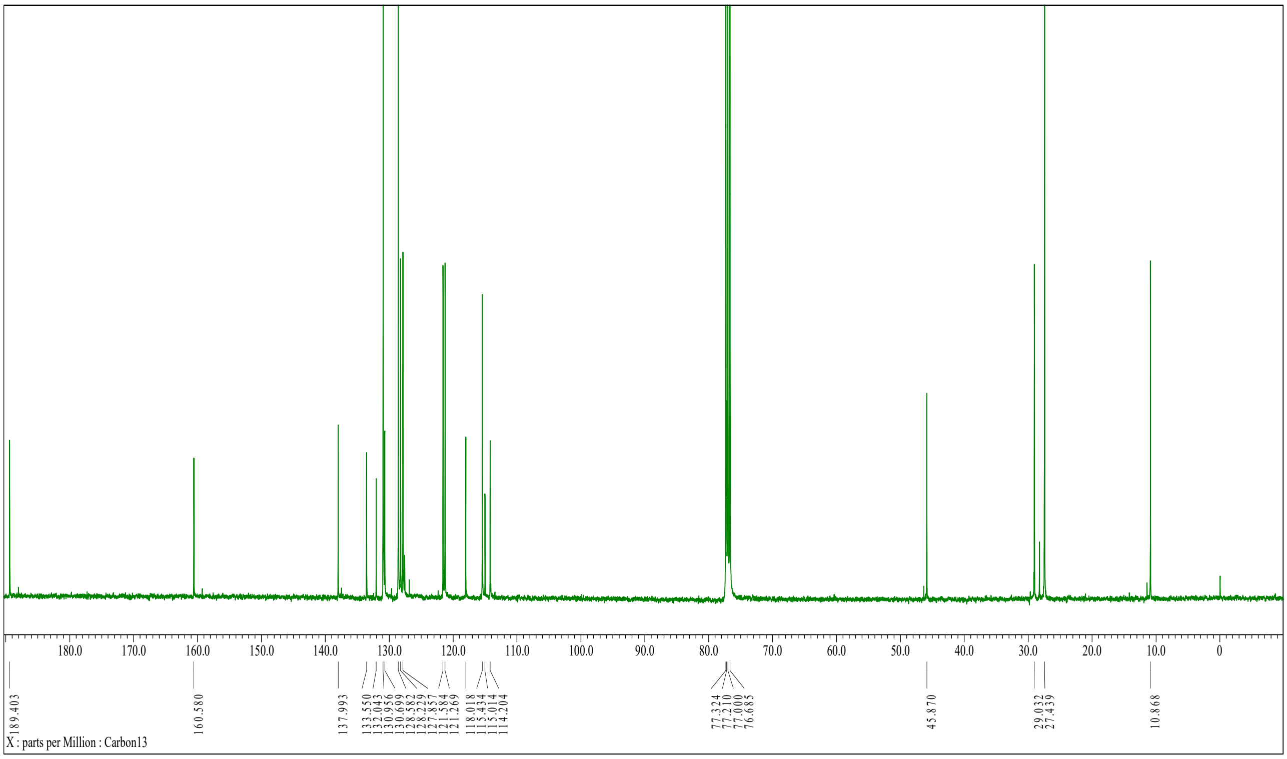

^{1}H NMR (400 MHz, $CDCl_3$) of 3bh

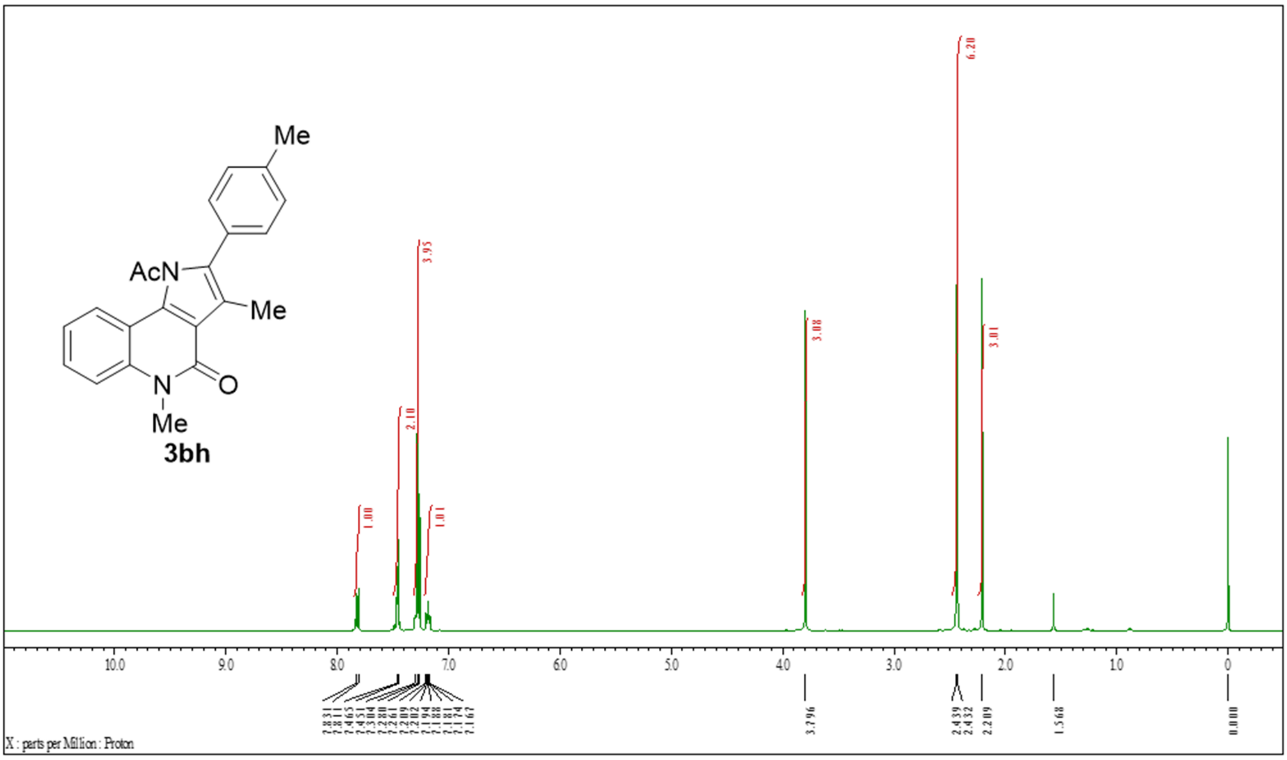

^{13}C NMR (100 MHz, $CDCl_3$) of 3bh

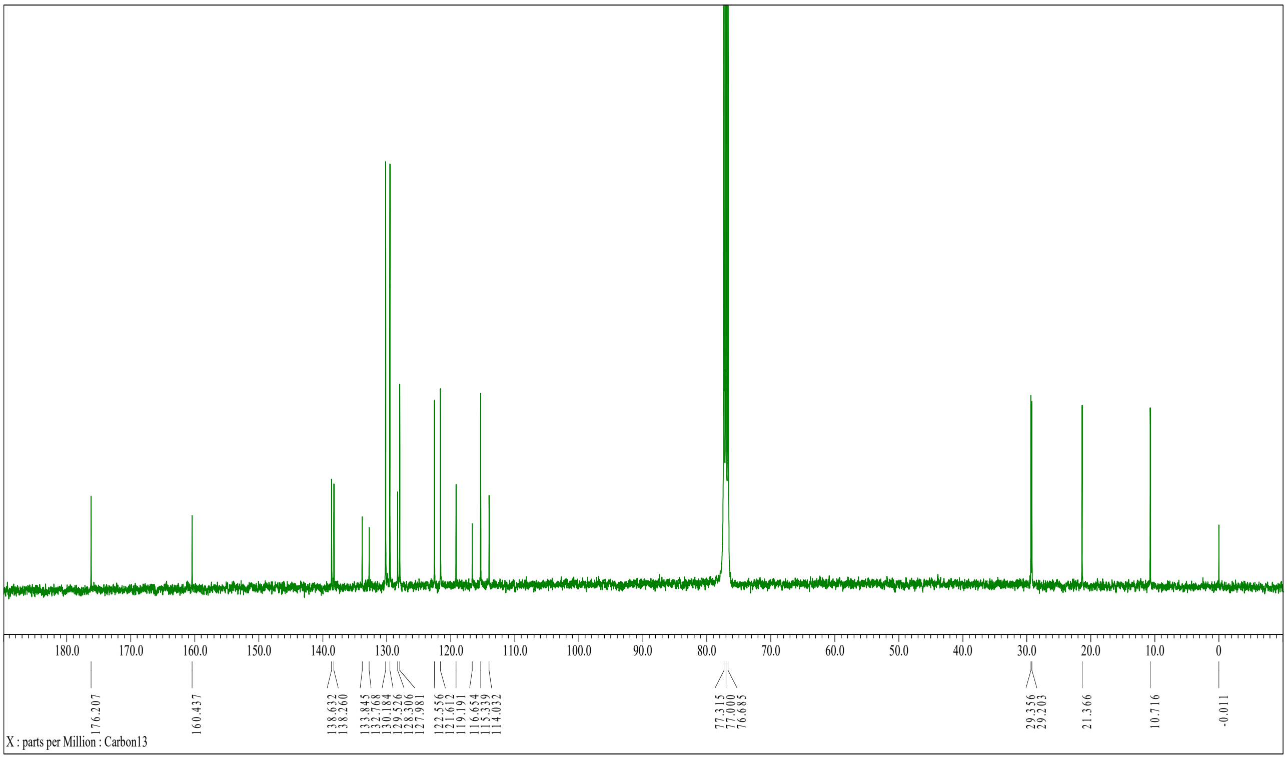

^{1}H NMR (400 MHz, $CDCl_3$) of 3bi

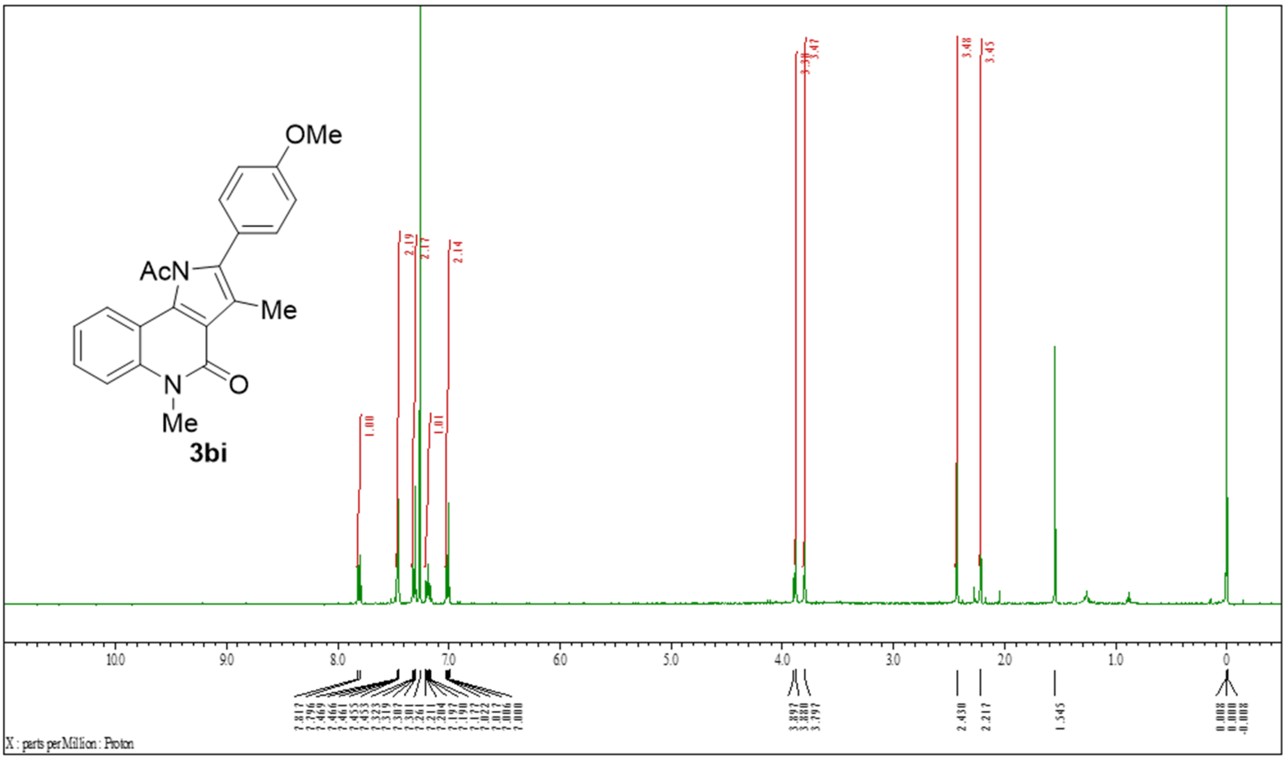

^{13}C NMR (100 MHz, $CDCl_3$) of 3bi

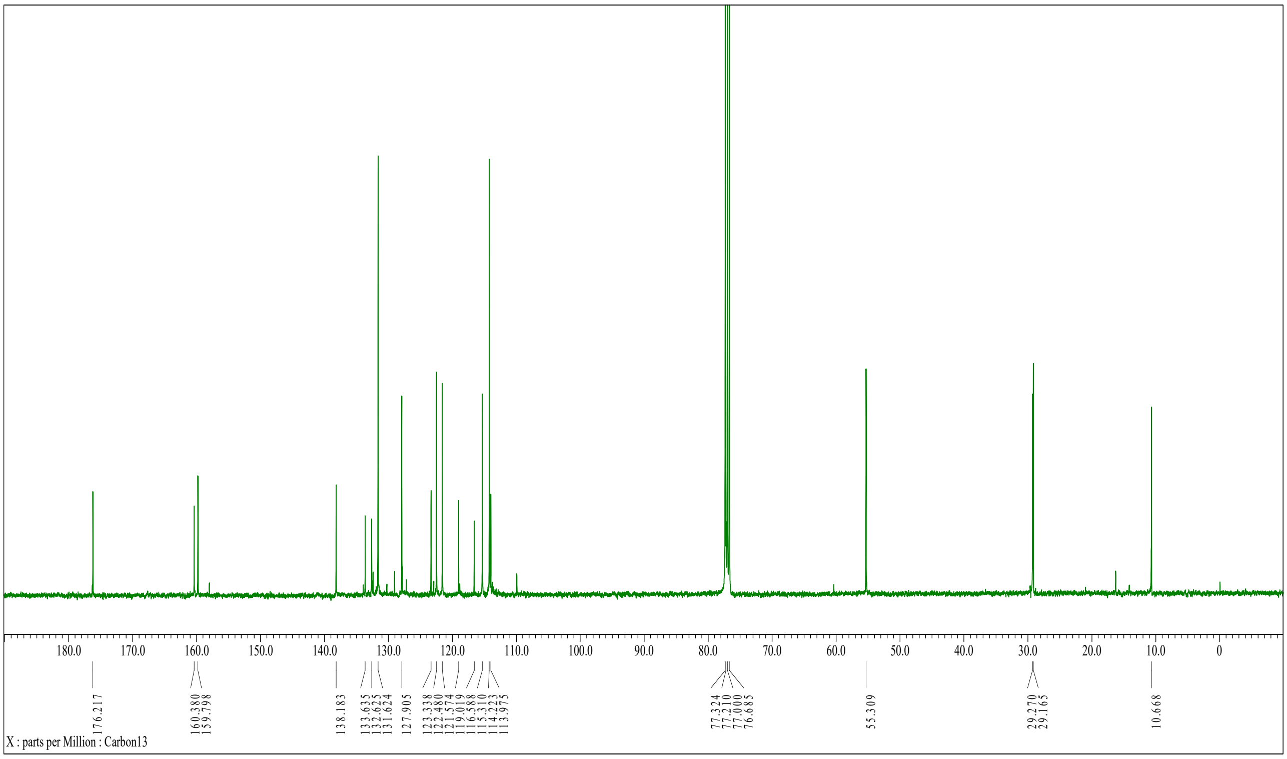

^{1}H NMR (400 MHz, $CDCl_3$) of 3bj

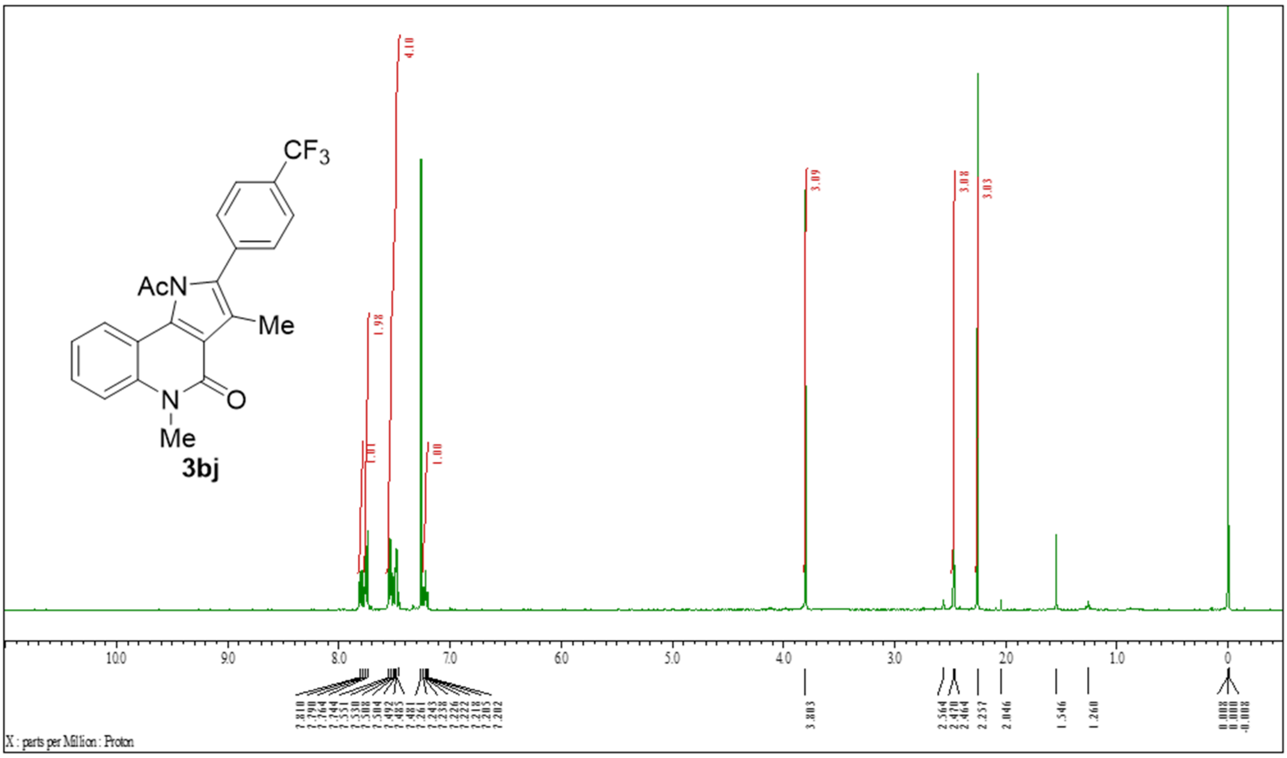

^{13}C NMR (100 MHz, $CDCl_3$) of 3bj

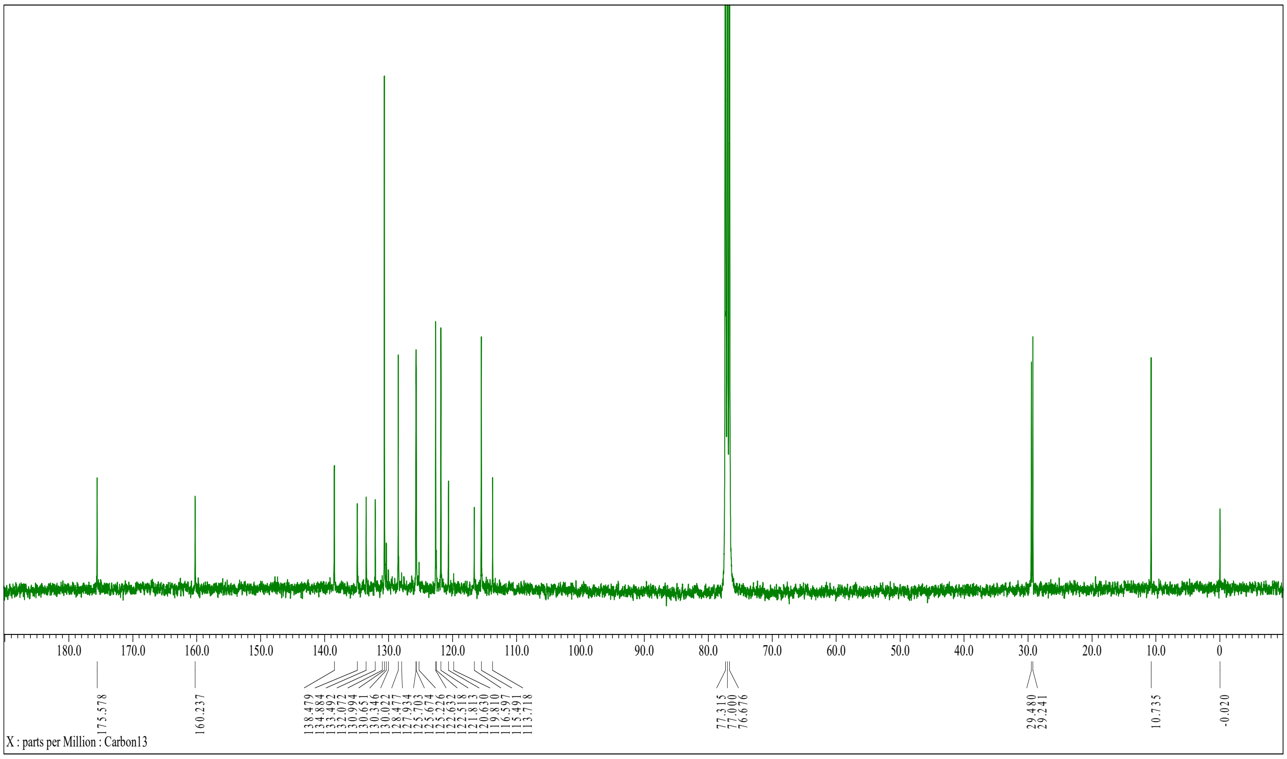

^{19}F NMR (376 MHz, $CDCl_3$) of 3bj

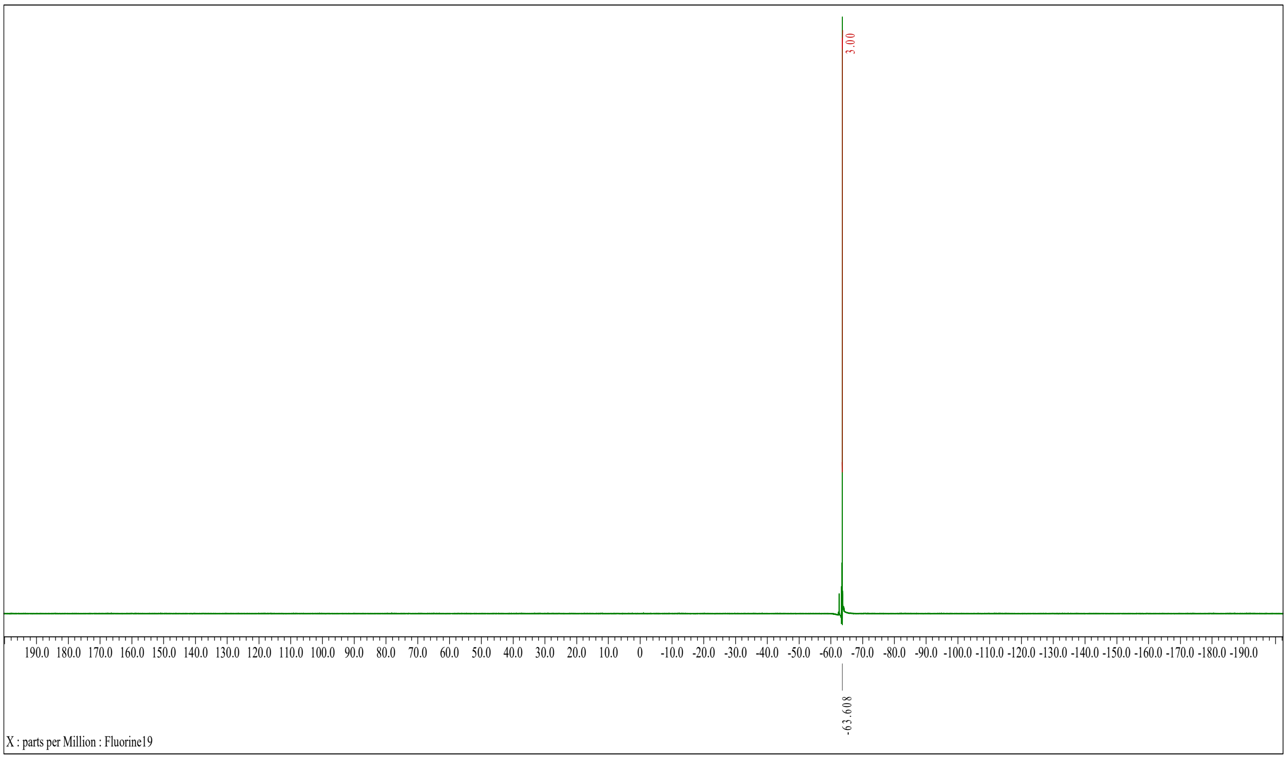

^{1}H NMR (400 MHz, CDCl$_3$) of 3bk

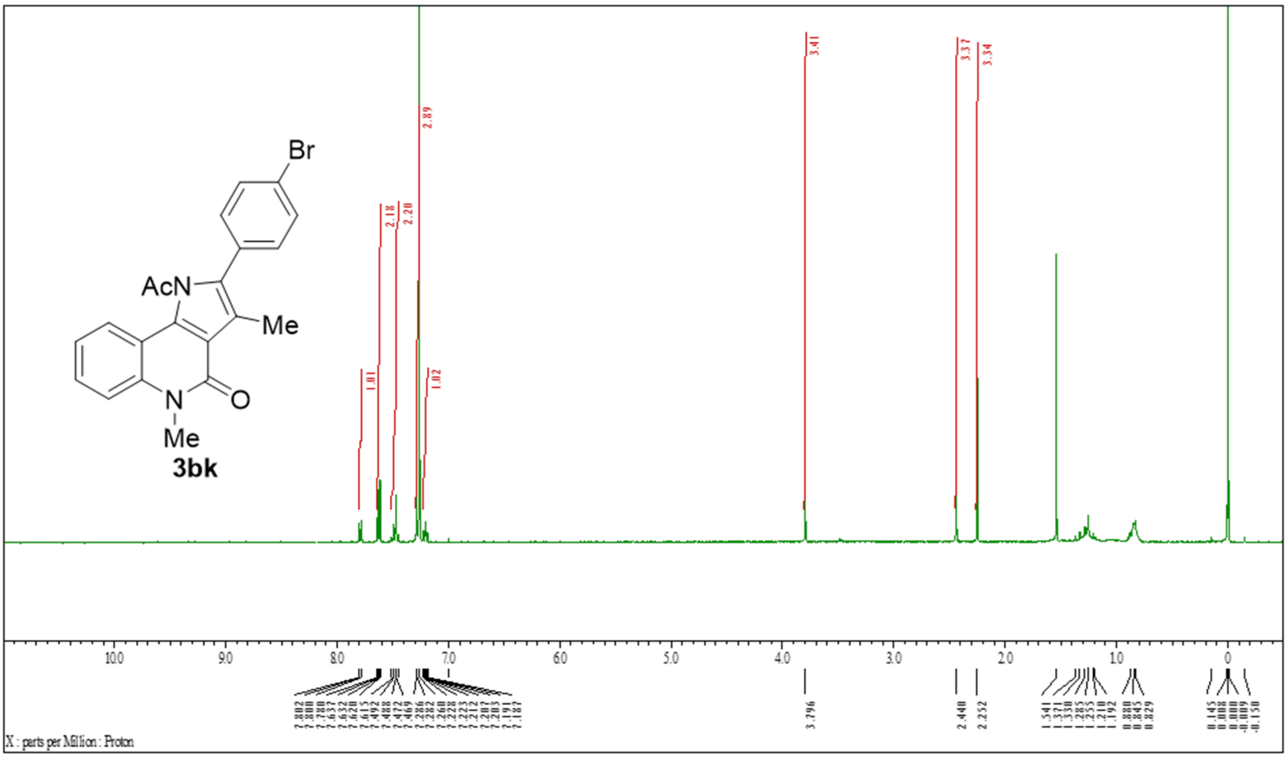

^{13}C NMR (100 MHz, CDCl$_3$) of 3bk

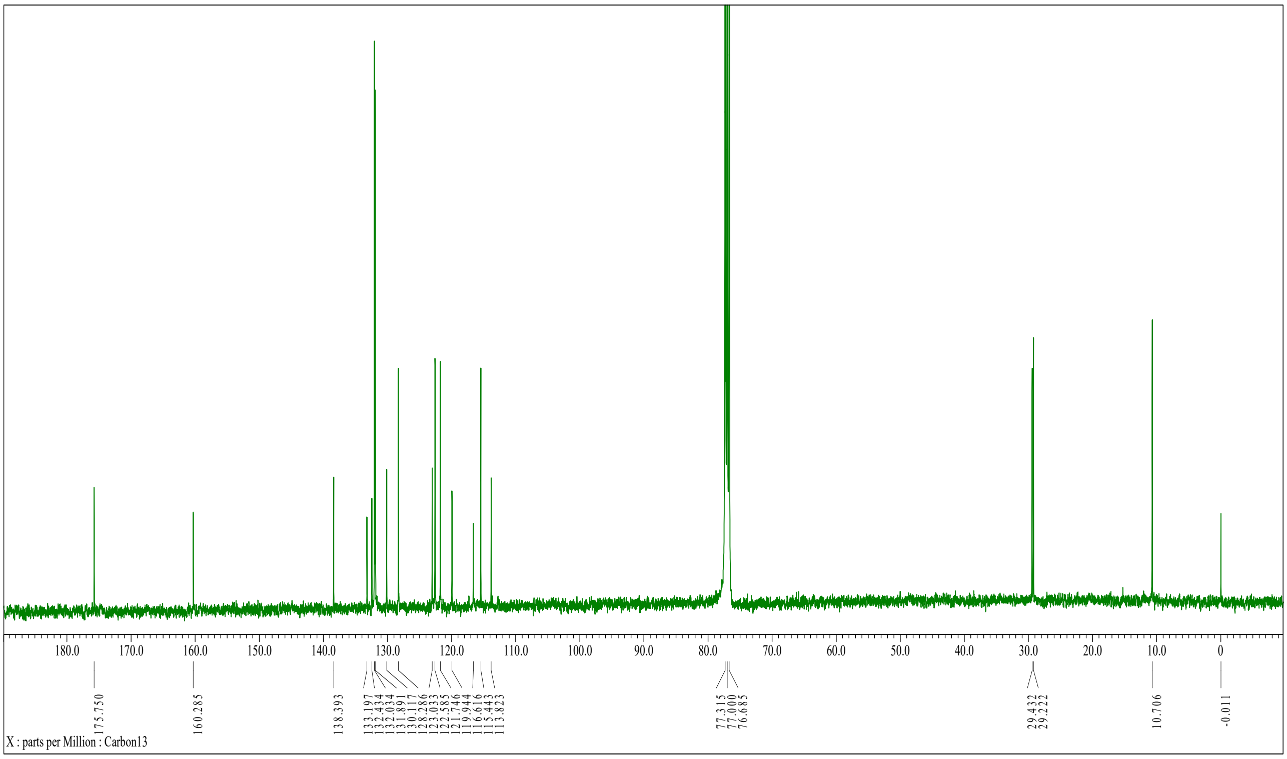

^{1}H NMR (400 MHz, $CDCl_3$) of 3bl

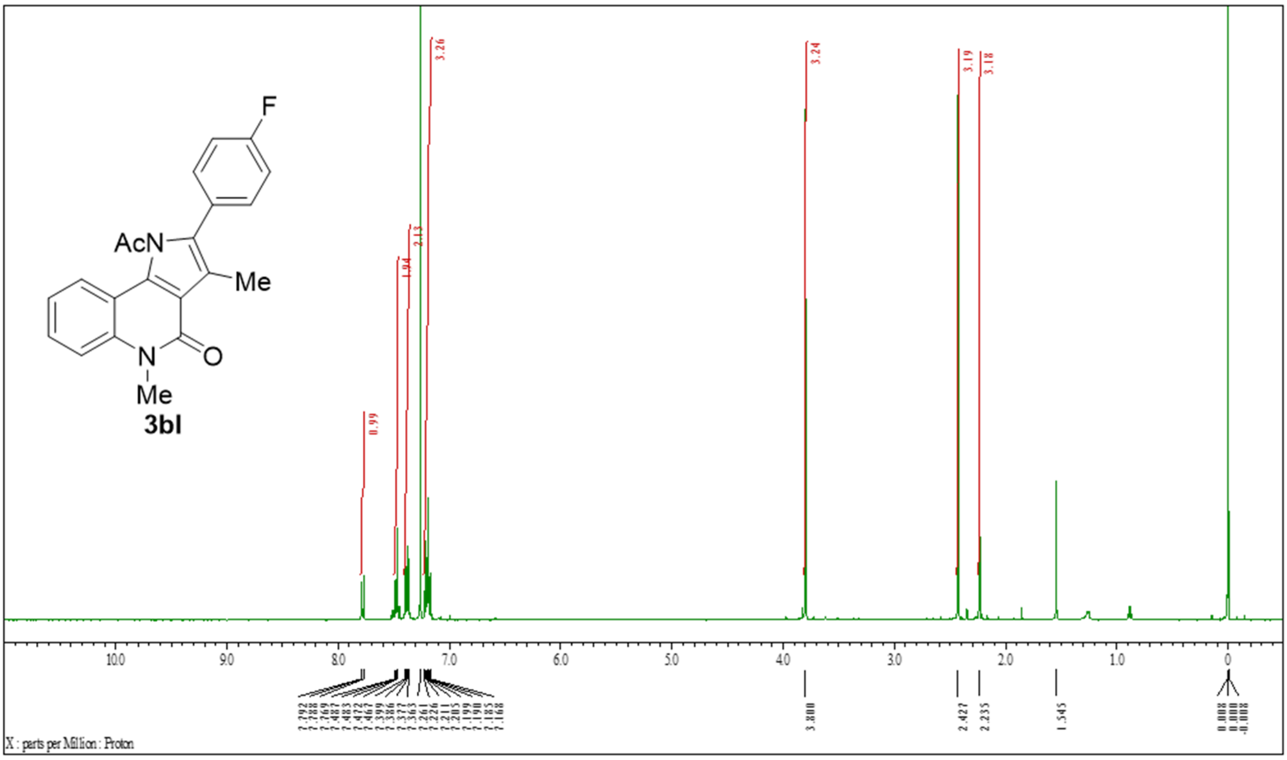

^{13}C NMR (100 MHz, $CDCl_3$) of 3bl

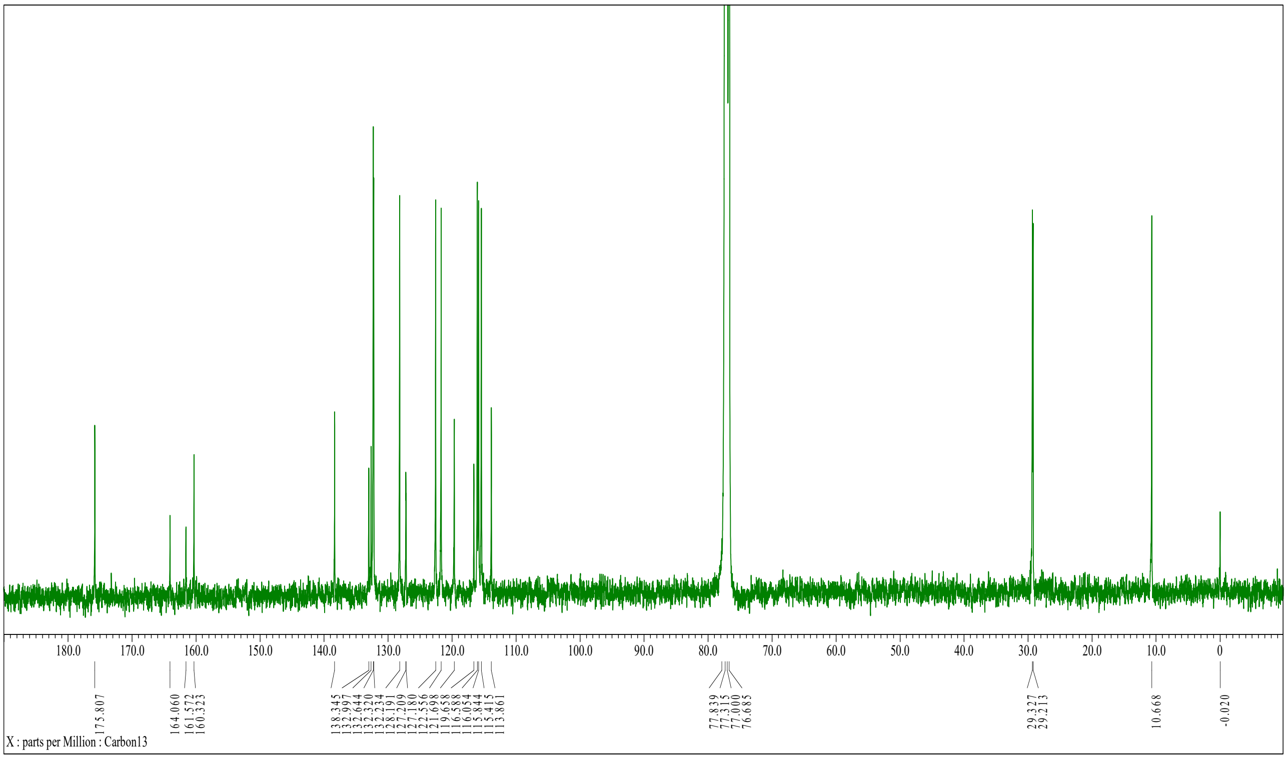

^{19}F NMR (376 MHz, $CDCl_3$) of 3bl

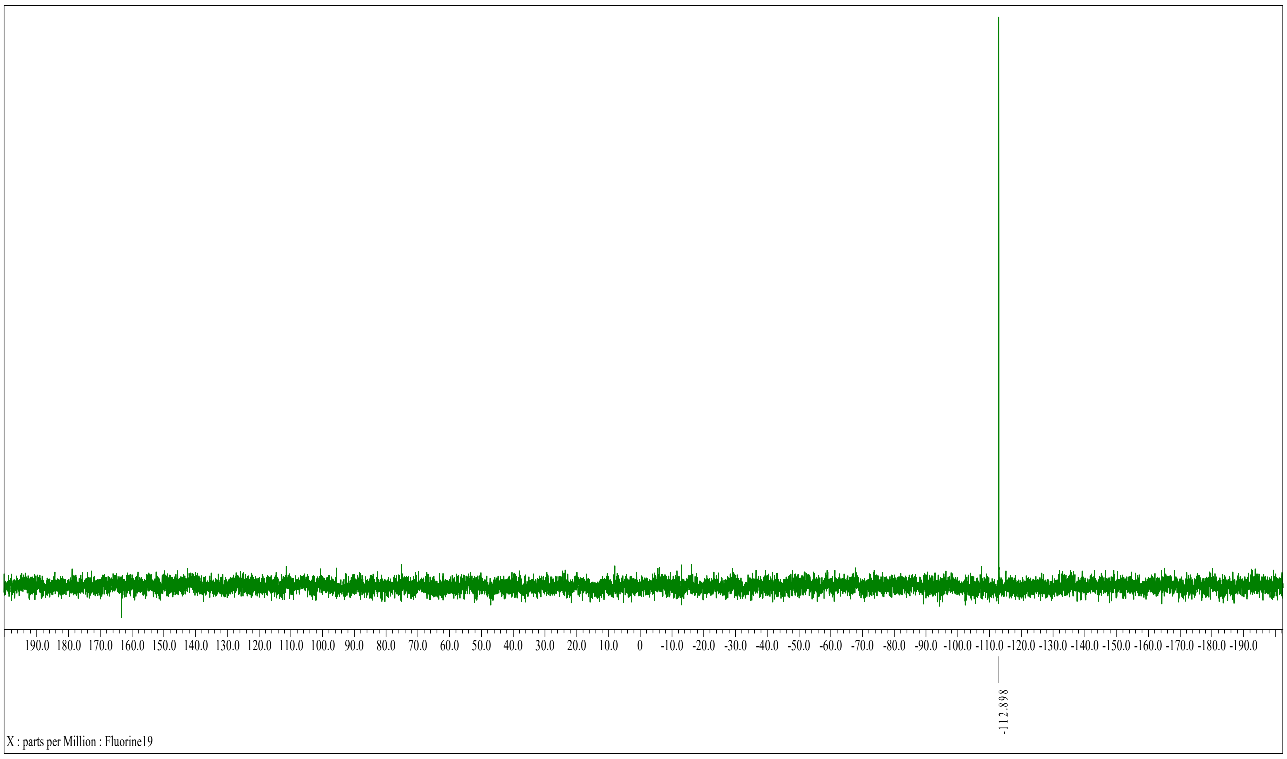

^{1}H NMR (400 MHz, $CDCl_3$) of 3bm

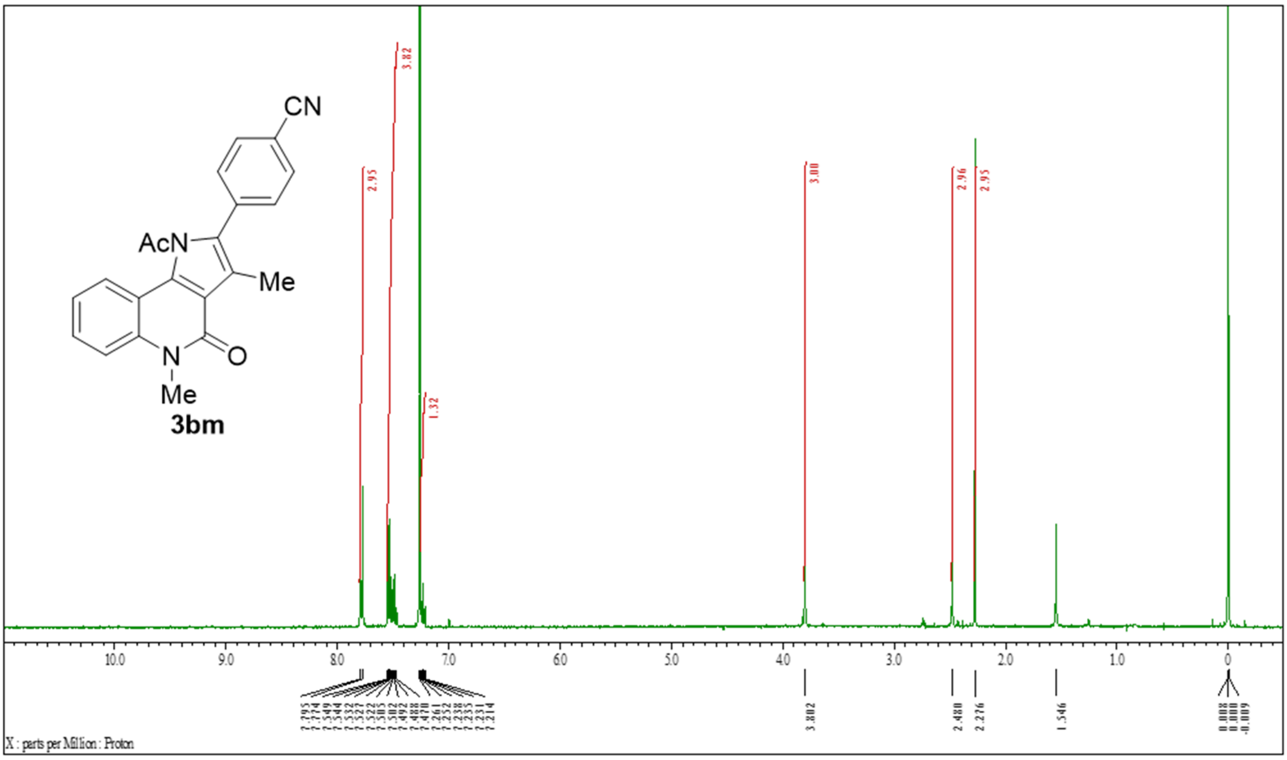

^{13}C NMR (100 MHz, $CDCl_3$) of 3bm

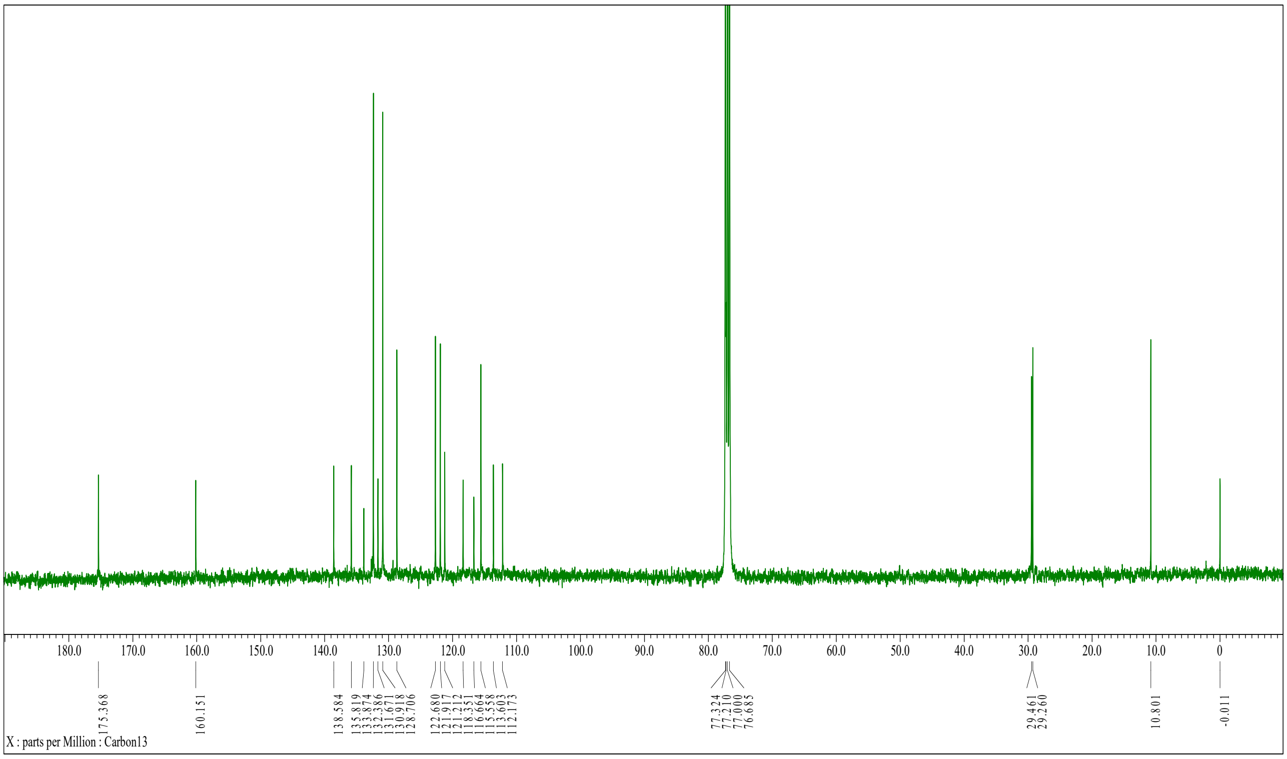

^{1}H NMR (400 MHz, $CDCl_3$) of 3bn

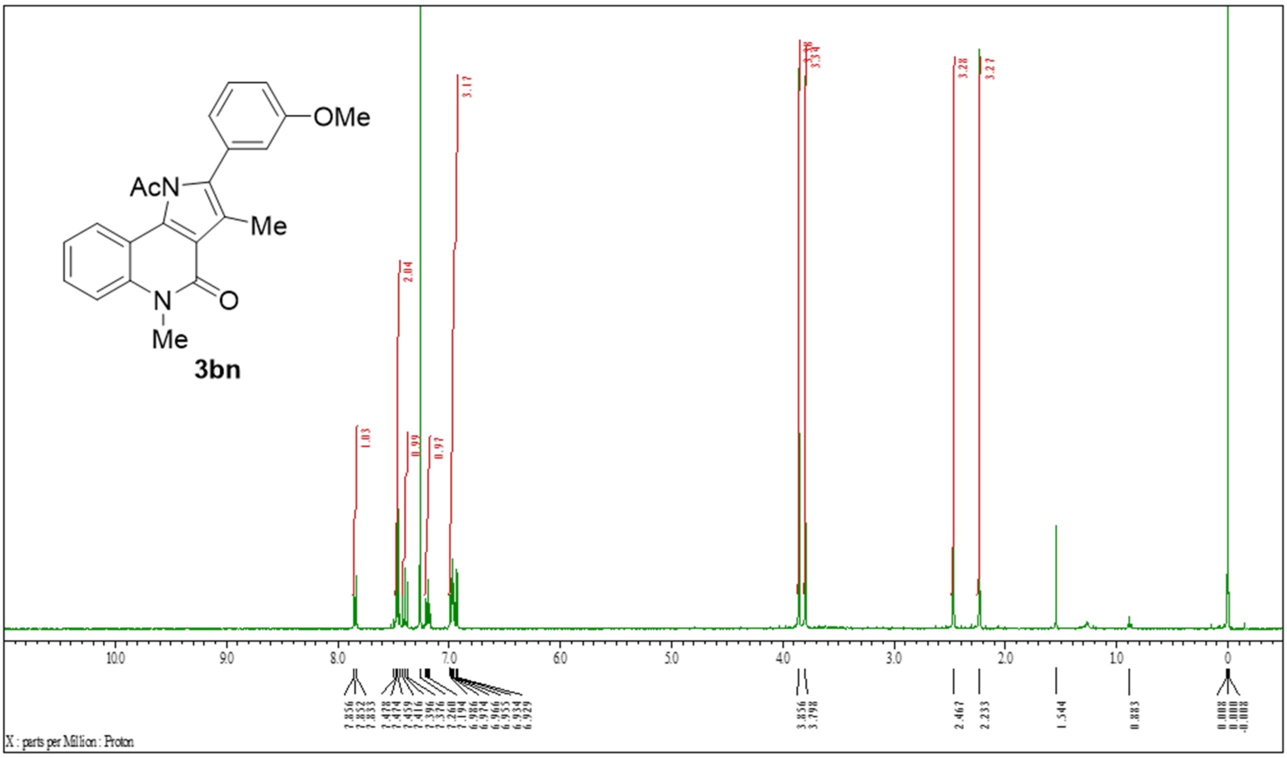

^{13}C NMR (100 MHz, $CDCl_3$) of 3bn

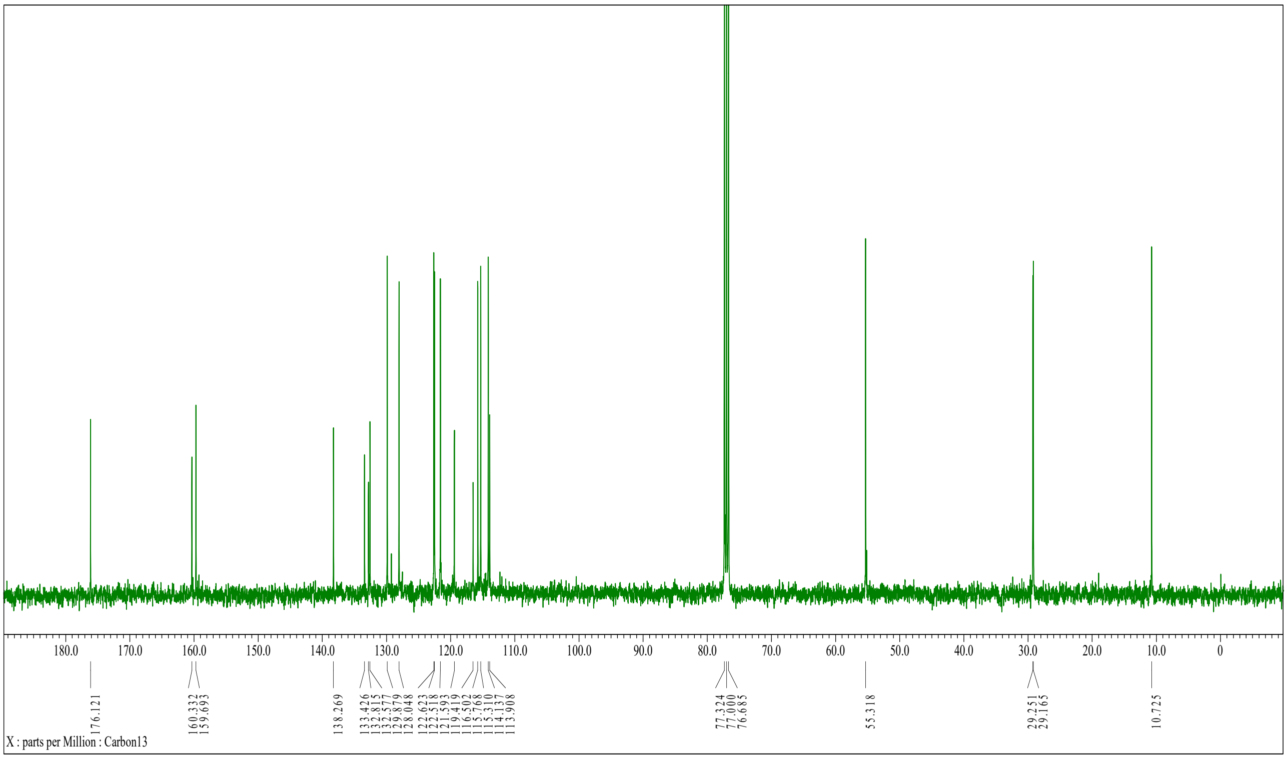

^{1}H NMR (400 MHz, $CDCl_3$) of 3bo

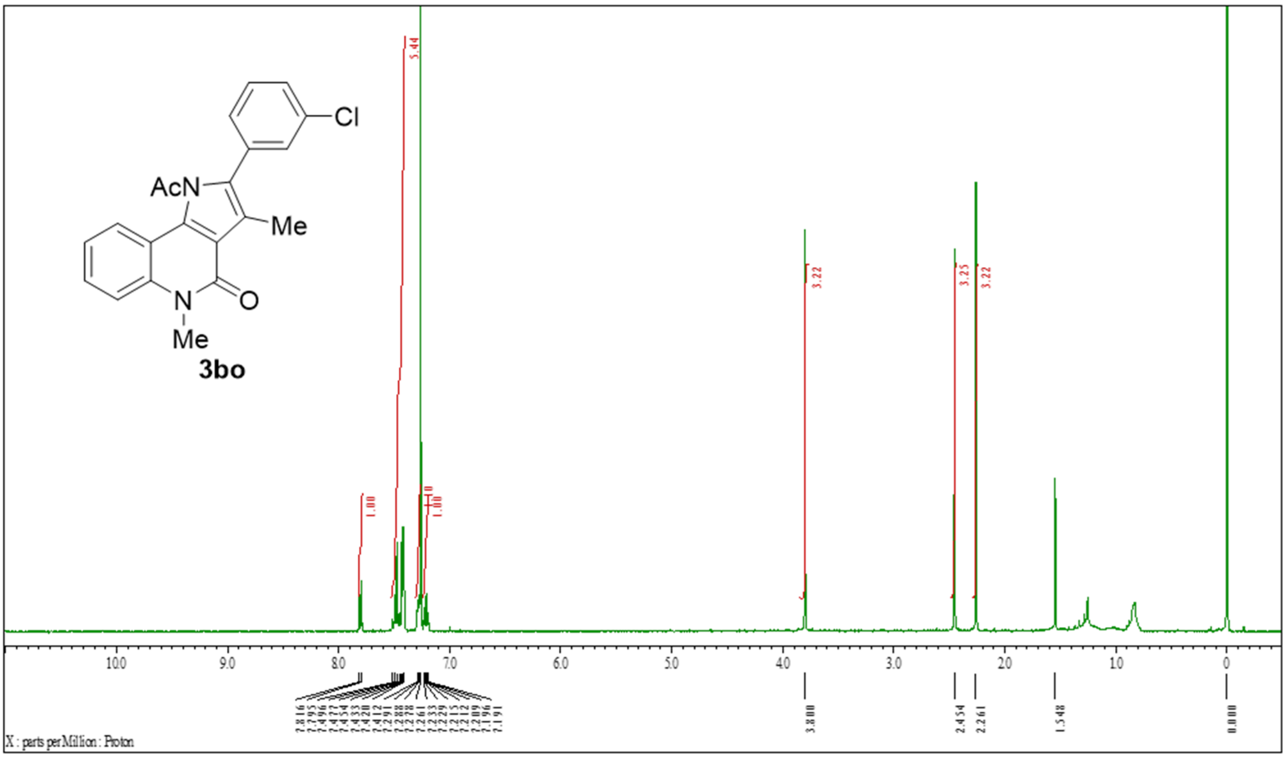

^{13}C NMR (100 MHz, $CDCl_3$) of 3bo

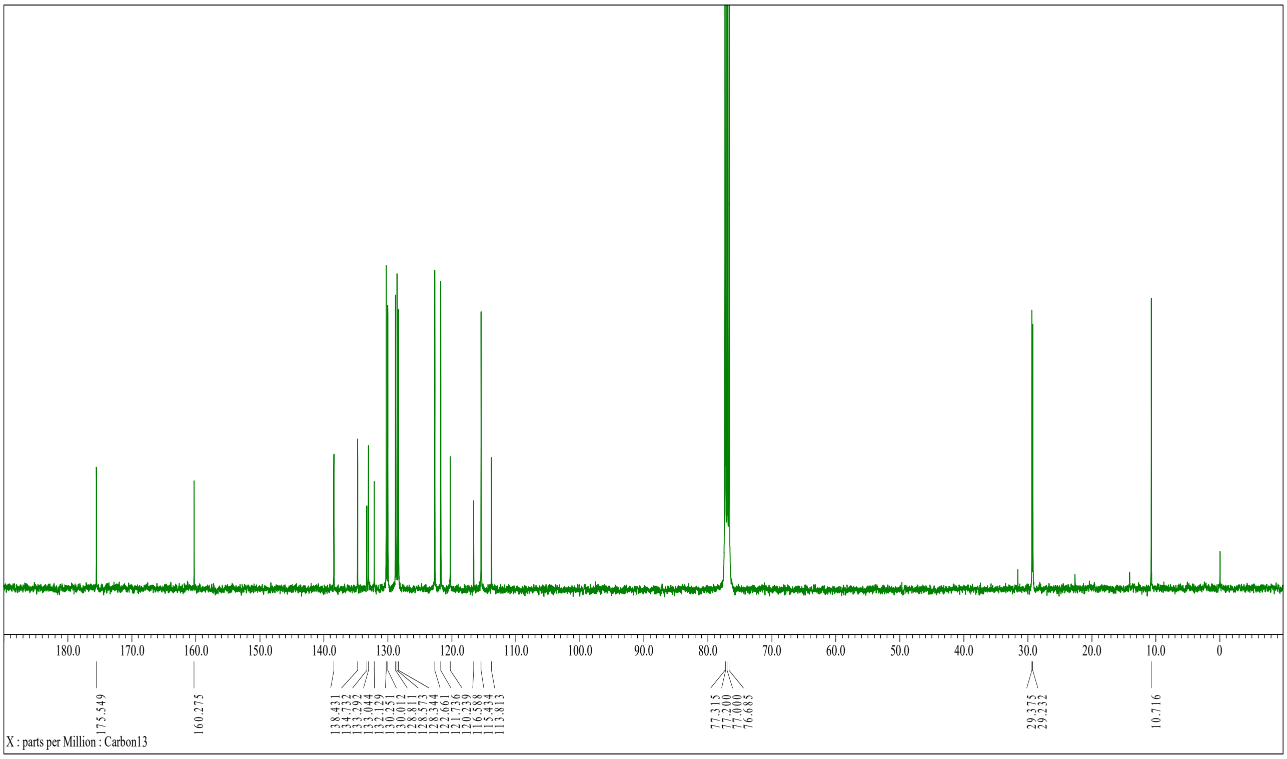

^{1}H NMR (400 MHz, $CDCl_3$) of 3bp

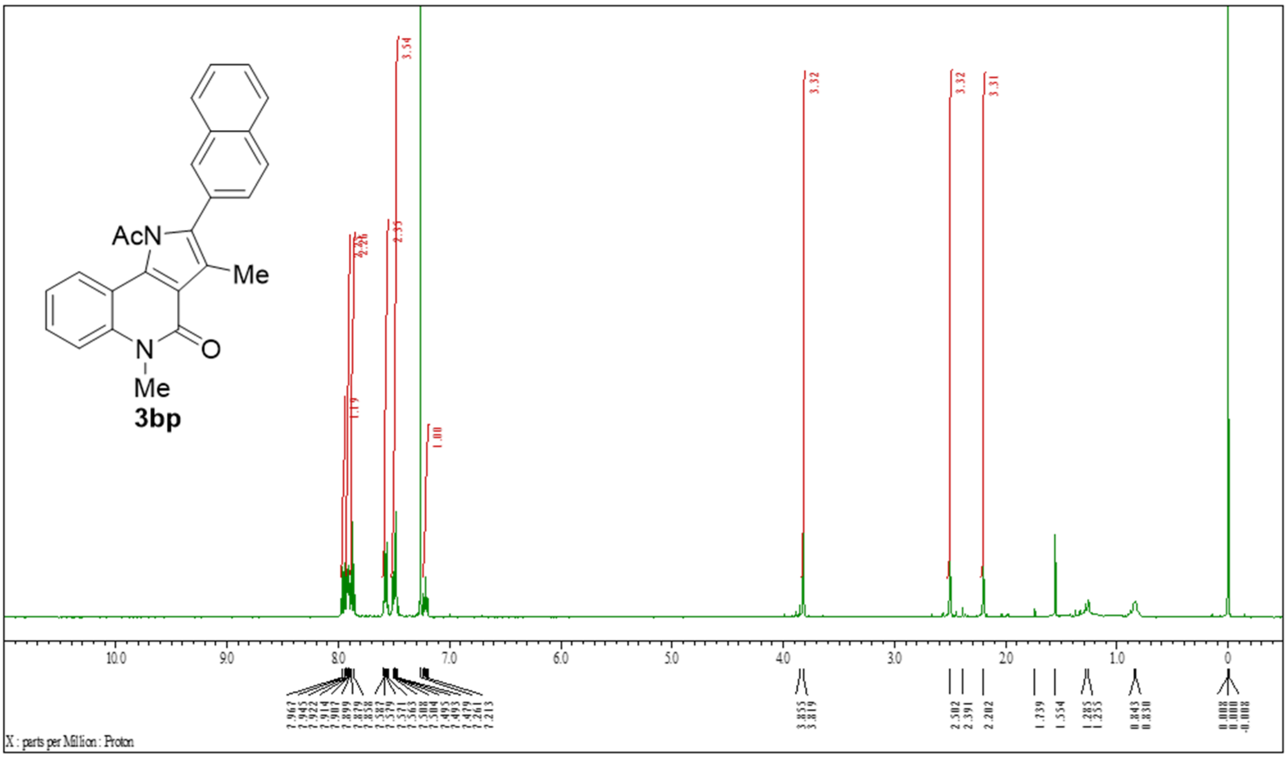

^{13}C NMR (100 MHz, $CDCl_3$) of 3bp

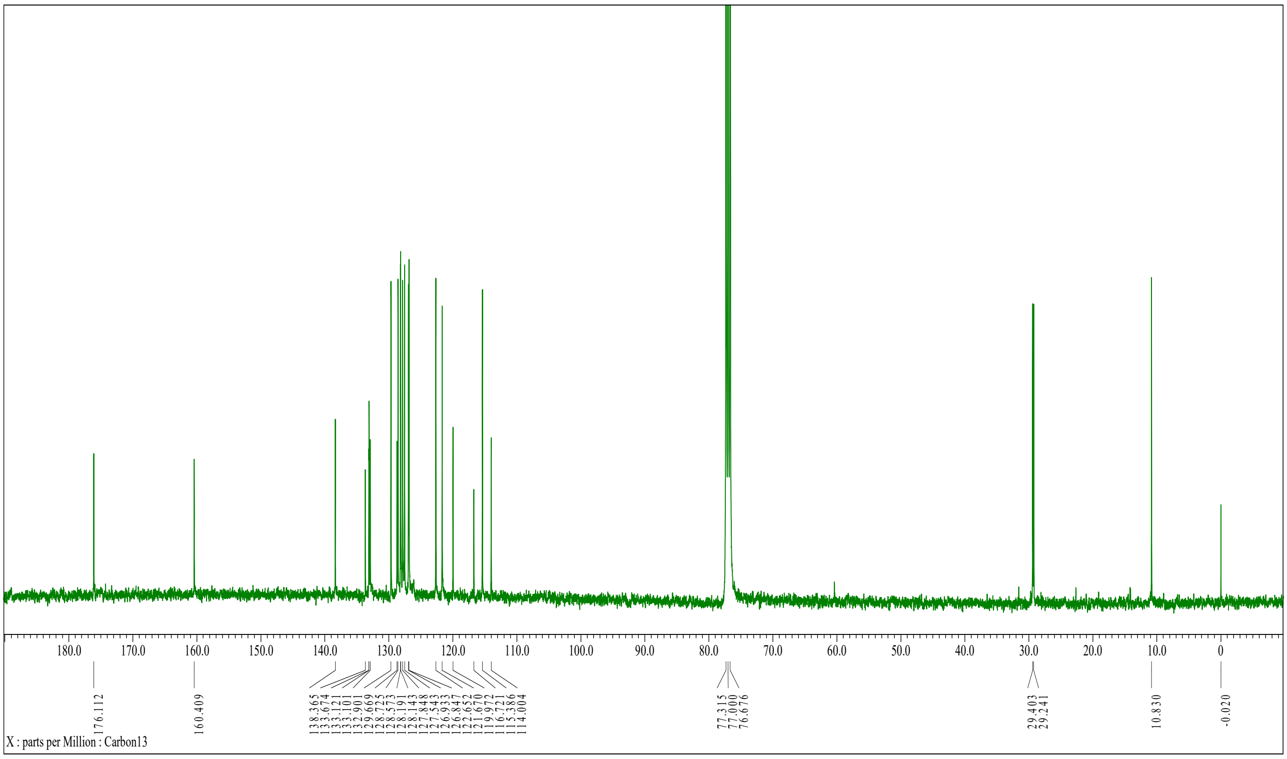

^{1}H NMR (400 MHz, $CDCl_3$) of 3bq

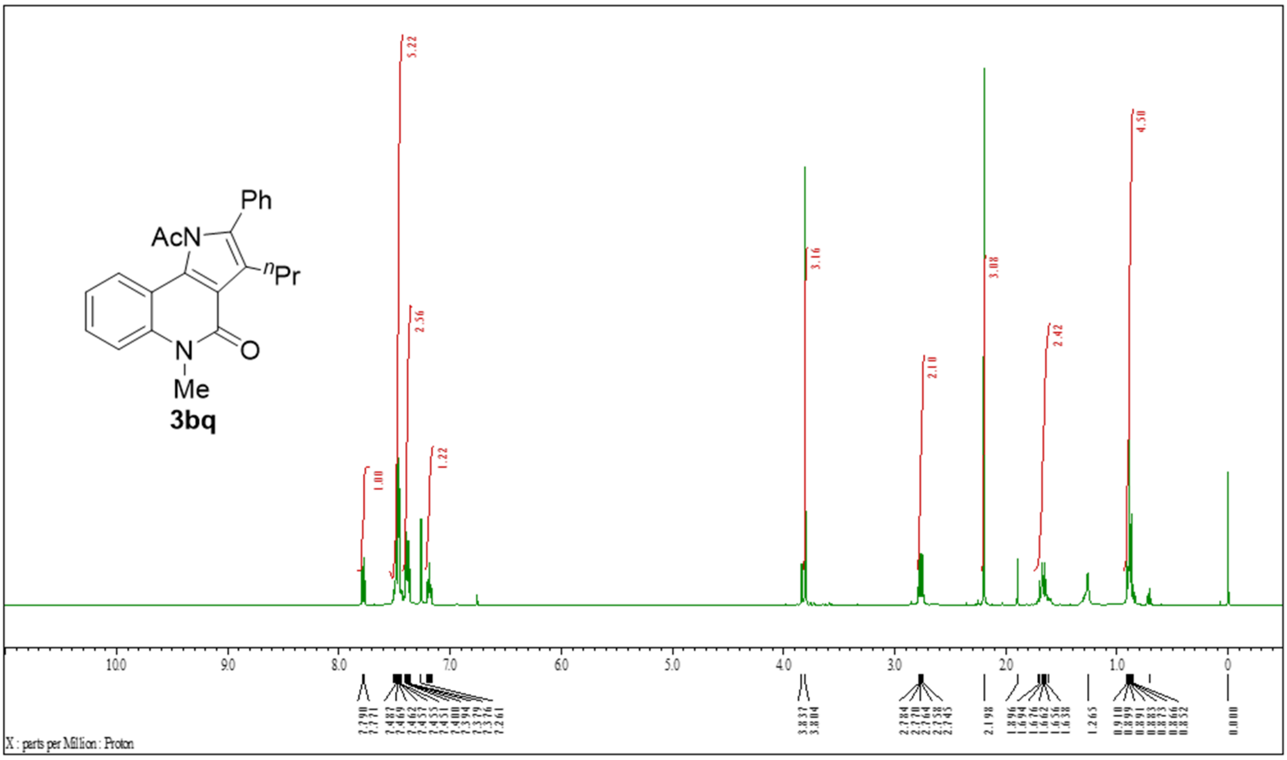

^{13}C NMR (100 MHz, $CDCl_3$) of 3bq

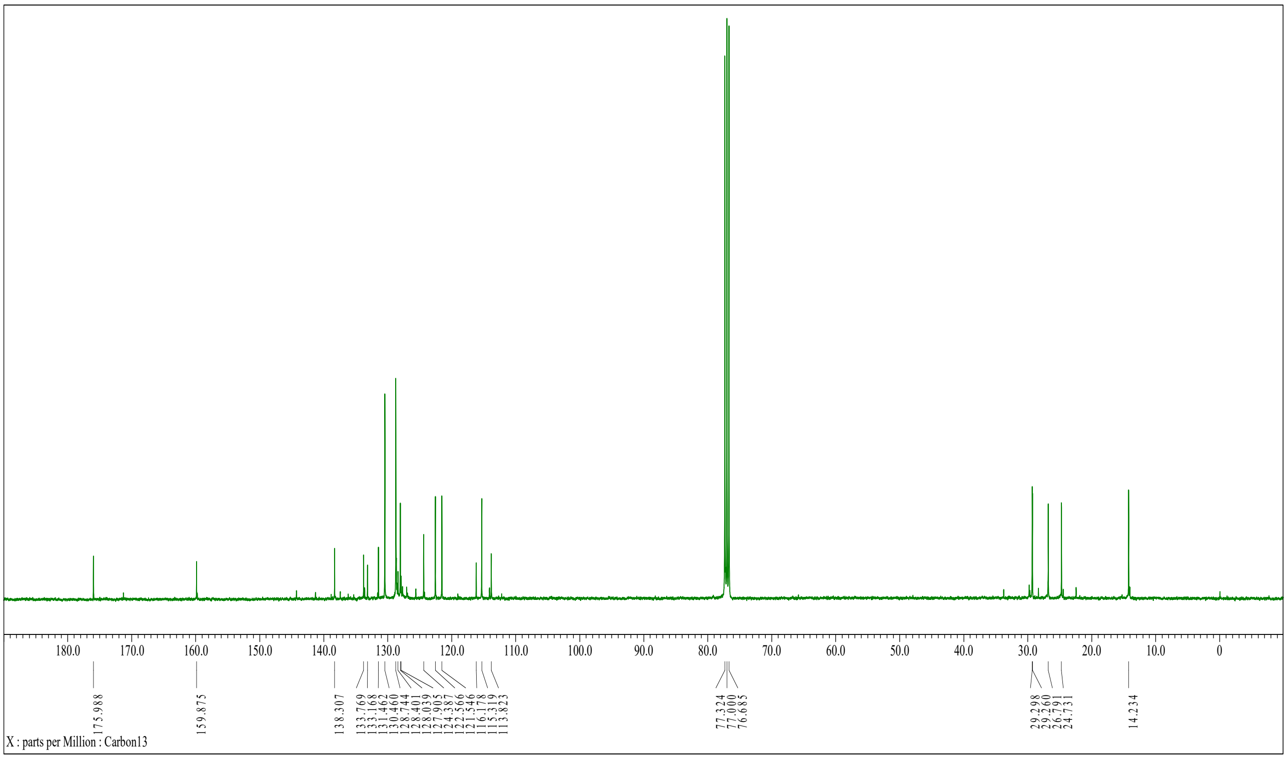

^{1}H NMR (400 MHz, $CDCl_3$) of 3br

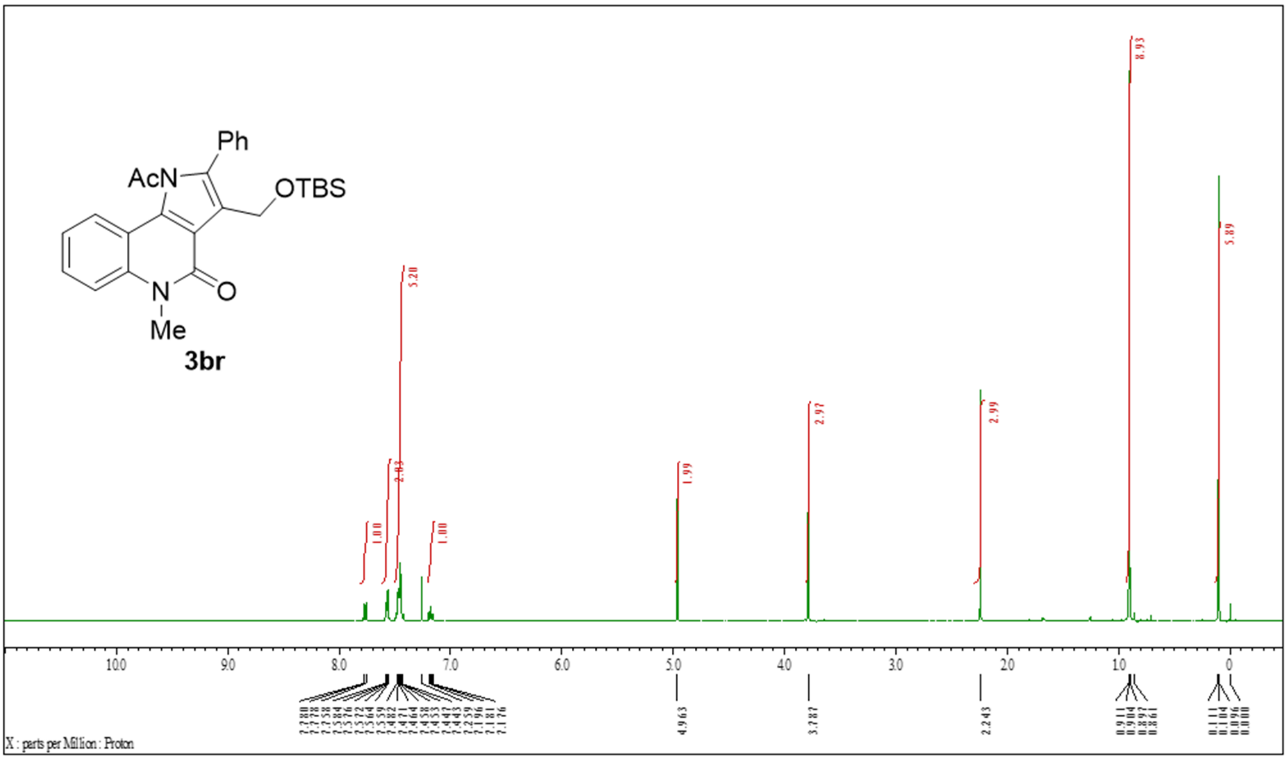

^{13}C NMR (100 MHz, $CDCl_3$) of 3br

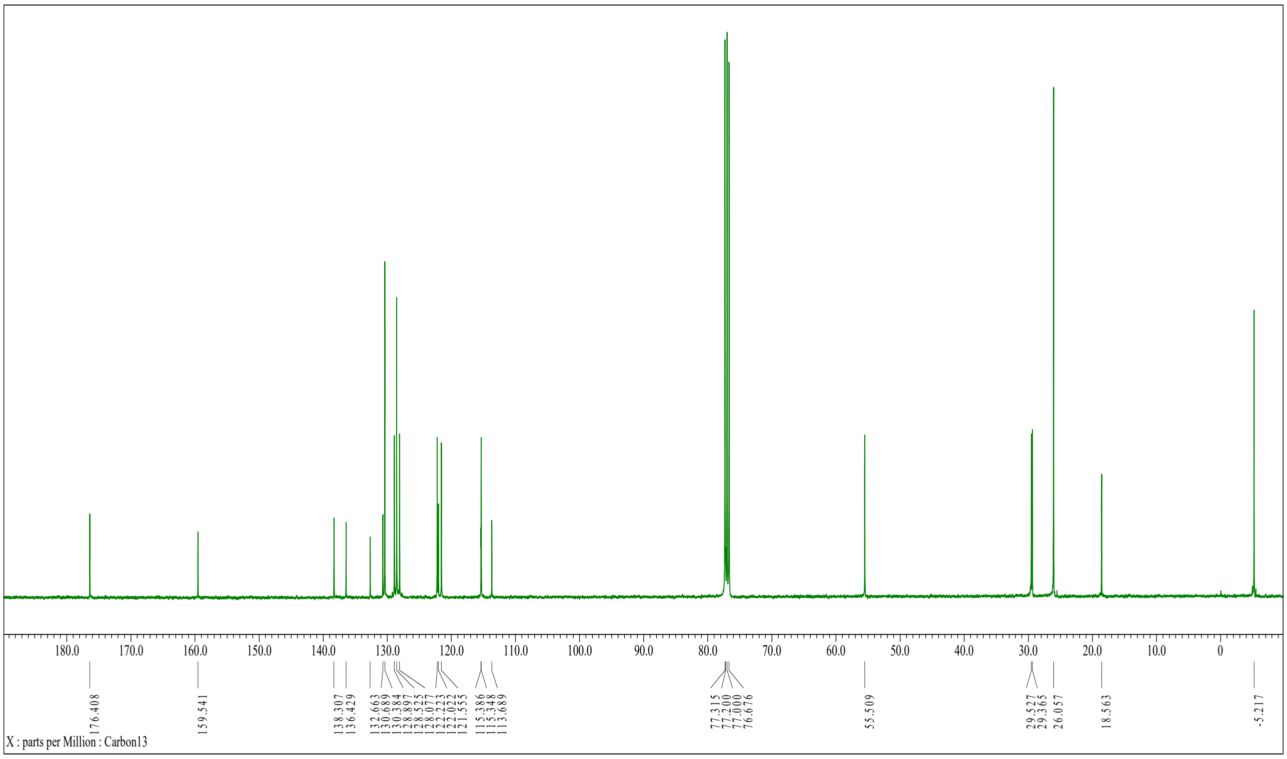

^{1}H NMR (400 MHz, $CDCl_3$) of 3bs

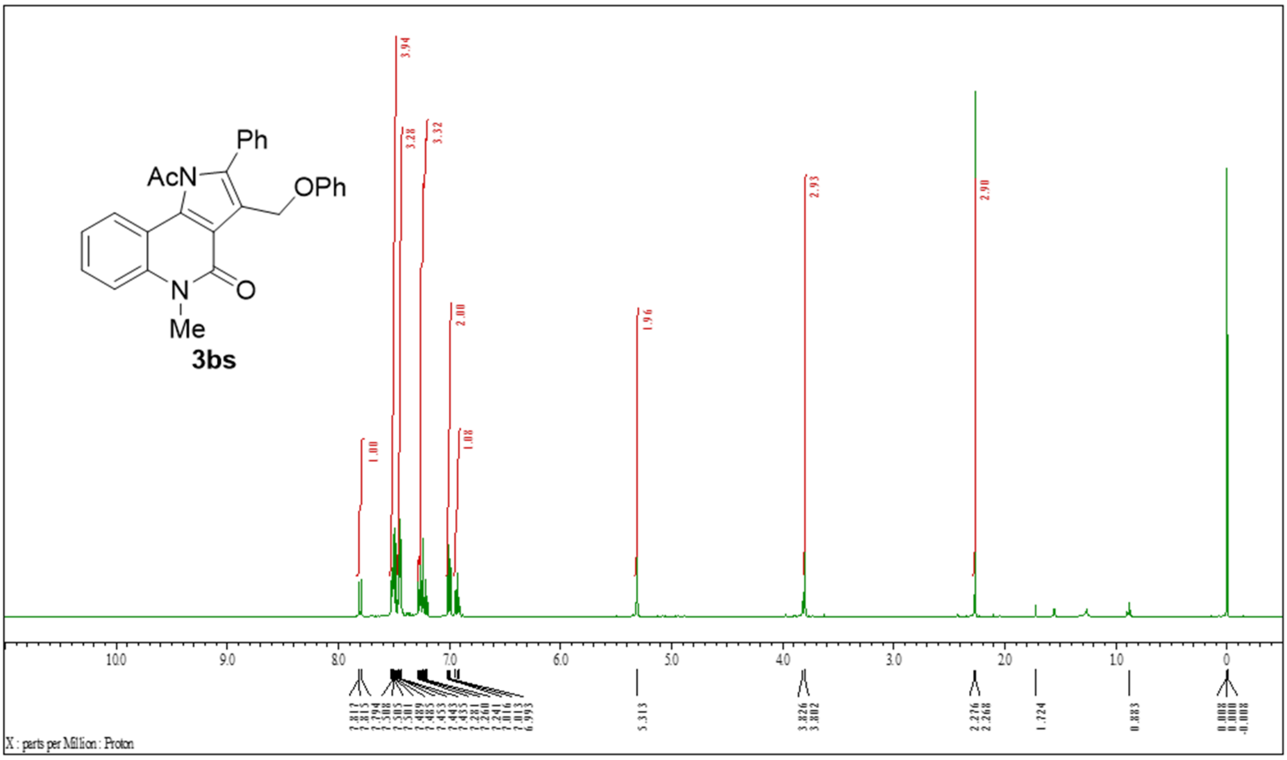

^{13}C NMR (100 MHz, $CDCl_3$) of 3bs

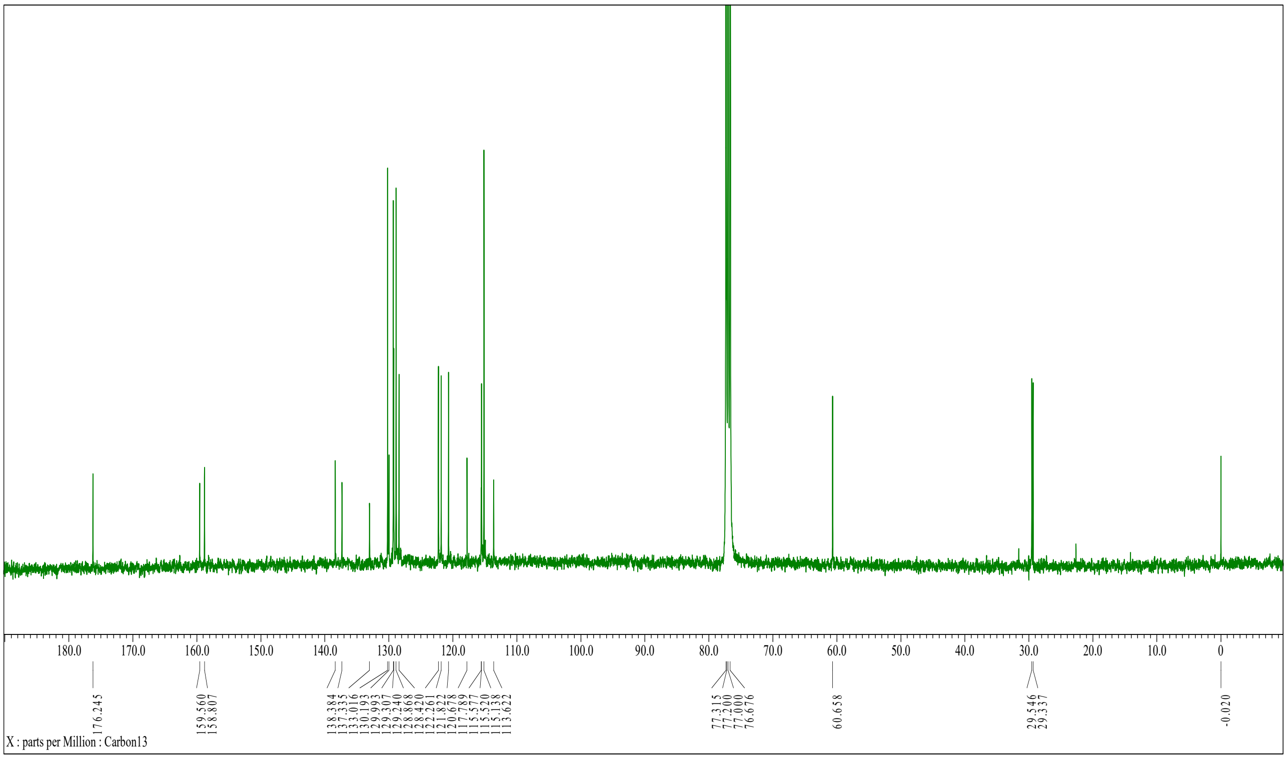

^{1}H NMR (400 MHz, CDCl$_3$) of 3bt

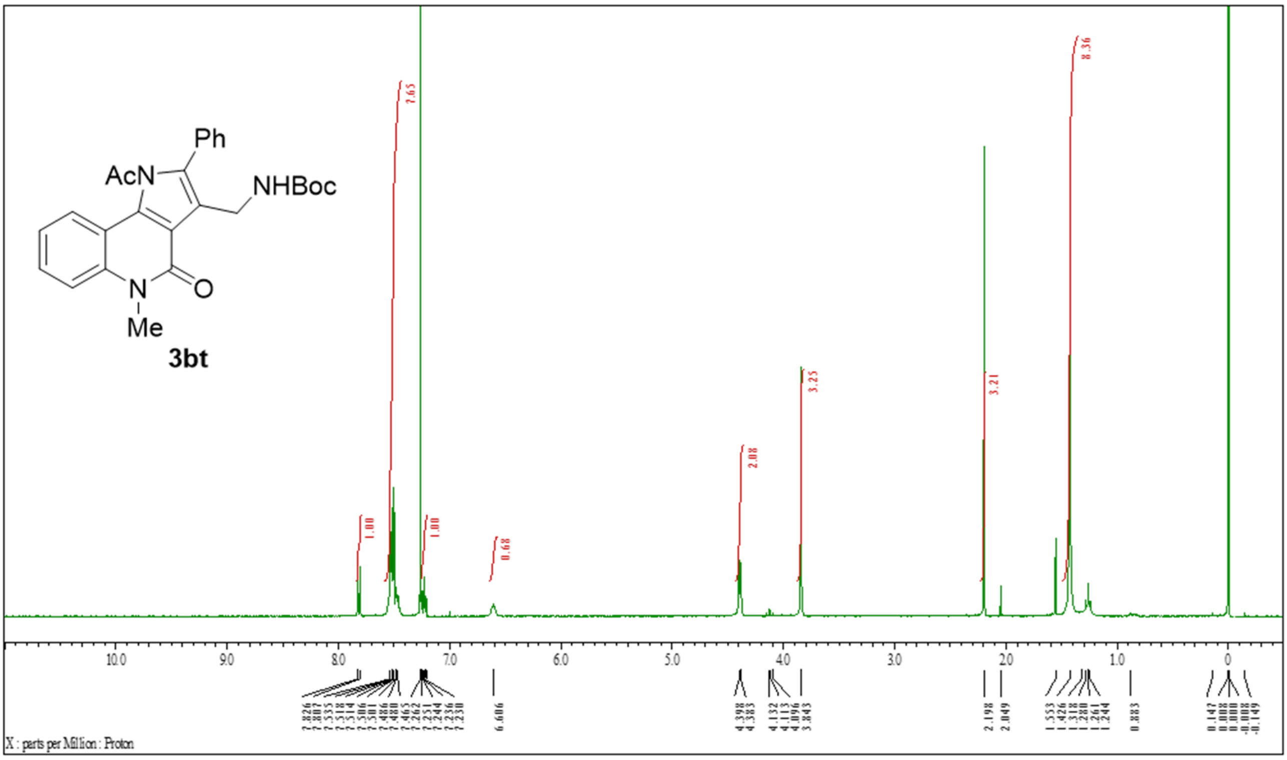

^{13}C NMR (100 MHz, CDCl$_3$) of 3bt

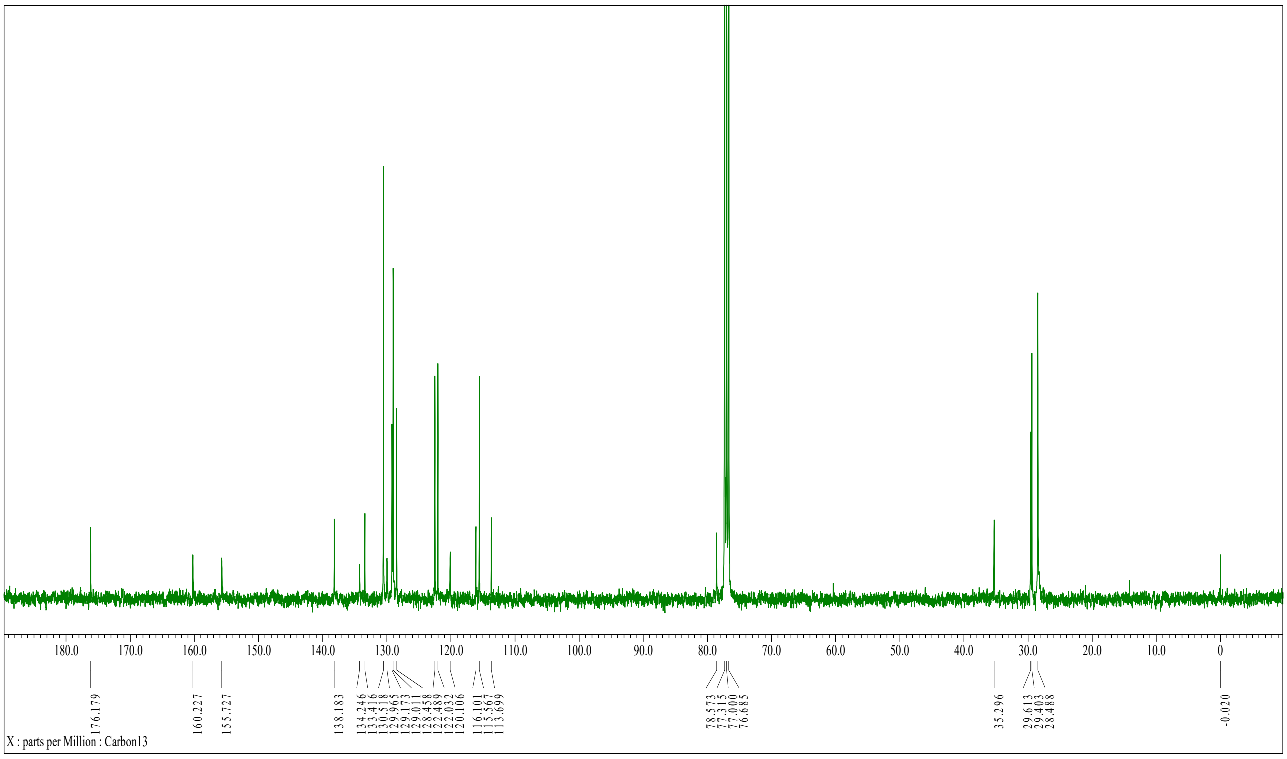

^{1}H NMR (400 MHz, $CDCl_3$) of 3bu

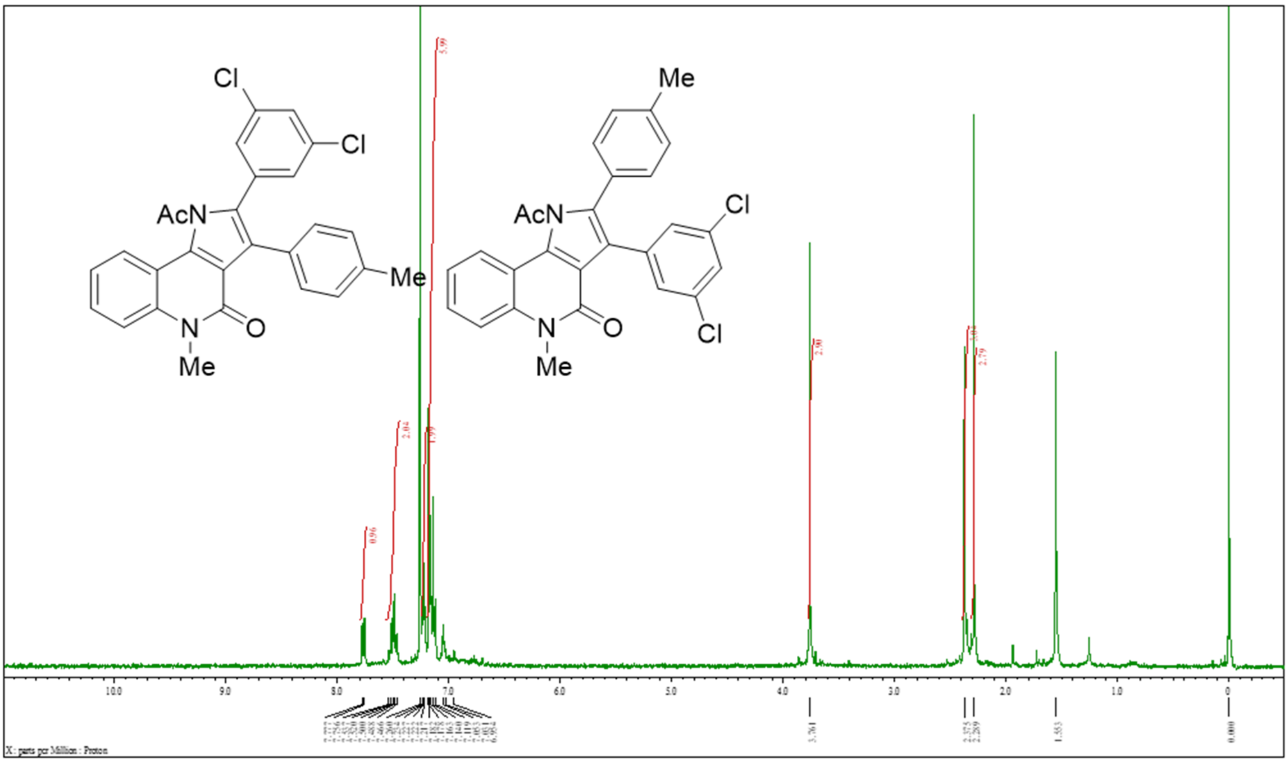

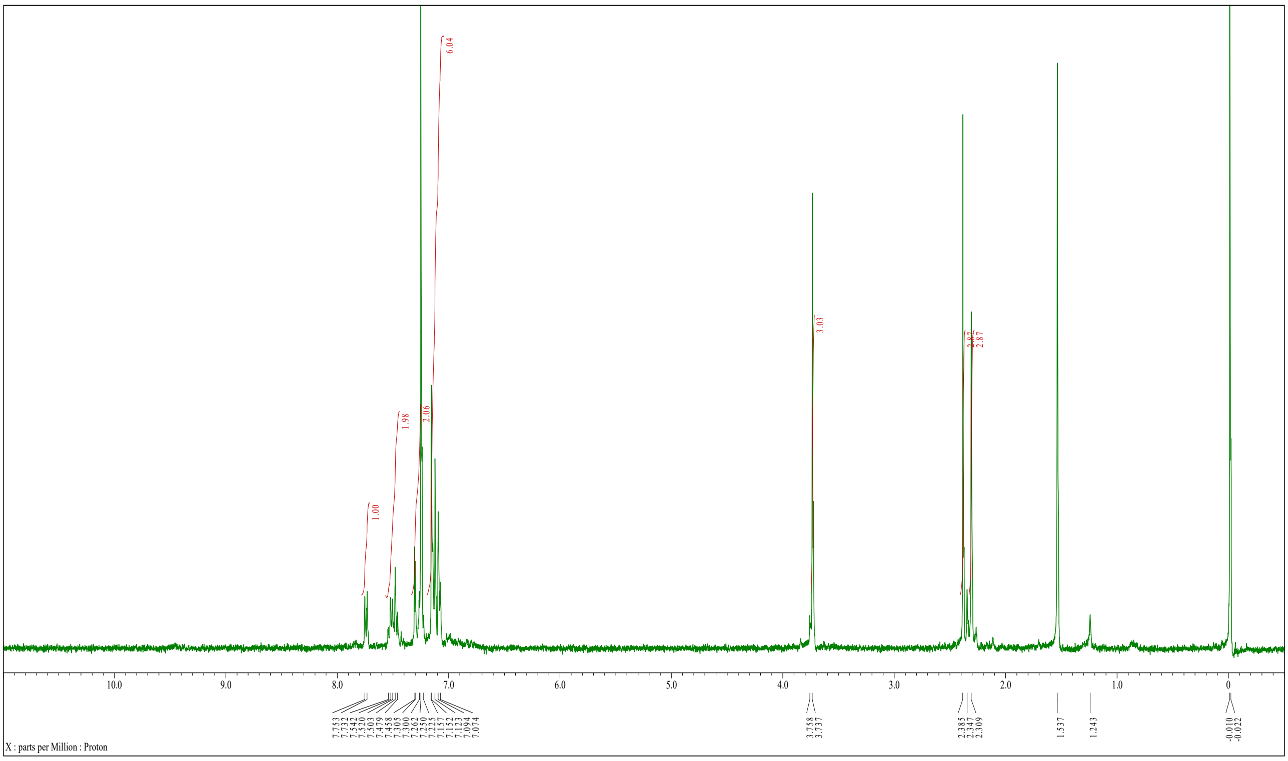

^{13}C NMR (100 MHz, $CDCl_3$) of 3bu

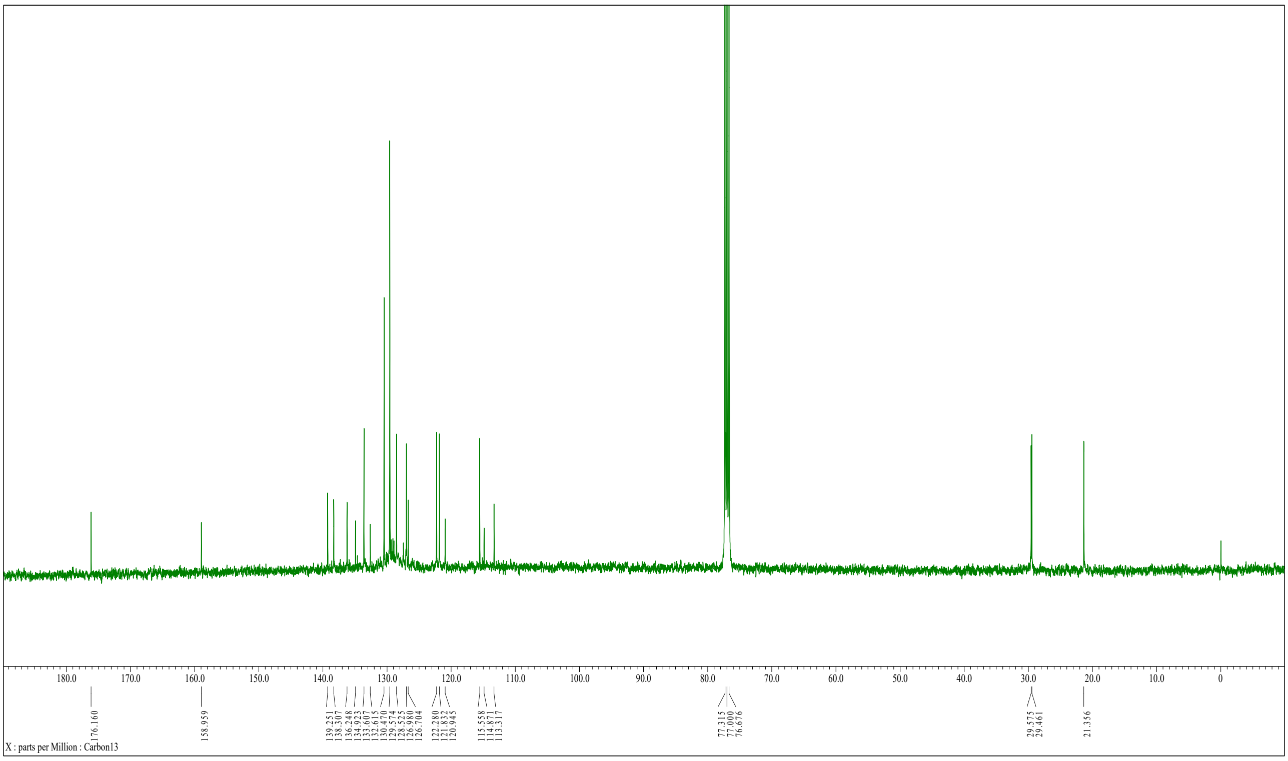

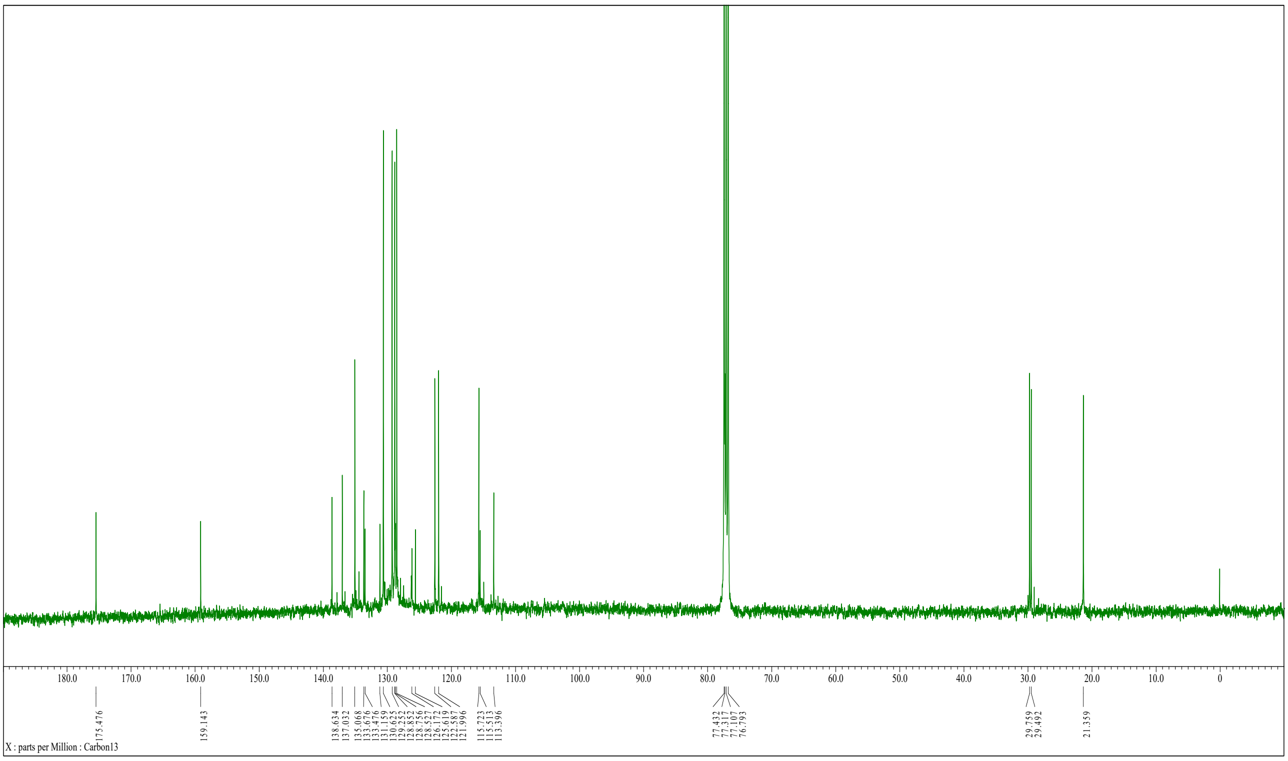

^{1}H NMR (400 MHz, $CDCl_3$) of 3ca

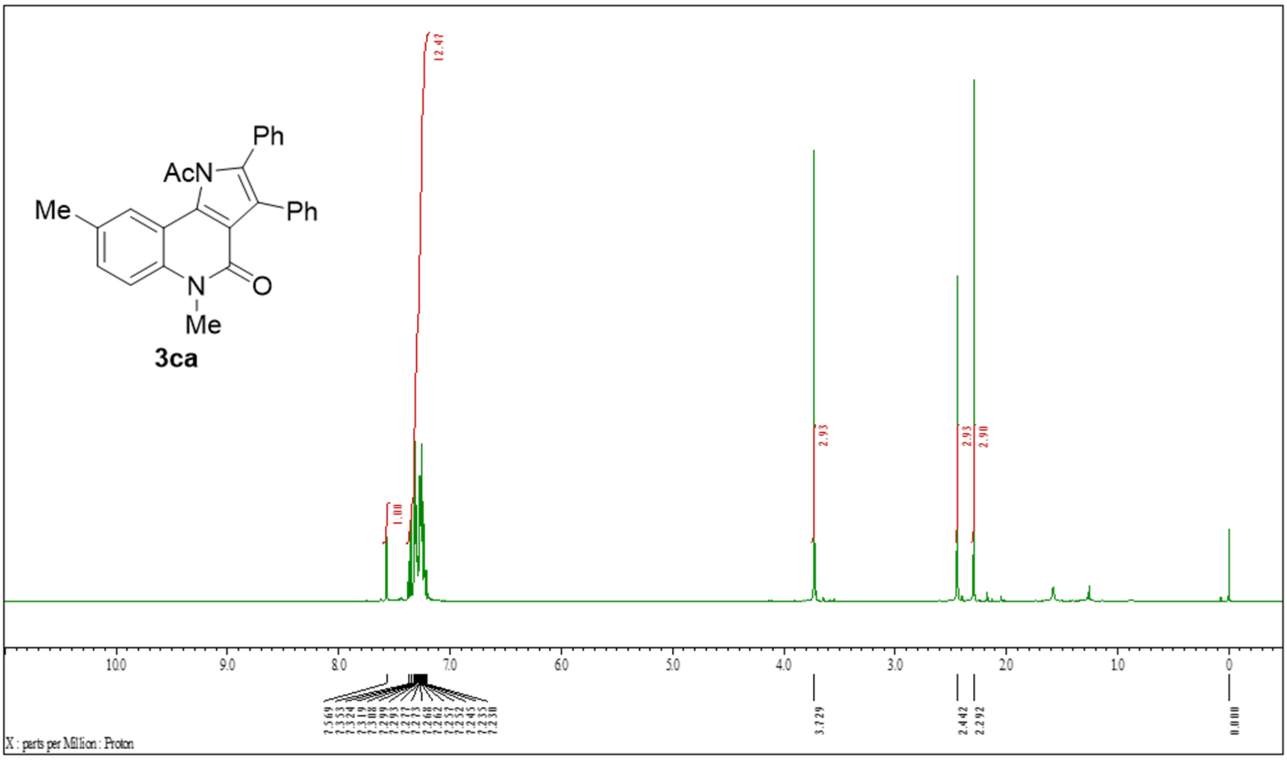

^{13}C NMR (100 MHz, $CDCl_3$) of 3ca

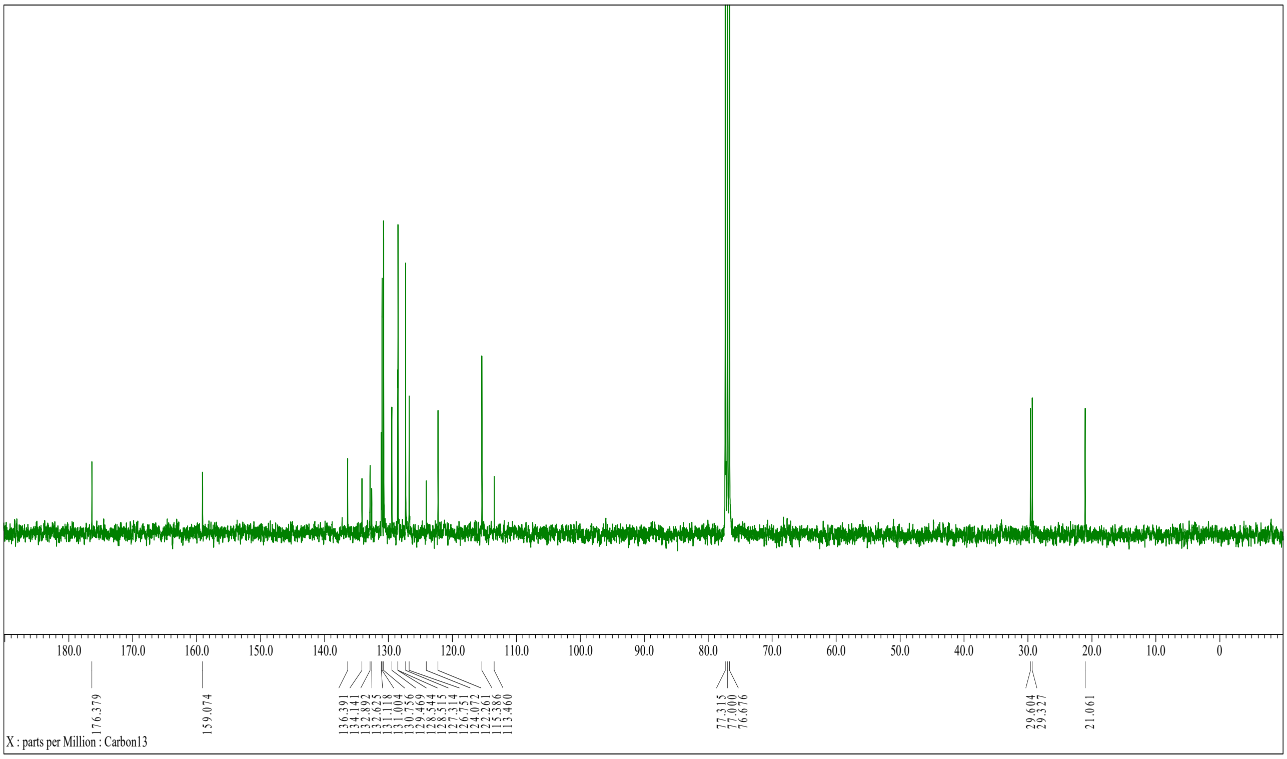

^{1}H NMR (400 MHz, $CDCl_3$) of 3da

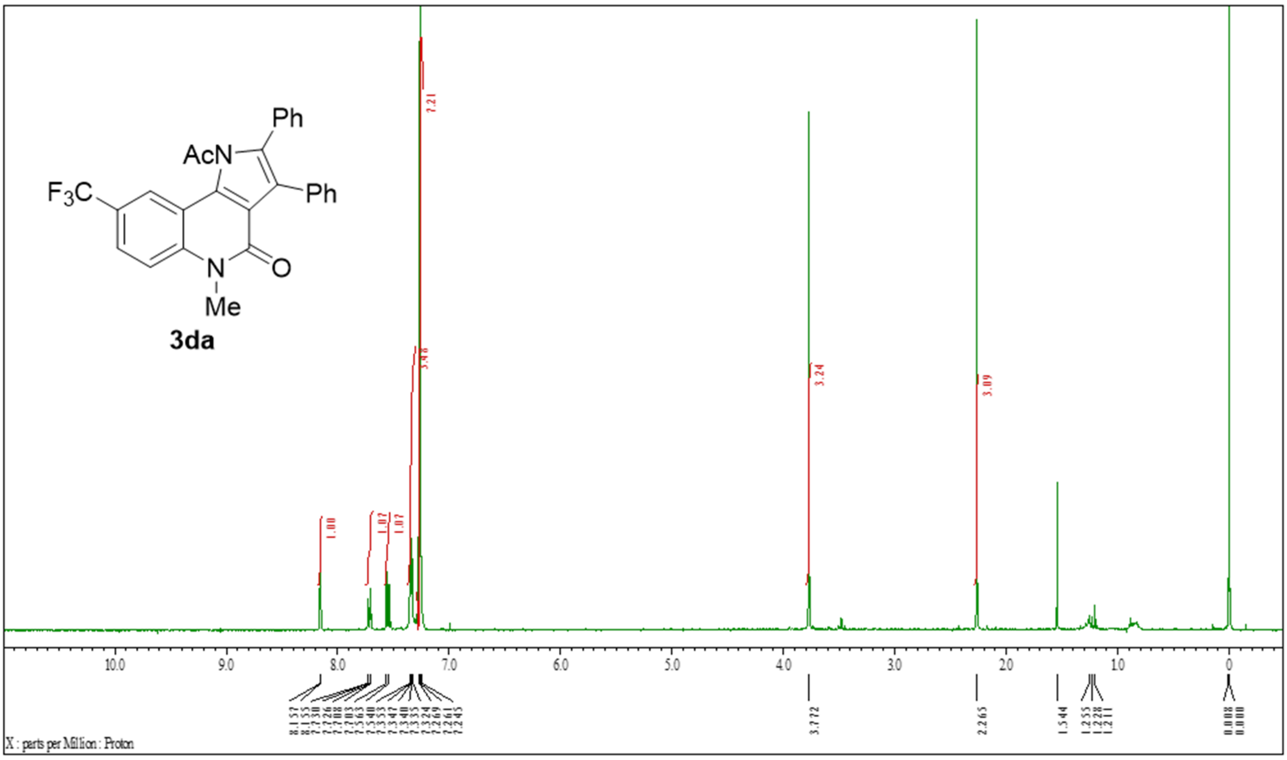

^{13}C NMR (100 MHz, $CDCl_3$) of 3da

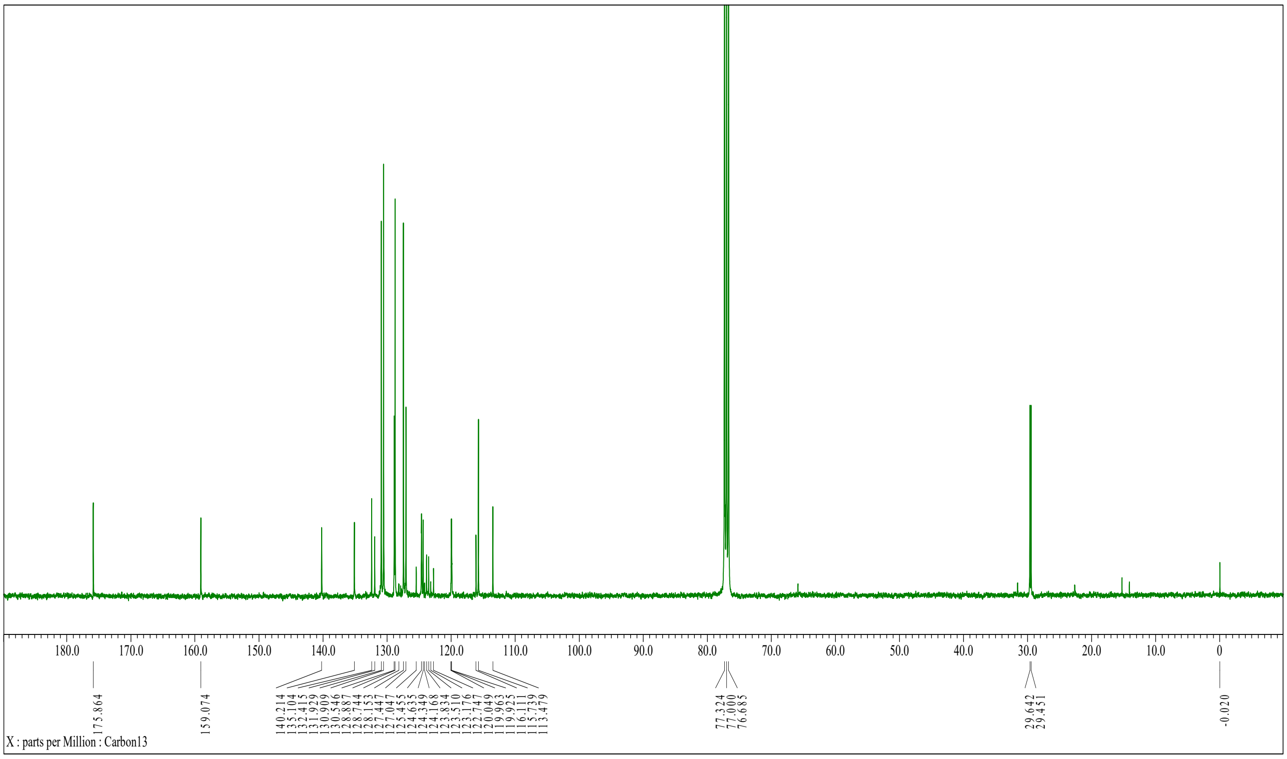

^{19}F NMR (376 MHz, $CDCl_3$) of 3da

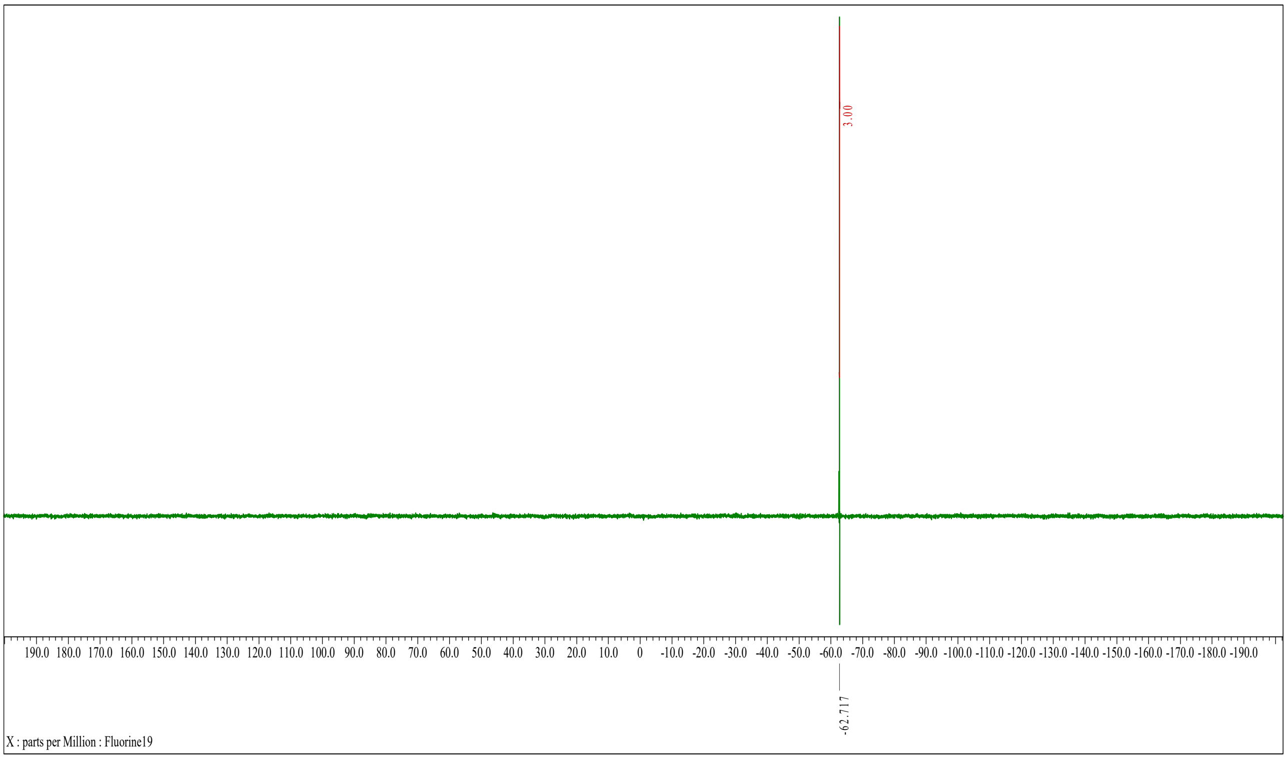

^{1}H NMR (400 MHz, $CDCl_3$) of 3ea

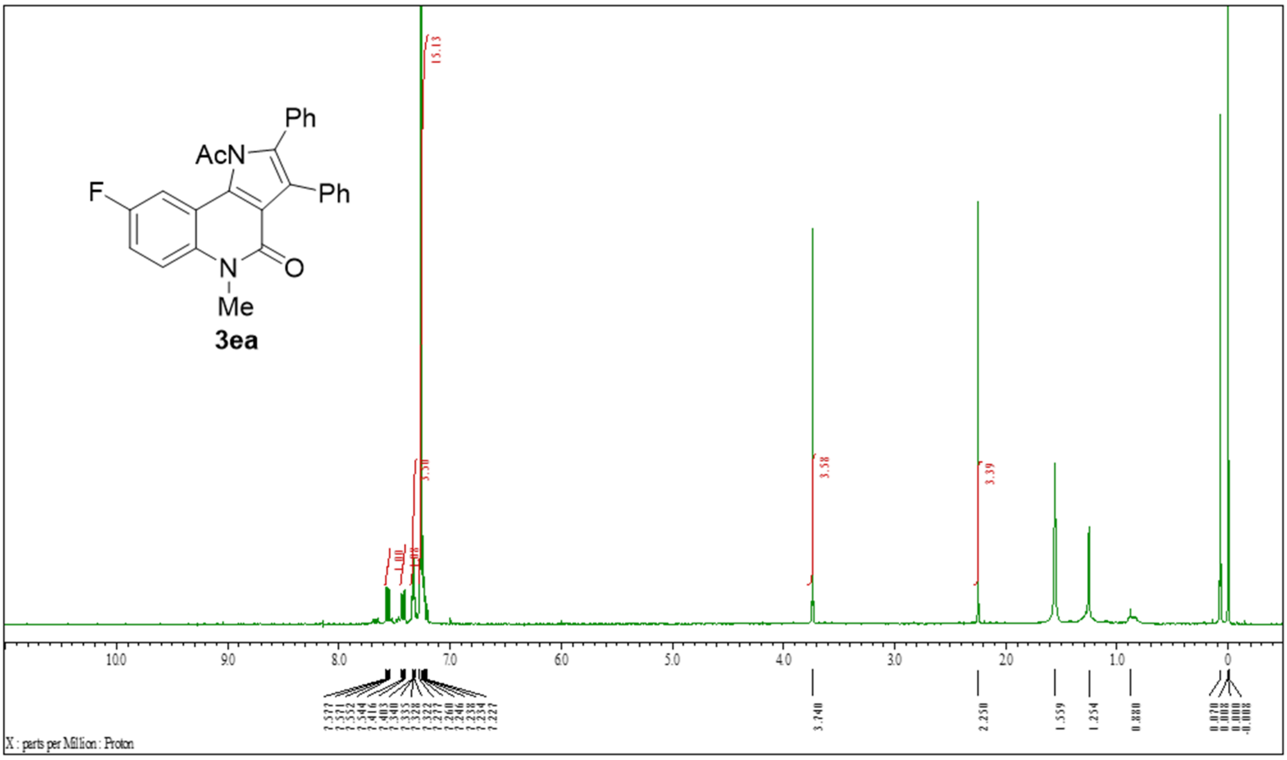

^{13}C NMR (100 MHz, $CDCl_3$) of 3ea

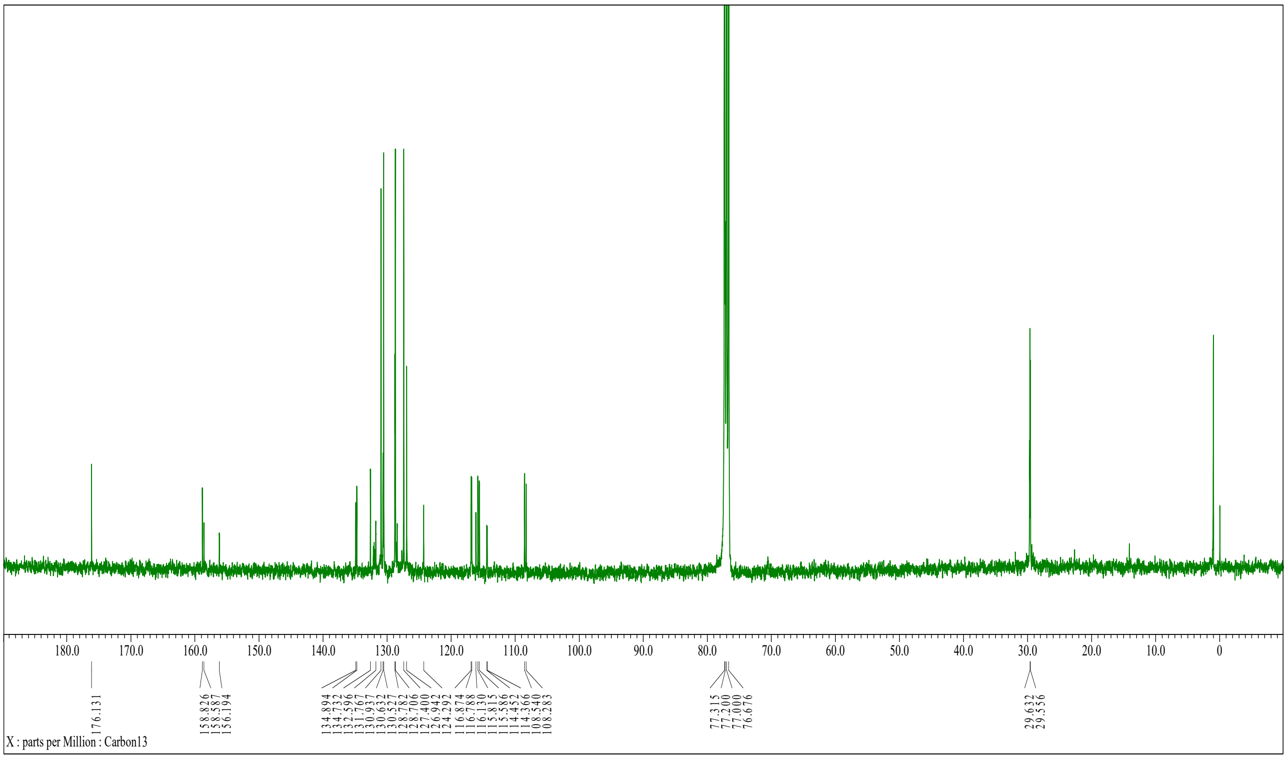

^{19}F NMR (376 MHz, $CDCl_3$) of 3ea

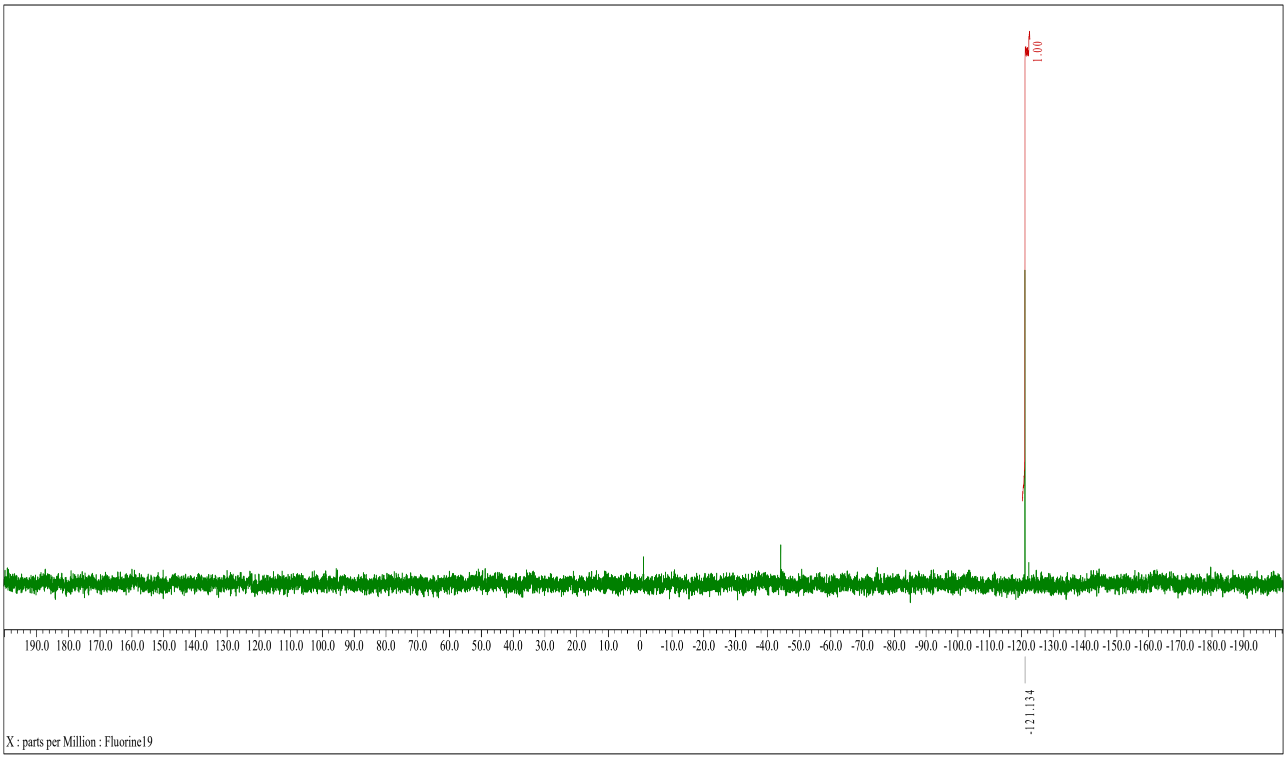

^{1}H NMR (400 MHz, CDCl$_3$) of 3fa

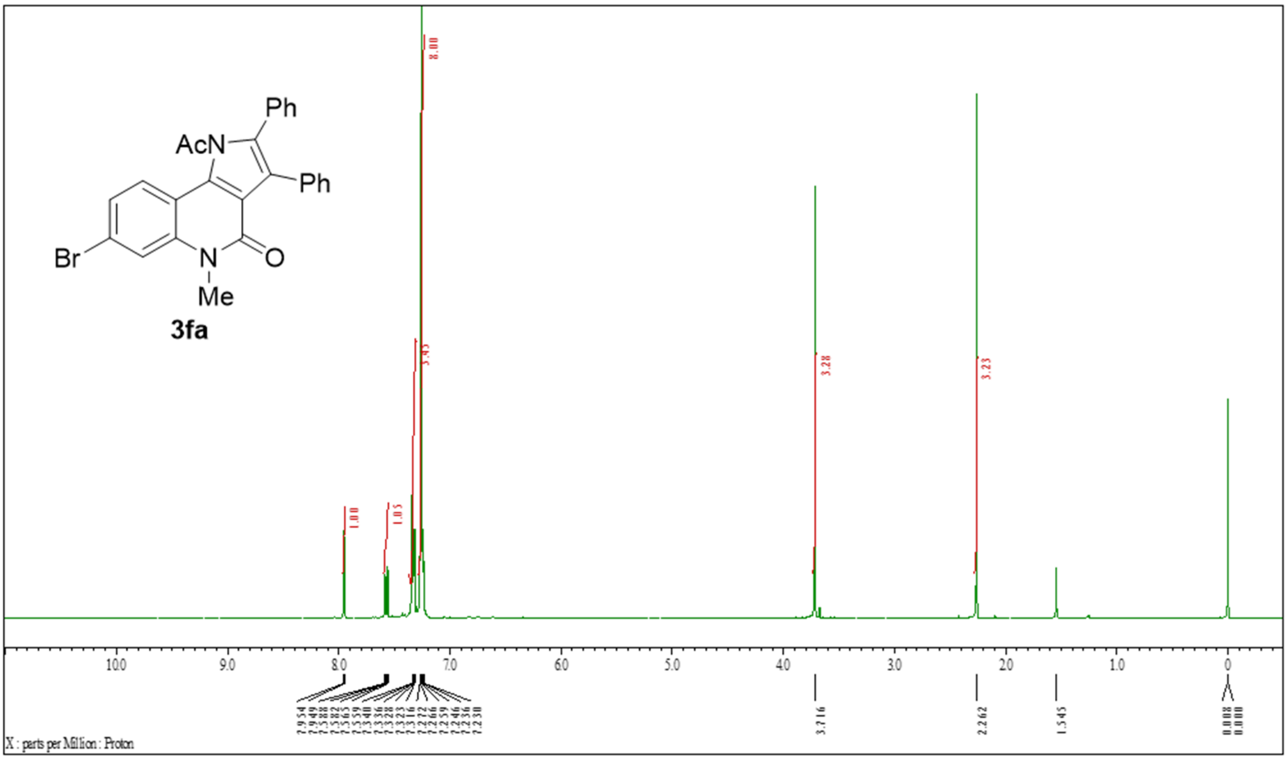

^{13}C NMR (100 MHz, CDCl$_3$) of 3fa

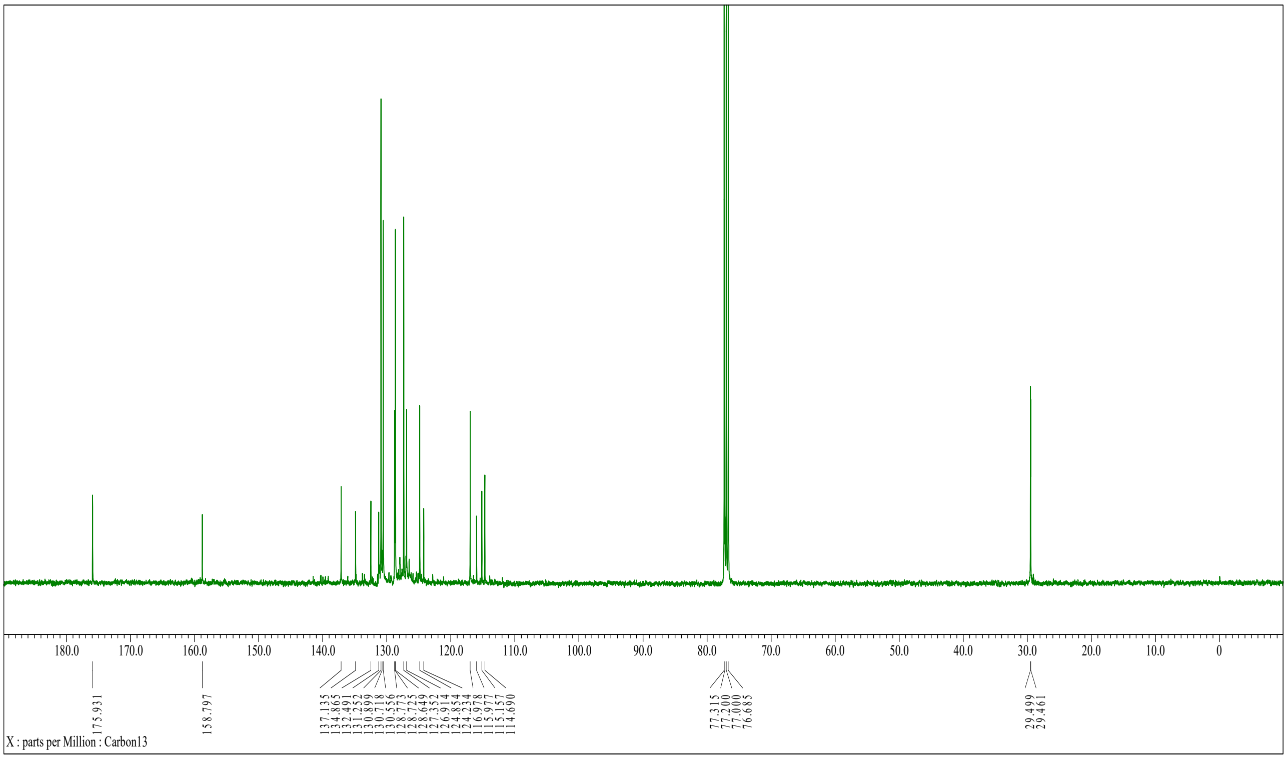

^{1}H NMR (400 MHz, $CDCl_3$) of 3ga

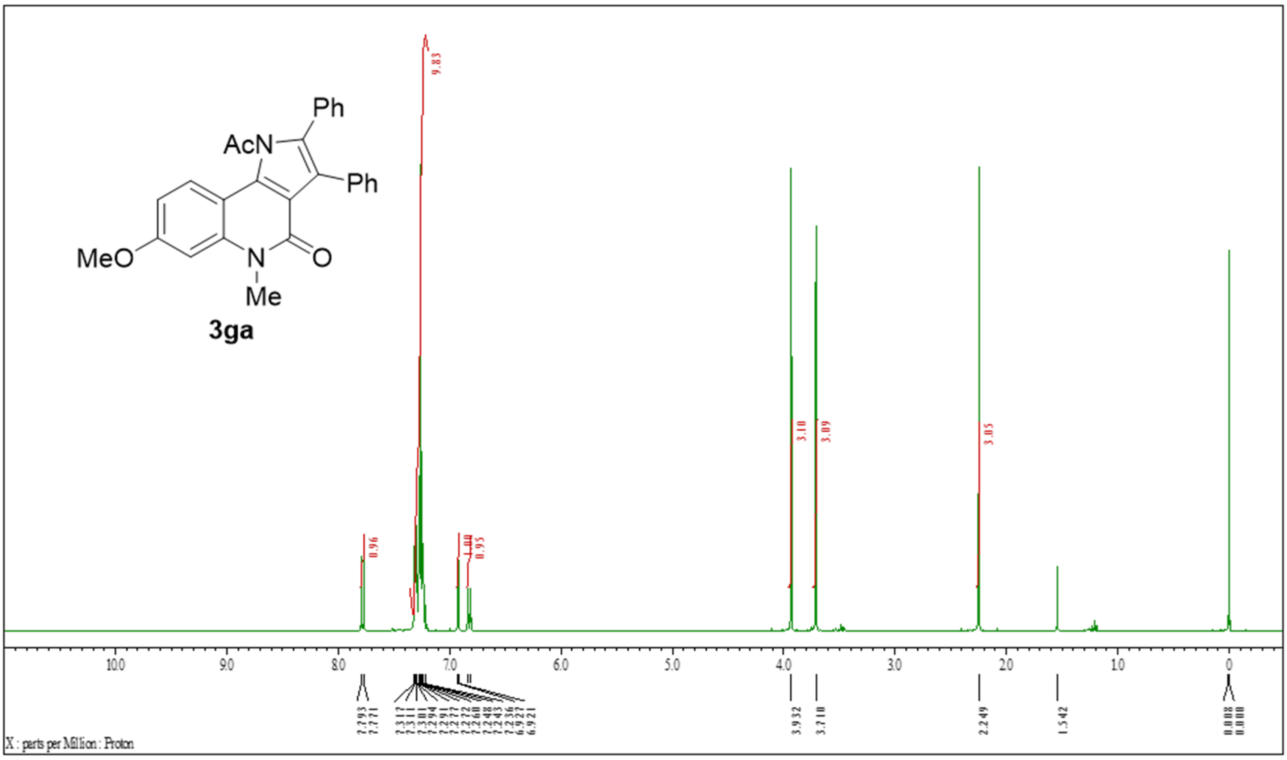

^{13}C NMR (100 MHz, $CDCl_3$) of 3ga

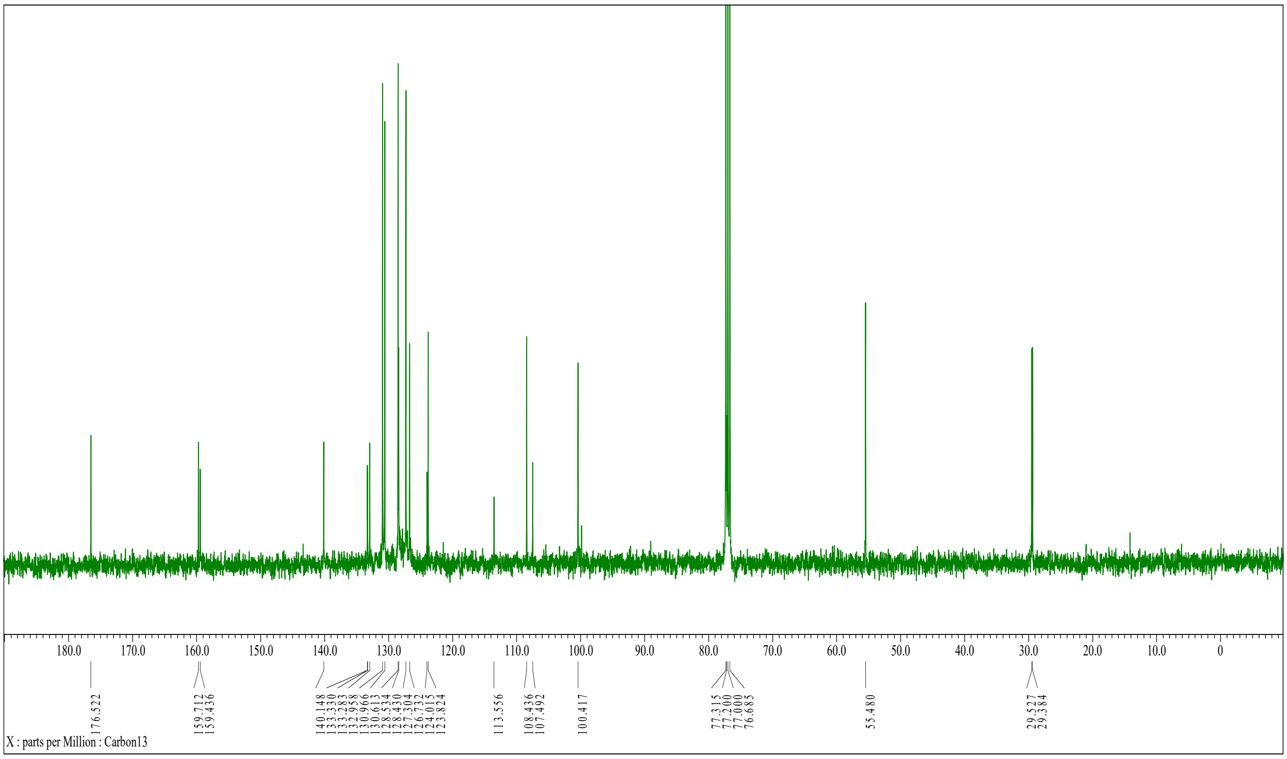

^{1}H NMR (400 MHz, $CDCl_3$) of 3ba'

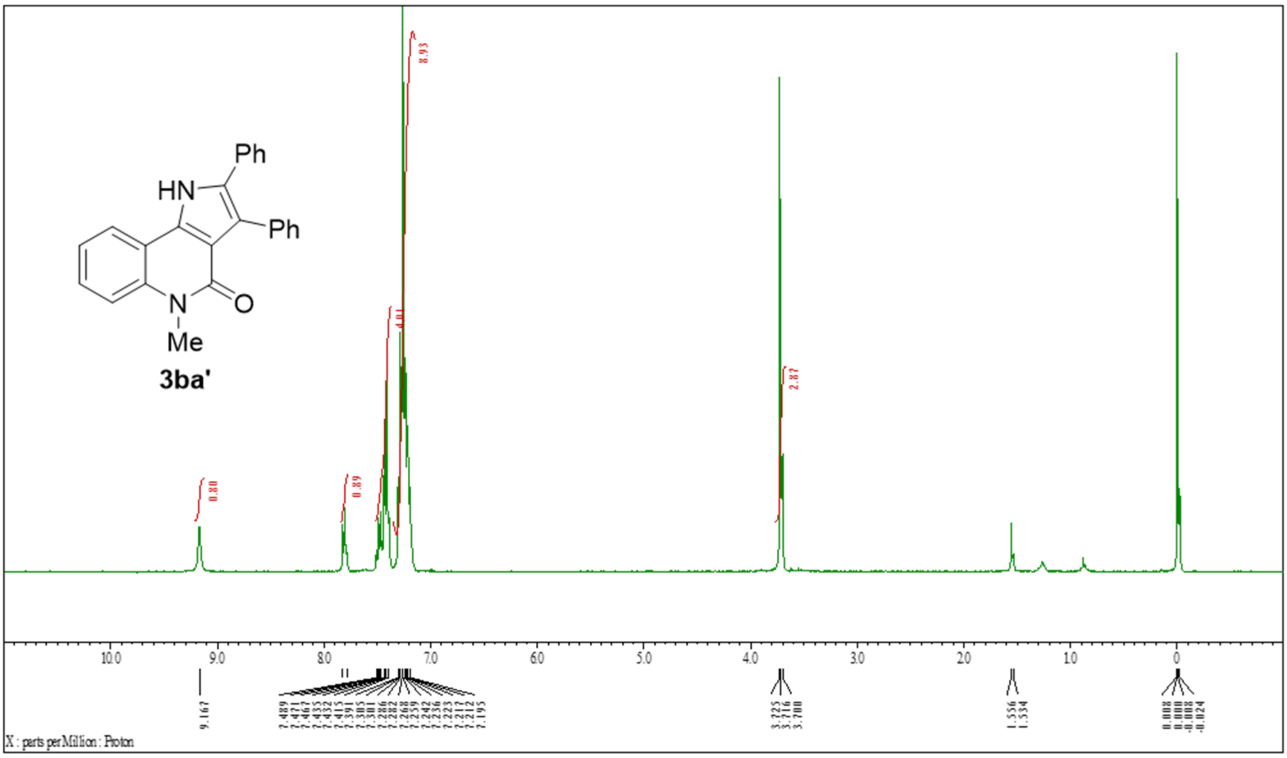

^{13}C NMR (100 MHz, $CDCl_3$) of 3ba'

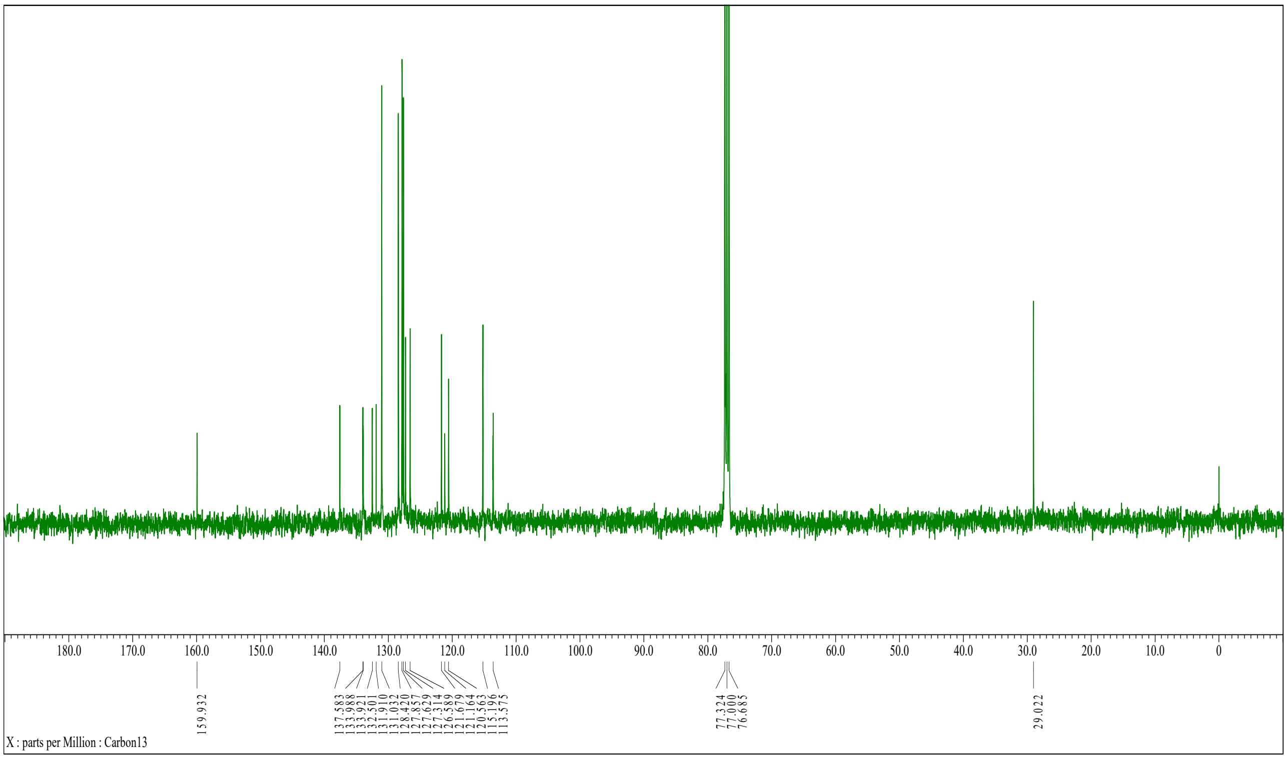

^{1}H NMR (400 MHz, $CDCl_3$) of 4ba

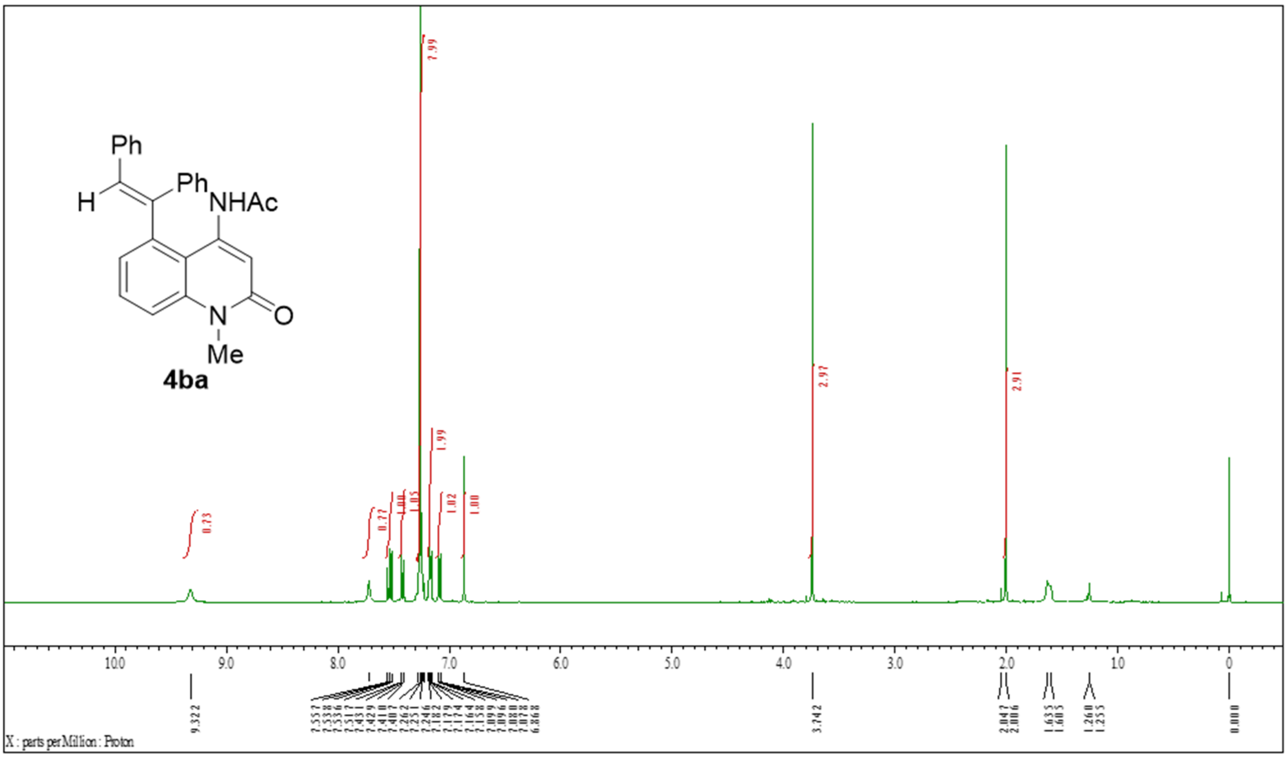

^{13}C NMR (100 MHz, $CDCl_3$) of 4ba

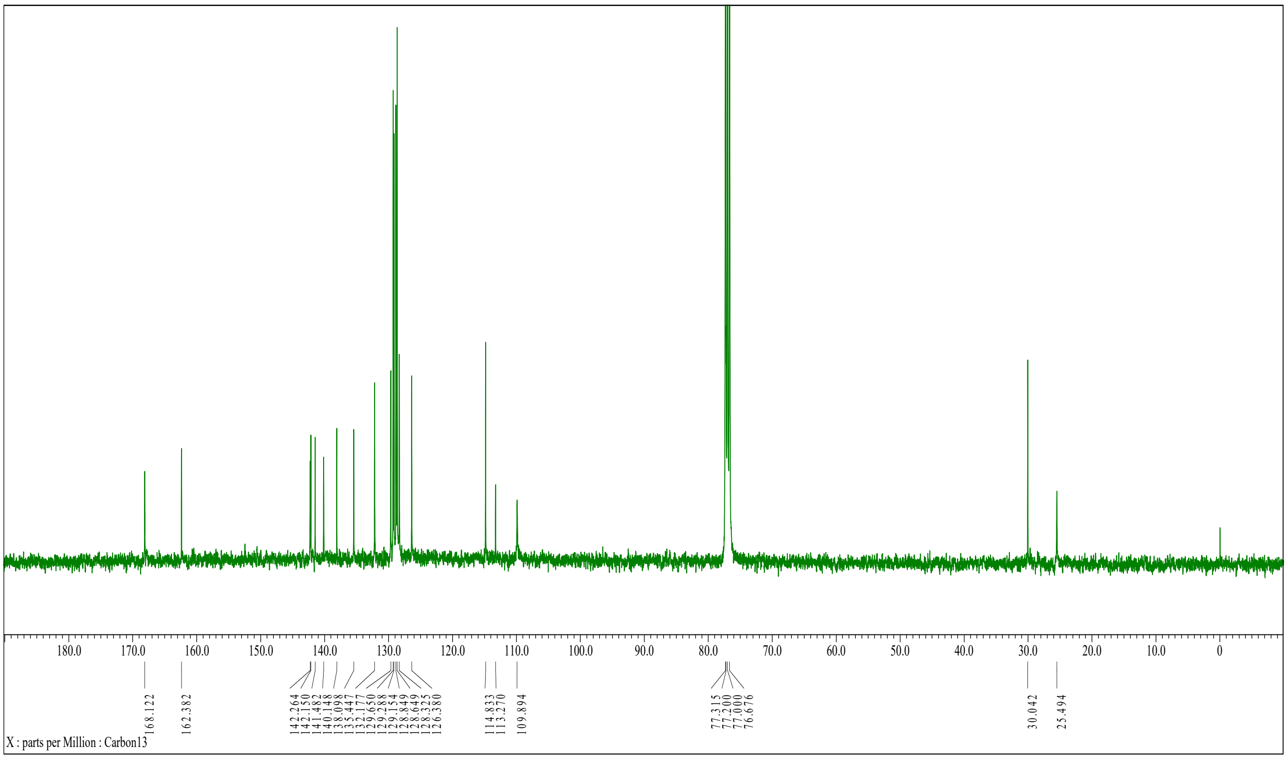

^{1}H NMR (400 MHz, $CDCl_3$) of 5aa

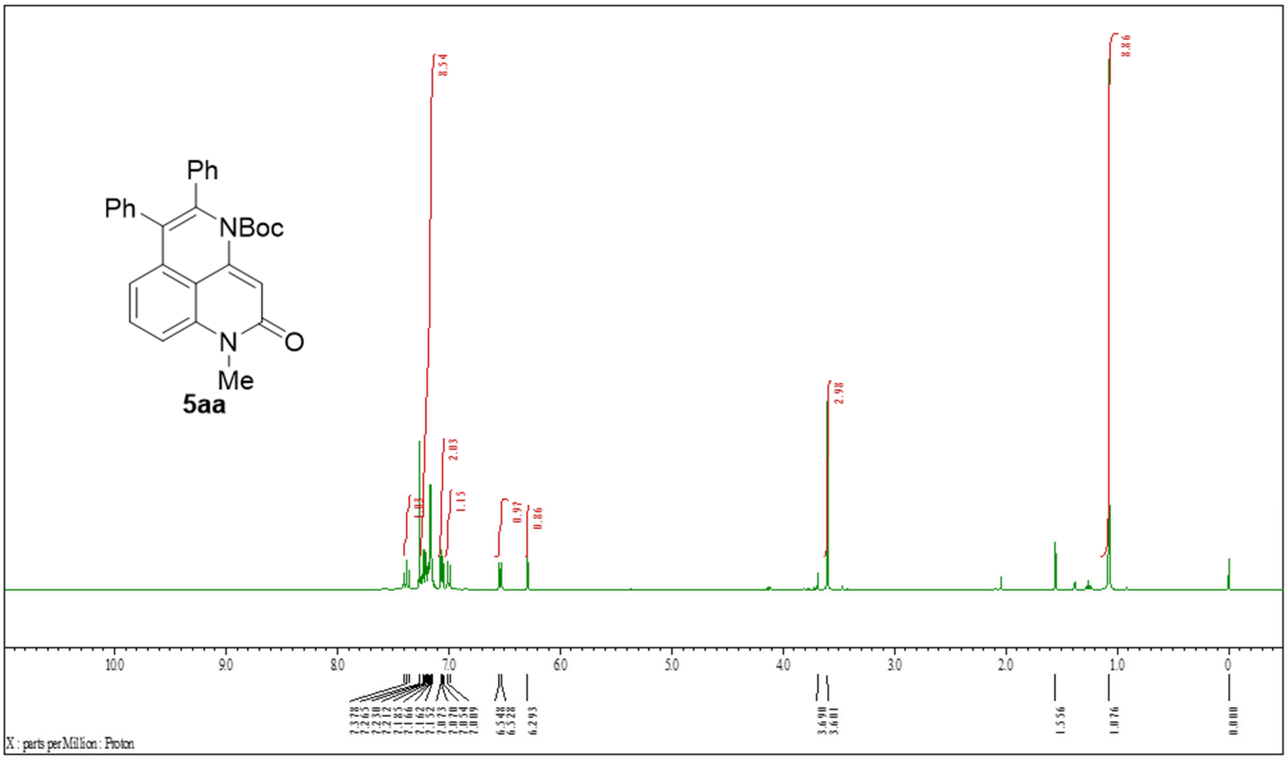

^{13}C NMR (100 MHz, $CDCl_3$) of 5aa

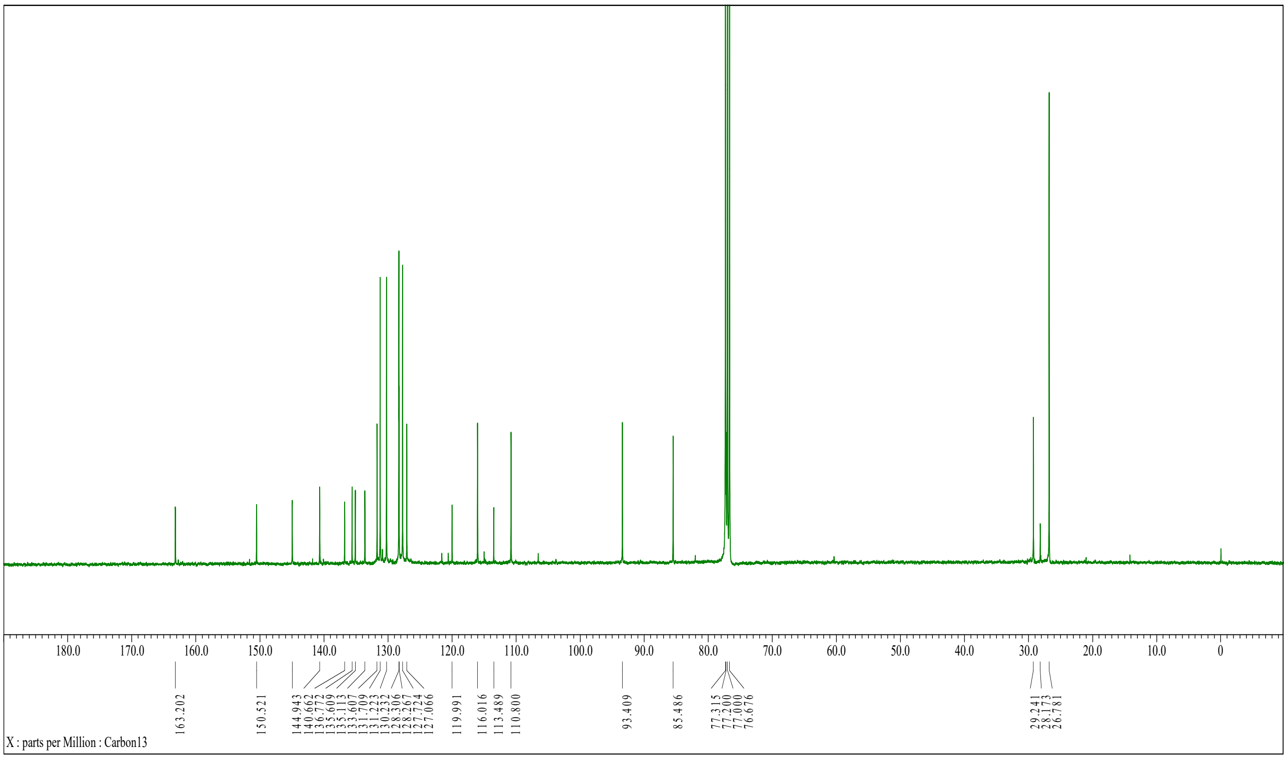

^{1}H NMR (400 MHz, $CDCl_3$) of 5ba

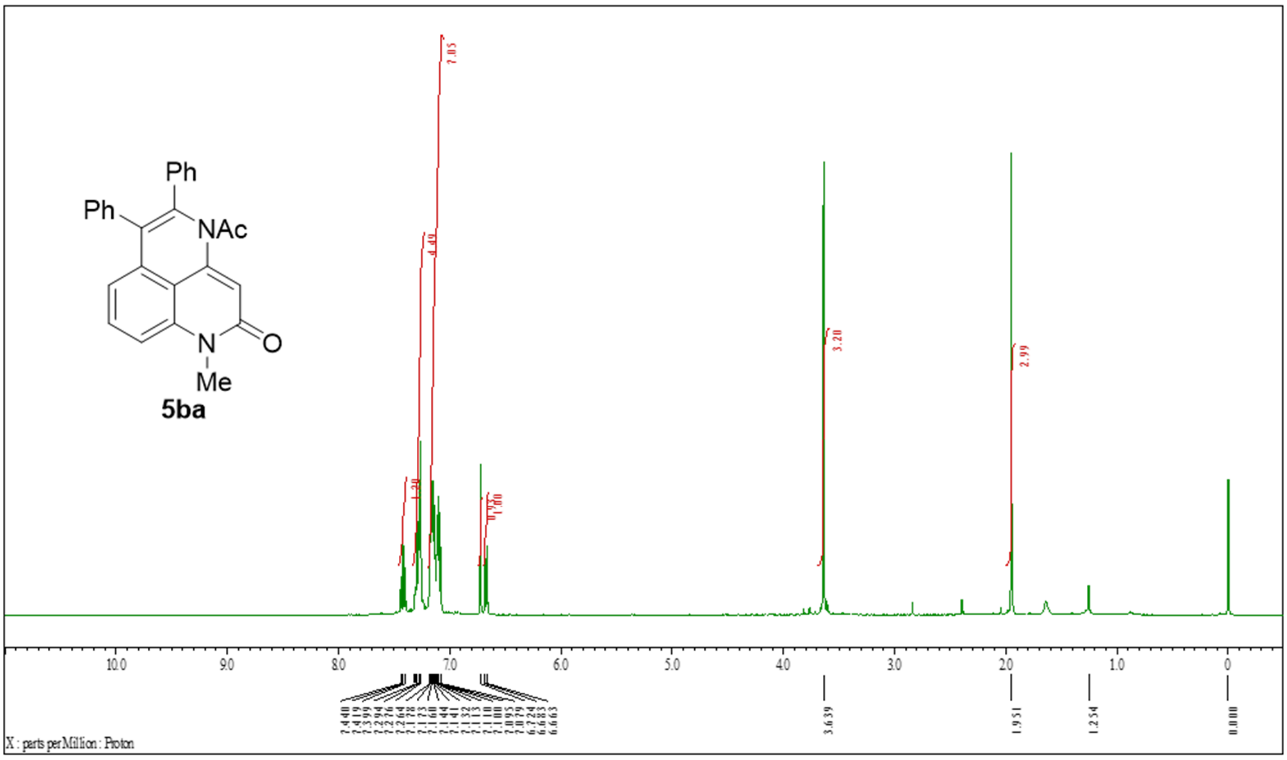

^{13}C NMR (100 MHz, $CDCl_3$) of 5ba

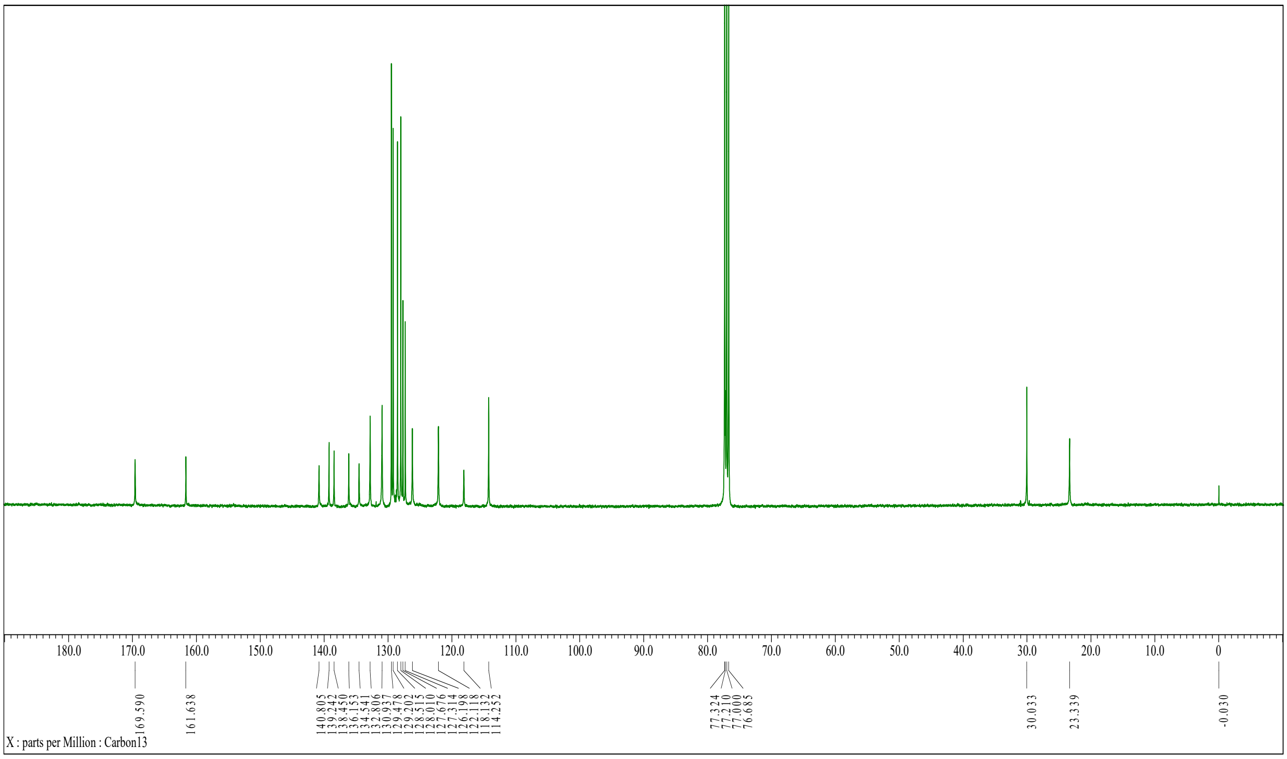

^{1}H NMR (400 MHz, $CDCl_3$) of 5ha

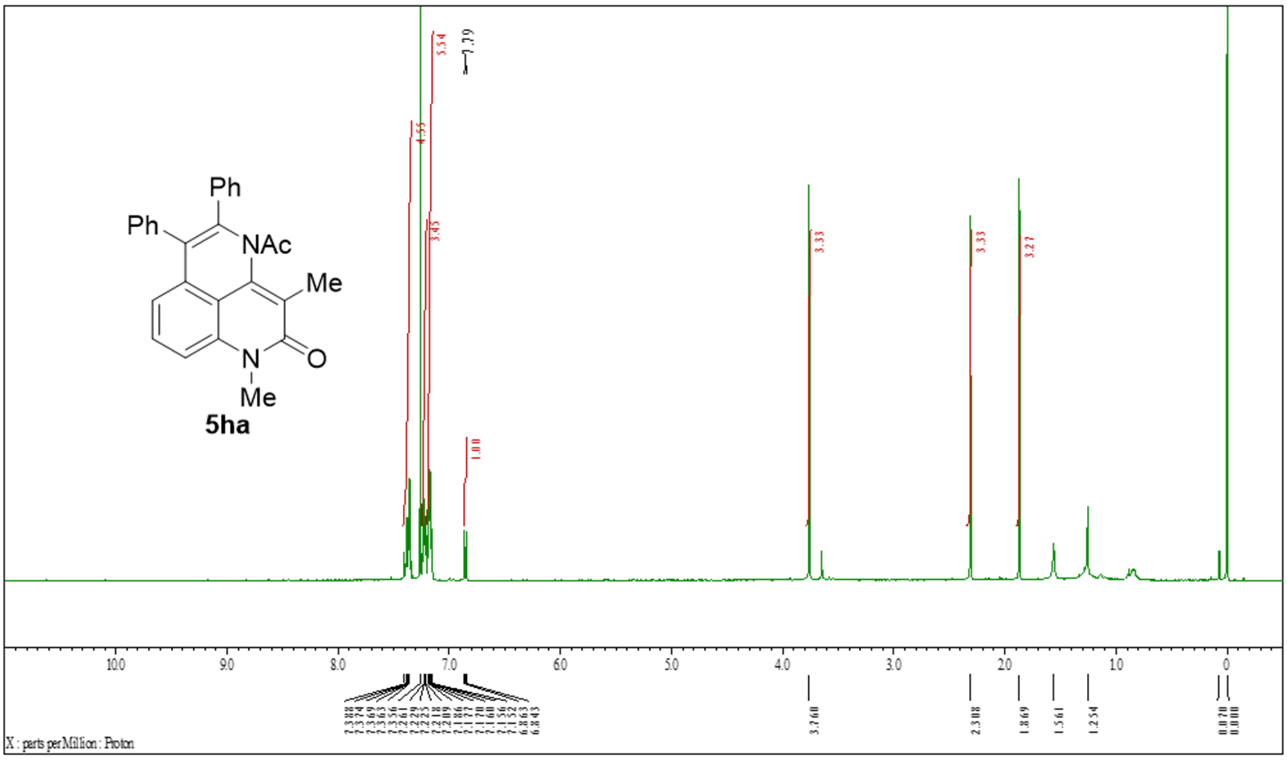

^{13}C NMR (100 MHz, $CDCl_3$) of 5ha

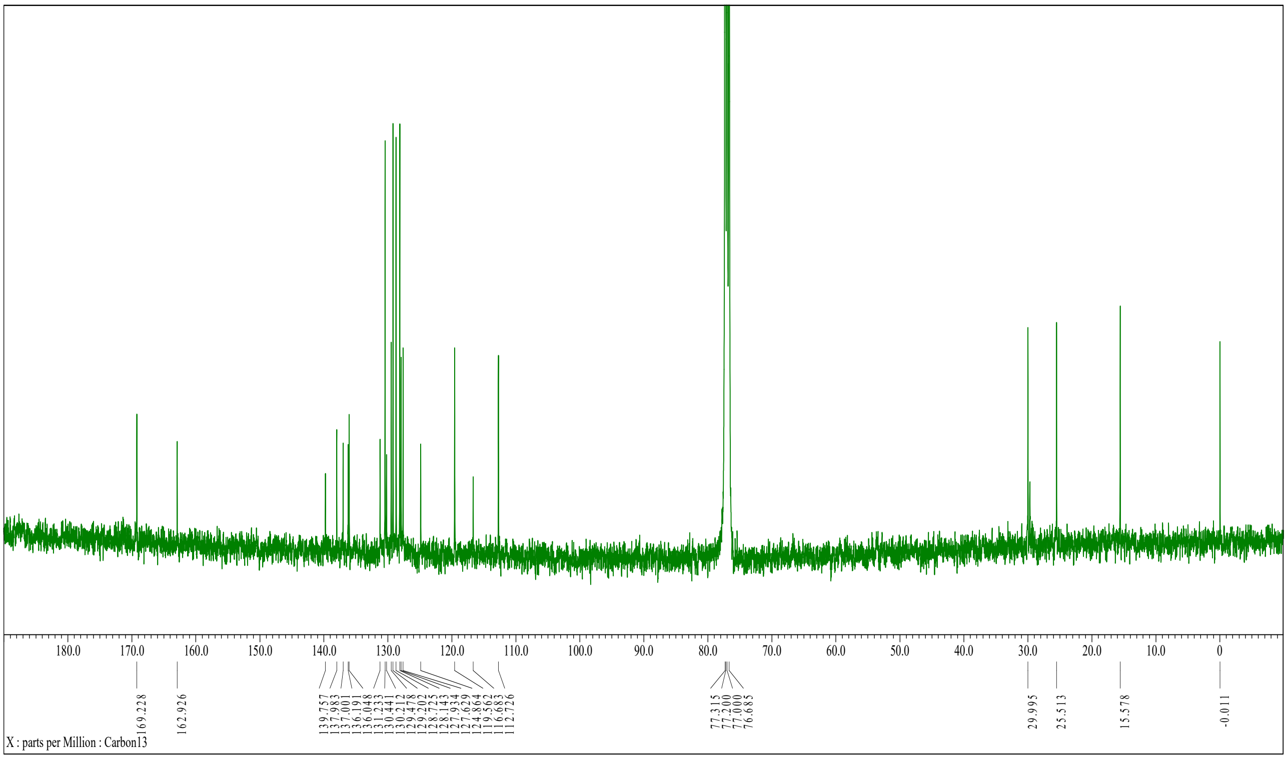

^{1}H NMR (400 MHz, $CDCl_3$) of A

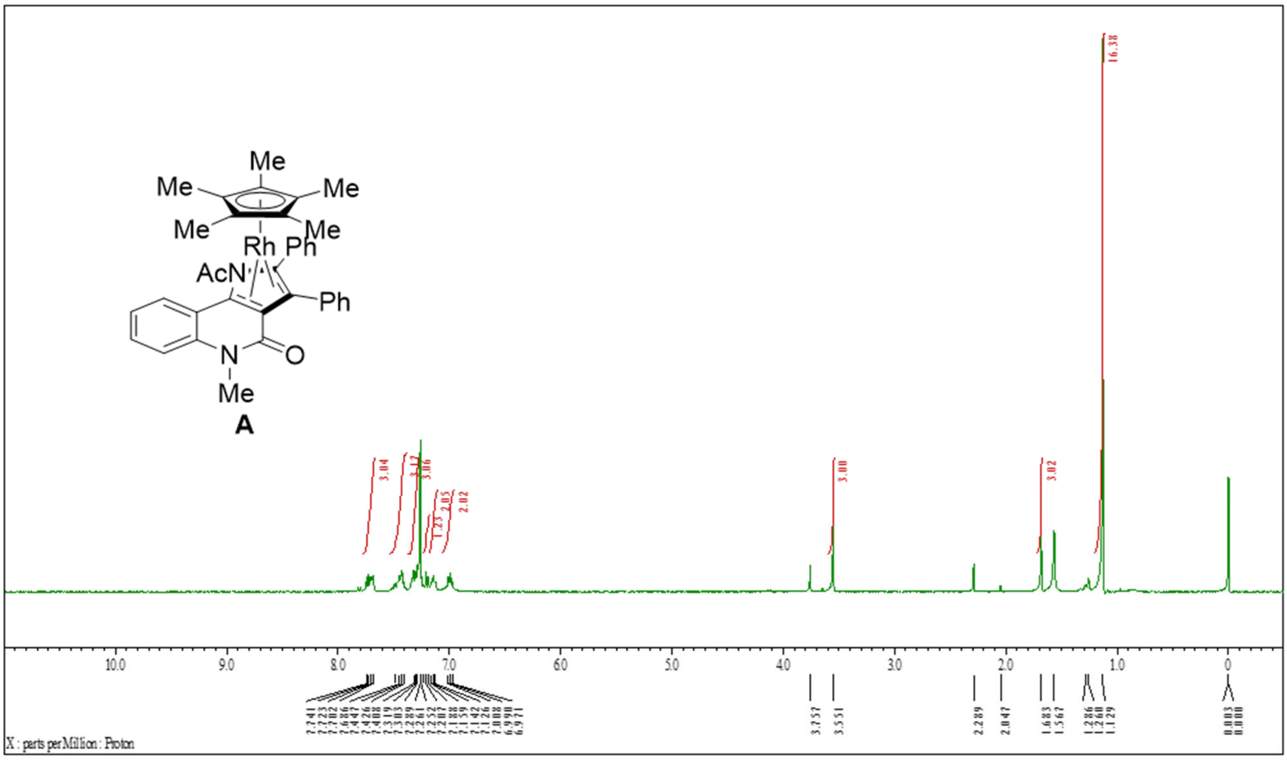

^{1}H NMR (400 MHz, $CDCl_3$) of B-Me

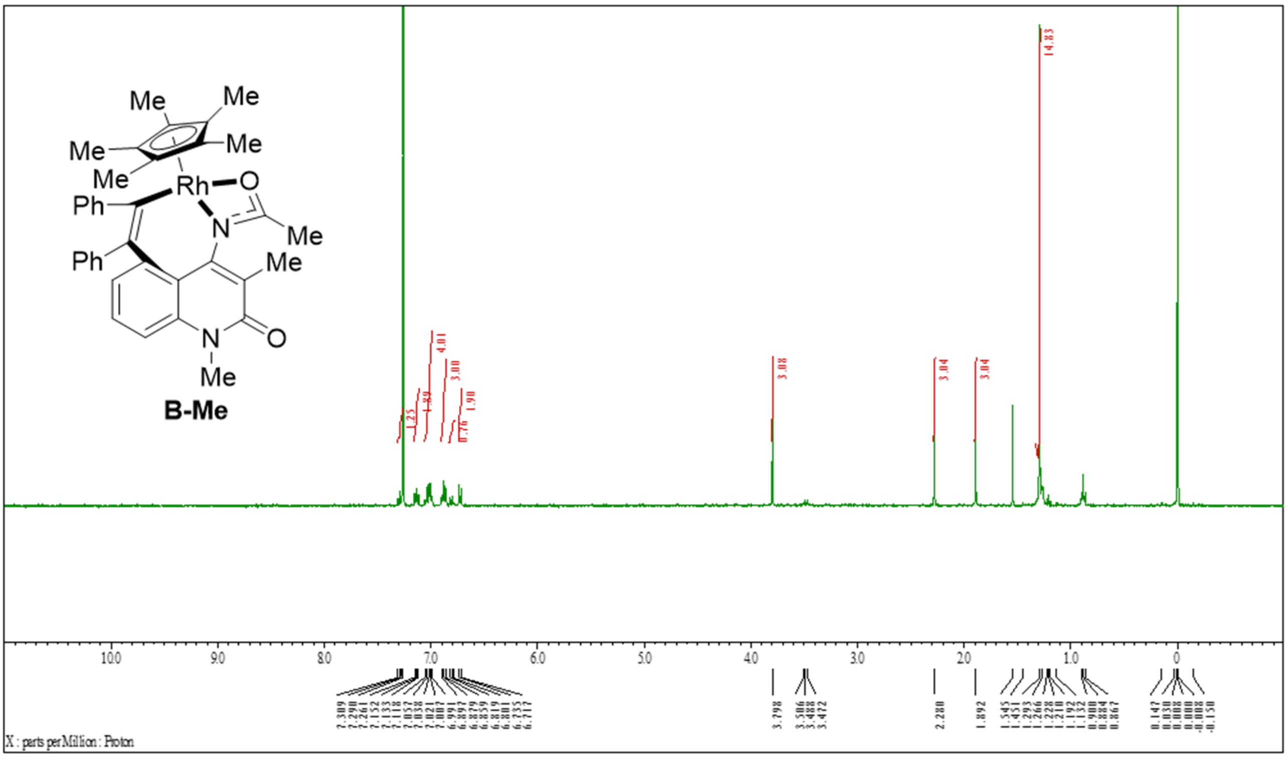

Supplement: SC-014-D3SC03987K-s001 [file SC-014-D3SC03987K-s001.pdf]
